# Will it gel? Successful computational prediction of peptide gelators using physicochemical properties and molecular fingerprints†

**DOI:** 10.1039/c6sc00722h

**Published:** 2016-04-13

**Authors:** Jyoti K. Gupta, Dave J. Adams, Neil G. Berry

**Affiliations:** a Department of Chemistry, University of Liverpool Liverpool L69 7ZD UK d.j.adams@liverpool.ac.uk ngberry@liverpool.ac.uk

## Abstract

The self-assembly of low molecular weight gelators to form gels has enormous potential for cell culturing, optoelectronics, sensing, and for the preparation of structured materials. There is an enormous “chemical space” of gelators. Even within one class, functionalised dipeptides, there are many structures based on both natural and unnatural amino acids that can be proposed and there is a need for methods that can successfully predict the gelation propensity of such molecules. We have successfully developed computational models, based on experimental data, which are robust and are able to identify *in silico* dipeptide structures that can form gels. A virtual computational screen of 2025 dipeptide candidates identified 9 dipeptides that were synthesised and tested. Every one of the 9 dipeptides synthesised and tested were correctly predicted for their gelation properties. This approach and set of tools enables the “dipeptide space” to be searched effectively and efficiently in order to deliver novel gelator molecules.

## Abstract 2

We have successfully developed computational models, based on experimental data, which are robust and are able to identify *in silico* dipeptide structures that can form gels.

## Graphical abstract

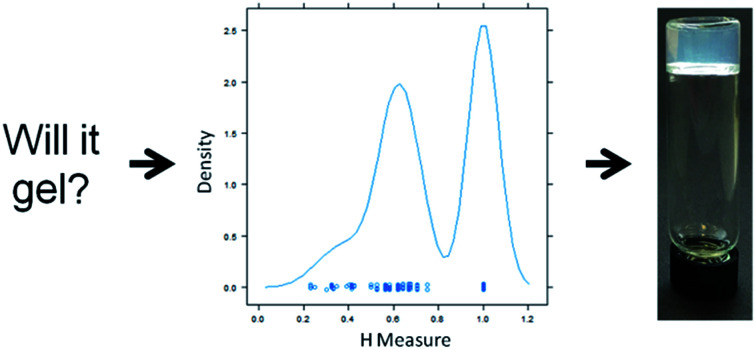

## Introduction

Supramolecular hydrogels are formed when low molecular weight gelators (LMWGs) self-assemble in solution to form fibrous structures.^1–3^ These gels have interesting properties. For example, self-supporting gels are often formed at very low concentrations of gelator (typically less than 1 wt%), and the gels are reversible, returning to the solution state on heating. There are many applications of these gels, from sensing, cell culturing and electronics, all of which require not just that a gel is formed, but often that the gelator contains specific functional groups.^4–6^ Whilst there is significant current interest in these materials, progress is perhaps most hampered by the lack of design rules for these gelators.^2,7^ An extremely large number of effective gelators are known, with a wide diversity of molecular structures. However, *a priori* design rules are few and far between and the majority of gelators are still discovered by serendipity or by close structural changes to a known gelator.^8^ Despite a number of pioneering reports where libraries of molecules have been formed by varying the molecular structures, it is also the case that many close structural analogues do not form gels.^9–11^ The reason for this is not clear, but is undoubtedly due to the fact that the self-assembly leading to gelation arises from a fine balance of non-covalent interactions. Hence, slight modifications in these interactions can very easily tip a gelator into becoming a non-gelator. This is perhaps most easily seen by the fact that each gelator is normally capable of gelling only a small range of solvents.^2^

A number of approaches have been used in an attempt to elucidate design rules. As mentioned above, library-based approaches have been used which usually comprises of synthesis of large numbers of closely related analogues. Other attempts have been made using structural-based design.^8^ Here, specific functional groups are included in a molecule to drive one-dimensional assembly, whilst restricting crystallisation. Recent work has attempted to rationalise gelation with specific solvation properties.^10,12–15^ However, *a priori* prediction of gelation is not possible using this approach as clearly not every molecule with specific Hammett parameters (for example) are gelators. Elsewhere, a number of groups have mined the Cambridge Crystallographic Structural Database for molecules with specific types of interaction.^16,17^ However, where specific moieties or parent structure are required in a gelator, this can present a considerable synthetic challenge to accommodate the desired functional group(s). Clearly, there are then a limited number of structural permutations that are possible whilst maintaining these groups. As such, arguably the most effective currently available option is a library approach.

One approach that has not received much traction to date is the use of computational approaches to predict the gelation ability of specific molecules. Very recently, Tuttle's group have examined the aggregation behaviour of dipeptides and tripeptides and successfully predicted the ability of these molecules to form gels.^18^ This is a major step forward; with 8000 possible tripeptides, this approach saves significant synthetic effort. Here, we present a tool that enables researchers to obtain high quality predictions for the propensity of a compound to form a gel. Employing this approach will greatly expedite the discovery of novel gelators compared with the traditional empirical approach. We have focussed on one family of gelator, functionalised amino acids and dipeptides.^19,20^

Quantitative structure–property relationships (QSPR) is a technology which links measured properties to compound chemical structure. It has proven successful in many aspects of molecular design particularly in the fields of drug discovery and crop protection. Indeed, several marketed drugs have been developed with the aid of such approaches.^21^ QSPR is based on the principle that experimentally measured endpoints are a function of molecular properties.^22^ QSPR models cannot be built directly but rather the molecules' properties are encoded as descriptors, which capture numerically the chemical information of the molecule for computational processes. Molecular descriptors can be classified into zero-dimensional (0D)-descriptors (*e.g.* molecular weight), 1D-descriptors (*e.g.* counts of certain molecular fragments) and 2D-descriptors (*e.g.* molecular constitution in terms of atom types and their connectivity^23^). Statistical and machine learning methods, such as Bayesian modelling, random forests and support vector machines, are employed to link these descriptors to the measured endpoint, *i.e.* gelation.^24^ A successful QSPR model will shed light on the key molecular characteristics that are linked to the gelation ability of a compound and also, crucially, enable rapid computational screening of libraries of molecules to identify candidates that are likely to possess the desired gelation properties.

Designing molecules with the desired physical and chemical properties for a particular application is a huge challenge. If reliable computational predictive methods can be realised then virtual screening of large *in silico* databases is possible, enabling rapid identification of candidates for experimental confirmation.^25^ Here we describe how computational models are built which link the real-world measured endpoint, *i.e.* gelator or non-gelator, to molecular structure.

## Experimental

### Synthesis & testing

The functionalised amino acid and dipeptide library examined here is prepared from previously reported compounds,^9,26–30^ as well as a number of new molecules. The full synthetic and characterisation details for the new molecules are described in the ESI.†

### Gelation testing was carried out using standard protocols

10 mg of the functionalised dipeptide was suspended in deionized water (2 mL) and an equimolar amount of NaOH added. The solution was stirred until a clear solution formed. The pH of the solutions was typically between 10 and 12. To adjust the pH, glucono-δ-lactone (GdL, 8.7 mg mL^−1^) was added to the solution. The sample was left to stand undisturbed overnight. After this time, a “yes” or a “no” was recorded based on the gelation ability of the samples. “Yes” refers to the formation of self-supporting gel (this was assessed after around 18 hours; further long term studies were carried out) and “no” refers to where no gel was formed. A small number of examples where a clear outcome was not reached (for example, a very weak material which was clearly structured, but was not self-supporting) were discounted from the study. These included 2-(2-(6-bromonaphthalen-2-yloxy)acetamido)propanoic acid^26^ and (2-((4-chloronaphthalen-1-yl)oxy)acetyl)phenylalanine.

### QSPR

The molecules described above were generated *in silico* using ChemDraw,^31^ converted to SMILES format, the descriptors were calculated using Pipeline Pilot.^32^ The Caret (Classification and Regression Training)^33^ library in R^34^ was used for both the visualisation and machine learning methods. The MODI index^35^ was calculated using our own scripts in R. We chose *H* measure as our metric as it has recently been shown that the most popular measure of classification models, under the curve (AUC), is fundamentally incoherent, in that it treats the relative severities of misclassifications differently when different classifiers are used. The *H* measure does not have these inadequacies.^36^ The domain of applicability of a model was considered using the “model applicability filter” in Pipeline Pilot tracking property ranges and using OPS analysis. Settings for all methods were default unless otherwise specified. The virtual library was generated in Chemdraw^31^ and SmiLib, using the SMILES code to enable fast generation of the library containing all the possible compounds that fit into our desired category^37^ (see ESI for further details†).

## Results and discussion

### Synthesis & testing

**Fig. 1:**
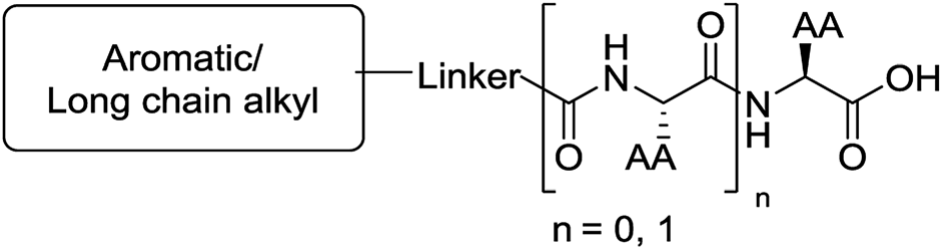
Generic structure of library (AA – amino acid); see ESI† for specific structures.

The functionalised dipeptide library examined here is prepared from previously reported compounds as well as a number of new molecules (see ESI† for all compounds and synthetic details; generic structure shown in Fig. 1).

In all cases, gelation was tested using a pH triggered approach, where we have used the hydrolysis of glucono-δ-lactone (GdL) to gluconic acid^38^ as described elsewhere to lower the pH of a solution of each potential gelator at pH 11 to around 4.^39^ The method by which gelation is triggered can strongly affect the ability of a molecule to form a gel, as well as the mechanical properties of the resulting gel.^40^ As such, we have focussed on molecules synthesised and tested by ourselves, such that we can be certain that the protocol followed was identical in each case. A slow pH change was chosen as this removes issues with stirring and mixing often associated with pH-triggered gelation.^39^

For categorisation assessment after 18 hours, the materials were classified by whether a self-supporting gel had formed or not (“yes” or “no” respectively). A “yes” means that a fully self-supporting gel was formed after around 18 hours. These gels were translucent, transparent, or turbid. A “no” means that no self-supporting gel was formed, with the sample usually being a fine powderous precipitate or a crystalline precipitate. In a small number of cases, a very weak material was formed, and these were discounted from the study as not giving a clear answer. We have focussed here on a single concentration of each potential gelator (5 mg mL^−1^); in our experience, this is always above the minimum gelator concentration (mgc) for this family of materials.^26,28,29^ As such, we do not believe that the use of this concentration is restrictive. Since we are interested in whether or not a gel is formed, as opposed to the specific properties of the resulting gels, we have not attempted to measure the mgc of the gelators, nor the mechanical properties of the resulting gels.

### Gelators and non-gelators

We have compiled sets of data consisting of (i) a training set of 34 compounds (17 gelators, 17 non-gelators) to build the predictive models, (ii) a test set 21 compounds (4 gelators, 17 non-gelators) to test the prediction ability of the models and (iii) an external validation set of 9 compounds (4 gelators, 5 non-gelators). The complete list of compounds and gelation properties is shown in the ESI (Table S1†).

### Predictive QSPR modelling

**Fig. 2:**
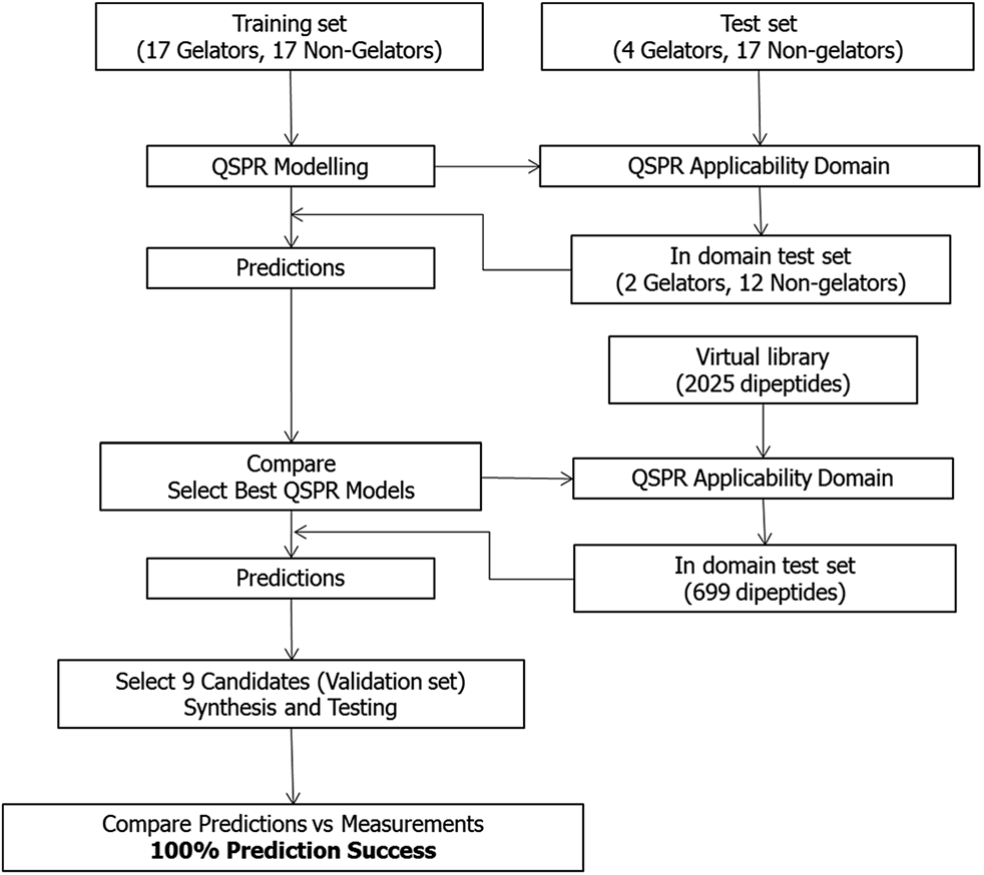
Overall QSPR modelling, synthesis and testing workflow.

No simple relationship was found between the descriptors and gelation properties using visualisation and data compression techniques (see ESI† for full discussion). We therefore developed QSPR classification models. These models are a more complex approach to linking the molecular descriptors with gelation ability than the visualisation approaches above. These models would ideally be able to successfully predict the gelation properties of dipeptides from their structural characteristics alone. The overall workflow of the QSPR modelling is shown in Fig. 2.

Before comprehensive QSPR modelling was undertaken, an assessment of the “modelability” of the training set data was performed using the MODI index.^35^ This index estimates the feasibility of obtaining predictive QSPR models from a binary classified data, *i.e.* gelators and non-gelators. If the MODI statistic is >0.65, then the data should be amenable to classification modelling. Both the training (MODI = 0.76) and test sets (MODI = 0.70) met this criterion. The computational QSPR models were generated using a variety of machine learning methods: Support Vector Machines (SVM),^41^ Random Forests (RF),^42^*k* nearest neighbours (*k*NN), Neural Networks (NN),^43^ Partial Least Squares (PLS),^44^ Naïve Bayesian (NB)^45^ and C5.0.^46^ All these modelling methods employed used both physicochemical descriptors and molecular fingerprints to capture molecular properties.

We employed several modelling techniques as each technique has its own strengths, and ultimately we want to deploy a set of models for making predictions on molecules yet to made and tested based on predictions that they would form a gel. Through a consensus of predictions (from several QSPR models), there can be a dramatic increase in the quality of virtual screening outcomes. Such a virtual screening approach using many robust models can show improved performance over single model predictions^47^ due to fact that the mean of repeated samplings is closer to the true value than one single measurement. Also, different methods *in silico* agree more on the ranking of “actives” than “inactives”, which arises from the fact that different ligand-based virtual screening protocols focus on different aspects of the ligand thus lead to different false positives. In the realm of drug discovery, it has been suggested that actives are clustered more tightly than inactives; thus, multiple samplings will recover more actives than inactives.

A repeated 5-fold cross-validation approach was used to select the optimal QSPR model for each method based on the largest *H* measure value. An ideal model has a *H* measure value of 1, with a random model taking a value of 0.5. Using a cross-validated approach gives a good estimate of the predictive power of the models.^48^ The models generated from each machine learning method with associated statistics are shown in Table 1. Once the optimal model had been selected, we further assessed the models' merits using a range of measures, Cohen's kappa, balanced accuracy and *H* measure (Table 1). We chose Cohen's kappa^49^ as a figure of merit due to its ability to assess the actual agreement of outcomes compared with chance agreement (kappa can range between −1 and +1 with a perfect model having a value of +1). As can be seen, the kappa values are very good for all models (>0.4).

**Table 1:** Optimisation and performance statistics of the QSPR models developed for the training set

Method	Resampling results of optimal model	Performance of optimal model on training set				
	*H* measure ± SD	Kappa	Balanced accuracy	*P* value	*H* measure	Overall quality of model
SVM	0.764 ± 0.28	0.941	0.971	2.04 × 10^−9^	1	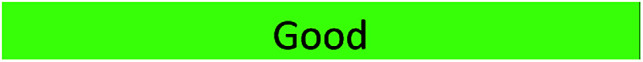
RF	0.771 ± 0.22	0.941	0.971	2.04 × 10^−9^	1	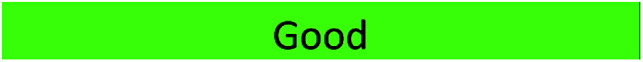
*k*NN	0.570 ± 0.26	0.824	0.912	3.83 × 10^−7^	0.738	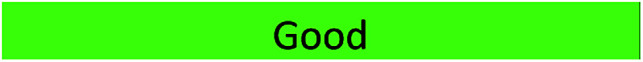
NN	0.774 ± 0.24	0.941	0.971	2.04 × 10^−9^	0.907	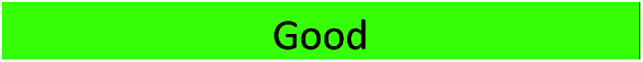
PLS	0.751 ± 0.22	0.529	0.765	1.47 × 10^−3^	0.761	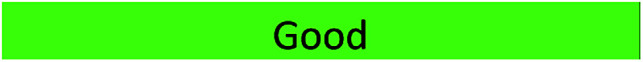
NB	0.701 ± 0.24	0.765	0.882	3.08 × 10^−6^	0.761	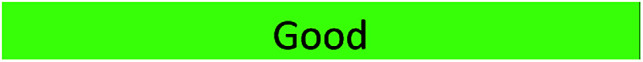
C5.0	0.646 ± 0.25	1	1	5.82 × 10^−11^	1	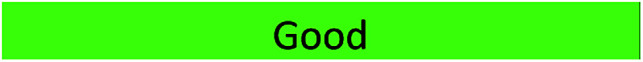

Balanced accuracy is a measure of the number of correctly classified molecules and can vary between 0 and 1 with an ideal model having a value of 1 and an acceptable value being >0.7. An assessment of the probability of the model found being better than the no-information rate (the accuracy rate that can be achieved without a model^48^ has been made and the very small values (<1 × 10^−5^) adds further strength that these models are good. Overall, it can be seen that the models developed are defined as “good” passing all of the desired criteria (*H* > 0.6, kappa > 0.4, balanced accuracy > 0.7, *P* value < 1 × 10^−5^).

The only way to truly assess the true predictive power of a model is to use the models developed on a set of compounds that the model has never seen before. When using models to make predictions, it is vital that the models are applied to molecules that are within the applicability domain of the model, as previously mentioned.^25^ This means that the chemistry of the molecule that one is making a prediction on is not too dissimilar from what the model has encountered previously. Hence, we applied the models to a test set of functionalised dipeptides (see ESI† for structures).

Of the 21 compounds in the test set, 14 (2 gelators, 12 non-gelators) lay within the “applicability domain” of the model as defined by the descriptors (physicochemical and fingerprint) used in the model building (see Experimental section).

The data in Table 2 indicates the overall performance of all the models to predict correctly the gel forming properties this test set of compounds. As can be seen, three models satisfy the criteria as described above for a “good” model. They are random forest, support vector machine and neural network.

**Table 2:** Performance on the models predicting the gelator properties of the 12 external test set compounds within the model domain of applicability. Green – meets criteria. Red – fails criteria. (Criteria for good: kappa > 0.4, balanced accuracy > 0.7, *H* > 0.6)

Method	Performance on external test set of 14 compounds in models applicability domain			
	Kappa	Balanced accuracy	*H* measure	Quality of predictions
SVM	0.417	0.708	0.703	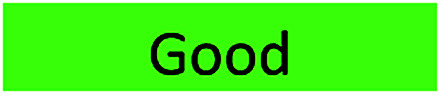
RF	0.759	0.958	1.000	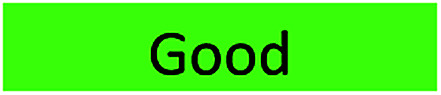
*k*NN	0.286	0.7941	0.311	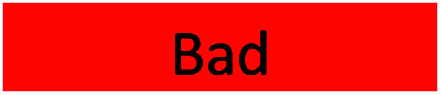
NN	0.462	0.875	1.000	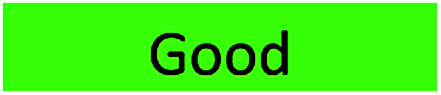
PLS	0.177	0.625	0.526	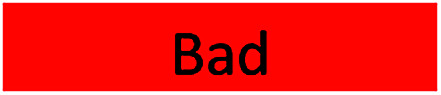
NB	0.286	0.791	0.526	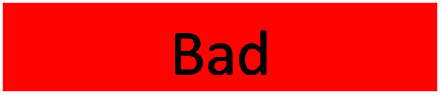
C5.0	0.103	0.583	0.334	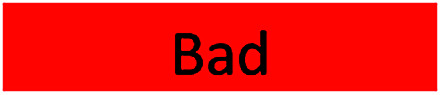

It is notable that *H* measure of the test set is correlated with the *H* measure from repeated cross-validation during model building (*r*^2^ = 0.727) demonstrating that the repeated cross-validation approach did indeed give a good indication on the performance of models on future compounds – thus these models are highly predictive for compounds that the models have never seen before.

**Fig. 3:**
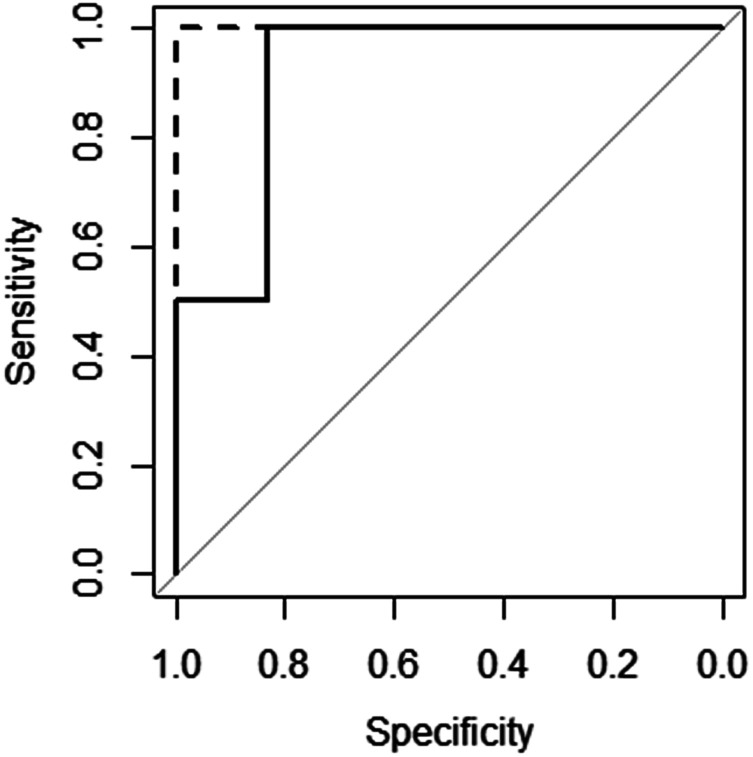
ROC curves for the SVM (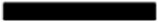), RF (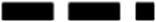) and NN (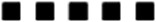) models (RF and NN plots lie on top of each other).

The excellent predictive performance of these models can also be seen in Fig. 3, which displays the ROC (Receiver Operator Characteristic) curves for these models.^50^ The NN model is perfect predicting each molecule's gelation abilities correctly with the RF and SVM models only slightly worse. This is indicated in the plots for RF and SVM diverting away from the vertical line of specificity equal to 1. A model which provides no predictive ability is indicated by the grey line – clearly all three good models are significantly better than this.

In order to increase confidence further in the three predictive models identified, a randomisation test was performed in which the measured gelation outcome for the training set compounds was randomised and the whole model building process repeated as was performed for the true data.^51^ The predictive power of models developed on the randomised data should be markedly inferior to the models developed using the true data. All of the statistical measures (kappa, balanced accuracy and *H* measure) for the performance of the models generated using the randomised data for the predictions of the 12 compounds in the test set are much worse than the equivalent models found using the true data (see Table S4, ESI†). This data further increased our confidence in the good SVM, RF and NN models identified.

Thus, with the set of models (SVM, RF and NN) that were demonstrated to perform excellently in predicting the gelation properties of dipeptides in the test set, we wished to use these models prospectively to identify candidate dipeptides from a large *in silico* library to synthesis and testing. This set of compound would act as a validation set and demonstrate the ability of our approach in successfully identifying both compounds that form gels and those that do not.

### Virtual library design, generation and screening

An *in silico* library of N-protected amino acids and dipeptides was generated with the generic form as shown in Fig. 1. The aromatic/long alkyl chain portion of the dipeptide included 1,2-substituted naphthalenes, 5,6,7,8-tetrahydronapthalenes, carbazole, fluorene, C_15_-alkyl, C_13_-alkyl and substituted aromatic rings. The amino acid (AA) side chains studied were glycine, valine, leucine, alanine, phenylalanine, isoleucine, methionine and tyrosine (see ESI† for full list of aromatics/long alkyl chains and amino acids).

The library in total contained 2025 compounds (ESI, Table S5†), each of which had the same set of descriptors calculated as for the training set of molecules. Even though we had identified three robust models for gelation predictions, these models have limitations. Their predictions will not be equally good for all possible molecules. Generally, the more similar a compound whose properties we wish to predict is to the molecules in a model's training data set, the better we expect the model's predictions to be. In other words, if a sample lies within the model's applicability domain (MAD), we expect the prediction to be trustworthy. If the sample lies outside the MAD, we expect the prediction to be less trustworthy. The MAD for the SVM, RF and NN models was defined using the molecular descriptors calculated (further information in the Experimental section and references therein). For the virtual library of 2025 compounds, those molecules which lay outside the model applicability domain for SVM, RF and NN models were removed, leaving 699 compounds.

For each of the 699 compounds, predictions were made on their gel forming ability using the SVM, RF and NN models. Nine candidate molecules were chosen (4 gelators, 5 non-gelators) to be synthesised and tested using the combined likelihood from the three machine learnt models. As can be seen there is an exact agreement between the predictions and measurements indicating a remarkable predictive power and performance of these models (Table 3). Additionally, it can be seen that the models predict compounds to be gelators where both amino acids are non-aromatic. Typically, these are much less likely to form gels as opposed to those that contain aromatic amino acids.^29^

**Table 3:** Structures of molecules predicted, synthesized and tested for gelation property. % likelihood is the average probability from SVM, RF and NN models that the prediction is as indicated

Compound	Prediction (% likelihood)	Measurement
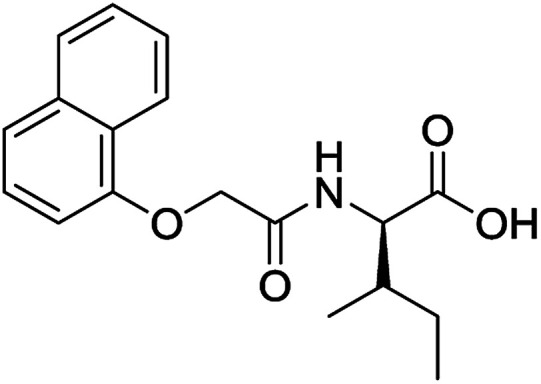	No (85%)	No
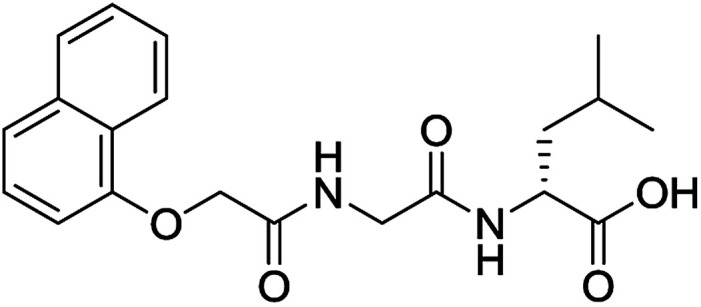	No (85%)	No
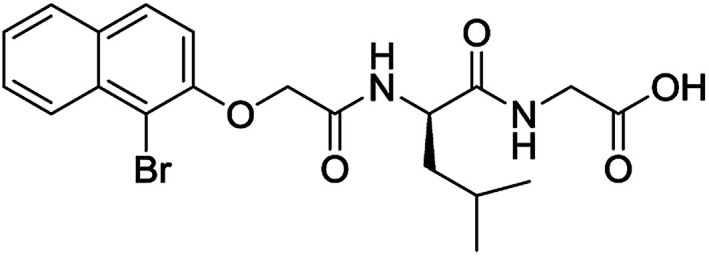	No (85%)	No
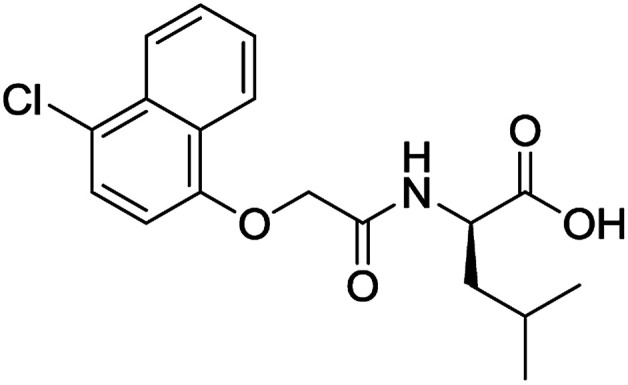	No (82%)	No
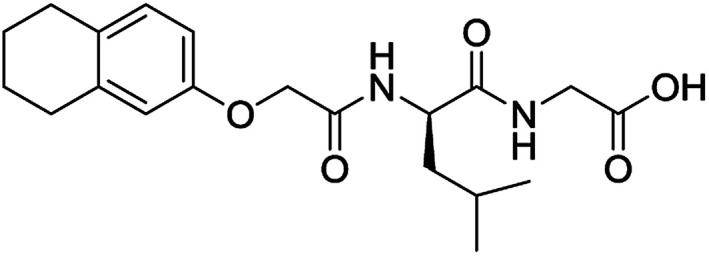	No (83%)	No
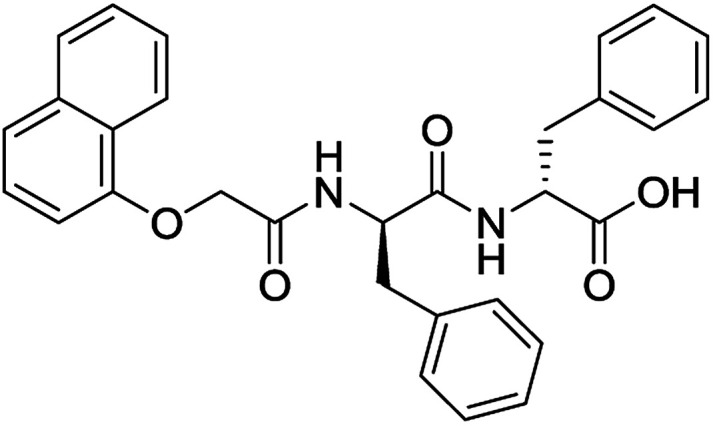	Yes (83%)	Yes
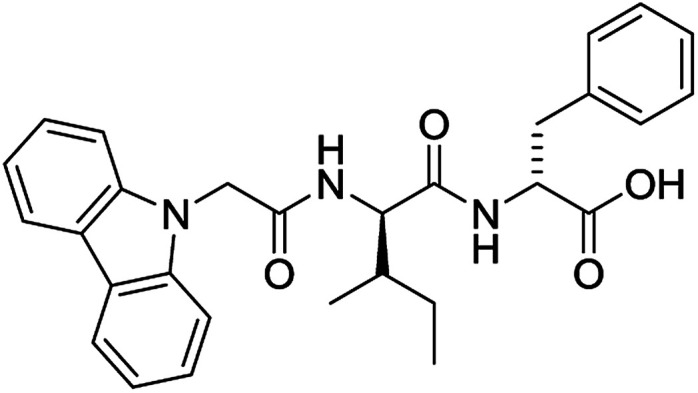	Yes (75%)	Yes
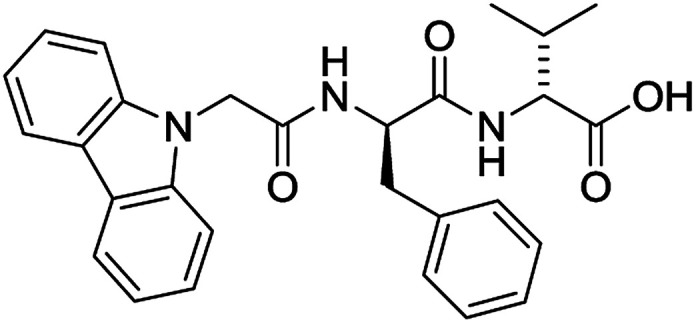	Yes (79%)	Yes
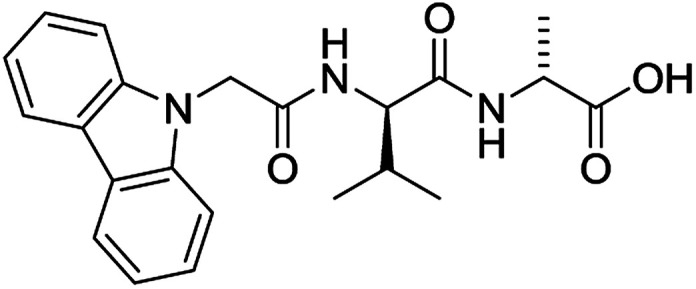	Yes (63%)	Yes

Whilst we stated earlier that to be certain of an identical protocol, we focused on molecules synthesised and tested by ourselves, we have nonetheless applied our protocols to a number of literature examples. A significant number fell outside the applicability domain. However, those that did all followed exactly our predictions. These included Fmoc-GF (predicted not to be a gelator in line with the experimental data^28,52^), as well as two naphthalene-based gelators (Nap–Gly–Val and Nap–Gly–Leu correctly predicted not to form gels^53^), benzimidazole-diphenylalanine (correctly predicted to form gels^54^), and Azo–Phe–Ala (correctly predicted to form a gel^55^).

As noted above, design rules are few and far between for low molecular weight gelators. Examination of the most influential descriptors in these complex models may reveal some key parameters which are highly influential on molecules with gelation ability. Amongst the 12 physicochemical descriptors calculated, five were important – the number of rings, predicted molecular aqueous solubility, polar surface area, solvent accessible surface area, *A* log *P* and number of rotatable bonds. However, for all models (SVM, RF, NN), there were a significant number of molecular fingerprint descriptors that were also very important (see ESI†). Unfortunately, these fingerprint descriptors are difficult to interpret by eye. Rather, the information that is encoded in them is best utilised in a virtual screening campaign, as we successfully employed here.

## Conclusions

In conclusion, we believe we have demonstrated the first successful predictive models of gelation properties of mono/dipeptides. It is clear that complex machine learning based approached are needed in order to make predictions as it is not solely by physical properties of the molecules that govern gelation propensity, but it is more subtle information encoded in the molecules structure. The online tool developed by us, provides predictions for the gelation property of any molecule that is submitted – both those similar and dissimilar to those encountered previously. An indication of the probability (as a percentage) of the prediction of a given molecule is given along with the prediction gelation propensity. In addition to this, the molecule is annotated whether it is within the “applicability domain” of the model. The “applicability domain” is the chemical space in which the predictive model can be used with confidence.

The applicability domain has been defined using the molecular fingerprints and physicochemical properties of each molecule within the training set. If a molecule lies outside of the applicability domain, it does not mean the prediction is incorrect, it just provides the user with extra information with which to make a decision *via* this applicability domain “warning”. These additional features (above a simple yes/no answer) allows the user to make their own informed decision on whether to make and test any given molecule given the predicted likelihood of a molecule forming a gel. We invite researchers to use the online interface through which users can predict the gelation properties under the conditions discussed in this paper, and (www.liv.ac.uk/∼ngberry/gel.html, username Gel, password gel123).

## Supplementary Material

SC-007-C6SC00722H-s001
