# Supplementary material for: Will it gel? Successful computational prediction of peptide gelators using physicochemical properties and molecular fingerprints
Source: Chem Sci. 2016 Apr 13;7(7):4713–9. doi: 10.1039/c6sc00722h (PMC6016447; doi:10.1039/c6sc00722h)
Supplement: SC-007-C6SC00722H-s001 [file SC-007-C6SC00722H-s001.pdf]

Electronic Supplementary Material (ESI) for Chemical Science.
This journal is © The Royal Society of Chemistry 2016

Will it Gel? Successful Computational Prediction of Peptide Gelators using Physicochemical Properties and Molecular Fingerprints

Jyoti Gupta, Dave J. Adams and Neil G. Berry

Department of Chemistry, University of Liverpool, Liverpool L69 7ZD U.K.

SUPPORTING INFORMATION

Section 1

Materials. A number of the potential gelators have been previously reported (structures **1-25**; Fig. S1; the structures of the commercially available Fmoc-amino acids (purchased from Sigma Aldrich and used as received) are also shown here (structures **26-31**)).[1-8]

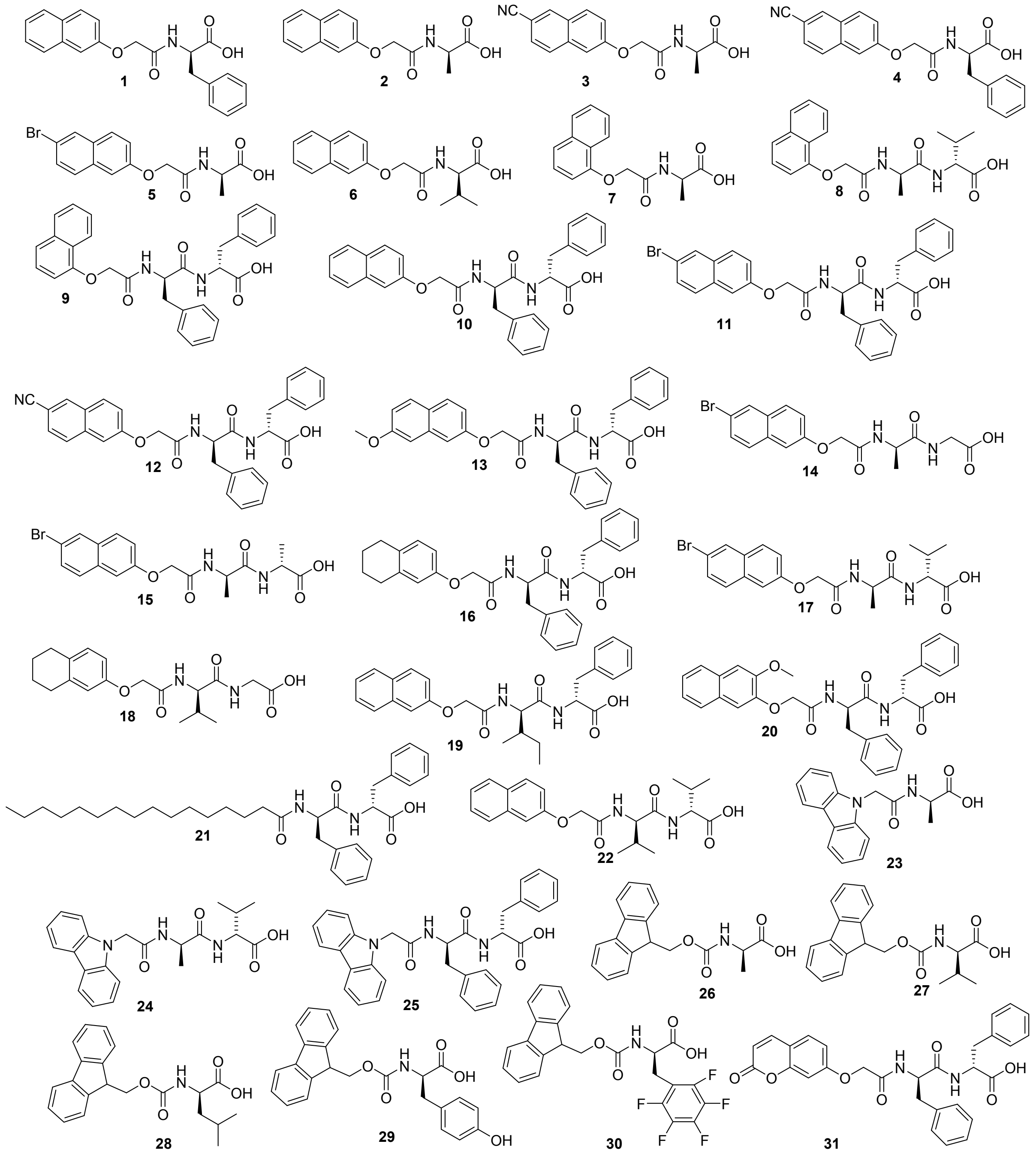

Figure S1. Previously reported LMWG **1-31**.

The other gelators were prepared from the corresponding phenol or naphthol. These were reacted with tert-butylchloroformate and deprotected to give the starting carboxylic acid as

described elsewhere.[1-3,5] Yields were typically > 85 %. This was then coupled either sequentially with C-protected amino acids or with the required C-protected dipeptides in yields of 70 -90%. The C-terminus was then deprotected with lithium hydroxide as described previously to give the final products in yields of > 70%.[1-3,5] Where dipeptides were coupled, these were prepared with the C-terminus protected by methyl or ethyl esters by coupling Boc-protected amino acids with amino acids protected by methyl or ethyl esters, followed by deprotection of the N-terminus with trifluoroacetic acid following a literature procedure, generally providing the products in yields of > 80 %.[9] Because all of the procedures are analogous to those described previously, full experimental protocols are not provided. The characterisation of all intermediates and final tested materials is listed below. The materials tested are labelled with name and number, with the outcome of the gelation tests described below in Section 2.

Characterisation for Dipeptides

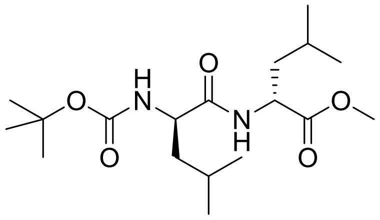

BocLLOMe: ^{1}H NMR ($CDCl_3$) 6.51 (d, NH, 1H, J_{HH} = 7.8 Hz), 4.92 (d, NH, 1H, J_{HH} = 8.3 Hz), 4.60 (dt, CHNH, 1H, J_{HH} = 9.1 Hz, J_{HH} = 5.0 Hz), 4.10 (m, CHNH, 1H),3.72 (s, OCH_3, 3H), 1.75 – 1.46 (m, CH_2 and CH, 6H), 1.44 (s, CH_3, 9H), 0.92 (m, CH_3, 12H) ppm. ^{13}C NMR ($CDCl_3$) 173.8, 172.2, 155.7, 80.1, 69.8, 52.9, 50.6, 54.6, 50.8, 28.3, 24.7, 24.6, 22.1, 21.8, 18.8 ppm. MS (ES) 381 ([M+Na]$^+$). Accurate mass calculated for $C_{18}H_{34}N_2O_5Na$: 381.2365. Found: 381.2374.

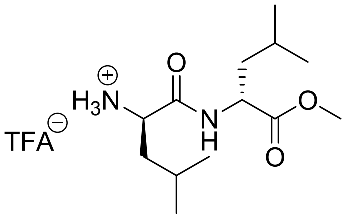

LLOMe.TFA: ^{1}H NMR (DMSO) 8.84 (d, NH, 1H, J_{HH} = 7.5 Hz), 8.22 (bs, NH_3, 3H), 4.34 (m, CHNH, 1H), 3.81 (m, CHNH, 1H), 3.63 (s, OCH_3, 3H), 1.68 – 1.49 (m, CH and CH_2, 6H), 0.91 (m, CH_3, 12H) ppm. ^{13}C NMR (DMSO) 172.3, 169.2, 51.9, 50.6, 50.4, 23.9, 23.3, 22.7, 22.5, 21.9, 21.1 ppm. MS (ES) 259 ([M+H]$^+$).

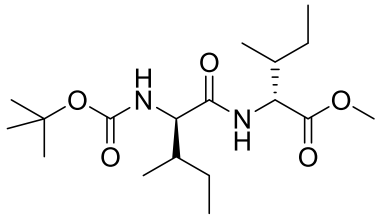

BocIleIleOMe: ^{1}H NMR ($CDCl_3$) 6.38 (bd, NH, 1H, J_{HH} = 7.4 Hz), 5.03 (m, NH, 1H), 4.56 (dd, CHNH, 1H, J_{HH} = 8.6 Hz, J_{HH} = 4.9 Hz), 3.94 (m, CHNH, 1H), 3.73 (s, OCH_3, 3H), 1.90 (m, $CH(CH_3)$, 2H), 1.46 (m, CH_2 and CH_3, 9H), 1.14 (m, CH_2, 2H), 0.93 (m, CH_3, 12H) ppm. ^{13}C NMR ($CDCl_3$) 172.1, 171.4, 155.8, 79.9, 59.3, 56.4, 52.1, 37.8, 36.9, 28.3, 25.1, 24.8, 15.5, 15.4, 11.5, 11.4 ppm. MS (ES) 381 ([M+Na]$^+$). Accurate mass calculated for $C_{18}H_{34}N_2O_5Na$: 381.2365. Found: 381.2372.

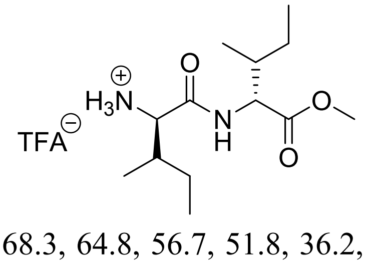

IleIleOMe.TFA: ^{1}H NMR (DMSO) 8.66 (d, NH, 1H, J_{HH} = 4.4 Hz), 8.21 (bs, NH_3, 3H), 4.24 (dd, CHNH, 1H, J_{HH} = 7.2 Hz, J_{HH} = 6.2 Hz), 3.77 (d, CHNH, 1H, J_{HH} = 5.9 Hz), 3.63 (s, OCH_3, 3H), 1.81 (m, CH, 2H), 1.45 (m, CH_2, 2H), 1.12 (m, CH_2, 2H), 0.88 (m, CH_3, 12H) ppm.^{13}C NMR (DMSO) 171.3, 168.3, 64.8, 56.7, 51.8, 36.2,

36.1, 24.7, 23.7, 15.3, 15.1, 11.1, 11.0 ppm. MS (ES) 259.2 ($[M+H]^+$). Accurate mass calculated for $C_{13}H_{27}N_2O_3$: 259.2022. Found: 259.2027.

Phenyl-protected Amino Acids and Dipeptides

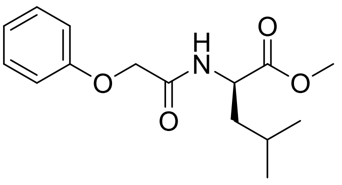

PhOLOMe: ^1H NMR ($CDCl_3$) 7.33 (t, ArH, 2H, J_{HH} = 7.4 Hz), 7.06 (t, ArH, 1H, J_{HH} = 7.4 Hz), 6.96 (d, ArH, 2H, J_{HH} = 9.0 Hz), 6.89 (bd, NH, 1H, J_{HH} =7.9 Hz), 4.73 (m, CHNH, 1H), 4.53 (s, OCH_2, 2H), 3.74 (s, OCH_3, 3H), 1.67 – 1.55 (m, CH and CH_2, 3H), 0.94 (d, CH_3, 3H, J_{HH} = 6.6 Hz), 0.92 (d, CH_3, 3H, J_{HH} = 6.6 Hz) ppm. ^{13}C NMR ($CDCl_3$) 172.9, 168.1, 157.2, 129.8, 122.2, 117.8, 67.3, 52.4, 50.2, 41.5, 24.8, 22.8, 21.9 ppm. MS 280 ($[M+H]^+$). Accurate mass calculated for $C_{15}H_{21}NO_4$: 280.1543. Found: 280.1545.

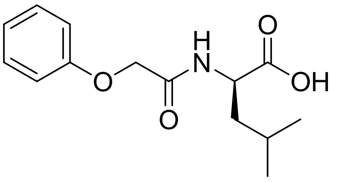

PhOLOH (32): ^1H NMR (DMSO) 8.27 (d, NH, 1H, J_{HH} = 8.2 Hz), 7.29 (m, ArH, 2H), 6.98 (m, ArH, 3H), 4.55 (d, OCH, 1H, J_{HH} = 14.7 Hz), 4.51 (d, OCH, 1H, J_{HH} = 14.7 Hz), 4.31 (m, CHNH, 1H), 1.57 (m, CH and CH_2, 3H), 0.88 (d, CH_3, 3H, J_{HH} = 6.1 Hz), 0.83 (d, CH_3, 3H, J_{HH} = 6.1 Hz) ppm. ^{13}C NMR (DMSO) 173.7, 167.7, 157.7, 129.4, 121.1, 114.7, 66.7, 49.9, 24.3, 22.9, 21.2 ppm. MS (CI) 266 ($[M+H]^+$). Accurate mass calculated for $C_{14}H_{19}NO_4$: 266.1387. Found: 266.1398.

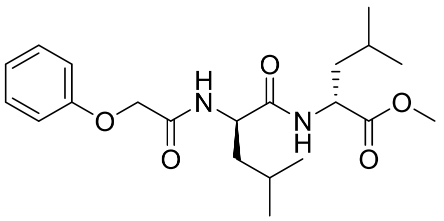

PhOLLOMe: ^1H NMR ($CDCl_3$) 7.30 (m, ArH, 3H), 7.01 (t, ArH, 1H, J_{HH} = 7.4 Hz), 6.92 (m, ARH and NH, 2H), 6.36 (d, NH, 1H, J_{HH} = 8.1 Hz), 4.56 (m, OCH and CHNH, 3H), 4.49 (d, OCH, 1H, J_{HH} = 15.0 Hz), 3.74 (s, OCH_3, 3H), 159 (m, CH and CH_2, 6H), 0.94 (m, CH_3, 12H) ppm. ^{13}C NMR ($CDCl_3$) 173.1, 171.1, 168.4, 157.1, 129.8, 122.3, 114.7, 67.2, 52.3, 51.2, 50.8, 41.4, 40.8, 24.9, 24.7, 22.8, 22.7, 22.1, 21.9 ppm. MS (ES) 415 ($[M+Na]^+$). Accurate mass calculated for $C_{21}H_{32}N_2O_5Na$: 415.2209. Found: 415.2218.

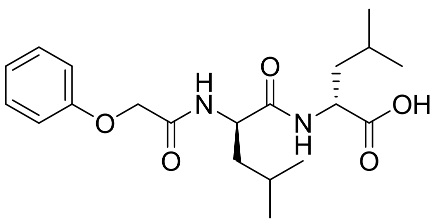

PhOLLOH (33): ^1H NMR (DMSO) 8.18 (d, NH, 1H, J_{HH} = 7.9 Hz), 8.02 (d, NH, 1H, J_{HH} = 8.5 Hz), 7.29 (m, ArH, 2H), 6.95 (m, ArH, 3H), 4.55 (dd, OCH, 1J_{HH} = 14.7 Hz), 4..50 (d, OCH, 1H, J_{HH} = 14.7 Hz), 4.44 (m, CHNH, 1H), 4.21 (m, CHNH, 1H), 1.51 (m, CH and CH_2, 6H), 0.85 (m, CH_3, 12H) ppm. ^{13}C NMR (DMSO) 173.9, 171.7, 167.3, 157.7, 129.4, 121.1. 114.6, 66.6, 50.4, 50.1, 41.0, 24.2, 24.0, 23.1, 22.8, 21.6, 21.3 ppm. MS (ES) 401 ($[M+Na]^+$). Accurate mass calculated for $C_{20}H_{30}N_2O_5Na$: 401.2052. Found: 401.2060.

PhOIOMe: ^1H NMR ($CDCl_3$) 7.18 (bd, NH, 1H, J_{HH} = 8.7 Hz), 7.07 (d, ArH, 1H, J_{HH} = 7.9 Hz), 6.77 (d, ArH, 1H, J_{HH} = 8.0 Hz), 6.60 (d, ArH, 1H, J_{HH} = 8.0 Hz), 4.67 (dd, CHNH, 1H,

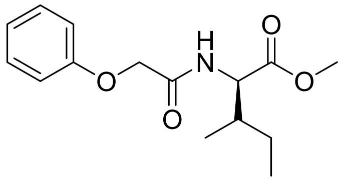

J_{HH} = 8.9 Hz, J_{HH} = 4.8 Hz), 4.52 (OCH, 1H, J_{HH} = 14.8 Hz), 4.48 (d, OCH, 1H, J_{HH} = 14.8 Hz), 3.75 (s, OCH_3, 3H), 2.74 (m, CH_2, 4H), 1.95 (m, CH, 1H), 1.80 (m, CH_2, 4H), 1.44 (m, CH, 1H), 1.15 (m, CH, 1H, 0.93 (m, CH_3, 6H) ppm. ^{13}C NMR ($CDCl_3$) 171.9, 168.1, 157.2, 129.8, 122.2, 114.8, 67.3, 56.0, 52.2, 37.9, 18.8, 15.4, 11.5 ppm. MS (CI) 334.2 ($[M+H]^+$). Accurate mass calculated for $C_{19}H_{27}NO_4$: 334.2013. Found: 334.2016.

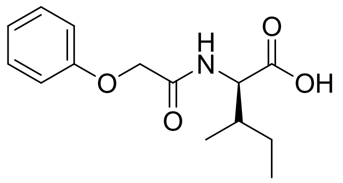

PhOIOH (34): ^{1}H NMR (DMSO) 8.06 (d, NH, 1H, J_{HH} = 8.5 Hz), 7.29 (m, ArH, 2H), 6.96 (m, ArH, 3H), 4.61 (d, OCH, 1H, J_{HH} = 14.6 Hz), 4.56 (d, OCH, 1H, J_{HH} = 14.6 Hz), 4.25 (dd, CHNH, 1H, J_{HH} = 8.5 Hz, J_{HH} = 5.9 Hz), 1.84 (m, CH, 1H), 1.40 (m, CH, 1H), 1.12 (m, CH, 1H), 0.85 (m, CH_3, 6H) ppm. ^{13}C NMR (DMSO) 172.7, 167.7, 157.7, 129.4, 121.1, 114.5, 66.4, 55.9, 36.3, 15.5, 11.2 ppm. MS (ES) 288 ($[M+Na]^+$). Accurate mass calculated for $C_{14}H_{19}NO_4Na$: 288.1212. Found: 288.1215.

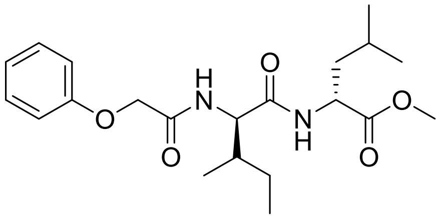

PhOILOMe: ^{1}H NMR ($CDCl_3$) 7.31 (m, ArH, 2H), 7.09 (d, NH, 1H, J_{HH} = 8.5 Hz), 7.02 (m, ArH, 1H), 6.93 (m, ArH, 2H), 6.11 (d, NH, 1H, J_{HH} = 8.0 Hz), 4.60 (m, CHNH, 1H), 4.55 (d, OCH, 1H, J_{HH} = 15.0 Hz), 4.50 (d, OCH, 1H, J_{HH} = 15.0 Hz), 4.34 (dd, CHNH, 1H, J_{HH} = 8.9 Hz, J_{HH} = 7.1 Hz), 3.74 (s, OCH_3, 3H), 1.90 (m, CH, 1H), 1.64 (m, CH_2, 2H), 1.11 (m, CH, 1H), 0.91 (m, CH_3, 12H) ppm. ^{13}C NMR ($CDCl_3$) 172.9, 170.3, 168.3, 129.8, 129.7, 122.2, 114.7, 67.2, 57.2, 52.3, 50.8, 41.9, 41.4, 37.2, 24.9, 22.7, 21.9, 19.0, 15.3, 11.2 ppm. MS 415 ($[M+Na]^+$). Accurate mass calculated for $C_{21}H_{32}N_2O_5Na$: 415.2209. Found: 415.2200.

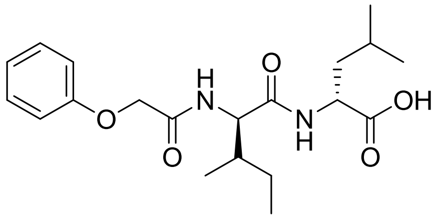

PhOILOH (35): ^{1}H NMR (DMSO) 8.27 (d, NH, 1H, J_{HH} = 7.8 Hz), 7.84 (d, NH, 1H, J_{HH} = 9.1 Hz), 7.29 (m, ArH, 2H), 6.94 (m, ArH, 2H), 4.58 (d, OCH, 1H, J_{HH} = 14.7 Hz), 4.54 (d, OCH, 1H, J_{HH} = 14.7 Hz), 4.31 (dd, CHNH, 1H, J_{HH} = 8.9 Hz, J_{HH} = 7.3 Hz), 4.20 (m, CHNH, 1H), 1.76 (m, CH, 1H), 1.60 (m, CH, 1H), 1.52 (m, CH, 2H), 1.41 (m, CH, 1H), 1.03 (m, CH, 1H), 0.84 (m, CH_3, 12H) ppm. ^{13}C NMR (DMSO) 173.8, 170.7, 167.2, 157.6, 129.4, 121.1, 114.6, 66.5, 56.0, 50.2, 37.1, 24.2, 24.0, 22.8, 21.2, 15.2, 10.9 ppm. MS (ES) 401 ($[M+Na]^+$). Accurate mass calculated for $C_{20}H_{30}N_2O_5Na$: 401.2052. Found: 401.2053.

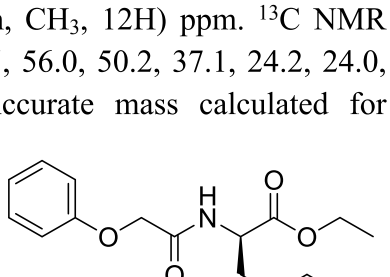

PhOFOEt: ^{1}H NMR ($CDCl_3$) 7.24 (m, ArH, 5H), 7.00 (m, ArH, 4H), 6.86 (m, ArH and NH, 2H), 4.93 (m, CHNH, 1H), 4.49 (d, OCH, 1H, J_{HH} = 15.0 Hz), 4.446 (d, OCH, 1H, J_{HH} = 15.0 Hz), 4.16 (q, CH_2, 2H, J_{HH} = 7.1 Hz), 3.13 (d, CH_2Ph, 2H, J_{HH} = 6.0 Hz), 1.22 (t, CH_3, 3H, J_{HH} = 7.1 Hz) ppm. ^{13}C NMR ($CDCl_3$) 170.9, 169.1,

167.9, 157.2, 135.6, 130.1, 129.7, 129.5, 129.3, 128.7, 128.3, 127.2, 122.1, 114.7, 67.2, 61.6, 52.6, 38.0, 14.1 ppm. MS (ES) 350 ($[M+Na]^+$). Accurate mass calculated for $C_{19}H_{21}NO_4Na$: 350.1368. Found: 350.1372.

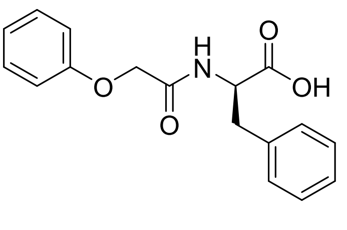

PhOFOH (36): ^{1}H NMR (DMSO) 8.25 (d, NH, 1H, J_{HH} = 8.2 Hz), 7.21 (m, ArH, 7H), 6.95 (t, ArH, 1H, J_{HH} = 7.3 Hz), 6.86 (m, ArH, 2H), 4.54 (m, CHNH and OCH, 2H), 4.44 (d, OCH, 1H, J_{HH} = 15.1 Hz), 3.13 (dd, CHPh, 1H, J_{HH} = 13.8 Hz, J_{HH} = 4.7 Hz), 2.99 (dd, CHPh, 1H, J_{HH} = 13.8 Hz, J_{HH} = 9.4 Hz) ppm. ^{13}C NMR (DMSO) 172.6, 167.6, 157.6, 137.4, 129.4, 129.1, 128.2, 126.4, 121.1, 114.6, 66.5, 52.9, 36.4 ppm. MS (ES) 322 ($[M+Na]^+$). Accurate mass calculated for $C_{17}H_{17}NO_4Na$: 322.1055. Found: 322.1058.

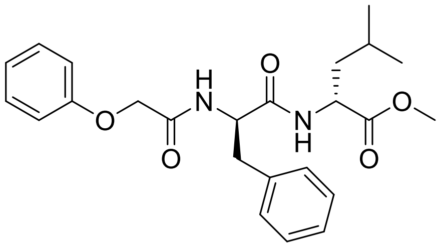

PhOFLOMe: ^{1}H NMR ($CDCl_3$) 7.26 (m, ArH, 4H), 7.20 (m, ArH, 2H), 7.12 (d, NH, 1H, J_{HH} = 7.8 Hz), 7.02 (m, ArH, 1H), 6.86 (m, ArH, 2H), 6.08 (d, NH, 1H, J_{HH} = 7.9 Hz), 4.73 (dd, CHNH, 1H, J_{HH} = 7.4 Hz, J_{HH} = 7.2 Hz), 4.53 (m, CHNH,1H), 4.50 (d, OCH, 1H, J_{HH} = 15.0 Hz), 4.45 (d, OCH, 1H, J_{HH} = 15.0 Hz), 3.71 (s, OCH_3), 3H), 3.16 (dd, CHPh, 1H, J_{HH} = 13.8 Hz, J_{HH} = 6.4 Hz), 3.06 (dd, CHPh, 1H, J_{HH} = 13.8 Hz, J_{HH} = 8.2 Hz), 1.49 (m, CH and CH_2, 3H), 0.87 (m, CH_3, 6H) ppm. ^{13}C NMR ($CDCl_3$) 172.7, 169.9, 168.4, 157.1, 136.1, 129.8, 129.4, 128.7, 127.2, 122.2, 114.6, 37.2, 53.8, 52.3, 50.9, 41.4, 37.9, 24.8, 22.7, 21.9 ppm. MS (ES) 449 ($[M+Na]^+$). Accurate mass calculated for $C_{24}H_{30}N_2O_5Na$: 449.2052. Found: 449.2050.

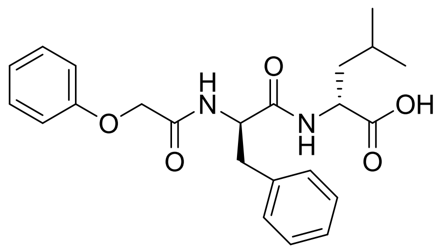

PhOFLOH (37): ^{1}H NMR (DMSO) 8.36 (d, NH, 1H, J_{HH} = 7.9 Hz), 8.07 (d, NH, 1H, J_{HH} = 8.6 Hz), 7.23 (m, ArH, 7H), 6.95 (t, ArH, 1H, J_{HH} = 7.3 Hz), 6.81 (dd, ArH, 2H, J_{HH} = 8.8 Hz, J_{HH} = 1.0 Hz), 4.66 (dt, CHNH, 1H, J_{HH} = 9.3 Hz, J_{HH} = 4.0 Hz), 4.43 (s, OCH_2, 2H), 4.26 (m, CHNH, 1H), 3.06 (dd, CHPh, 1H, J_{HH} = 13.8 Hz, J_{HH} = 4.1 Hz), 2.86 (dd, CHPh, 1H, J_{HH} = 13.8 Hz, J_{HH} = 9.6 Hz), 1.53 (m, CH and CH_2, 3H), 0.90 (d, CH_3, 3H, J_{HH} = 6.5 Hz), 0.85 (d, CH_3, 3H, J_{HH} = 6.5 Hz) ppm. ^{13}C NMR (DMSO) 173.9, 170.8, 167.3, 157.7, 137.5, 129.4, 129.3, 127.9, 126.3, 121.1, 114.6, 66.5, 53.1, 50.3, 37.5, 24.2, 22.8, 21.3 ppm. MS (ES) 411 ($[M-H]^-$). Accurate mass calculated for $C_{23}H_{27}N_2O_5$: 411.1920. Found: 411.1931.

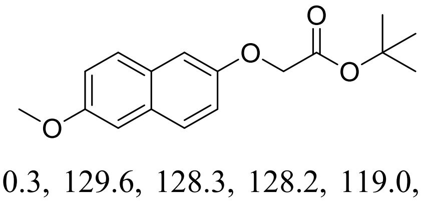

6-MeONapO*t*Bu: ^{1}H NMR ($CDCl_3$) 7.64 (d, ArH, 1H, $^3J_{HH}$ = 8.9 Hz), 7.59 (d, ArH, 1H, $^3J_{HH}$ = 8.8 Hz), 7.24 (s, ArH, 1H), 7.18 (dd, ArH, 1H, $^3J_{HH}$ = 9.1 Hz, $^3J_{HH}$ = 2.6 Hz), 7.09 (m, ArH, 2H), 4.58 (s, OCH_2, 2H), 3.88 (s, OCH_3, 3H), 1.49 (s, $C(CH_3)_3$, 9H) ppm. ^{13}C NMR ($CDCl_3$) 168.1, 156.5, 130.3, 129.6, 128.3, 128.2, 119.0, 118.9, 118.1, 107.9, 106.3, 82.2, 66.2, 55.3, 28.1 ppm. MS (CI) 306 ($[M+NH_4]^+$). Analysis calculated for C17H20O4: C, 70.81 %; H, 6.99 %. Found C, 70.81 %; H, 6.97 %.

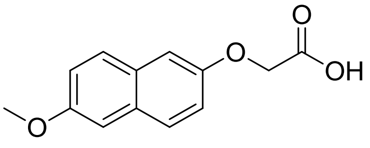

6-MeONapOH: ^{1}H NMR (DMSO) 7.77 (d, ArH, 1H, $^3J_{HH}$ = 8.9 Hz), 7.71 (d, ArH, 1H, $^3J_{HH}$ = 9.1 Hz), 7.27 (d, ArH, 1H, $^3J_{HH}$ = 2.6 Hz), 7.23 (d, ArH, 1H, $^3J_{HH}$ = 2.4 Hz), 7.13 (dd, ArH, 1H, $^3J_{HH}$ = 9.0Hz, $^3J_{HH}$ = 2.4 Hz), 4.76 (s, OCH_2, 2H), 3.84 (s, OCH_3, 3H) ppm. ^{13}C NMR (DMSO) 170.2, 155.8, 154.0, 129.6, 129.1, 128.1, 118.8, 118.6, 107.3, 106.1, 64.6, 55.1 ppm. MS (CI) 250 ($[M+NH_4]^+$). Analysis calculated for C13H12O4: C, 67.23 %; H, 5.21 %. Found C, 67.31 %; H, 5.24 %.

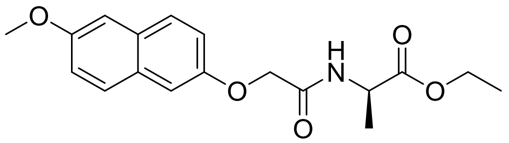

6-MeONapAOEt: ^{1}H NMR ($CDCl_3$) 7.68 (d, ArH, 1H, $^3J_{HH}$ = 9.1 Hz), 7.63 (d, ArH, 1H, $^3J_{HH}$ = 8.9 Hz), 7.13 (m, ArH and NH, 5H), 4.69 (t, C*H*NH, 1H, $^3J_{HH}$ = 7.4 Hz), 4.59 (s, OCH_2, 2H), 4.21 (q, C*H*$_2$CH$_3$, 1H, $^3J_{HH}$ = 7.4 Hz), 3.89 (s, OCH_3, 3H), 1.47 (d, CHC*H*$_3$, 3H, $^3J_{HH}$ = 7.4 Hz), 1.27 (t, CH$_2$C*H*$_3$, 3H, $^3J_{HH}$ = 7.4 Hz) ppm. ^{13}C NMR ($CDCl_3$) 172.5, 167.9, 156.6, 153.6m 130.4, 129.4, 128.6, 128.4, 119.4, 118.5, 108.1, 106.0, 67.5, 61.6, 55.3, 47.8, 18.5, 14.1 ppm. MS (ES) 354 ($[M+Na]^+$). Accurate mass calculated for $C_{18}H_{21}NO_5Na$: 354.1317. Found: 354.1313.

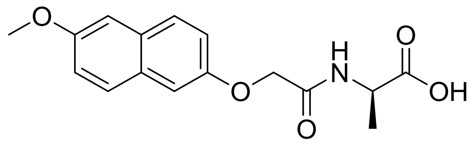

6-MeONapAOH (38): ^{1}H NMR (DMSO) 8.41 (d, NH, 1H, $^3J_{HH}$ = 7.5 Hz), 7.76 (d, ArH, 1H, $^3J_{HH}$ = 8.8 Hz), 7.69 (d, ArH, 1H, $^3J_{HH}$ = 9.0 Hz), 7.25 (m, ArH, 2H), 7.22 (dd, ArH, 1H, $^3J_{HH}$ = 8.9 Hz,$^3J_{HH}$ = 2.5 Hz), 7.13 (dd, ArH, 1H, $^3J_{HH}$ = 9.0 Hz, $^3J_{HH}$ = 2.5 Hz), 4.59 (d, OCH_2, 2H, $^3J_{HH}$ = 2.3 Hz), 4.35 (t, C*H*NH, 1H, $^3J_{HH}$ = 7.0 Hz), 3.84 (s, OCH_3, 3H), 1.34 (d, CHC*H*$_3$, 3H, $^3J_{HH}$ = 7.3 Hz) ppm. ^{13}C NMR (DMSO) 173.8, 167.6, 155.8, 153.9, 129.7, 129.1, 128.2, 128.1, 118.8, 107.7, 106.1, 66.8, 55.1, 47.3, 17.0 ppm. MS (ES) 326 ($[M+Na]^+$). Accurate mass calculated for $C_{16}H_{17}NO_5Na$: 326.1004. Found: 326.1005.

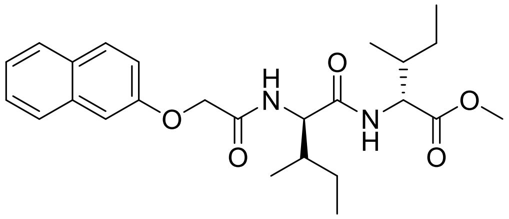

2NapIleIleOMe: ^{1}H NMR ($CDCl_3$) 7.78 (m, ArH, 2H), 7.72 (d, ArH, 1H, J_{HH} = 8.2 Hz), 7.45 (t, ArH, 1H, J_{HH} = 8.1 Hz), 7.35 (t, ArH, 1H, J_{HH} = 6.9 Hz), 7.20 (m, ArH and NH, 3H), 6.45 (d, NH, 1H, J_{HH} = 8.4 Hz), 4.67 (d, OCH, 1H, J_{HH} = *14.9* Hz), 4.62 (d, OCH, 1H, J_{HH} = 14.9 Hz), 4.55 (dd, CHNH, 1H, J_{HH} = 8.4 Hz, J_{HH} = 4.8 Hz), 4.43 (dd, CHNH, 1H, J_{HH} = 4.4 Hz), J_{HH} = 7.3 Hz), 3.74 (s, OCH_3, 3H), 1.87 (m, CH, 2H), 1.39 (m, CH_2, 2H), 1.11 (m, CH_2, 2H), 0.92 (m, CH_3, 12H) ppm. ^{13}C NMR ($CDCl_3$) 172.0, 170.5, 168.1, 154.9, 134.3, 129.9, 129.5, 127.7, 126.9, 126.7, 124.7, 118.2, 17.6, 67.3, 57.3, 56.5, 52.1, 37.7, 37.3, 25.2, 24.9, 15.4, 15.3, 11.6, 11.2 ppm. MS (ES) 441 ($[M-H]^-$). Accurate mass calculated for $C_{25}H_{33}N_2O_5$: 441.2389. Found: 441.2930.

2NapIIOH (39): ^{1}H NMR (DMSO) 8.16 (d, NH, 1H, J_{HH} = 8.0 Hz), 8.00 (d, NH, 1H, J_{HH} = 9.0 Hz), 7.84 (m, ArH, 2H), 7.74 (d, ArH, 1H, J_{HH} = 8.1 Hz), 7.47 (t, ArH, 1H, J_{HH} = 6.9 Hz), 7.34 (t, ArH, 1H, J_{HH} = 6.9 Hz), 7.24 (m, ArH, 2H), 4.72 (d, OCH, 1H, J_{HH} = 14.7 Hz),

4.67 (d, OCH, 1H, J_{HH} = 14.7 Hz), 4.42 (m, CHNH,1H), 4.16 (dd, CHNH, 1H, J_{HH} = 7.9 Hz, J_{HH} = 6.2 Hz), 1.76 (m, CH, 2H), 1.40 (m, CH, 2H), 1.11 (m, CH_2, 1H), 0.83 (m, CH_3, 12H) ppm. ^{13}C NMR (DMSO) 172.7, 170.9, 167.1, 155.5, 133.9, 129.4, 128.7, 127.56, 126.6, 126.4, 123.8, 118.5, 107.2, 66.7, 56.4, 56.0, 37.0, 36.1, 24.7, 24.1, 15.4, 15.2, 11.2, 10.9 ppm. MS (ES) 451 ([M+Na]$^+$). Accurate mass calculated for $C_{24}H_{32}N_2O_5Na$: 451.2209. Found: 451.2212.

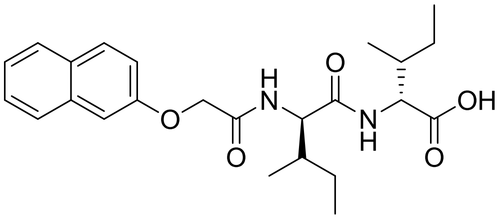

2NapGFOEt: ^{1}H NMR ($CDCl_3$) 7.78 (d, ArH, 2H, J_{HH} = 8.8 Hz), 7.73 (d, ArH, 1H, J_{HH} = 8.2 Hz), 7.46 (dt, ArH, 1H, J_{HH} = 6.9 Hz, J_{HH} = 1.2 Hz), 7.35 (dt, ArH, 1H, J_{HH} = 6.9 Hz, J_{HH} = 1.2 Hz), 7.22 (m, ArH and NH, 5H), 7.13 (d, ArH, 1H, J_{HH} = 2.5 Hz), 7.09 (m, ArH, 2H), 6.43 (d, NH, 1H, J_{HH} = 7.8 Hz), 4.84 (dt, CHNH, 1H, J_{HH} = 5.9 Hz, J_{HH} = 5.9 Hz), 4.62 (s, OCH_2, 2H), 4.16 (q, CH_2, 2H, J_{HH} = 7.1 Hz), 4.02 (d, CH_2NH, 2H, J_{HH} = 5.5 Hz), 3.13 (dd, CHPh, 1H, J_{HH} = 13.9 Hz), J_{HH} = 5.9 Hz), 3.07 (dd, CHPh, 1H, J_{HH} = 13.9 Hz, J_{HH} = 5.9 Hz), 1.24 (t, CH_3, 3H, J_{HH} = 7.1 Hz) ppm. ^{13}C NMR ($CDCl_3$) 171.1, 168.7, 167.8, 154.9, 135.6, 134.2, 129.9, 129.5, 129.3, 128.6, 127.7, 127.2, 127.0, 126.8, 124.4, 118.2, 107.5, 69.8, 67.2, 61.7, 53.2, 42.6, 37.8, 14.1 ppm. MS (ES) 457 [M+Na]$^+$. Accurate mass calculated for $C_{25}H_{26}N_2O_5Na$: 457.1739. Found: 1457.1734.

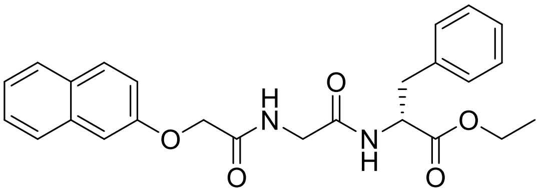

2NapGFOH (40): ^{1}H NMR (DMSO) 8.31 (t, NH, 1H, J_{HH} = 5.9 Hz), 8.25 (d, NH, 1H, J_{HH} = 8.0 Hz), 7.84 (m, ArH, 3H), 7.47 (dt, ArH, 1H, J_{HH} = 6.7 Hz, J_{HH} = 1.2 Hz), 7.37 (m, ArH, 2H), 7.23 (m, ArH, 6H), 4.64 (s, OCh_2, 2H), 4.46 (m, CHNH, 1H), 3.87 (dd, CHNH, 1H, J_{HH} = 16.7 Hz, J_{HH} = 6.0 Hz), 3.74 (dd, CHNH, 1H, J_{HH} = 16.7 Hz, J_{HH} = 5.7 Hz), 3.06 (dd, CHPh, 1H, J_{HH} = 13.8 Hz, J_{HH} = 5.1 Hz), 2.88 (dd, CHPh, 1H, J_{HH} = 13.8 Hz, J_{HH} = 8.9 Hz) ppm. ^{13}C NMR (DMSO) 172.8, 168.5, 167.8, 155.5, 137.4, 134.0, 129.3, 129.1, 128.7, 128.2, 127.5, 126.8, 126.4, 123.9, 118.5, 107.4, 66.9, 53.5, 41.4, 36.8 ppm. MS (ES) 405 [M-H]$^-$. Accurate mass calculated for $C_{23}H_{21}N_2O_5$: 405.1450. Found: 405.1450.

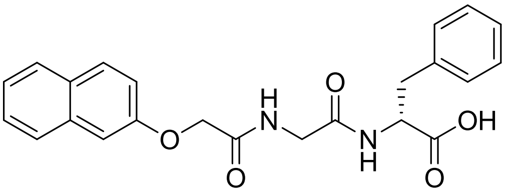

2NapLOMe: ^{1}H NMR ($CDCl_3$) 7.78 (m, ArH, 2H), 7.74 (d, ArH, 1H, J_{HH} = 8.2 Hz), 7.46 (t, ArH, 1H, J_{HH} = 6.9 Hz), 7.36 (t, ArH, 1H, J_{HH} = 6.9 Hz), 7.20 (dd, ArH, 1H, J_{HH} = 6.4 Hz, J_{HH} = 2.6 Hz), 7.16 (d, ArH, 1H, J_{HH} = 2.6 Hz), 6.94 (bd, NH, 1H, J_{HH} = 8.6 Hz), 4.76 (m, CHNH, 1H), 4.76 (s, OCH_2, 2H), 3.72 (s, OCH_3, 3H), 1.61 (m, CH and CH_2, 3H), 0.93 (d, CH_3, 3H, J_{HH} = 6.4 Hz), 0.88 (d, CH_3, 3H, J_{HH} = 6.3 Hz) ppm. ^{13}C NMR ($CDCl_3$) 172.9, 167.9, 155.0, 134.3, 129.9, 129.5, 127.7, 126.9, 126.7, 124.4, 118.2, 107.8, 67.4, 52.4, 50.3, 41.5, 24.8, 22.8, 21.8 ppm. MS (ES) 352 ([M+Na]$^+$). Accurate mass calculated for $C_{19}H_{23}NO_4Na$: 352.1525. Found: 352.1526.

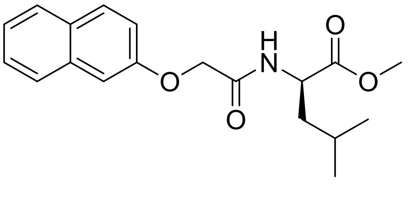

2NapLOH (41): ^{1}H NMR (DMSO) 8.40 (d, NH, 1H, J_{HH} = 8.2 Hz), 7.85 (dd, ArH, 1H, J_{HH} = 8.7 Hz, J_{HH} = 2.5 Hz), 7.75 (d, ArH, 2H, J_{HH} = 8.2 Hz), 7.47 (t, ArH, 1H, J_{HH} = 7.0 Hz), 7.37 (t, ArH, 1H, J_{HH} = 7.0 Hz), 7.25 (m, ArH, 2H), 4.70 (d, OCH, 1H, J_{HH} = 14.7 Hz), 4.65 (d, OCH, 1H, J_{HH} = 14.7 Hz), 4.34 (m, CHNH, 1H), 1.62 (m, CH and CH_2, 3H), 0.86 (d, CH_3, 3H, J_{HH} = 6.0 Hz), 0.82 (d, CH_3, 3H, J_{HH} = 6.0 Hz) ppm. ^{13}C NMR (DMSO) 173.8, 167.7, 155.6, 134.0, 129.2, 128.7, 127.5, 126.7, 126.4, 123.8, 118.7, 107.3, 66.7, 49.8, 24.3, 22.9, 21.1 ppm. MS (ES) 338 ($[M+Na]^+$). Accurate mass calculated for $C_{18}H_{21}NO_4Na$: 338.1368. Found: 338.1376.

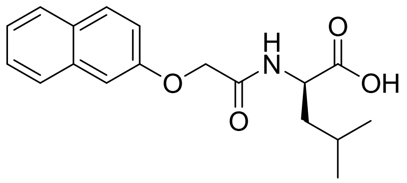

2NapLGOEt: ^{1}H NMR ($CDCl_3$) 7.79 (m, ArH, 2H), 7.73 (d, ArH, 1H, J_{HH} = 8.2 Hz), 7.46 (t, ArH, 1H, J_{HH} = 6.9 Hz), 7.36 (t, ArH, 1H, J_{HH} = 6.9 Hz), 7.20 (dd, ArH, 1H, J_{HH} = 8.9 Hz, J_{HH} = 2.6 Hz), 7.14 (d, ArH, 1H, J_{HH} = 2.6 Hz), 6.96 (bd, NH, 1H, J_{HH} = 8.4 Hz), 6.60 (m, NH, 1H), 4.66 (s, OCH_2, 2H), 4.61 (m, CHNH, 1H), 4.20 (q, CH_2CH_3, 2H, J_{HH} = 8.2 Hz), 3.98 (dd, CH_2NH, 2H, J_{HH} =5.4 Hz, J_{HH} = 1.6 Hz), 1.78 – 1.54 (m, CH and CH_2, 3H), 1.28 (t, CH_3, 3H, J_{HH} = 7.1 Hz), 0.91 (m, CH_3, 6H) ppm. ^{13}C NMR ($CDCl_3$) 171.6, 169.5, 168.5, 154.9, 134.2, 130.0, 129.5, 127.7, 126.9, 126.8, 124.4, 118.2, 107.6, 67.2, 61.6, 51.1, 41.3, 40.7, 24.6, 22.8, 21.9, 14.1 ppm. MS (ES) 423 ($[M+Na]^+$). Accurate mass calculated for $C_{22}H_{28}N_2O_5Na$: 423.1896. Found: 423.1884.

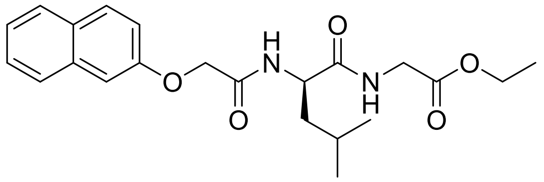

2NapLGOH (42): ^{1}H NMR (DMSO) 8.35 (t, NH, 1H, J_{HH} = 5.8 Hz), 8.21 (d, NH, 1H, J_{HH} = 8.6 Hz), 7.84 (m, ArH, 1H), 7.75 (d, ArH, 1H, J_{HH} = 8.1 Hz), 7.45 (t, ArH, 1H, J_{HH} = 6.9 Hz), 7.27 (m, ArH, 2H), 4.68 (d, OCH, 1H, J_{HH} = 14.6 Hz), 4.65 (d, OCH, 1H, J_{HH} = 14.6 Hz), 4.46 (m, CHNH, 1H), 3.73 (m, CH_2NH, 2H), 1.53 (m, CH and CH_2, 2H), 0.84 (d, CH_3, 3H, J_{HH} = 6.2 Hz), 0.80 (d, CH_3, 3H, J_{HH} = 6.2 Hz) ppm. ^{13}C NMR (DMSO) 172.1, 171.0, 167.3, 155.6, 133.9, 129.3, 128.7, 127.5, 126.6, 126.4, 123.7, 118.6, 107.2, 66.7, 50.4, 241, 23.0, 21.3 ppm. MS (ES) 395 ($[M+Na]^+$). Accurate mass calculated for $C_{20}H_{24}N_2O_5Na$: 395.1583. Found: 395.1574.

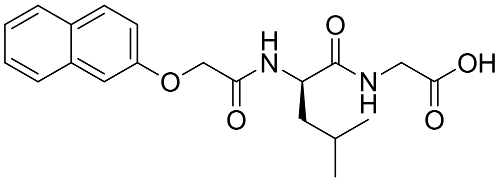

BrNapLOMe: ^{1}H NMR ($CDCl_3$) 7.94 (d, ArH, 1H, J_{HH} = 1.8 Hz), 7.70 (d, ArH, 1H, J_{HH} = 0.1 Hz), 7.61 (d, ArH, 1H, J_{HH} = 8.8 Hz), 7.53 (dd, ArH, 1H, J_{HH} = 8.7 Hz, J_{HH} = 1.8 Hz), 7.24 (dd, ArH, 1H, J_{HH} = 8.8 Hz, J_{HH} = 2.6 Hz), 7.12 (d, ArH, 1H, J_{HH} = 2.6 Hz), 6.92 (bd, NH, 1H, J_{HH} = 8.5 Hz), 4.75 (m, CHNH, 1H), 4.64 (s, OCH_2, 2H), 3.73 (s, OCH_3, 3H), 1.56 (m, CH and CH_2, 3H), 0.92 (d, CH_3, 3H, J_{HH} = 6.3 Hz), 0.89 (d, CH_3, 3H, J_{HH} = 6.3 Hz) ppm. ^{13}C NMR ($CDCl_3$) 172.8, 167.5, 151.5 132.9, 130.5, 129.5, 128.2, 126.3, 125.2, 114.6, 109.8, 68.8, 52.4, 50.5, 41.5, 24.9, 22.8, 21.9 ppm. MS (ES) 430 ($[M+Na]^+$). Accurate mass calculated for $C_{19}H_{22}NO_4Na^{79}Br$: 430.0630. Found: 430.0635.

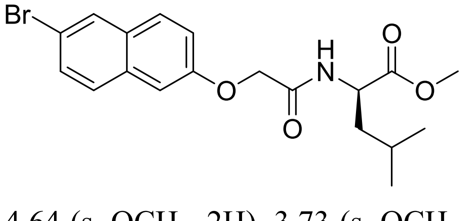

BrNapLOH (43): ^{1}H NMR (DMSO) 8.40 (d, NH, 1H, J_{HH} = 8.2 Hz), 7.13 (d, ArH, 1H, J_{HH} = 2.0 Hz), 7.85 (d, ArH, 1H, J_{HH} = 9.7 Hz), 7.72 (d, ArH, 1H, J_{HH} = 8.8 Hz), 7.58 (dd, ArH, 1H, J_{HH} = 8.8 Hz, J_{HH} = 2.0 Hz), 7.30 (m, ArH, 2H), 4.69 (d, OCH, 1H, J_{HH} = 14.7 Hz), 4.65 (d, OCH, 1H, J_{HH} = 14.7 Hz), 4.32 (m, CHNH, 1H), 1.56 (m, CH and CH_2, 3H), 0.86 (d, CH_3, 3H, J_{HH} = 6.1 Hz), 0.81 (d, CH_3, 3H, J_{HH} = 6.1 Hz) ppm. ^{13}C NMR (DMSO) 173.8, 167.5, 156.0, 132.6, 129.9, 129.4, 129.3, 128.9, 128.6, 119.9, 116.5, 107.4, 66.7, 49.9, 24.3, 22.9, 21.4 ppm. MS (ES) 392 ([M-H]$^-$). Accurate mass calculated for $C_{18}H_{19}NO_4{}^{79}Br$: 392.0497. Found: 392.0482.

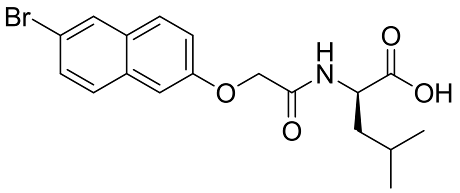

BrNapLGOEt: ^{1}H NMR ($CDCl_3$) 7.92 (d, ArH, 1H, J_{HH} = 1.8 Hz), 7.68 (d, ArH, 1H, J_{HH} = 9.1Hz), 7.59 (d, ArH, 1H, J_{HH} = 8.8 Hz), 7.51 (dd, ArH, 1H, J_{HH} = 8.8 Hz, J_{HH} = 1.8 Hz), 7.21 (dd, ArH, 1H, J_{HH} = 8.9 Hz, J_{HH} = 2.6 Hz), 7.10 (bd, NH, 1H, J_{HH} = 8.9 Hz), 6.74 (t, NH, 1H, J_{HH} = 5.2 Hz), 4.63 (m, CH and OCH_2, 3H), 4.20 (q, CH_2CH_3, 2H, J_{HH} = 7.1 Hz), 4.02 (dd, CHNH, 1H, J_{HH} = 18.2 Hz, J_{HH} = 5.4 Hz), 3.98 (dd, CHNH, 1H, J_{HH} = 18.2 Hz, J_{HH} = 5.4 Hz), 1.74 (m, CH, 1H), 1.58 (m, CH_2, 2H), 1.28 (t, CH_3, 3H, J_{HH} = 7.1 Hz), 0.92 (m, CH_3, 6H) ppm. ^{13}C NMR ($CDCl_3$) 171.9, 171.7, 168.7, 155.0, 132.7, 130.6, 130.1, 129.7, 129.1, 128.6, 119.2, 118.1, 107.7, 67.1, 51.1, 41.4, 41.2, 24.7, 22.8, 21.9 ppm. Accurate mass calculated for $C_{22}H_{27}N_2O_5{}^{79}BrNa$: 501.1001. Found: 501.1009.

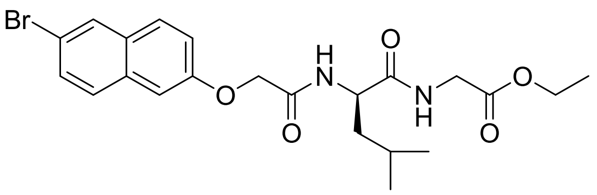

BrNapLGOH (44): ^{1}H NMR (DMSO) 8.35 (t, NH, 1H, J_{HH} = 5.9 Hz), 8.22 (d, NH, 1H, J_{HH} = 8.9 Hz), 8.13 (d, ArH, 1H, J_{HH} = 1.9 Hz), 7.85 (d, ArH, 1H, J_{HH} = 9.7 Hz), 7.73 (d, ArH, 1H, J_{HH} = 8.9 Hz), 7.58 (dd, ArH, 1H, J_{HH} = 8.7 Hz, J_{HH} = 1.9 Hz), 7.32 (m, ArH, 2H), 4.70 (d, OCH, 1H, J_{HH} = 14.6 Hz), 4.65 (d, OCH, 1H, J_{HH} = 14.6 Hz), 4.44 (m, CHNH, 1H), 3.77 (dd, CHNH, 1H, J_{HH} = 17.5 Hz, J_{HH} = 5.9 Hz), 3.72 (dd, CHNH, 1H, J_{HH} = 17.5 Hz, J_{HH} = 5.9 Hz), 1.54 (m, CH and CH_2, 3H), 0.84 (d, CH_3, 3H, J_{HH} = 6.2 Hz), 0.80 (d, CH_3, 3H, J_{HH} = 6.1 Hz) ppm. ^{13}C NMR (DMSO) 172.1, 171.0, 167.2, 156.0, 132.6, 129.9, 129.4, 129.3, 128.8, 128.6, 119.8, 116.5, 107.3, 66.7, 50.4, 40.8, 24.1, 23.0, 21.3 ppm. MS (ES) 449 ([M-H)]. Accurate mass calculated for $C_{20}H_{22}N_2O_5{}^{79}Br$: 449.0712. Found: 449.0709.

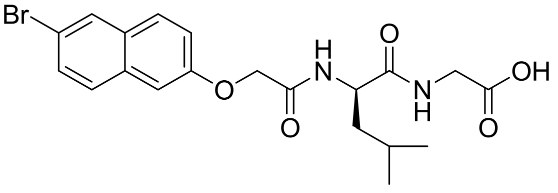

BrNapIFOEt: 7.94 (d, ArH, 1H, J_{HH} = 1.8 Hz), 7.70 (d, ArH, 1H, J_{HH} = 9.0 Hz), 7.60 (d, ArH, 1H, J_{HH} = 8.8 Hz), 7.53 (dd, ARH, 1H, J_{HH} = 8.8 Hz, J_{HH} = 1.8 Hz), 7.20 – 7.16 (m, ArH and NH, 5H), 7.09 (m, ArH, 4H), 6.32 (d, NH, 1H, J_{HH} = 7.9 Hz), 4.83 (dt, CHNH, 1H, J_{HH} = 7.9 Hz) J_{HH} = 6.1 Hz), 4.62 d, OCH, 1H, J_{HH} = 14.9 Hz), 4.55 (d, OCH,

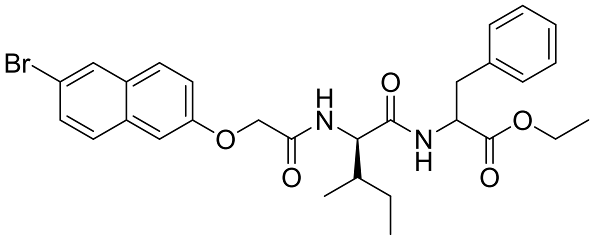

1H, J_{HH} = 14.9 Hz), 4.34 (dd, CHNH, 1H, J_{HH} = 8.8 Hz, J_{HH} = 6.9 Hz), 4.17 (q, CH_2, 2H, J_{HH} = 7.1 Hz), 3.11 (dd, CHPh, 1H, J_{HH} = 13.9 Hz, J_{HH} = 5.9 Hz), 3.04 (dd, CHPh, 1H, J_{HH} = 13.9 Hz, J_{HH} = 6.3 Hz), 1.84 (m, CH, 1H), 1.40 (m, CH, 1H), 1.24 (t, CH_3, 3H, J_{HH} = 7.1 Hz), 1.01 (m, CH, 1H), 0.87 (d, CH_3, 3H, J_{HH} = 6.8 Hz), 0.81 (t, CH_3, 3H, J_{HH} = 7.4 Hz) ppm. ^{13}C NMR ($CDCl_3$) 171.1, 170.1, 167.7, 155.2. 135.6. 132.7. 130.6. 130.1. 129.7, 129.3, 129.1, 128.6, 127.2, 119.3, 117.9, 107.6, 67.2, 61.6, 57.2, 53.1, 37.8, 37.1, 30.9, 24.8, 15.3, 14.1, 11.2 ppm. MS (ES) 591 ([M+Na]$^+$). Accurate mass calculated for $C_{29}H_{33}N_2O_5Na^{79}Br$: 591.1471. Found: 591.1472.

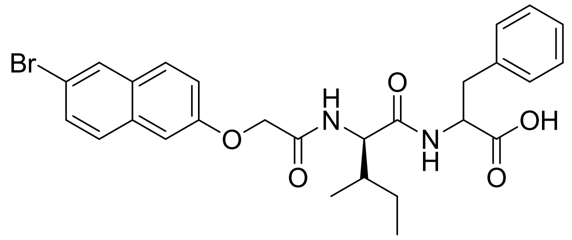

BrNapIFOH (45): ^{1}H NMR (DMSO) 8.38 (d, NH, 1H, J_{HH} = 7.8 Hz), 8.12 (d, ArH, 1H, J_{HH} = 1.8 Hz), 7.92 (d, ArH, 1H, J_{HH} = 9.2 Hz), 7.88 (d, ArH, 1H, J_{HH} = 9.8 Hz), 7.71 (d, ArH, 1H, J_{HH} = 8.8 Hz), 7.57 (dd, ArH, 1H, , J_{HH} = 8.8 Hz, J_{HH} = 1.8 Hz), 7.30 – 7.15 (m, ArH and NH, 7H), 4.69 (d, OCH, 1H, J_{HH} = 14.6 Hz), 4.64 (d, OCH, 1H, J_{HH} = 14.6 Hz), 4.44 (m, CHNH, 1H), 4.31 (dd, CHNH, 1H, J_{HH} = 8.9 Hz, J_{HH} = 7.4 Hz), 3.04 (dd, CHPh, 1H, J_{HH} = 13.9 Hz, J_{HH} = 5.3 Hz), 2.88 (dd, CHPh, 1H, J_{HH} = 13.9 Hz, J_{HH} = 9.2 Hz), 1.73 (m, CH, 1H), 1.36 (m, CH, 1H), 1.00 (m, CH, 1H), 0.78 (m, CH_3, 6H) ppm.172.7, 170.7, 166.9, 155.9, 137.5, 132.6, 129.8, 129.4, 129.3, 129.0, 128.8, 128.7, 128.1, 126.3, 119.7, 116.5, 107.4, 66.7, 56.1, 53.3, 38.8, 36.9, 23.9, 15.2, 10.8 ppm. MS (ES) 539 ([M-H]$^-$). Accurate mass calculated for $C_{27}H_{28}N_2O_5{}^{79}Br$: 539.1182. Found: 539.1187.

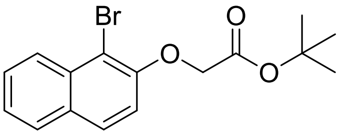

1-Br-2Nap-O^{t}Bu: ^{1}H NMR ($CDCl_3$) 8.23 (d, ArH, 1H, J_{HH} = 8.6 Hz), 7.77 (m, ArH, 2H), 7.55 (m, ArH, 1H), 7.39 (m, ArH, 1H), 7.14 (d, ArH, 1H), 4.72 (s, OCH_2, 2H), 1.48 (s, CH_3, 9H) ppm. ^{13}C NMR ($CDCl_3$) 167.7, 152.5, 133.2, 130.3, 128.8, 128.0, 127.8, 126.4, 124.8, 114.8, 109.9, 82.6, 67.4, 28.0 ppm. MS (ES) 359 ([M+Na]$^+$). Accurate mass calculated for $C_{16}H_{17}O_3Na^{79}Br$: 359.0259. Found: 359.0250.

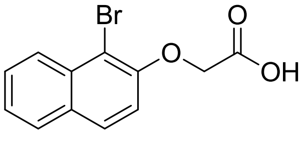

1-Br-2Nap-OH: ^{1}H NMR (DMSO) 8.11 (d, ArH, 1H, J_{HH} = 8.6 Hz), 7.96 (d, ArH, 1H, J_{HH} = 9.0 Hz), 7.96 (d, ArH, 1H, J_{HH} = 8.1 Hz), 7.76 (dt, ArH, 1H, J_{HH} = 9.8 Hz, J_{HH} = 1.2 Hz), 7.48 (dt, ArH, 1H, J_{HH} = 6.9 Hz, J_{HH} = 1.0 Hz), 7.40 (d, ArH, 1H, J_{HH} = 9.1 Hz), 4.98 (s, OCH_2, 2H) ppm. ^{13}C NMR (DMSO) 169.9, 152.4, 132.3, 129.85, 129.0, 128.3, 128.1, 125.2, 124.5, 114.7, 107.2, 65.5 ppm. MS (ES) 279 ([M-H]$^-$). Accurate mass calculated for $C_{12}H_8O_3{}^{79}Br$: 278.9657. Found: 278.9663.

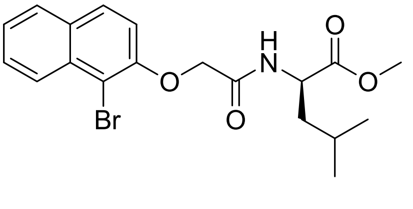

1-Br-2NapLOMe: ^{1}H NMR ($CDCl_3$) 8.22 (d, NH, 1H, J_{HH} = 8.6 Hz), 7.84 (d, ArH, 1H, J_{HH} = 8.9 Hz), 7.81 (d, ArH, 1H, J_{HH} = 8.2 Hz), 7.60 (m, ArH, 1H), 7.45 (m, ArH, 1H), 7.20 (d, ArH, 1H, J_{HH} = 8.9 Hz), 4.72 (m, OCH_2 and CHNH, 3H), 3.80 (s, OCH_3, 3H), 1.65 (m, CH and CH_2, 3H), 0.95 (m, CH_3, 6H) ppm. ^{13}C NMR ($CDCl_3$) 172.8, 167.5, 151.5, 132.9, 130.5, 129.5,

128.2, 128.1, 126.3, 125.1, 114.6, 109.8, 68.8, 52.4, 50.5, 41.5, 24.9, 22.8, 21.9 ppm. MS (ES) 430 ([M+Na]$^+$). Accurate mass calculated for $C_{19}H_{22}NO_4Na^{79}Br$: 430.0630. Found: 430.0624.

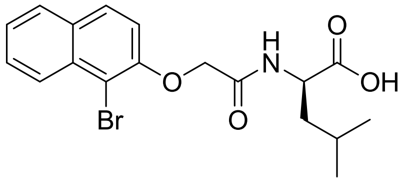

1-Br-2NapLOH: ^{1}H NMR (DMSO) 8.22 (d, NH, 1H, J_{HH} = 8.1 Hz), 8.09 (d, ArH, 1H, J_{HH} = 8.5 Hz), 7.96 (m, ArH, 2H), 7.64 (m, ArH, 1H), 7.50 (m, ArH, 1H), 7.41 (d, ArH, 1H, J_{HH} = 9.1 Hz), 4.86 (d, OCH, 1H, J_{HH} = 14.9 Hz), 4.80 (d, OCH, 1H, J_{HH} = 14.9 Hz), 4.32 (m, CHNH, 1H), 1.60 (m, CH and CH_2, 3H), 0.89 (d, CH_3, 3H, J_{HH} = 6.2 Hz), 0.86 (d, CH_3, 3H, J_{HH} = 6.2 Hz) ppm. ^{13}C NMR (DMSO) 173.6, 167.3, 152.4, 132.2, 129.7, 129.1, 128.3, 128.2, 125.3, 124.7, 115.4, 107.6, 68.0, 50.0, 24.3, 22.8, 21.3 ppm. MS (ES) 416 ([M+Na]$^+$). Accurate mass calculated for $C_{18}H_{20}NO_4Na^{79}Br$: 416.0473. Found: 416.0471.

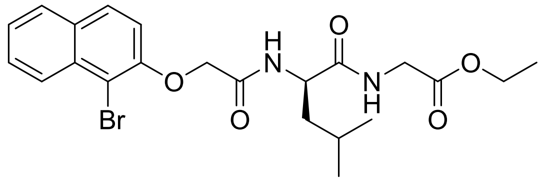

1-Br-2NapLGOEt: ^{1}H NMR ($CDCl_3$) 8.21 (d, NH, 1H, J_{HH} = 8.6 Hz), 7.83 (d, ArH, 1H, J_{HH} = 8.9 Hz), 7.80 (d, ArH, 1H, J_{HH} = 8.2 Hz), 7.60 (m, ArH, 1H), 7.45 (m, ArH, 1H), 7.19 (d, ArH, 1H), 6.72 (bt, NH, 1H, J_{HH} = 5.4 Hz), 4.73 (d, OCH, 1H, J_{HH} = 14.5 Hz), 4.69 (d, OCH, 1H, J_{HH} = 14.5 Hz), 4.61 (m, CHNH, 1H), 4.20 (q, CH_2, 2H, J_{HH} = 7.1 Hz), 4.04 (t, CH_2NH, 2H, J_{HH} = 5.4 Hz), 1.80 (m, CH, 1H), 1.68 (m, CH_2, 2H), 1.27 (t, CH_3, 3H, J_{HH} = 7.1 Hz), 0.97 (m, CH_3, 6H) ppm. ^{13}C NMR ($CDCl_3$) 171.6, 169.6, 168.1, 151.4, 132.9, 130.5, 129.5, 128.2, 126.3, 125.2, 114.7, 109.9, 68.9, 61.6, 51.4, 41.4, 40.7, 24.8, 22.9, 21.9, 19.0, 14.1 ppm. MS (ES) 501 ([M+Na]$^+$). Accurate mass calculated for $C_{22}H_{27}N_2O_5Ma^{79}Br$: 501.1001. Found: 501.0993.

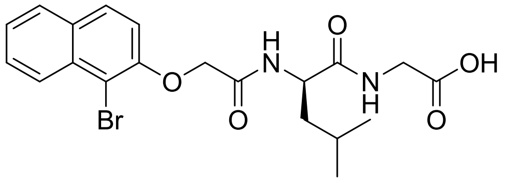

1-Br-2NapLGOH (46): ^{1}H NMR (DMSO) 8.44 (t, NH, 1H, J_{HH} = 5.6 Hz), 8.11 (d, ArH, 1H, J_{HH} = 8.5 Hz), 8.06 (d, NH, 1H, J_{HH} = 8.7 Hz), 7.97 (d, ArH, 1H, J_{HH} = 9.0 Hz), 7.95 (d, ArH, 1H, J_{HH} = 8.2 Hz), 7.66 (m, ArH, 1H), 7.46 (m, ArH, 1H), 7.41 (d, ArH, 1H, J_{HH} = 9.0 Hz), 4.84 (d, OCH, 1H, J_{HH} = 14.8 Hz), 4.80 (d, OCH, 1H, J_{HH} = 14.8 Hz), 4.46 (m, CHNH, 1H), 3.77 (t, CH_2NH, 2H), 4.54 (m, CH and CH_2), 0.87 (m, CH_3, 6H) ppm. ^{13}C NMR (DMSO) 171.9, 171.0, 166.9, 152.3, 132.2, 129.7, 129.2, 128.3, 128.2, 125.2, 124.6, 115.3, 107.6, 67.9, 50.4, 41.4, 25.1, 24.1, 23.0, 21.6 ppm. MS (ES) 473 ([M+Na]$^+$). Accurate mass calculated for $C_{20}H_{23}N_2O_5Ma^{79}Br$: 473.0688. Found: 473.0685.

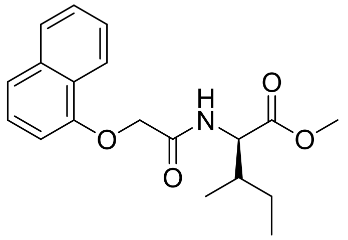

1NapIOMe: ^{1}H NMR ($CDCl_3$) 8.24 (m, ArH, 1H), 7.84 (m, ArH, 1H), 7.54 (m, ArH, 3H), 7.38 (t, ArH, 1H, J_{HH} = 8.1 Hz), 7.20 (bd, NH, 1H, J_{HH} = 8.6 Hz), 6.83 (d, ArH, 1H, J_{HH} = 7.6 Hz), 4.57 (d, OCH, 1H, J_{HH} = 14.9 Hz), 4.71 (m, OCH and CHNH, 2H), 3.75 (s, OCh_3, 3H), 1.95 (m, CH, 1H), 1.41 (m, CH_2, 1H), 1.11 (m, CH_2, 1H), 0.90 (m, CH_3, 6H) ppm. ^{13}C NMR ($CDCl_3$) 171.9, 168.0, 152.9, 134.6, 127.8, 126.7, 125.9, 125.7, 125.2, 121.9, 121.2, 105.8,

67.7, 56.1, 52.2, 37.9, 25.1, 15.5, 11.5 ppm. MS (CI) 330 ($[M+H]^+$). Accurate mass calculated for $C_{19}H_{23}NO_4$: 330.1700. Found: 330.1708.

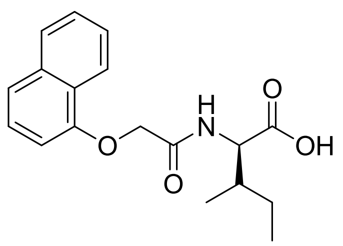

1NapIOH (47): ^{1}H NMR (DMSO) 8.22 (m, ArH and NH, 2H), 7.90 (m, ArH, 1H), 7.51 (m, ArH, 3H), 7.40 (t, ArH, 1H, J_{HH} = 7.8 Hz), 6.90 (d, ArH, 1H, J_{HH} = 7.1 Hz), 4.83 (d, OCH, 1H, J_{HH} = 14.5 Hz), 4.79 (d, OCH, 1H, J_{HH} = 14.5 Hz), 4.31 (dd, CHNH, 1H, J_{HH} = 8.5 Hz, J_{HH} = 5.8 Hz), 1.87 (m, CH, 1H), 1.43 (m, CH_2, 1H), 1.19 (m, CH_2, 1H), 0.87 (m, CH_3, 6H) ppm. ^{13}C NMR (DMSO) 172.7, 167.5, 153.3, 134.0, 127.5, 126.5, 125.9, 125.4, 124.8, 121.5, 120.5, 105.6, 66.9, 55.9, 36.4, 24.6, 15.5, 11.2 ppm. MS (ES) 314 ($[M-H]^-$). Accurate mass calculated for $C_{18}H_{20}NO_4$: 314.1392. Found: 314.1385.

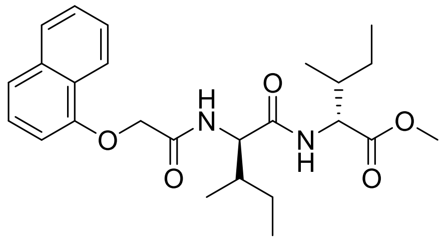

1NapIIOMe: ^{1}H NMR ($CDCl_3$) 8.23 (m, ArH, 1H), 7.82 (m, ArH, 1H), 7.51 (m, ArH, 3H), 7.34 (t, ArH, 1H, J_{HH} = 8.1 Hz), 7.26 (m, NH, 1H), 6.81 (d, ArH, 1H, J_{HH} = 8.1 Hz), 6.48 (bd, NH, 1H, J_{HH} = 8.4 Hz), 4.75 (d, OCH, 1H, J_{HH} = 15.0 Hz), 4.70 (d, OCH, 1H, J_{HH} = 15.0 Hz), 4.58 (dd, CHNH, 1H, J_{HH} = 8.4 Hz, J_{HH} = 4.8 Hz), 4.46 (dd, CHNH, 1H, J_{HH} = 8.7 Hz, J_{HH} = 6.9 Hz), 3.74 (s, OCH_3, 3H), 1.71 (m, CH, 2H), 1.43 (m, CH_2, 2H), 1.19 (m, CH_2, 2H), 0.91 (m, CH_3, 12H) ppm. ^{13}C NMR ($CDCl_3$) 172.2, 170.4, 168.3, 152.9, 134.7, 127.7, 126.8, 125.9, 125.6, 125.2, 121.9, 121.3, 105.7, 67.7, 57.3, 53.5, 52.1, 31.7, 27.4, 25.2, 24.9, 15.5, 15.3, 11.6, 11.2 ppm. MS (CI) 443 ($[M+H]^+$). Accurate mass calculated for $C_{25}H_{34}N_2O_5$: 443.2540. Found: 443.2552.

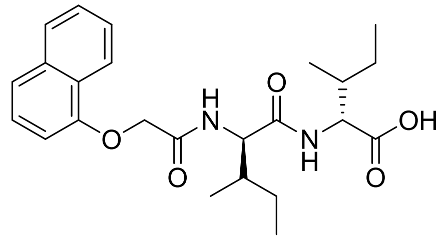

1NapIIOH (48): ^{1}H NMR (DMSO) 8.21 (m, ArH, 1H), 8.16 (d, ArH, 1H, J_{HH} = 8.0 Hz), 7.99 (d, ArH, 1H, J_{HH} = 9.0 Hz), 7.89 (m, ArH, 1H) 7.52 (m, ArH and NH, 3H), 7.36 (t, ArH, 1H, J_{HH} = 7.9 Hz), 6.89 (d, NH, 1H, J_{HH} = 7.6 Hz), 4.79 (d, OCH, 1H, J_{HH} = 14.5 Hz), 4.75 (d, OCH, 1H, J_{HH} = 14.5 Hz), 4.44 9dd, CHNH, 1H, J_{HH} = 8.9 Hz, J_{HH} = 7.3 Hz), 4.17 (dd, CHNH, 1H, J_{HH} = 7.9 Hz, J_{HH} = 6.1 Hz), 1.77 (m, CH, 2H), 1.45 (m, CH, 2H), 1.19 (m, CH, 1H), 1.00 (m, CH, 1H), 0.85 (m, CH_3, 12H) ppm. ^{13}C NMR (DMSO) 172.6, 170.9, 167.1, 153.4, 127.5, 126.5, 125.9, 125.4, 121.4, 120.6, 105.6, 67.1, 56.4, 55.9, 37.2, 36.1, 24.7, 24.1, 15.5, 15.2, 11.2, 10.9 ppm. MS (ES) 427 ($[M-H]^-$). Accurate mass calculated for $C_{24}H_{31}N_2O_5$: 427.2233. Found: 427.2227.

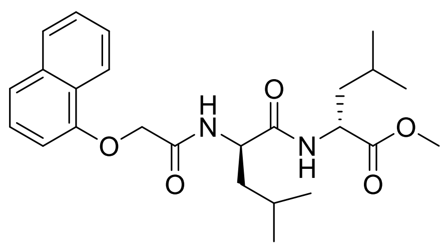

1NapLLOMe: ^{1}H NMR ($CDCl_3$) 8.22 (m, ArH, 1H), 7.82 (m, ArH, 1H), 7.52 (m, ArH, 3H), 7.35 (t, ArH, 1H, J_{HH} = 8.1 Hz), 7.03 (d, NH, 1H, J_{HH} = 8.4 Hz), 6.80 (d, ArH, 1H, J_{HH} = 7.6 Hz), 6.46 (bd, NH, 1H, J_{HH} = 8.1 Hz), 4.73 (d, OCH, 1H, J_{HH} = 15.1 Hz), 4.69 (d, OCH, 1H, J_{HH} = 15.1 Hz), 4.60 (m, CHNH, 2H), 3.74 (s, OCH_3, 3H), 1.61 (m, CH and CH_2, 6H), 0.91 (m, CH_3, 12H) ppm. ^{13}C NMR ($CDCl_3$) 173.1,

171.2, 168.4, 152.9, 134.6, 127.8, 126.8, 125.9, 125.6, 125.2, 122.0, 121.3, 105.7, 67.7, 52.3, 51.3, 50.8, 41.4, 41.0, 24.9, 24.7, 22.9, 22.8, 22.1, 21.9 ppm. MS (CI) 443 ([M+H]$^+$). Accurate mass calculated for $C_{25}H_{34}N_2O_5$: 443.2540. Found: 443.2557.

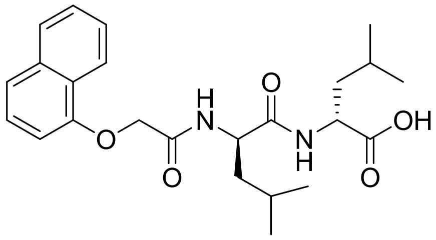

1NapLLOH (49): ^{1}H NMR (DMSO) 8.23 (m, ArH, 2H), 8.14 (d, ArH, 1H, J_{HH} = 8.6 Hz), 7.88 (m, ArH and NH, 3H), 7.38 (t, ArH, 1H, J_{HH} = 8.0 Hz), 6.88 (d, NH, 1H, J_{HH} = 7.6 Hz), 4.76 (d, OCH, 1H, J_{HH} = 14.7 Hz), 4.72 (d, OCH, 1H, J_{HH} = 14.7 Hz), 4.48 (m, CHNH, 1H), 4.23 (m, CHNH, 1H), 4.62 – 1.49 (m, CH and CH_2, 6H), 0.85 (m, CH_3, 12H) ppm. ^{13}C NMR (DMSO) 173.8, 171.7, 167.1, 153.2, 134.0, 127.4, 126.5, 125.9, 125.4, 124.7, 121.6, 120.6, 105.6, 67.1, 50.4, 50.1, 40.1, 24.2, 24.1, 23.1, 22.8, 22.8, 21.6, 21.2 ppm. MS (ES) 427 ([M-H]$^-$). Accurate mass calculated for $C_{24}H_{31}N_2O_5$: 427.2233. Found: 427.2247.

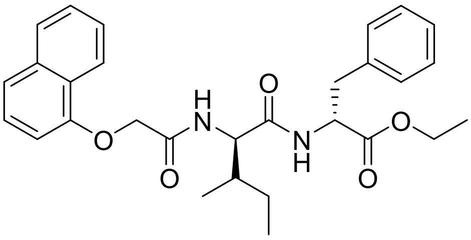

1NapIFOEt: ^{1}H NMR ($CDCl_3$) 823 (m, ArH, 1H), 7.84 (m, ArH, 1H), 753 (m, ArH, 3H), 7.35 (t, ArH, 1H, J_{HH} = 7.8 Hz), 7.25 – 7.13 (m, ArH and NH, 4H), 7.08 (m, ArH, 2H), 6.80 (d, ArH, 1H, J_{HH} = 7.6 Hz), 6.27 (d, NH, 1H, J_{HH} = 8.0 Hz), 4.86 (m, CHNH, 1H), 4.72 (d, OCH, 1H J_{HH} = 14.9 Hz), 4.66 (d, OCH, 1H, J_{HH} = 14.9 Hz), 4.37 (dd, CHNH, 1H, J_{HH} = 8.8 Hz, J_{HH} = 6.6 Hz), 4.18 (q, CH_2, 2H, J_{HH} = 7.2 Hz), 3.14 (dd, CHPh, 1H, J_{HH} = 13.9 Hz, J_{HH} = 5.6 Hz), 3.04 (dd, CHPh, 1H, J_{HH} = 13.9 Hz, J_{HH} = 6.4 Hz), 1.87 (m, CH, 1H), 1.35 (m, CH_2, 1H), 1.23 (t, CH_3, 3H, J_{HH} = 7.2 Hz), 0.91 (m, CH_2, 1H), 0.88 (d, CH_3, 3H, J_{HH} = 6.8 Hz), 0.81 (t, CH_3, 3H, J_{HH} = 7.3 Hz) ppm. ^{13}C NMR ($CDCl_3$) 171.1, 170.1, 168.2, 152.9, 135.6, 134.7, 129.3, 128.6, 127.8, 127.2, 126.8, 126.0, 125.6, 125.2, 121.9, 121.3, 105.6, 67.6, 61.6, 57.2, 53.0, 37.9, 37.2, 24.8, 15.3, 14.1, 11.2 ppm. MS (ES) 513 ([M+Na]$^+$). Accurate mass calculated for $C_{29}H_{34}N_2O_5Na$: 513.2365. Found: 513.2357.

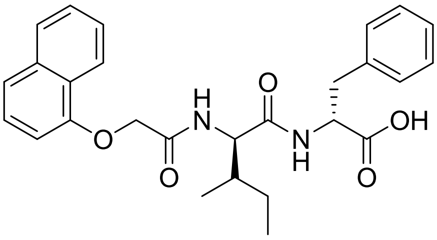

1NapIFOH (50): ^{1}H NMR (DMSO) 8.38 (d, NH, 1H, J_{HH} = 7.9 Hz), 8.20 (m, NH, 1H), 7.87 (m, ArH, 2H), 7.52 (m, ArH, 3H), 7.23 – 7.16 (m, ArH, 5H), 6.88 (d, ArH, 1H, J_{HH} = 7.7 Hz), 4.77 (d, OCH, 1H, J_{HH} = 14.5 Hz), 4.72 (d, OCH, 1H, J_{HH} = 14.5 Hz), 4.46 (m, CHNH, 1H), 4.33 (m, CHNH, 1H), 3.06 (dd, CHPh, 1H, J_{HH} = 14.0 Hz, J_{HH} = 5.2 Hz), 2.89 (dd, CHPh, 1H, J_{HH} = 14.0 Hz, J_{HH} = 9.3 Hz), 1.78 (m, CH, 1H), 1.36 (m, CH_2, 1H), 0.98 (m, CH_2, 1H), 0.80 (m, CH_3, 6H) ppm. ^{13}C NMR (DMSO) 172.7, 170.6, 167.0, 153.1, 137.5, 134.0, 129.0, 128.1, 127.5, 126.5, 126.3, 125.9, 125.4, 121.4, 120.6, 105.7, 67.0, 56.1, 52.5, 52.3, 36.6, 36.5, 23.9, 15.2, 10.9 ppm. MS (EI) 485 [M+Na]$^+$. Accurate mass calculated for $C_{27}H_{30}N_2O_5Na$: 485.2052. Found: 485.2050.

1NapGLOMe: ($CDCl_3$) 8.27 (m, ArH, 1H), 7.82 (m, ArH, 1H), 7.52 (m, ArH, 3H), 7.37 (m, ArH and NH,

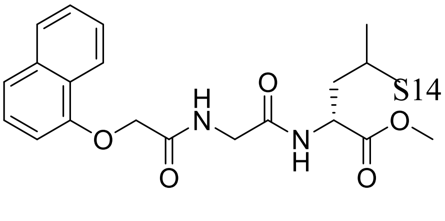

2H), 6.81 (d, ArH, 1H, J_{HH} = 7.5 Hz), 6.36 (d, NH, 1H, J_{HH} = 8.1 Hz), 4.75 (s, OCH_2, 2H), 4.64 (m, CHNH, 1H), 4.10 (d, CH_2NH, 2H, J_{HH} = 5.4 Hz), 3.74 (2, OCH_3, 3H), 1.62 (m, CH and CH_2, 3H), 0.93 (m, CH_3, 6H) ppm. ^{13}C NMR ($CDCl_3$) 173.1, 168.9, 168.1, 152.9, 134.6, 127.7, 126.8, 125.9, 125.6, 125.2, 121.9, 121.5, 105.5, 67.6, 52.4, 50.8, 42.7, 41.5, 24.9, 22.8, 21.9 ppm. MS (CI) 387 ($[M+H]^+$). Accurate mass calculated for $C_{21}H_{26}N_2O_5$: 387.1914. Found: 387.1925.

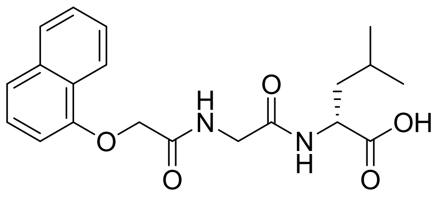

1NapGLOH (51): (DMSO) 8.38 (m, ArH, 1H), 8.31 (t, NH, 1H, J_{HH} = 5.7 Hz), 8.18 (d, NH, 1H, J_{HH} = 8.0 Hz), 7.89 (m, ArH, 1H), 7.53 (m, ArH, 3H), 7.41 (t, ArH, 1H, J_{HH} = 7.8 Hz), 6.96 (d, ArH, 1H, J_{HH} = 7.2 Hz), 4.73 (s, OCH_2, 2H), 4.27 (m, CHNH, 1H), 3.90 (dd, CHNH, 1H, J_{HH} = 16.7 Hz, J_{HH} = 5.8 Hz), 3.85 (dd, CHNH, 1H, J_{HH} = 16.7 Hz, J_{HH} = 5.8 Hz), 1.63 (m, CH, 1H), 1.53 (m, CH_2, 2H), 0.89 (d, CH_3, 3H, J_{HH} = 6.5 Hz), 0.85 (d, CH_3, 3H, J_{HH} = 6.5 Hz) ppm. ^{13}C NMR (DMSO) 173.9, 168.5, 167.7, 153.1, 134.0, 127.4, 126.5, 126.1, 125.3, 124.7, 121.9, 120.7, 105.7, 67.2, 50.2, 41.4, 24.2, 22.8, 21.3 ppm. MS (ES) 371 ($[M-H]^-$). Accurate mass calculated for $C_{20}H_{23}N_2O_5$: 371.1607. Found: 371.1606.

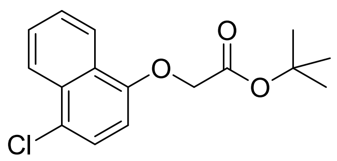

4-Cl-1-NapO^{t}Bu: ^1H NMR ($CDCl_3$) 8.38 (d, ArH, 1H, J_{HH} = 8.4 Hz), 8.19 (d, ArH, 1H, J_{HH} = 8.3 Hz), 7.61 (t, ArH, 1H, J_{HH} = 6.9 Hz), 7.55 (t, ArH, 1H, J_{HH} = 6.9 Hz), 7.41 (d, ArH, 1H, J_{HH} = 8.2 Hz), 6.60 (d, ArH, 1H, J_{HH} = 8.2 Hz), 4.67 (s, OCH_2, 2H), 1.52 (s, CH_3, 9H) ppm. ^{13}C NMR ($CDCl_3$) 167.6, 152.9, 131.4, 127.7, 126.2, 125.4, 124.1, 122.7, 104.9, 82.2, 66.1, 28.9 ppm. MS (ES) 315 ($[M+Na]^+$). Accurate mass calculated for $C_{16}H_{17}O_3Na^{35}Cl$: 315.0764. Found: 315.0755.

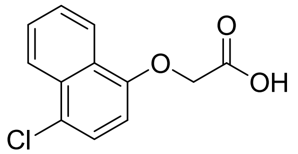

4-Cl-1-NapOH: ^1H NMR (DMSO) 8.31 (d, ArH, 1H, J_{HH} = 7.7 Hz), 8.12 (d, ArH, 1H, J_{HH} = 8.0 Hz), 7.72 (t, ArH, 1H, J_{HH} = 6.9 Hz), 7.65 (t, ArH, 1H, J_{HH} = 6.9 Hz), 7.58 (d, ArH, 1H, J_{HH} = 8.3 Hz), 6.91 (d, ArH, 1H, J_{HH} = 8.3 Hz), 4.92 (s, OCH_2, 2H) ppm. ^{13}C NMR (DMSO) 169.8, 152.5, 126.1, 125.9, 123.5, 122.3, 122.1, 105.8, 65.0 ppm. MS (CI) 254.1 ($[M+NH_4]^+$).

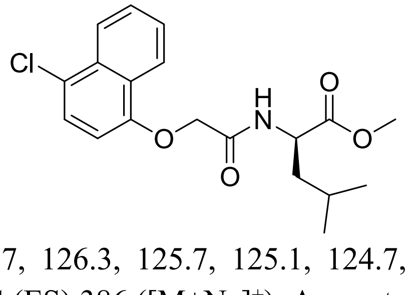

4-Cl-1-NapLOMe: ^1H NMR ($CDCl_3$) 8.26 (m, ArH, 2H), 7.61 (m, ArH, 2H), 7.46 (d, ArH, 1H, J_{HH} = 8.3 Hz), 6.96 (bd, NH, 1h, J_{HH} = 8.4 Hz), 6.75 (d, ArH, 1H, J_{HH} = 8.3 Hz), 4.75 (m, OCH and CHNH, 2H), 4.69 (d, OCH, 1H, J_{HH} = 15.0 Hz), 3.74 (s, OCH_3, 3H), 1.60 (m, CH and CH_2, 3H), 0.93 (d, CH_3, 3H, J_{HH} = 6.3 Hz), 0.90 (d, CH_3, 3H) ppm. ^{13}C NMR ($CDCl_3$) 172.9, 167.7, 152.0, 131.5, 127.9, 126.7, 126.3, 125.7, 125.1, 124.7, 121.7, 105.9, 67.9, 52.5, 50.4, 41.6, 24.9, 22.8, 21.9 ppm. MS (ES) 386 ($[M+Na]^+$). Accurate mass calculated for $C_{19}H_{22}NO_4Na^{35}Cl$: 386.1135. Found: 386.1126.

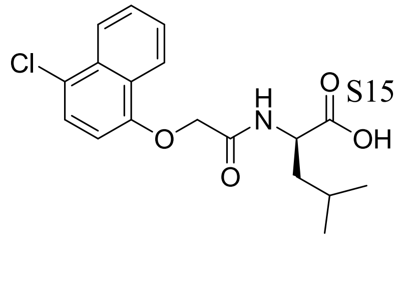

4-Cl-1-NapLOH (52): ^1H NMR (DMSO) 8.44 (d, NH, 1H, J_{HH} = 8.2 Hz), 8.38 (d, ArH, 1H, J_{HH} = 7.8 Hz), 8.13 (d,

ArH, 1H, J_{HH} = 8.0 Hz), 7.73 (m, ArH, 1H), 7.65 (m, ArH, 1H), 7.57 (d, ArH, 1H, J_{HH} = 8.3 Hz), 6.90 (d, ArH, 1H, J_{HH} = 8.3 Hz), 4.81 (d, OCH, 1H, J_{HH} = 14.5 Hz), 4.76 (d, OCH, 1H, J_{HH} = 14.5 Hz), 4.35 (m, CHNH, 1H), 1.61 (m, CH and CH_2, 3H), 0.89 (d, CH_3, 3H, J_{HH} = 6.1 Hz), 0.85 (d, CH_3, 3H, J_{HH} = 6.1 Hz) ppm. ^{13}C NMR (DMSO) 173.7, 167.3, 152.7, 13.4, 128.1, 126.3, 126.0, 125.9, 123.5, 122.7, 122.3, 106.1, 67.3, 49.9, 24.3, 22.8, 21.2, 17.9 ppm. MS (ES) 372 ([M+Na]$^+$). Accurate mass calculated for $C_{18}H_{20}NO_4Na^{35}Cl$: 372.0979. Found: 372.0973.

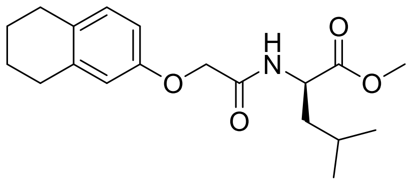

2-TetrahydroNapLOMe: ^{1}H NMR ($CDCl_3$) 6.99 (d, ArH, 1H, J_{HH} = 8.4 Hz), 6.88 (bd, NH, 1H, J_{HH} = 8.5 Hz), 6.70 (dd, ArH, 1H, J_{HH} = 8.4 Hz, J_{HH} = 2.8 Hz), 6.63 (d, ArH, 1H, J_{HH} = 2.8 Hz), 4.71 (m, CHNH, 1H), 4.48 (s, OCH_2, 2H), 3.74 (s, OCH_3, 3H), 2.72 (m, CH_2, 4H), 1.75 (m, CH_2, 4H), 1.59 (m, CH and CH_2, 3H), 0.93 (m, CH_3, 6H) ppm. ^{13}C NMR ($CDCl_3$) 172.9, 168.5, 155.1, 138.6, 130.9, 130.2, 114.8, 112.5, 67.5, 52.4, 50.2, 41.5, 29.7, 28.6, 24.8, 23.3, 23.0, 22.8, 21.8 ppm. MS (ES) 356 ([M+Na]$^+$). Accurate mass calculated for $C_{19}H_{27}NO_4Na$: 356.1838. Found: 356.1830.

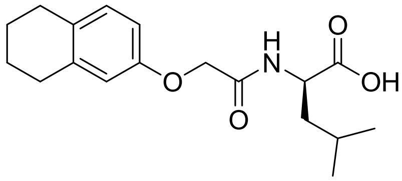

2-TetrahydroNapLOH (53): ^{1}H NMR (DMSO) 8.20 (d, NH, 1H, J_{HH} = 8.2 Hz), 6.94 (d, ArH, 1H, J_{HH} = 8.4 Hz), 6.68 (dd, ArH, 1H, J_{HH} = 8.4 Hz, J_{HH} = 2.7 Hz), 6.63 (d, ArH, 1H, J_{HH} = 2.7 Hz), 4.48 (d, OCH, 1H, J_{HH} = 14.8 Hz), 4.44 (d, OCH, 1H, J_{HH} = 14.8 Hz), 4.30 (m, CHNH, 1H), 2.65 (m, CH_2, 4H), 1.70 (m, CH_2, 4H), 1.57 (m, CH and CH_2, 3H), 0.88 (d, CH_3, 3H, J_{HH} = 6.1 Hz), 0.83 (d, CH_3, 3H, J_{HH} = 6.1 Hz) ppm. ^{13}C NMR (DMSO) 173.8, 167.9, 155.5, 137.5, 129.6, 129.1, 114.3, 112.6, 66.7, 49.8, 28.9, 27.9, 24.2, 22.9, 22.8, 22.6, 22.1 ppm. MS (ES) 342 ([M+Na]$^+$). Accurate mass calculated for $C_{18}H_{25}NO_4Na$: 342.1681. Found: 342.1674.

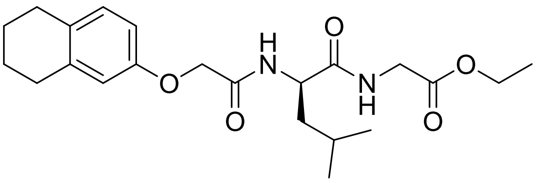

2-TetrahydroNapLGOEt: ^{1}H NMR ($CDCl_3$) 6.98 (d, ArH, 1H, J_{HH} = 8.5 Hz), 6.90 (bd, NH, 1H, J_{HH} = 8.4 Hz), 6.68 (dd, ArH, 1H, J_{HH} = 8.3 Hz, J_{HH} = 2.8 Hz), 6.62 (d, ArH, 1H, J_{HH} = 2.8 Hz), 4.57 (m, CHNH, 1H), 4.49 (s, OCH_2, 2H), 4.21 (q, CH_2, 2H), 4.00 (m, CH_2NH, 2H), 2.71 (m, CH_2, 4H)m 1.73 (m, CH_2, 4H), 1.69 (m, CH and CH_2, 3H), 1.27 (t, CH_3, 3H, J_{HH} = 7.1 Hz), 0.92 (m, CH_3, 6H) ppm. ^{13}C NMR ($CDCl_3$) 171.6, 169.5, 168.9, 154.9, 138.7, 130.9, 130.2, 114.7, 112.4, 67.4, 61.6, 51.1, 41.4, 40.6, 29.6, 28.6, 24.7, 23.3, 23.0, 22.9, 21.9, 14.1 ppm. MS (ES) 427 ([M+Na]$^+$). Accurate mass calculated for $C_{22}H_{32}N_2O_5Na$: 427.2209. Found: 427.2204.

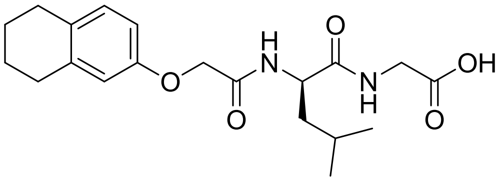

2-TetrahydroNapLGOH (54): ^{1}H NMR (DMSO) 8.30 (t, NH, 1H, J_{HH} = 5.8 Hz), 8.00 (d, NH, 1H, J_{HH} = 8.7 Hz), 6.95 (d, ArH, 1H, J_{HH} = 8.4 Hz), 6.67 (dd, ArH, 1H, J_{HH} = 8.4 Hz, J_{HH} = 2.7 Hz), 6.61 (d, ArH, 1H, J_{HH} = 2.7 Hz), 4.46 (m, OCH_2

and CHNH, 3H), 3.74 (m, CH_2NH, 2H), 2.63 (m, CH_2, 4H), 1.70 (m, CH_2, 4H), 1.50 (m, CH and CH_2, 3H), 0.86 (d, CH_3, 3H, J_{HH} = 6.1 Hz), 0.83 (d, CH_3, 3H, J_{HH} = 6.1 Hz) ppm. ^{13}C NMR (DMSO) 172.1, 171.0, 167.6, 155.5, 137.5, 129.7, 129.1, 114.2, 112.6, 66.7, 50.3, 29.0, 27.9, 24.0, 23.1, 22.9, 22.6, 21.4 ppm. MS (ES) 399 ($[M+Na]^+$). Accurate mass calculated for $C_{20}H_{28}N_2O_5Na$: 399.1896. Found: 399.1899.

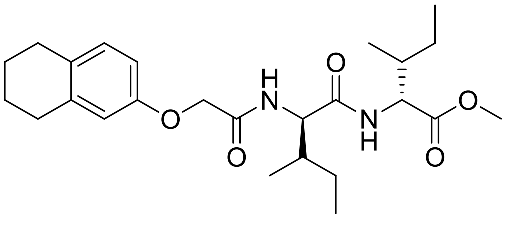

2-TetrahydroNapIIOMe: ^1H NMR ($CDCl_3$) 7.11 (d, ArH, NH, J_{HH} = 8.8 Hz), 6.98 (d, ArH, 1H, J_{HH} = 8.4 Hz), 6.68 (dd, ArH, 1H, J_{HH} = 8.3 Hz, J_{HH} = 2.7Hz), 6.63 (d, ArH, 1H, J_{HH} = 2.7 Hz), 6.41 (d, NH, 1H, J_{HH} = 8.4 Hz), 4.55 (dd, CHNH, 1H, J_{HH} = 8.4 Hz, J_{HH} = 4.8 Hz), 4.50 (d, OCH, 1H, J_{HH} = 15.1 Hz), 4.45 (d, OCH, 1H, J_{HH} = 15.1 Hz), 4.37 (dd, CHNH, 1H, J_{HH} = 8.8 Hz), J_{HH} = 7.3 Hz), 3.74 (s, OCH_3, 3H), 2.71 (m, CH_2, 4H), 1.89 (m, CH, 2H), 1.78 (m, CH_2, 4H), 1.48 (m, CH_2, 2H), 1.11 (m, CH_2, 2H), 0.90 (m, CH_3, 12H) ppm. ^{13}C NMR ($CDCl_3$) 171.9, 170.5, 168.6, 154.9, 138.6, 130.9, 130.2, 114.8, 112.4, 57.4, 57.3, 56.5, 52.1, 37.7, 37.1, 29.6, 28.6, 25.2, 24.9, 23.3, 23.0, 15.5, 15.3, 11.6, 11.2 ppm. MS (ES) 469 ($[M+Na]^+$). Accurate mass calculated for $C_{25}H_{38}N_2O_5Na$: 469.2678. Found: 469.2687.

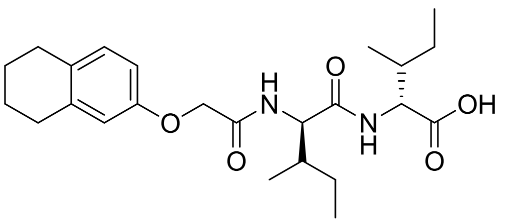

2-TetrahydroNapIIOH (55): ^1H NMR (DMSO) 8.14 (d, NH, 1H, J_{HH} = 8.0 Hz), 7.77 (d, NH, 1H, J_{HH} = 9.0 Hz), 6.94 (d, ArH, 1H, J_{HH} = 8.4 Hz), 6.66 (dd, ArH, 1H, J_{HH} = 8.3 Hz, J_{HH} = 2.7 Hz), 6.61 (d, ArH, 1H, J_{HH} = 2.7Hz), 4.51 (d, OCH, 1H, J_{HH} = 14.9 Hz), 4.46 (d, OCH, 1H, J_{HH} = 14.9 Hz), 4.38 (dd, CHNH, 1H, J_{HH} = 9.0 Hz, J_{HH} = 7.4 Hz), 4.14 (dd, CHNH, 1H, J_{HH} = 8.0 Hz, J_{HH} = 6.1 Hz), 2.65 (m, CH_2, 4H), 1.70 (m, CH_2 and CH, 6H), 1.39 (m, CH_2, 2H), 1.10 (m, CH, 2H), 0.84 (m, CH_3, 12H) ppm. ^{13}C NMR (DMSO) 172.7, 170.9, 167.4, 155.4, 137.5, 129.7, 129.2, 114.2, 112.5, 66.7, 56.4, 55.9, 37.1, 36.1, 29.0, 27.9, 24.7, 24.0, 22.9, 22.6, 15.4, 15.2, 11.2, 10.9 ppm. MS (ES) 455 ($[M+Na]^+$). Accurate mass calculated for $C_{24}H_{36}N_2O_5Na$: 455.2522. Found: 455.2510.

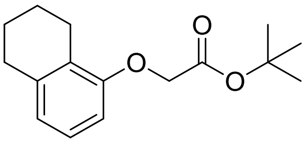

1-TetrahydroNapO^{t}Bu: ^1H NMR ($CDCl_3$) 7.01 (dd, 1H, ArH, J_{HH} = 7.8 Hz, J_{HH} = 7.8 Hz), 6.71 (d, ArH, 1H, J_{HH} = 7.6 Hz), 6.49 (d, ArH, 1H, J_{HH} = 8.1 Hz), 4.50 (s, OCH_2, 2H), 2.74 (m, CH_2, 4H), 1.76 (m, CH_2, 4H), 1.48 (s, CH_3, 9H) ppm. ^{13}C NMR ($CDCl_3$) 168.4, 155.8, 138.9, 126.5, 125.5, 122.3, 107.7, 82.1, 65.9, 41.9, 29.7, 28.1, 23.1, 22.8, 22.7, 22.7 ppm. MS (ES) 285 ($[M+Na]^+$). Accurate mass calculated for $C_{16}H_{22}O_3Na$: 285.1467. Found: 282.1463.

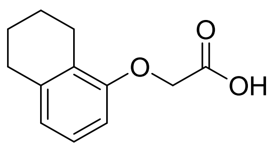

1-TetrahydroNapOH: ^1H NMR (DMSO) 7.00 (dd, ArH, 1H, J_{HH} = 7.9 Hz, J_{HH} = 7.9 Hz), 6.67 (d, ArH, 1H, J_{HH} = 7.6 Hz), 6.59 (d, ArH, 1H, J_{HH} = 7.9Hz), 4.55 (s, OCH_2, 2H), 2.69 (m, CH_2, 2H), 2.59 (m, CH_2, 2H), 1.69 (m, CH_2, 4H) ppm. ^{13}C NMR (DMSO) 170.3, 155.4,

137.9, 125.6, 125.1, 121.5, 107.9, 64.6, 28.9, 22.7, 22.3 ppm. MS (CI) 207 ([M+H]$^+$). Accurate mass calculated for $C_{12}H_{15}O_3$: 207.1016. Found: 207.1024.

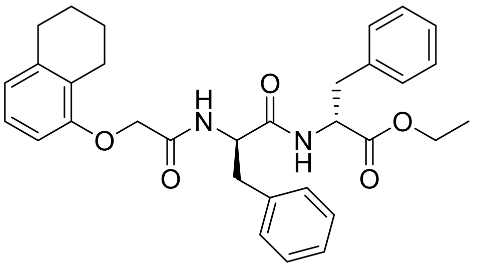

1-TetrahydroNapFFOEt: ^{1}H NMR ($CDCl_3$) 7.15 (m, ArH, 8H), 7.00 (m, ArH and NH, 4H), 6.50 (d, ArH, 1H, J_{HH} = 7.6 Hz), 6.50 (d, ArH, 1H, J_{HH} = 8.0 Hz), 6.30 (d, NH, 1H, J_{HH} = 7.7 Hz), 4.77 (m, CHNH, 2H), 4.41 (d, OCH, 1H, J_{HH} = 15.0 Hz), 4.37 (d, OCH, 1H, J_{HH} = 15.0 Hz), 4.14 (m, CH_2, 2H), 3.01 (m, CH_2Ph, 4H), 2.76 (m, CH_2, 2H), 2.54 (m, CH_2, 2H), 1.79 (m, CH_2, 4H), 1.22 (t, CH_3, 3H, J_{HH} = 7.2 Hz) ppm. ^{13}C NMR ($CDCl_3$) 170.9, 169.9, 168.7, 154.8, 139.2, 135.9, 135.6, 129.4, 129.2, 128.9, 128.7, 128.5, 127.1, 127.0, 125.9, 125.8, 122.9, 108.0, 66.9, 61.6, 53.5, 53.3, 37.9, 37.6, 29.6, 23.0, 22.7, 22.6, 14.1 ppm. MS (ES) 551 ([M+Na]$^+$). Accurate mass calculated for $C_{32}H_{36}N_2O_5Na$: 551.2522. Found: 551.2516.

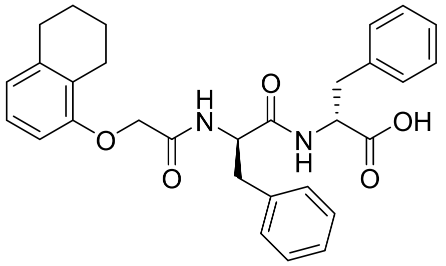

1-TetrahydroNapFFOH (56): ^{1}H NMR (DMSO) 8.46 (d, NH, 1H, J_{HH} = 7.9 Hz), 7.73 (d, NH, 1H, J_{HH} = 8.5 Hz), 7.21 (m, ArH, 10H), 6.95 (t, ArH, 1H, J_{HH} = 7.9 Hz), 6.67 (d, ArH, 1H, J_{HH} = 7.6 Hz), 6.48 (d, ArH, 1H, J_{HH} = 8.0 Hz), 4.65 (m, CHNH, 1H), 4.46 (m, CHNH, 1H), 4.41 (d, OCH, 1H, J_{HH} = 14.8 Hz), 4.33 (d, OCH, 1H, J_{HH} = 14.8 Hz), 3.10 (dd, CHPh, 1H, J_{HH} = 13.9 Hz, J_{HH} = 5.1 Hz), 3.02 (dd, CHPh, 1H, J_{HH} = 13.9 Hz, J_{HH} = 4.4 Hz), 2.93 (dd, CHPh, 1H, J_{HH} = 13.9 Hz, J_{HH} = 8.9 Hz), 2.86 (dd, CHPh, 1H, J_{HH} = 13.9 Hz, J_{HH} = 8.9 Hz), 2.50 (m, CH_2. 4H), 1.68 (m, CH_2, 4H) ppm. ^{13}C NMR (DMSO) 172.7, 170.5, 167.3, 155.1, 137.9, 137.4, 137.1, 129.3, 129.1, 128.2, 127.9, 126.4, 126.3, 125.7, 125.0, 121.8, 107.4, 66.7, 53.5, 52.7, 37.5, 36.7, 28.9, 22.5, 22.3 ppm. MS (ES) 523 ([M+Na]$^+$). Accurate mass calculated for $C_{30}H_{32}N_2O_5Na$: 523.2209. Found: 523.2200.

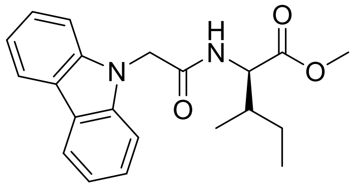

CarbIOMe: ^{1}H NMR ($CDCl_3$) 8.12 (d, ArH, 2H, J_{HH} = 7.7 Hz), 7.48 (m, ArH, 2H), 7.38 (d, ArH, 2H, J_{HH} = 8.2 Hz), 7.31 (m, ArH, 2H), 5.88 (d, NH, 1H, J_{HH} = 8.8 Hz), 4.98 (d, NCH, 1H, J_{HH} = 18.0 Hz), 4.93 (d, NCH, 1H, J_{HH} = 18.0 Hz), 4.51 (m, CHNH, 1H), 3.56 (s, OCH_3, 3H), 1.66 (m, CH, 1H), 1.48 (m, CH_2, 1H), 0.93 (m, CH_2, 1H), 0.67 (m, CH_3, 6H) ppm. ^{13}C NMR ($CDCl_3$) 171.4, 167.9, 140.3, 126.5, 123.5, 120.7, 120.4, 108.6, 56.3, 52.0, 47.1, 37.3, 24.7, 15.5, 11.2 ppm. MS (ES) 375 ([M+Na]$^+$). Accurate mass calculated for $C_{21}H_{24}N_2O_3Na$: 375.1685. Found: 375.1682.

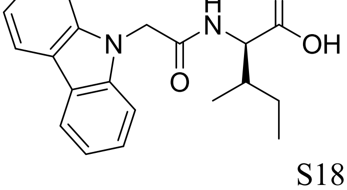

Carb-I-OH (57): ^{1}H NMR (DMSO) 8.59 (d, NH, 1H, J_{HH} = 8.6 Hz), 8.14 (d, ArH, 2H, J_{HH} = 7.7 Hz), 7.55 (d, ArH, 2H, J_{HH} = 8.2 Hz), 7.40 (dt, ArH, 2H, J_{HH} = 8.2 Hz, J_{HH} = 1.1 Hz), 7.16 (dt, ArH, 2H, J_{HH} = 7.7 Hz, J_{HH} = 0.9 Hz), 5.18 (d, NCH, 1H,

J_{HH} = 16.7 Hz), 5.11 (d, NCH, 1H, J_{HH} = 16.6 Hz), 4.22 (dd, CHNH, 1H, J_{HH} = 8.6 Hz, J_{HH} = 5.7 Hz), 1.84 (m, CH, 1H), 1.44 (m, CH_2, 1H), 1.23 (m, CH_2, 1H), 0.87 (m, CH_3, 6H) ppm. ^{13}C NMR (DMSO) 172.8, 167.6, 140.6, 125.6, 122.1, 120.1, 118.9, 109.3, 53.3, 45.1, 36.5, 24.7, 15.6, 11.3 ppm. MS (ES) 337 ([M-H]$^-$). Accurate mass calculated for $C_{20}H_{21}N_2O_3$: 337.1552. Found: 337.1543.

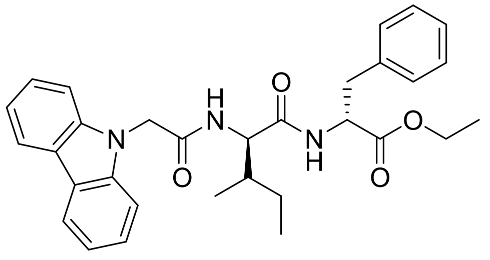

CarbIFOEt: ^{1}H NMR ($CDCl_3$) 8.13 (d, ArH, 2H, J_{HH} = 7.8 Hz), 7.48 (m, ArH, 3H), 7.33 (m, ArH, 5H), 7.20 (m, ArH, 3H), 6.96 (m, ArH, 2H), 6.06 (d, NH, 1H, J_{HH} = 8.1 Hz), 5.83 (d, NH, 1H, J_{HH} = 8.7 Hz), 4.97 (d, NCH, 1H, J_{HH} = 17.9 Hz), 4.91 (d, NCH, 1H, J_{HH} = 17.9 Hz), 4.70 (m, CHNH, 1H), 4.18 (m, CH_2, 2H), 3.04 (dd, CHPh, 1H, J_{HH} = 13.8 Hz, J_{HH} = 5.8 Hz), 2.83 (dd, CHPh, 1H, J_{HH} = 13.8 Hz, J_{HH} = 7.2 Hz), 1.61 (, CH_2, 2H), 1.22 (t, CH_3, 3H, J_{HH} = 7.2 Hz), 0.61 (d, CH_3, 3H, J_{HH} = 6.8 Hz), 0.54 (t, CH_3, 3H, J_{HH} = 7.2 Hz) ppm. ^{13}C NMR ($CDCl_3$) 171.1, 19.6, 168.2, 140.3, 135.7, 129.4, 129.2, 128.5, 127.1, 126.6, 123.5, 120.8, 120.5, 120.4, 108.4, 61.6, 57.5, 52.9, 47.0, 37.9, 36.2, 24.1, 18.9, 15.2, 14.1, 10.9 ppm. MS (ES) 536 ([M+Na]$^+$). Accurate mass calculated for $C_{31}H_{35}N_3O_4Na$: 536.2525. Found: 536.2515.

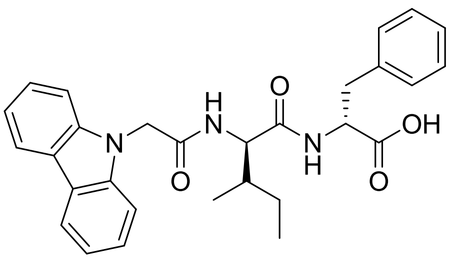

CarbIFOH (58): ^{1}H NMR (DMSO) 8.62 (d, NH, 1H, J_{HH} = 9.2 Hz), 8.13 (d, ArH, 2H, J_{HH} = 7.6 Hz), 7.55 (d, ArH, 2H, J_{HH} = 8.2 Hz), 7.49 (d, NH, 1H, J_{HH} = 5.8 Hz), 7.42 (m, ArH, 2H), 7.20 (m, ArH, 2H), 7.09 (m, ArH, 5H), 5.16 (d, NCH_2, 1H, J_{HH} = 16.7 Hz), 5.06 (d, NCH_2, 1H, J_{HH} = 16.7 Hz), 4.06 (m, CHNH, 1H), 3.95 (m, CHNH, 1H), 3.08 (dd, CHPh, 1H, J_{HH} = 13.2 Hz, J_{HH} = 5.3 Hz), 2.98 (dd, CHPh, 1H, J_{HH} = 13.2 Hz, J_{HH} = 5.2 Hz), 1.75 (m, CH, 1H), 1.38 (m, CH_2, 1H), 1.06 (m, CH_2, 1H), 0.79 (m, CH_3, 6H) ppm. This compound was too insoluble to record a ^{13}C NMR of sufficient quality. Attempts were made in DMSO and DMF. MS (ES) 508 ([M+Na]$^+$). Accurate mass calculated for $C_{29}H_{31}N_3O_4Na$: 508.2212. Found: 508.2203.

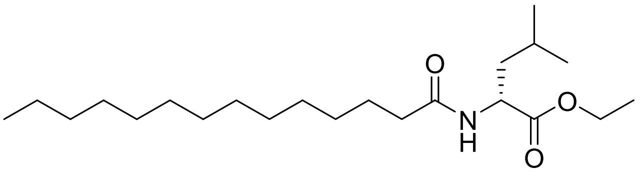

C_{14}LOEt: ^{1}H NMR ($CDCl_3$) 5.85 (d, NH, 1H, J_{HH} = 8.3 Hz), 4.64 (t, CHNH, 1H, J_{HH} = 8.7 Hz, J_{HH} = 4.9 Hz), 4.18 (q, CH_2, 2H, J_{HH} = 7.2 Hz), 2.21 (t, CH_2, 2H, J_{HH} = 7.4 Hz), 1.64 (m, CH_2, 4H), 1.52 (m, CH, 1H), 1.27 (m, CH_2, 20H), 0.95 (d, CH_3, 3H, J_{HH} = 6.2 Hz), 0.93 (d, CH_3, 3H, J_{HH} = 6.2 Hz), 0.88 (t, CH_3, 3H, J_{HH} = 7.2 Hz) ppm. ^{13}C NMR ($CDCl_3$) 173.3, 172.9, 61.3, 50.6, 41.9, 36.6, 31.9, 30.9, 29.7, 29.5, 29.5, 29.3, 29.2, 25.6, 24.9, 22.8, 22.7, 22.0, 14.1 ppm. MS (ES) 392 ([M+Na]$^+$). Accurate mass calculated for $C_{22}H_{43}NO_3Na$: 392.3141. Found: 392.3149.

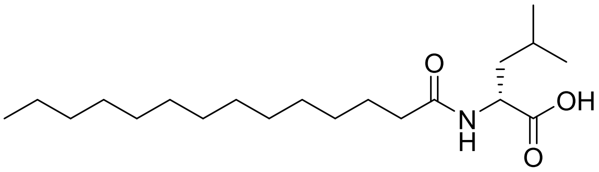

C_{14}LOH (59): ^{1}H NMR (DMSO) 7.99 (d, NH, 1H, J_{HH} = 8.0 Hz), 4.21 (m, CHNH, 1H), 2.09 (m, 2H, CH_2), 1.62 (m, CH, 1H), 1.48 (m, CH_2, 4H), 1.24 (m, CH_2, 20H), 0.85 (m, CH_3, 9H) ppm. ^{13}C NMR (DMSO) 174.3, 172.2, 49.9, 34.9, 31.3, 28.9, 28.9, 28.7, 28.7, 25.5, 25.2, 24.3, 22.8, 22.1, 21.1, 13.9 ppm. MS (ES) 364 ($[M+Na]^+$). Accurate mass calculated for $C_{20}H_{39}NO_3Na$: 364.2828. Found: 364.2811.

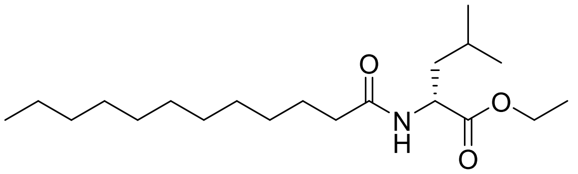

C_{12}LOEt: ^{1}H NMR ($CDCl_3$) 5.83 (d, NH, 1H, J_{HH} = 8.2 Hz), 4.64 (t, CHNH, 1H, J_{HH} = 8.7Hz, J_{HH} = 5.0 Hz), 4.18 (q, CH_2, 2H, J_{HH} = 7.1 Hz), 2.21 (t, CH_2, 2H, J_{HH} = 7.4 Hz), 1.64 (m, CH and CH_2, 5H), 1.27 (m, CH_2, 16H), 0.95 (d, CH_3, 3H, J_{HH} = 6.1 Hz), 0.94 (d, CH_3, 3H, J_{HH} = 6.1 Hz), 0.88 (t, CH_3, 3H, J_{HH} = 7.1 Hz) ppm. ^{13}C NMR ($CDCl_3$) 173.3, 172.9, 61.3, 50.6, 41.9, 36.6, 31.9, 30.9, 29.6, 29.5, 29.3, 29.2, 25.6, 24.9, 22.8, 22.7, 22.0, 14.1 ppm. MS (ES) 364 ($[M+Na]^+$). Accurate mass calculated for $C_{20}H_{39}NO_3Na$: 364.2828. Found: 364.2840.

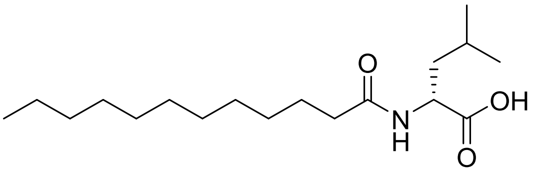

C_{12}LOH (60): ^{1}H NMR (DMSO) 7.99 (d, NH, 1H, J_{HH} = 8.0 Hz), 4.21 (m, CHNH, 1H), 2.09 (m, 2H, CH_2), 1.62 (m, CH, 1H), 1.48 (m, CH_2, 4H), 1.24 (m, CH_2, 16H), 0.88 (m, CH_3, 9H) ppm. ^{13}C NMR (DMSO) 174.3, 172.2, 49.9, 34.9, 31.3, 28.9, 28.9, 28.7, 28.7, 28.5, 25.2, 25.1, 24.3, 22.8, 22.1, 21.1, 13.9 ppm. MS (ES) 336 ($[M+Na]^+$). Accurate mass calculated for $C_{18}H_{35}NO_3Na$: 336.2515. Found: 336.2522.

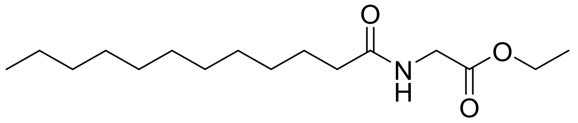

C_{14}GOEt: ^{1}H NMR ($CDCl_3$) 5.95 (s, NH, 1H), 4.22 (q, CH_2, 2H, J_{HH} = 7.2 Hz), 4.03 (d, CH_2NH, 2H, J_{HH} = 5.1 Hz), 2.50 (t, CH_2, 2H, J_{HH} = 7.8 Hz), 1.63 (m, CH_2, 2H), 1.27 (m, CH_2 and CH_3, 23H), 0.88 (t, CH_3, 3H, J_{HH} = 7.2Hz) ppm. ^{13}C NMR 173.3, 170.2, 61.5, 41.4, 36.5, 31.9, 29.7, 29.6, 29.6, 29.5, 29.4, 29.3, 29.2, 25.6, 22.7, 14.1 ppm. MS (ES) 336 ($[M+Na]^+$). Accurate mass calculated for $C_{18}H_{35}NO_3Na$: 336.2515. Found: 336.2509.

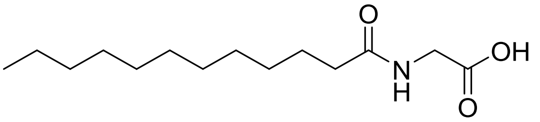

C_{14}GOH (61): ^{1}H NMR (DMSO) 8.04 (t, NH, 1H, J_{HH} = 5.9 Hz), 3.70 (d, CH_2NH, 2H, J_{HH} =5.9 Hz), 2.10 (t, CH_2, 2H, J_{HH} = 7.5 Hz), 1.48 (m, CH_2, 2H), 1.24 (m, CH_2, 20H), 0.86 (t, CH_3, 3H, J_{HH} = 7.0 Hz) ppm. ^{13}C NMR (DMSO) 172.4, 40.7, 35.0, 31.3, 29.0, 28.9, 28.9, 28.8, 28.7, 28.6, 25.2, 22.1, 13.9 ppm. MS (ES) 308 ($[M+Na]^+$). Accurate mass calculated for $C_{16}H_{31}NO_3Na$: 308.2202. Found: 308.2204.

BrNapAFOEt: ^{1}H NMR ($CDCl_3$) 7.97 (d, ArH, 1H, J_{HH} = 1.9 Hz), 7.73 (d, ArH, 1H, J_{HH} = 9.0 Hz), 7.64 (d, ArH, 1H, J_{HH} = 8.8 Hz), 7.55 (dd, ArH, 1H, J_{HH} = 8.8 Hz, J_{HH} = 1.9 Hz), 7.18 (m, ArH and NH, 8H), 6.46 (d, NH, 1H, J_{HH} = 7.7 Hz), 4.84 (m, CHNH, 2H), 4.61 (d, OCH, 1H, J_{HH} = 14.8 Hz), 4.53 (d, OCH, 1H, J_{HH} = 14.8 Hz), 4.20 (q, CH_2, 2H, J_{HH} = 7.2 Hz), 3.16 (dd, CHPh, 1H, J_{HH} = 14.0 Hz, J_{HH} = 5.8 Hz), 3.07 (dd, CHPh, 1H, J_{HH} = 14.0 Hz, J_{HH} = 6.4 Hz), 1.42 (d, CH_3, 3H, J_{HH} = 7.0 Hz), 1.27 (t, CH_3, 3H, J_{HH} = 7.2 Hz) ppm. ^{13}C NMR ($CDCl_3$) 171.1, 167.7, 155.2, 135.6, 132.7, 130.6, 130.1, 129.7, 129.3, 129.1, 128.6, 128.5, 127.2, 119.3, 118.0, 107.6, 37.1, 31.6, 53.2, 48.3, 37.4, 15.0, 14.1 ppm. MS (ES) 549 ($[M+Na]^+$). Accurate mass calculated for $C_{26}H_{27}N_2O_5Na^{79}Br$: 549.1001. Found: 549.1019.

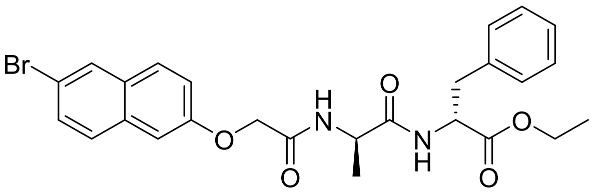

BrNapAFOH (62): ^{1}H NMR (DMSO) 8.26 (d, NH, 1H, J_{HH} = 7.9 Hz), 8.14 (d, NH, 1H, J_{HH} = 8.0 Hz), 7.86 (d, ArH, 1H, J_{HH} = 9.0 Hz), 7.76 (d, ArH, 1H, J_{HH} = 8.9 Hz), 7.57 (dd, ArH, 1H, J_{HH} = 8.7 Hz, J_{HH} = 2.2 Hz), 7.29 (m, ArH, 8H), 4.65 (d, OCH, 1H, J_{HH} = 14.8 Hz), 4.61 (d, OCH, 1H, J_{HH} = 14.8 Hz), 4.43 (m, CHNH, 2H), 3.04 (dd, CHPh, 1H, J_{HH} = 13.9 Hz, J_{HH} = 5.2 Hz), 2.89 (dd, CHPh, 1H, J_{HH} = 13.9 Hz, J_{HH} = 8.8 Hz), 1.24 (d, CH_3, 3H, J_{HH} = 7.0 Hz) ppm. ^{13}C NMR (DMSO) 172.7, 171.8, 166.8, 155.9, 137.4, 132.6, 129.9, 129.4, 129.3, 129.1, 128.9, 128.6, 128.1, 126.4, 119.7, 116.6, 107.4, 66.7, 53.5, 47.6, 36.5, 18.4 ppm. MS (ES) 521 ($[M+Na]^+$). Accurate mass calculated for $C_{24}H_{23}N_2O_5Na^{79}Br$: 521.0688. Found: 521.0685.

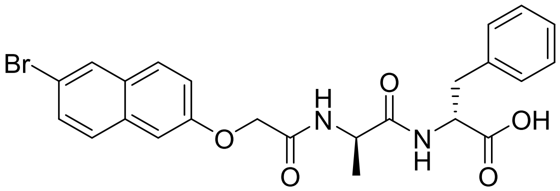

CarbFVOMe: ^{1}H NMR ($CDCl_3$) 8.11 (d, ArH, 2H, J_{HH} = 7.8 Hz), 7.44 (m, ArH, 2H), 7.30 (m, ArH, 4H), 7.24 (t, ArH, 2H, J_{HH} = 7.7 Hz), 7.06 (m, ArH, 1H), 6.97 (t, ArH, 2H, J_{HH} = 7.7 Hz), 6.68 (d, NH, 1H, J_{HH} = 8.6 Hz), 5.92 (d, NH, 1H, J_{HH} = 7.9 Hz), 4.93 (d, NCH, 1H, J_{HH} = 18.1 Hz), 4.86 (d, NCH, 1H, J_{HH} = 18.1 Hz), 4.66 (dt, CHNH, 1H, J_{HH} = 7.9 Hz, J_{HH} = 6.7 Hz), 4.30 (dd, CHNH, 1H, J_{HH} = 8.6 Hz, J_{HH} = 4.8 Hz), 3.73 (s, OCH_3, 3H), 2.83 (d, CH_2Ph, 2H, J_{HH} = 6.7 Hz), 2.01 (m, $CH(CH_3)_2$, 1H), 0.76 (d, CH_3, 3H, J_{HH} = 6.9 Hz), 0.72 (d, CH_3, 3H, J_{HH} = 6.9 Hz) ppm. ^{13}C NMR ($CDCl_3$) 171.9, 169.8, 168.4, 140.0, 135.3, 129.3, 128.8, 128.6, 126.9, 136.6, 123.5, 120.7, 120.4, 108.3, 57.2, 54.1, 52.2, 46.8, 36.9, 34.4, 48.8, 17.8 ppm. MS (ES) 508 ($[M+Na]^+$). Accurate mass calculated for $C_{29}H_{31}N_3O_4Na$: 508.2212. Found: 508.2201.

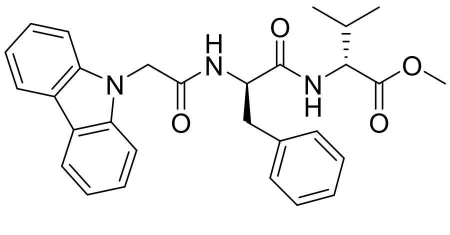

CarbFVOH (63): ^{1}H NMR (DMSO) 8.06 (d, NH, 1H, J_{HH} = 8.6 Hz), 8.16 (d, NH, 1H, J_{HH} = 8.4 Hz), 7.10 (d, ArH, 2H, J_{HH} = 7.7 Hz), 7.38 – 7.16 (m, ArH, 11H), 5.04 (d, NCH, 1H, J_{HH} = 17.8 Hz), 4.92 (d, NCH, 1H, J_{HH} = 17.8 Hz), 4.71 (m, CHNH, 1H), 4.14 (dd, CHNH, 1H, J_{HH} = 8.4 Hz, J_{HH} = 5.7 Hz), 3.06 (dd, CHPh, 1H,

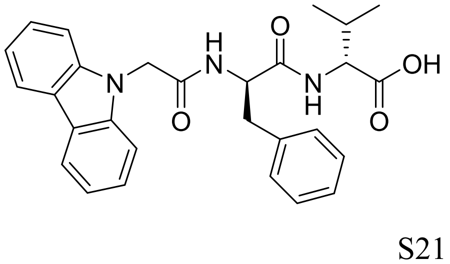

J_{HH} = 13.8 Hz, J_{HH} = 4.0 Hz), 2.81 (dd, CHPh, 1H, J_{HH} = 13.8 Hz, J_{HH} = 10.1 Hz), 2.06 (m, $CH(CH_3)_2$, 1H), 0.86 (d, CH_3, 6H) ppm. ^{13}C NMR (DMSO) 172.8, 171.1, 167.1, 140.4, 137.6, 129.3, 1280, 126.3, 125.6, 122.0, 119.9, 118.9, 109.3, 57.5, 53.5, 45.2, 37.7, 19.1, 18.0 ppm. MS (ES) 494 ($[M+Na]^+$). Accurate mass calculated for $C_{28}H_{29}N_3O_4Na$: 494.2056. Found: 494.2047.

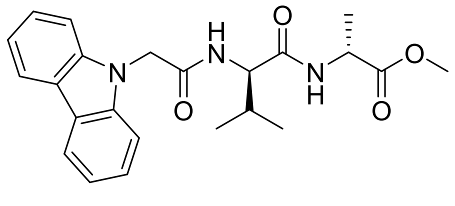

CarbVAOMe: ^1H NMR ($CDCl_3$) 8.12 (d, ArH, 2H, J_{HH} = 7.7 Hz), 7.50 (m, ArH, 2H), 7.37 (d, ArH, 2H, J_{HH} = 8.2 Hz), 7.31 (m, ArH, 2H), 6.17 (NH, 1H J_{HH} = 7.0 Hz), 5.94 (d, NH, 1H, J_{HH} = 8.4 Hz), 4.01 (d, NCH, 1H, J_{HH} = 18.1 Hz), 4.94 (d, NCH, 1H, J_{HH} = 18.1 Hz), 4.45 (m, CHNH, 1H), 4.20 (dd, CHNH, 1H, J_{HH} = 8.7 Hz, J_{HH} = 6.3 Hz), 3.72 (s, OCH_3, 3H), 1.93 (m, $CH(CH_3)_2$, 1H), 1.30 (d, CH_3, 3H, J_{HH} = 7.2 Hz), 0.74 (d, CH_3, 3H, J_{HH} = 6.8 Hz), 0.51 (d, CH_3, 3H, J_{HH} = 6.8 Hz) ppm. ^{13}C NMR (DMSO) 172.8, 170.7, 167.3, 140.5, 125.6, 122.1, 120.9, 118.9, 109.3, 56.9, 51.7, 47.5, 45.2, 31.0, 19.0, 17.9, 16.7 ppm. MS (ES) 432 ($[M+Na]^+$). Accurate mass calculated for $C_{23}H_{27}N_3O_4Na$: 432.1899. Found: 432.1891.

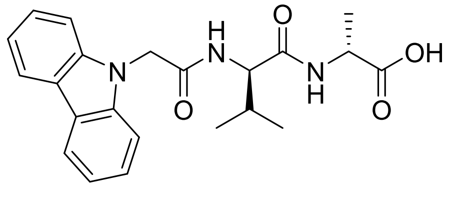

CarbVAOH (64): ^1H NMR (DMSO) 8.38 (d, NH, 1H, J_{HH} = 9.2 Hz), 8.35 (d, NH, 1H, J_{HH} = 6.9 Hz), 8.14 (d, ArH, 2H, J_{HH} = 7.7 Hz), 7.55 (d, ArH, 2H, J_{HH} = 8.2 Hz), 7.43 (m, ArH, 2H), 7.20 (m, ArH, 2H), 5.18 (d, NCH, 1H, J_{HH} = 16.7 Hz), 5.12 (d, NCH, 1H , J_{HH} = 16.7 Hz), 4.26 (dd, CHNH,1H, J_{HH} = 9.1 Hz, J_{HH} = 6.6 Hz), 4.20 (m, CHNH, 1H), 2.01 (m, $CH(CH_3)_2$, 1H), 1.26 (d, CH_3, 3H, J_{HH} = 7.3 Hz), 0.87 (m, CH_3, 6H) ppm. ^{13}C NMR (DMSO) 173.9, 170.5, 167.3, 14.6, 125.6, 122.1, 120.1, 118.9, 109.4, 57.0, 47.4, 45.2, 31.1, 19.1, 17.9, 16.9 ppm. MS (ES) 418 ($[M+Na]^+$). Accurate mass calculated for $C_{22}H_{25}N_3O_4Na$: 418.1743. Found: 418.1733.

Section 2

Gelation tests: The gelation was probed using the following method. The molecule of choice was dispersed in deionised water. Sodium hydroxide (1 molar equivalent of a 0.1 M solution) was added such that the final concentration of the molecule was 5 mg/mL. To 2 mL of this solution was added 16 mg glucono-δ-lactone (GdL). After swirling briefly to dissolve the GdL, the sample was allowed to stand at room temperature overnight (18 hours). At this point, the sample was determined to be a transparent gel (G), a turbid gel (TG), or a non-gelling precipitate (P) or non-gelling crystals (C). The results are shown in Table S1. Set refers to whether the compound is in the training (Tr), testing (Te) or validation (V) set.

**Compound**	**Set**	**Gelation?**	**Compound**	**Set**	**Gelation?**	**Compound**	**Set**	**Gelation?**
**1**	Te	P	**22**	Tr	P	**43**	Tr	C
**2**	Te	P	**23**	Tr	G	**44**	Tr	G
**3**	Te	P	**24**	Tr	G	**45**	Tr	P
**4**	Te	G	**25**	Tr	G	**46**	V	C
**5**	Te	TG	**26**	Te	P	**47**	V	P
**6**	Te	P	**27**	Te	P	**48**	Tr	P
**7**	Tr	C	**28**	Te	P	**49**	Tr	P
**8**	Tr	C	**29**	Te	G	**50**	Tr	P
**9**	Tr	G	**30**	Te	G	**51**	V	P
**10**	Tr	G	**31**	Tr	C	**52**	V	P
**11**	Tr	G	**32**	Te	P	**53**	Te	P
**12**	Tr	G	**33**	Te	C	**54**	V	P
**13**	Tr	G	**34**	Te	P	**55**	Tr	G
**14**	Tr	G	**35**	Te	C	**56**	V	TG
**15**	Tr	G	**36**	Te	P	**57**	Te	TG
**16**	Tr	G	**37**	Te	P	**58**	V	G
**17**	Tr	G	**38**	Te	C	**59**	Tr	P
**18**	Tr	P	**39**	Tr	P	**60**	Tr	P
**19**	Tr	G	**40**	Tr	P	**61**	Tr	P
**20**	Tr	TG	**41**	Te	P	**62**	Tr	P
**21**	Tr	G	**42**	Tr	P	**63**	V	TG
						**64**	V	G

Table S1 Allocation of the compounds to the training (Tr), testing (Te) or validation (V) set. Whether the compound resulted in a transparent gel (G), a turbid gel (TG), or a non-gelling precipitate (P) or non-gelling crystals (C) is also shown.

Section 3

QSPR

The molecules were generated *in* silico using ChemDraw,[10] converted to SMILES format, The descriptors were calculated using Pipeline Pilot[11] (ALogP, Molecular_Weight, Num_Atoms, Num_RotatableBonds, Num_Rings, Num_H_Acceptors, Num_H_Donors, Molecular_SurfaceArea, Molecular_PolarSurfaceArea, Molecular_PolarSASA, Molecular_Solubility, LogD, ECFP4 and FCFP4). Molecular fingerprint descriptors ECFP4 and FCFP4 were converted to a list of integer bits 2048 bits long with the least significant bit packed first.[12]

Visualisation was achieved using the PCA,[13] cMDS,[13] isoMDS[14] and Sammon[14] R libraries. Machine learning was performed using the Caret library in R.[15]

The Caret (Classification and Regression Training)[15] library in R[13] was used to remove descriptors with near zero variance (constant or almost constant values throughout all the samples) and highly correlated descriptors ($r^2 \geq 0.9$), reducing the number of descriptors to analyse and the remaining 57 descriptors auto-scaled. This set of 57 descriptors were used for both the visualisation and machine learning methods.

Model training was performed using pre-processing of centring and scaling, tunelength of 10 with a 5-fold cross validation repeated 50 times using H measure as an objective function.

Previous recommendations for structure-property modelling have been published.[16] Below in Table S2, we map onto these five points this current work.

Recommendation	This work
A defined end point	Formation of gel using approach described in experimental section
An unambiguous algorithm	Methods fully described in experimental and results and discussion section
A defined domain of applicability	Domain of applicability is defined in experimental section
Appropriate measures of goodness-of–fit, robustness and predictivity	Measures described and justified in experimental and results and discussion section
A mechanistic interpretation, if possible	Interpretation suggested in results and discussion section

Table S2. OECD QSPR Guidelines and how this work maps onto them

Visualising and exploring "gelator space"

The search for new molecules that can form gels can be referred to in terms of exploration and navigation through "chemical space".[17] "Chemical space" has been defined as the set of all possible molecules and their associated properties in multi-dimensional space. Visualisation techniques can be extremely powerful tools for picturing many molecules at once and understanding trends in their properties. Chemical space visualizations show each molecule as a point within a plot and permits visualisation of where a compound resides within chemical space using descriptors to define the coordinates. There are almost limitless possible ways of visualising chemical space with no one single representation being suitable for all purposes. Further to this, different representations of chemical space will be of different utility for each researcher. The simplest approaches to visualisation of "gelator space" use a few simple parameters related to molecular properties. The perceived closeness of the points in chemical space may reflect the chemist's idea of similarity of the molecules under consideration. A useful representation of chemical space would very quickly enable identification of areas of chemical space that are rich in gelators whilst also highlighting areas that are predicted to contain few gel forming compounds. Consequently, several visualisation techniques were investigated in order to map areas of gelator space.

If such a relatively simple and powerful relationship existed (as it does in other fields, e.g. Lipinski Guidelines in drug discovery[18]), visualization of datasets with such descriptor coordinates would be easily interpretable and very intuitive. However, as can be seen in Figure S2, for the training set across the 12 molecular properties shown there is not a simple relationship between one single descriptor and the ability of the molecule to gel, i.e. the distributions of gelator/non-gelator for each molecular property all overlap to some extent. The same is true when molecular fingerprints are examined in the same way (see Figure S3). Therefore, as perhaps expected from the difficulty in generating design rules for gelators, it is through a complex interplay of molecular properties that the gelation of a molecule is exercised. Hence, we hypothesise that it is via more sophisticated techniques such as mapping chemical space and and machine learning that we will be able to develop models that are truly predictive on the ability of compounds to form gels.

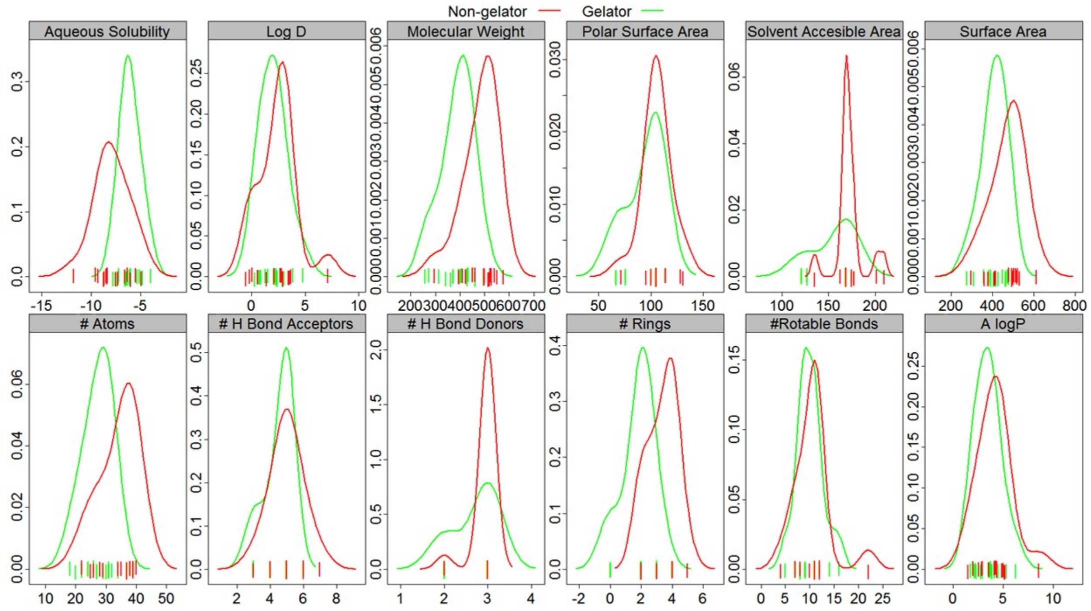

Figure S2. Plot of 12 physicochemical descriptors calculated and their distributions on forming (green) or not forming (red) a gel for the training set. The value of each molecule's descriptor is depicted by | marker.

In map-based representations of the chemical space, the multi-dimensional descriptor spaces of the compounds are folded (mapped) into the two-dimensional plot space, similar to the construction of flat Earth maps from three-dimensional data.[17] Data projection techniques provide a route to condense a large amount of multi-dimensional chemical information, i.e. all the molecular properties calculated can be reduced into a smaller number of dimensions in order to aid visualisation and analysis.

Principal component analysis (PCA) is such an approach. It is a simple non-parametric method of extracting relevant information from complex data that are often confusing, clouded, or even redundant. PCA transforms a number of possibly interdependent or correlated variables into a smaller number of significant, independent, and uncorrelated orthogonal components[19]. Each principal component is a linear combination of the molecular properties being considered. A principal component analysis of the 34 molecules in the training set and the all the descriptors, revealed that 48.54% of the total variance of the molecular descriptors is captured in the first three principal components. A plot of the first two components (Figure S3a) shows that there is not a clear demarcation within principal component space which separates out gelators from non-gelators completely, i.e. there is no clear clustering in any of the plots of gelators/non-gelators that completely separates gelators from non-gelators.

Another approach for viewing "gelator space" via dimensionality reduction is to utilise multidimensional scaling techniques, which attempt to transform a set of data points to fewer dimensions in such a way that the distances between points are preserved as much as possible. Despite their lower visual interpretability compared with PCA, the maps allow better clustering of the compounds and possess higher predictive ability.[17]

If the data are reduced to two dimensions, this allows one to visualize the compounds and see by inspection the relative distances between them - similar molecules would cluster together. Three different methods (classical, Kruskal and Sammon, Figure S3 b, c and d) were used in order to see if a clear distinction between gelator and non-

gelator space for the training set. However, visually there is no clustering in any of the plots of gelators/non-gelators that completely separates gelators from non-gelators.

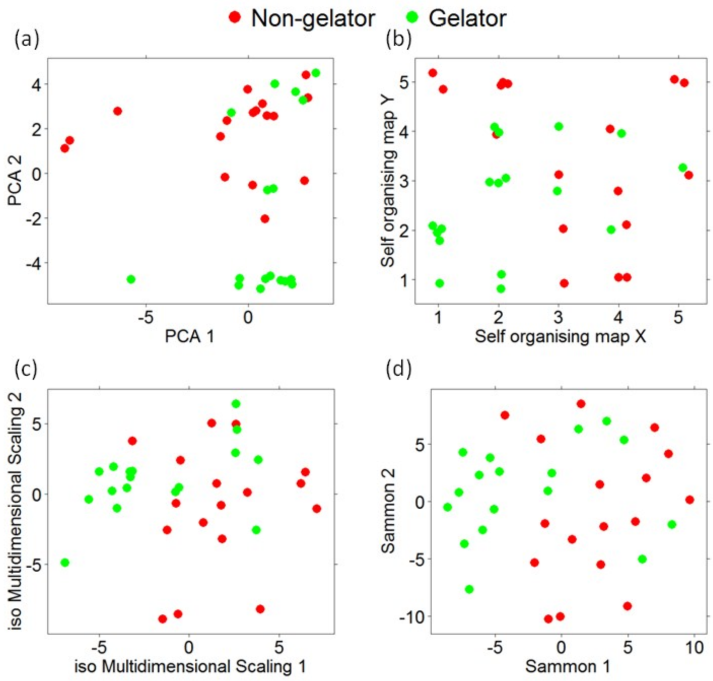

Figure S3. a) PCA, b) Classical, c) Kruskal and d) Sammon plots of forming (green) or not forming (red) a gel. Data shown for the training set.

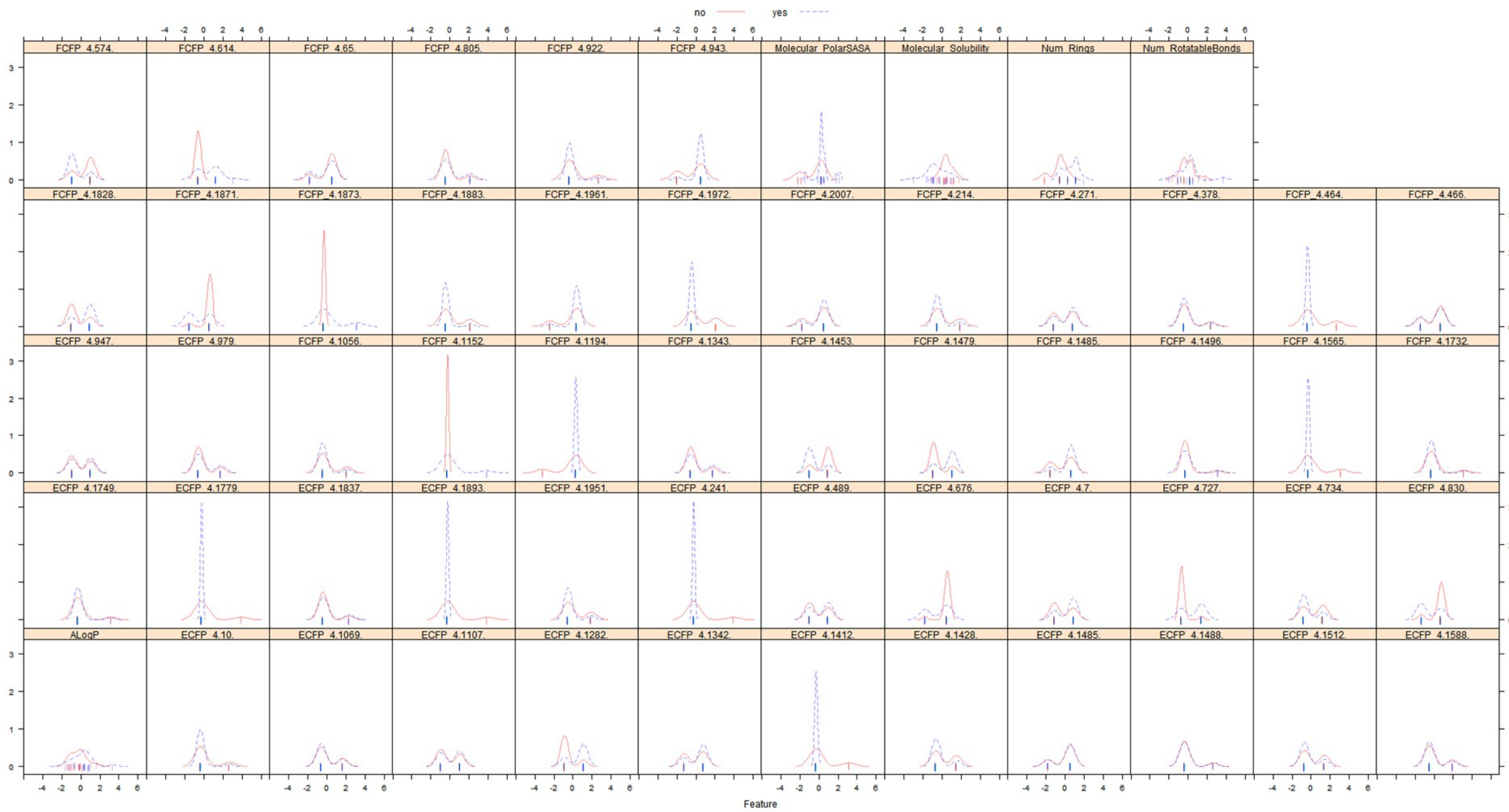

Figure S4 Plot of the 57 descriptors and their distributions on forming (blue dotted) or not forming (red solid) a gel

Random Forest
Model Summary plots

i – model tuning, ii – distribution of H measure for cross validated models, iii – ROC curve for optimal model, iv – descriptor importance

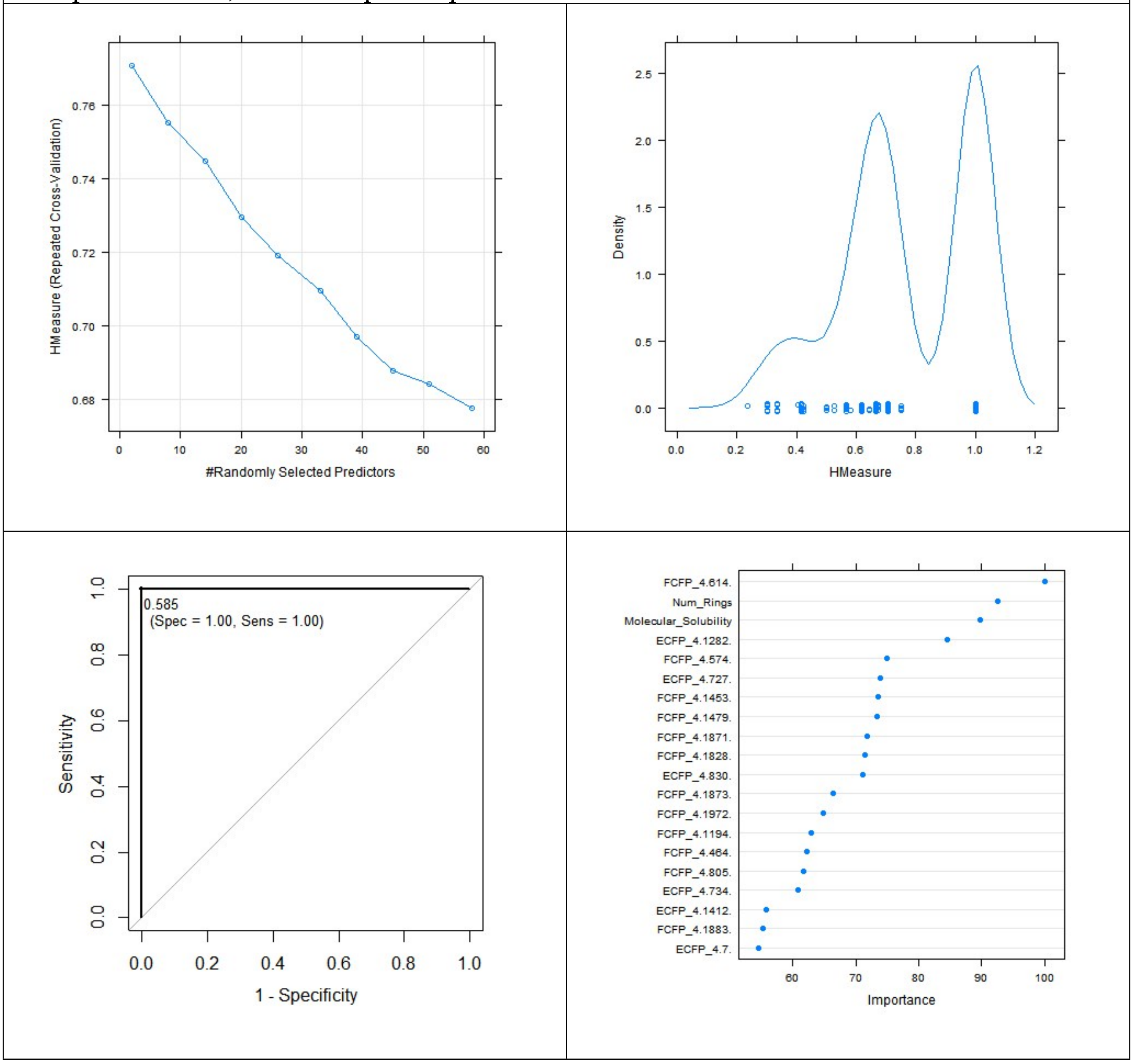

Figure S5 Model summary plot for the Random Forest model

Support Vector Machines
Model Summary plots

i – model tuning, ii – distribution of H measure for cross validated models, iii – ROC curve for optimal model, iv – descriptor importance

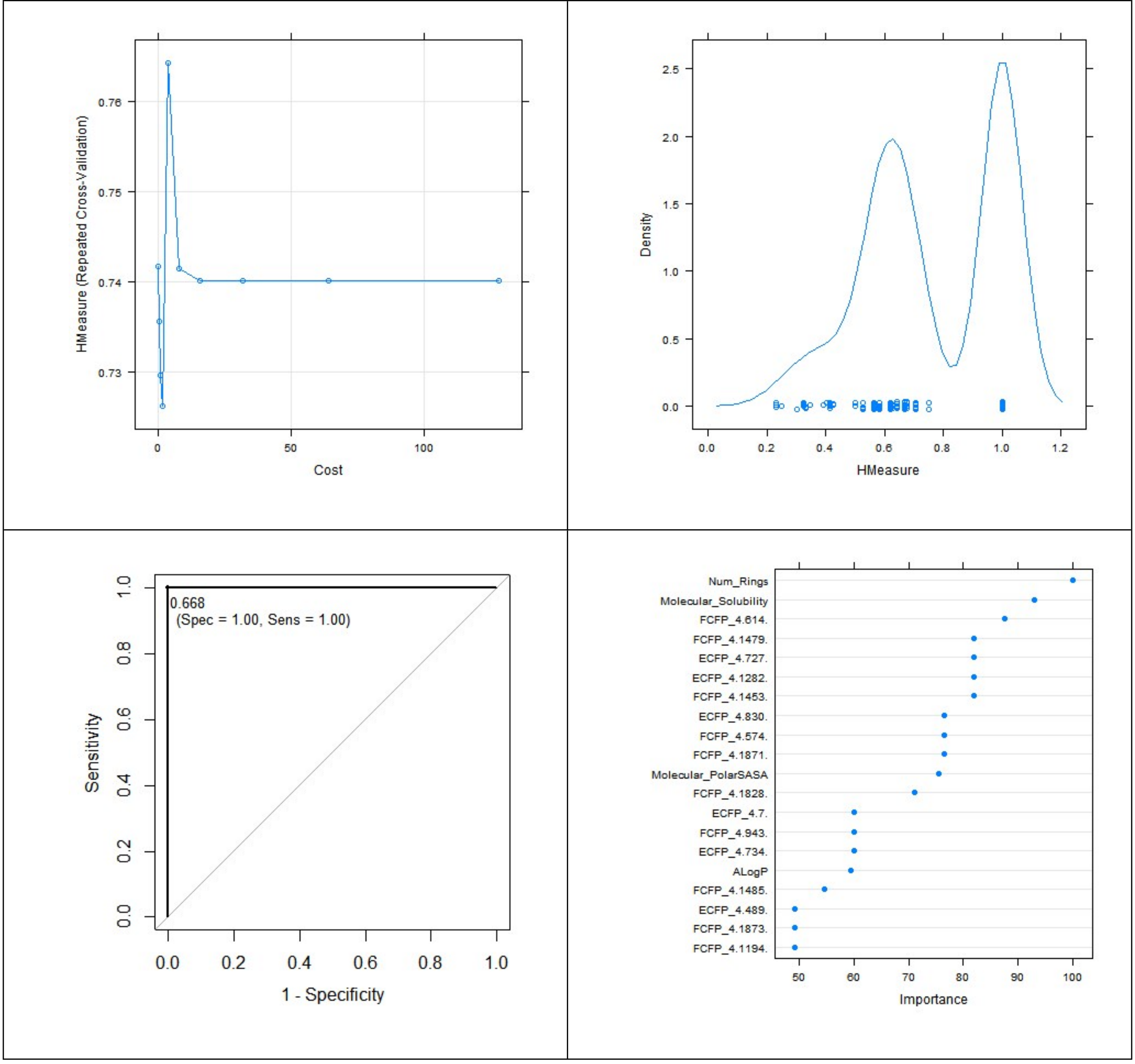

Figure S6 Model summary plot for the Support Vector Machines model

***k*NN**

Model Summary plots

i – model tuning, ii – distribution of H measure for cross validated models, iii – ROC curve for optimal model, iv – descriptor importance

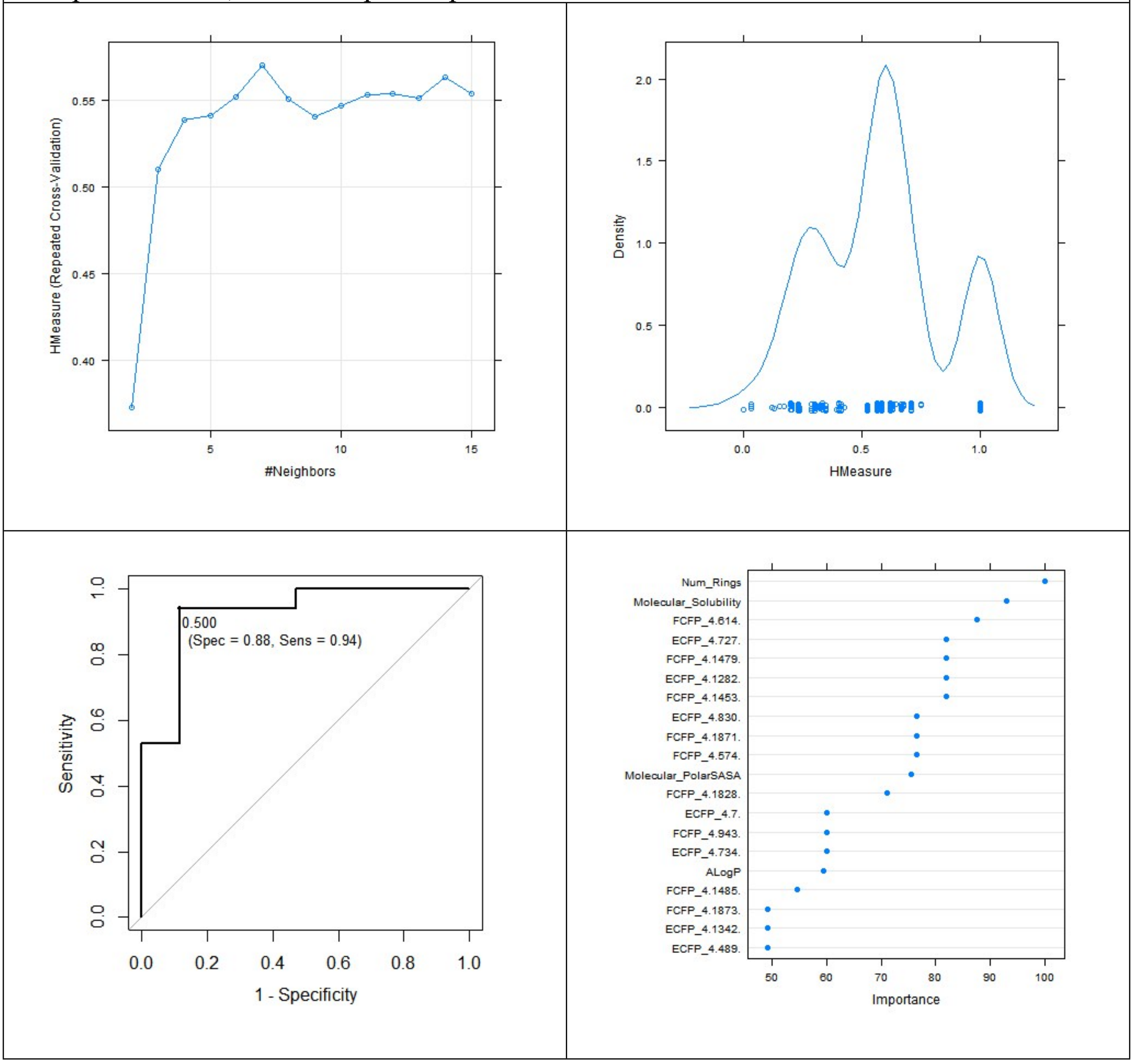

Figure S7 Model summary plot for the *k*NN model

Neural Network
Model Summary plots

i – model tuning, ii – distribution of H measure for cross validated models, iii – ROC curve for optimal model, iv – descriptor importance

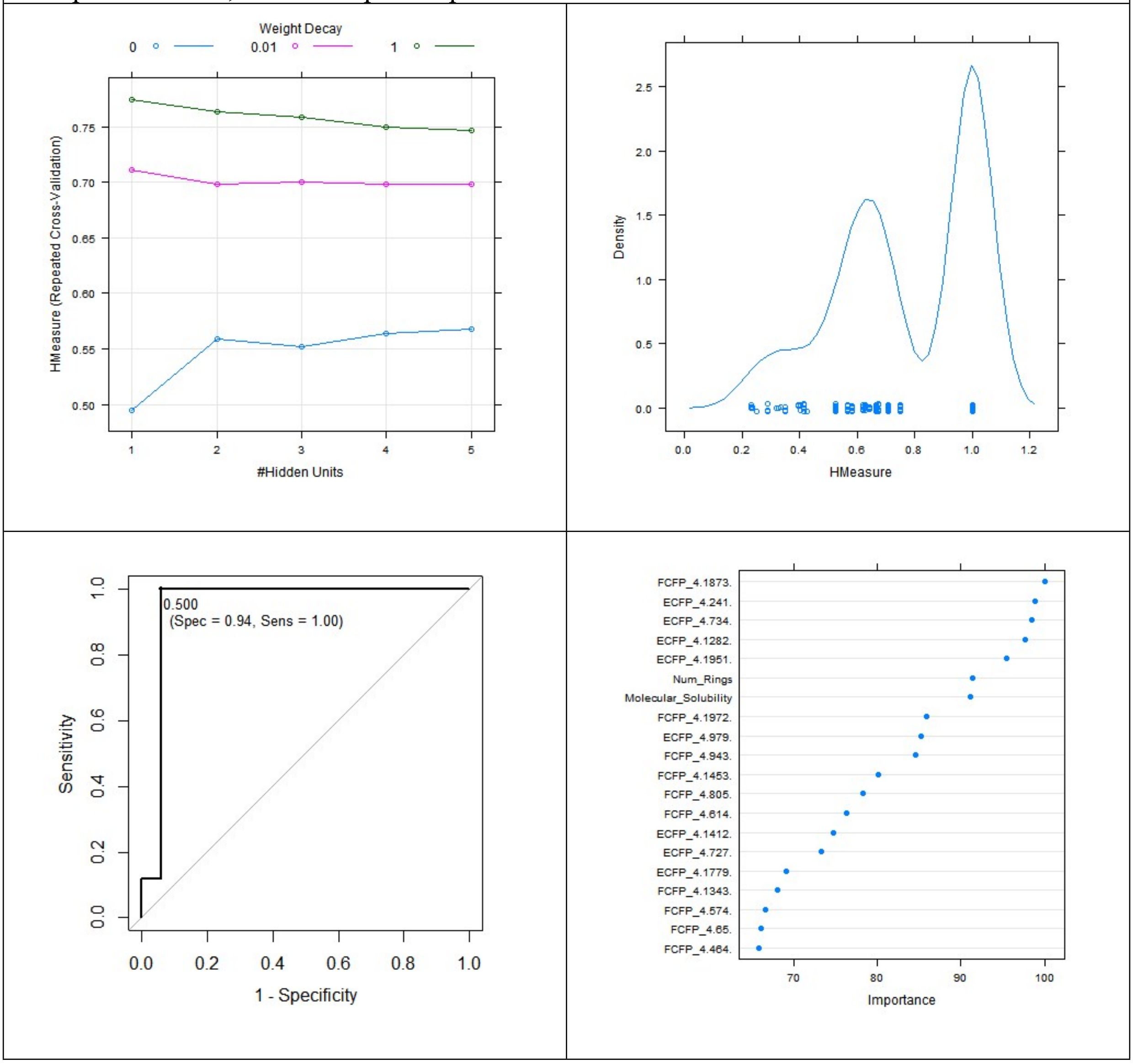

Figure S8 Model summary plot for the Neural Network model

PLS
Model Summary plots

i – model tuning, ii – distribution of H measure for cross validated models, iii – ROC curve for optimal model, iv – descriptor importance

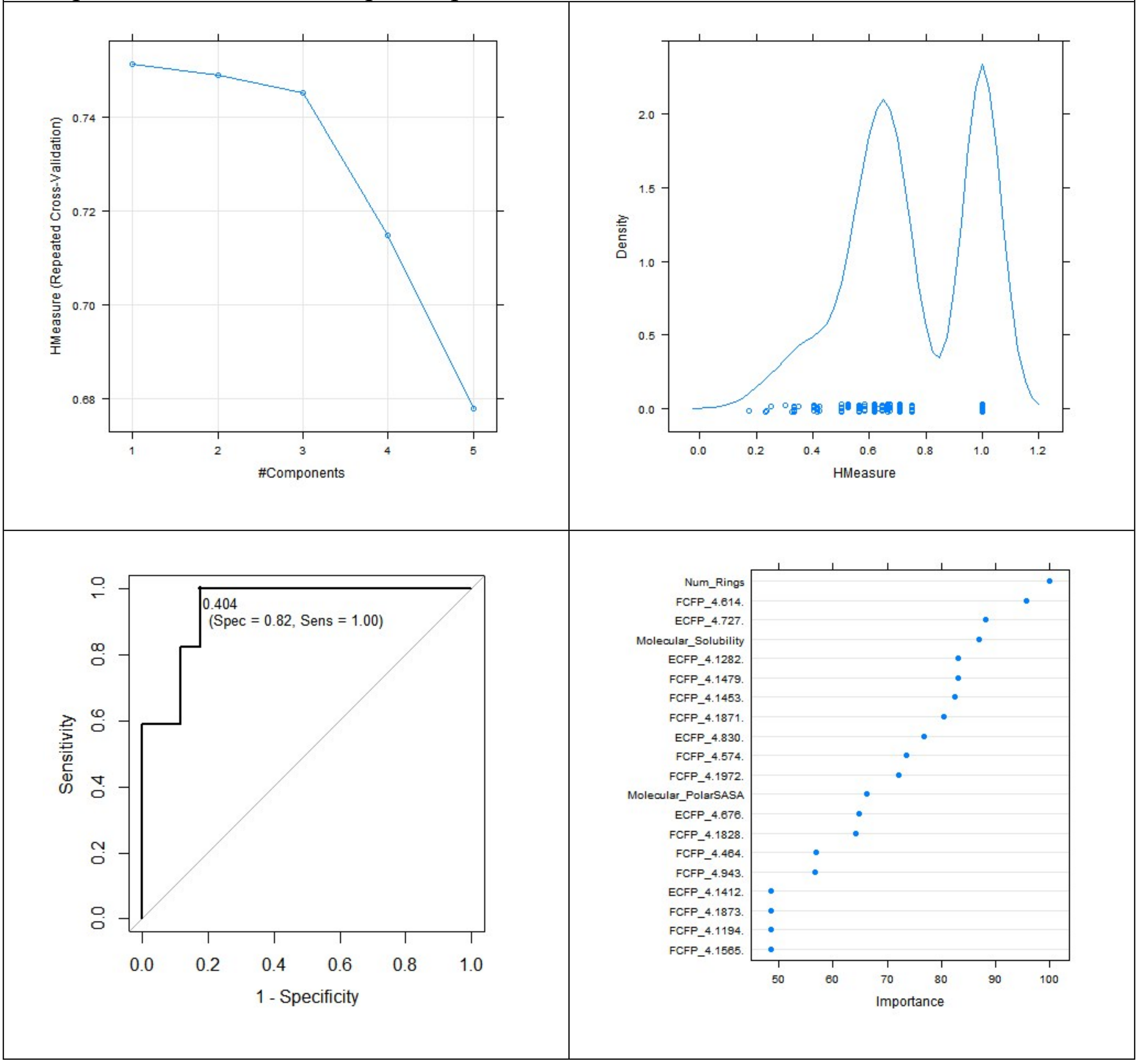

Figure S9 Model summary plot for the PLS model

Naïve Bayes
Model Summary plots

i – model tuning, ii – distribution of H measure for cross validated models, iii – ROC curve for optimal model, iv – descriptor importance

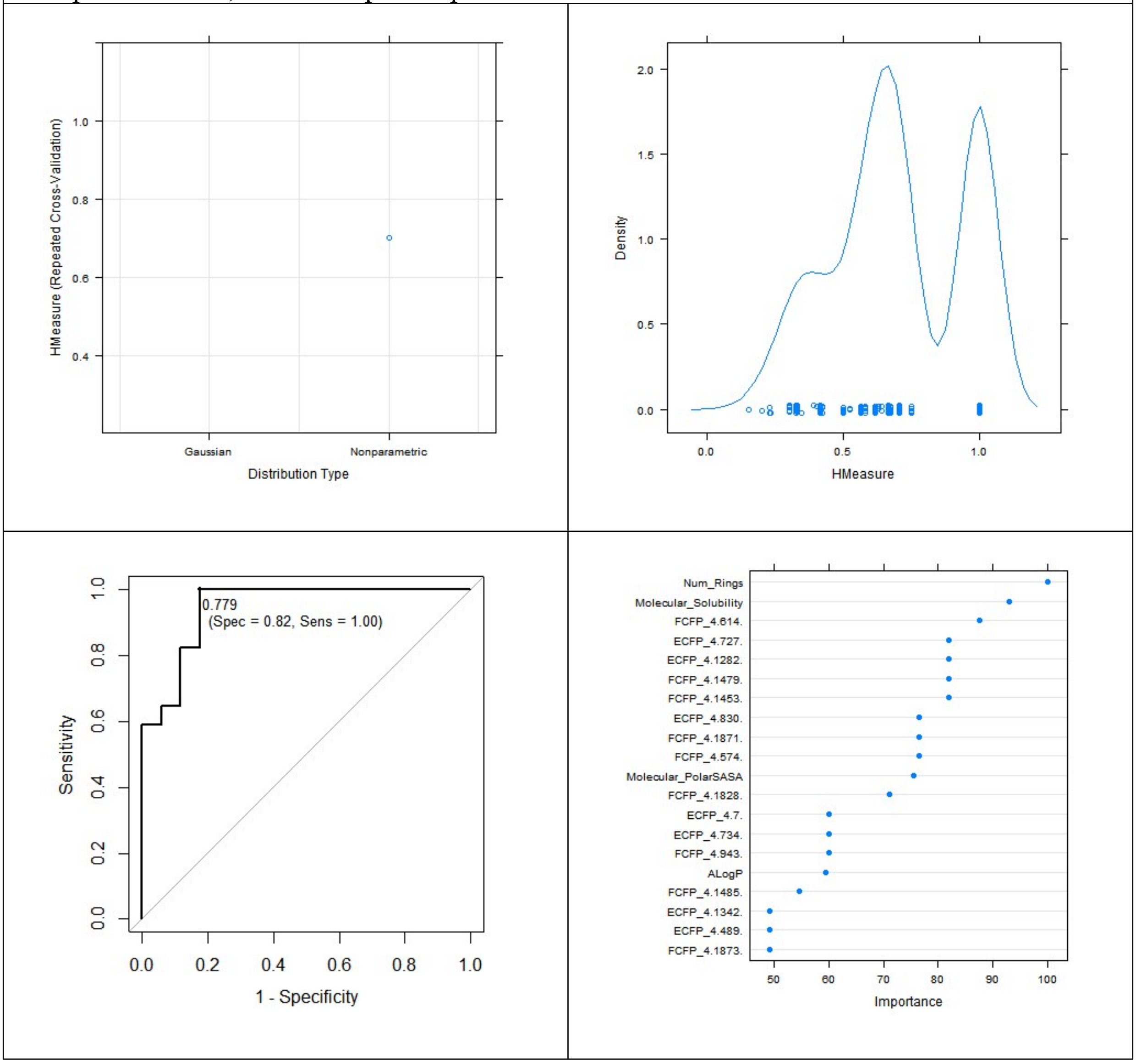

Figure S10 Model summary plot for the Naïve Bayes model

C5.0

Model Summary plots

i – model tuning, ii – distribution of H measure for cross validated models, iii – ROC curve for optimal model, iv – descriptor importance

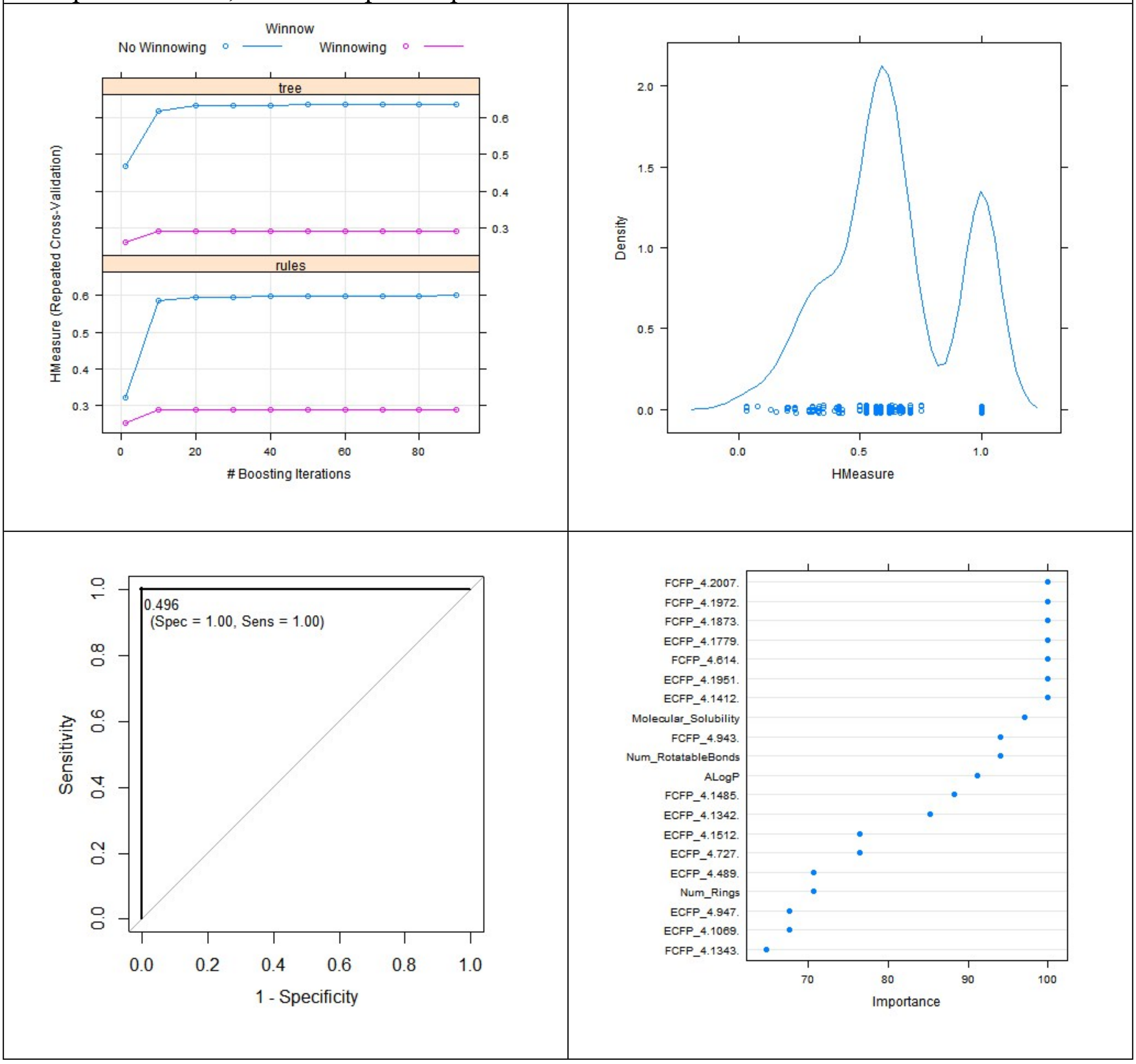

Figure S11 Model summary plot for the C5.0 model

Flexible Discriminant Analysis
Model Summary plots

i – model tuning, ii – distribution of H measure for cross validated models, iii – ROC curve for optimal model, iv – descriptor importance

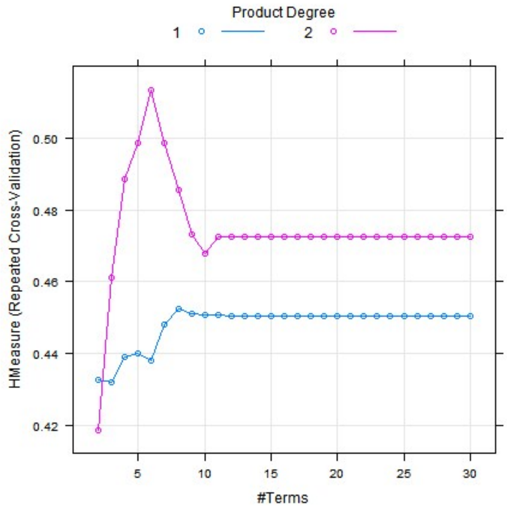

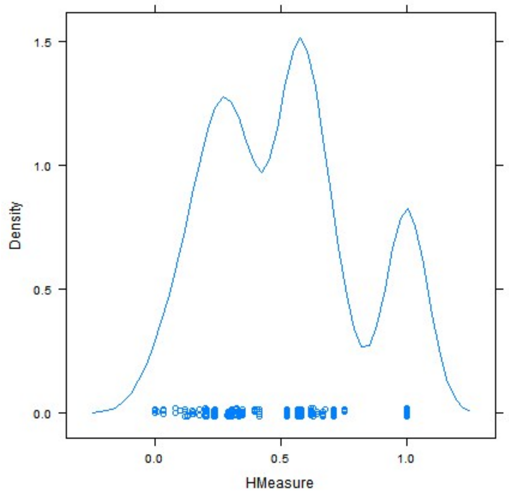

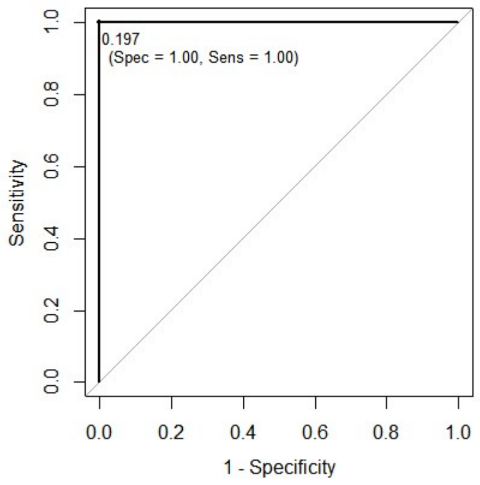

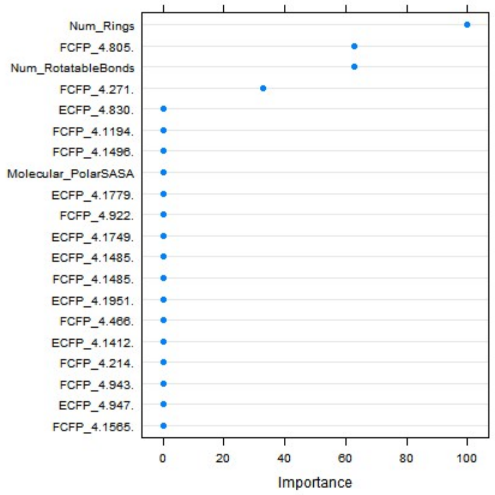

Figure S12 Model summary plot for the Flexible Discriminant Analysis model

	Performance on external test set of all 21 compounds			
Method	Kappa	Balanced Accuracy	H measure	Quality of predictions
SVM	0.577	0.816	0.778	Good
RF	0.859	0.971	0.891	Good
kNN	0.577	0.8324	0.587	Good
NN	0.553	0.882	1	Good
PLS	0.411	0.824	0.729	Good
NB	0.400	0.824	0.729	Good
C5.0	0.013	0.507	0.198	Bad

Table S3 Performance of the models predicting the gelator properties of the 20 external test set compounds both within and outside the model domain of applicability using models generated from true training set data. Green – meets criteria. Red – fails criteria. (Criteria for good: H > 0.6, Kappa > 0.4, Balanced accuracy > 0.7).

Method	Resampling results of optimal model using scrambled data	Performance of optimal model selected by repeated 5-fold cross-validation			
	H measure ± SD	Kappa	Balanced Accuracy	H measure	Quality of model
**SVM**	0.5343± 0.24	0.235	0.618	0.133	Bad
**RF**	0.524 ± 0.24	-0.059	0.471	0.109	Bad
**kNN**	0.401 ± 0.25	-0.294	0.353	0.231	Bad
**NN**	0.517 ± 0.23	0.058	0.529	0.180	Bad
**PLS**	0.565 ± 0.26	0.059	0.529	0.180	Bad
**NB**	0.545 ± 0.23	0.146	0.5	0.158	Bad
**C5.0**	0.481 ± 0.25	-0.059	0.471	0.227	Bad

Table S4 Performance of the models generated from scrambled training set data. Green – meets criteria. Red – fails criteria. (Criteria for good: H > 0.6, Kappa > 0.4, Balanced accuracy > 0.7).

Method	Performance on external test set of 14 compounds within models domain of applicability using models built on randomised data			
	Kappa	Balanced Accuracy	H measure	Quality of predictions
SVM	-0.068	0.417	0.334	Bad
RF	0.000	0.500	0.315	Bad
*k*NN	0103	0.583	0.180	Bad
NN	0.417	0.708	0.396	Bad
PLS	0.170	0.708	0.359	Bad
NB	-0.235	0.333	0.406	Bad
C5.0	0.025	0.541	0.007	Bad

Table S5 Performance of the models predicting the gelator properties of the 13 external test set compounds within the model domain of applicability using models generated from scrambled training set data. Green – meets criteria. Red – fails criteria. (Criteria for good: H > 0.6, Kappa > 0.4, Balanced accuracy > 0.7).

2025 Compound Virtual Library

Compound codes in right hand column can be used to request predictions from the authors.

C%11(COC1=CC=C(Cl)C2=CC=CC=C21)=O.O=C%10CN%11.O=C(O)CN%10	1.1_1
C%11(COC1=CC=C(Cl)C2=CC=CC=C21)=O.O=C%10CN%11.CC(C)[CH](C(O)=O)N%10	1.1_2
C%11(COC1=CC=C(Cl)C2=CC=CC=C21)=O.O=C%10CN%11.CC(C)C[CH](C(O)=O)N%10	1.1_3
C%11(COC1=CC=C(Cl)C2=CC=CC=C21)=O.O=C%10CN%11.C[CH](C(O)=O)N%10	1.1_4
C%11(COC1=CC=C(Cl)C2=CC=CC=C21)=O.O=C%10CN%11.O=C(O)[CH](CC1=CC=CC=C1)N%10	1.1_5
C%11(COC1=CC=C(Cl)C2=CC=CC=C21)=O.O=C%10CN%11.CC[CH](C)[CH](C(O)=O)N%10	1.1_6
C%11(COC1=CC=C(Cl)C2=CC=CC=C21)=O.O=C%10CN%11.CSCC[CH](C(O)=O)N%10	1.1_7
C%11(COC1=CC=C(Cl)C2=CC=CC=C21)=O.O=C%10CN%11.OC(C=C1)=CC=C1C[CH](C(O)=O)N%10	1.1_8
C%11(COC1=CC=C(Cl)C2=CC=CC=C21)=O.O=C%10CN%11.O=C(O)[CH](CC1=C(F)C(F)=C(F)C(F)=C1F)N%10	1.1_9
C%11(COC1=CC=C(Cl)C2=CC=CC=C21)=O.CC(C)[CH](C%10=O)N%11.O=C(O)CN%10	1.2_1
C%11(COC1=CC=C(Cl)C2=CC=CC=C21)=O.CC(C)[CH](C%10=O)N%11.CC(C)[CH](C(O)=O)N%10	1.2_2
C%11(COC1=CC=C(Cl)C2=CC=CC=C21)=O.CC(C)[CH](C%10=O)N%11.CC(C)C[CH](C(O)=O)N%10	1.2_3
C%11(COC1=CC=C(Cl)C2=CC=CC=C21)=O.CC(C)[CH](C%10=O)N%11.C[CH](C(O)=O)N%10	1.2_4
C%11(COC1=CC=C(Cl)C2=CC=CC=C21)=O.CC(C)[CH](C%10=O)N%11.O=C(O)[CH](CC1=CC=CC=C1)N%10	1.2_5
C%11(COC1=CC=C(Cl)C2=CC=CC=C21)=O.CC(C)[CH](C%10=O)N%11.CC[CH](C)[CH](C(O)=O)N%10	1.2_6
C%11(COC1=CC=C(Cl)C2=CC=CC=C21)=O.CC(C)[CH](C%10=O)N%11.CSCC[CH](C(O)=O)N%10	1.2_7
C%11(COC1=CC=C(Cl)C2=CC=CC=C21)=O.CC(C)[CH](C%10=O)N%11.OC(C=C1)=CC=C1C[CH](C(O)=O)N%10	1.2_8
C%11(COC1=CC=C(Cl)C2=CC=CC=C21)=O.CC(C)[CH](C%10=O)N%11.O=C(O)[CH](CC1=C(F)C(F)=C(F)C(F)=C1F)N%10	1.2_9
C%11(COC1=CC=C(Cl)C2=CC=CC=C21)=O.CC(C)C[CH](C%10=O)N%11.O=C(O)CN%10	1.3_1
C%11(COC1=CC=C(Cl)C2=CC=CC=C21)=O.CC(C)C[CH](C%10=O)N%11.CC(C)[CH](C(O)=O)N%10	1.3_2
C%11(COC1=CC=C(Cl)C2=CC=CC=C21)=O.CC(C)C[CH](C%10=O)N%11.CC(C)C[CH](C(O)=O)N%10	1.3_3
C%11(COC1=CC=C(Cl)C2=CC=CC=C21)=O.CC(C)C[CH](C%10=O)N%11.C[CH](C(O)=O)N%10	1.3_4
C%11(COC1=CC=C(Cl)C2=CC=CC=C21)=O.CC(C)C[CH](C%10=O)N%11.O=C(O)[CH](CC1=CC=CC=C1)N%10	1.3_5
C%11(COC1=CC=C(Cl)C2=CC=CC=C21)=O.CC(C)C[CH](C%10=O)N%11.CC[CH](C)[CH](C(O)=O)N%10	1.3_6
C%11(COC1=CC=C(Cl)C2=CC=CC=C21)=O.CC(C)C[CH](C%10=O)N%11.CSCC[CH](C(O)=O)N%10	1.3_7
C%11(COC1=CC=C(Cl)C2=CC=CC=C21)=O.CC(C)C[CH](C%10=O)N%11.OC(C=C1)=CC=C1C[CH](C(O)=O)N%10	1.3_8
C%11(COC1=CC=C(Cl)C2=CC=CC=C21)=O.CC(C)C[CH](C%10=O)N%11.O=C(O)[CH](CC1=C(F)C(F)=C(F)C(F)=C1F)N%10	1.3_9

C%11(COC1=CC=C(Cl)C2=CC=CC=C21)=O.C[CH](C%10=O)N%11.O=C(O)CN%10	1.4_1
C%11(COC1=CC=C(Cl)C2=CC=CC=C21)=O.C[CH](C%10=O)N%11.CC(C)[CH](C(O)=O)N%10	1.4_2
C%11(COC1=CC=C(Cl)C2=CC=CC=C21)=O.C[CH](C%10=O)N%11.CC(C)C[CH](C(O)=O)N%10	1.4_3
C%11(COC1=CC=C(Cl)C2=CC=CC=C21)=O.C[CH](C%10=O)N%11.C[CH](C(O)=O)N%10	1.4_4
C%11(COC1=CC=C(Cl)C2=CC=CC=C21)=O.C[CH](C%10=O)N%11.O=C(O)[CH](CC1=CC=CC=C1)N%10	1.4_5
C%11(COC1=CC=C(Cl)C2=CC=CC=C21)=O.C[CH](C%10=O)N%11.CC[CH](C)[CH](C(O)=O)N%10	1.4_6
C%11(COC1=CC=C(Cl)C2=CC=CC=C21)=O.C[CH](C%10=O)N%11.CSCC[CH](C(O)=O)N%10	1.4_7
C%11(COC1=CC=C(Cl)C2=CC=CC=C21)=O.C[CH](C%10=O)N%11.OC(C=C1)=CC=C1C[CH](C(O)=O)N%10	1.4_8
C%11(COC1=CC=C(Cl)C2=CC=CC=C21)=O.C[CH](C%10=O)N%11.O=C(O)[CH](CC1=C(F)C(F)=C(F)C(F)=C1F)N%10	1.4_9
C%11(COC1=CC=C(Cl)C2=CC=CC=C21)=O.O=C%10[CH](CC1=CC=CC=C1)N%11.O=C(O)CN%10	1.5_1
C%11(COC1=CC=C(Cl)C2=CC=CC=C21)=O.O=C%10[CH](CC1=CC=CC=C1)N%11.CC(C)[CH](C(O)=O)N%10	1.5_2
C%11(COC1=CC=C(Cl)C2=CC=CC=C21)=O.O=C%10[CH](CC1=CC=CC=C1)N%11.CC(C)C[CH](C(O)=O)N%10	1.5_3
C%11(COC1=CC=C(Cl)C2=CC=CC=C21)=O.O=C%10[CH](CC1=CC=CC=C1)N%11.C[CH](C(O)=O)N%10	1.5_4
C%11(COC1=CC=C(Cl)C2=CC=CC=C21)=O.O=C%10[CH](CC1=CC=CC=C1)N%11.O=C(O)[CH](CC1=CC=CC=C1)N%10	1.5_5
C%11(COC1=CC=C(Cl)C2=CC=CC=C21)=O.O=C%10[CH](CC1=CC=CC=C1)N%11.CC[CH](C)[CH](C(O)=O)N%10	1.5_6
C%11(COC1=CC=C(Cl)C2=CC=CC=C21)=O.O=C%10[CH](CC1=CC=CC=C1)N%11.CSCC[CH](C(O)=O)N%10	1.5_7
C%11(COC1=CC=C(Cl)C2=CC=CC=C21)=O.O=C%10[CH](CC1=CC=CC=C1)N%11.OC(C=C1)=CC=C1C[CH](C(O)=O)N%10	1.5_8
C%11(COC1=CC=C(Cl)C2=CC=CC=C21)=O.O=C%10[CH](CC1=CC=CC=C1)N%11.O=C(O)[CH](CC1=C(F)C(F)=C(F)C(F)=C1F)N%10	1.5_9
C%11(COC1=CC=C(Cl)C2=CC=CC=C21)=O.CC[CH](C)[CH](C%10=O)N%11.O=C(O)CN%10	1.6_1
C%11(COC1=CC=C(Cl)C2=CC=CC=C21)=O.CC[CH](C)[CH](C%10=O)N%11.CC(C)[CH](C(O)=O)N%10	1.6_2
C%11(COC1=CC=C(Cl)C2=CC=CC=C21)=O.CC[CH](C)[CH](C%10=O)N%11.CC(C)C[CH](C(O)=O)N%10	1.6_3
C%11(COC1=CC=C(Cl)C2=CC=CC=C21)=O.CC[CH](C)[CH](C%10=O)N%11.C[CH](C(O)=O)N%10	1.6_4
C%11(COC1=CC=C(Cl)C2=CC=CC=C21)=O.CC[CH](C)[CH](C%10=O)N%11.O=C(O)[CH](CC1=CC=CC=C1)N%10	1.6_5
C%11(COC1=CC=C(Cl)C2=CC=CC=C21)=O.CC[CH](C)[CH](C%10=O)N%11.CC[CH](C)[CH](C(O)=O)N%10	1.6_6
C%11(COC1=CC=C(Cl)C2=CC=CC=C21)=O.CC[CH](C)[CH](C%10=O)N%11.CSCC[CH](C(O)=O)N%10	1.6_7
C%11(COC1=CC=C(Cl)C2=CC=CC=C21)=O.CC[CH](C)[CH](C%10=O)N%11.OC(C=C1)=CC=C1C[CH](C(O)=O)N%10	1.6_8
C%11(COC1=CC=C(Cl)C2=CC=CC=C21)=O.CC[CH](C)[CH](C%10=O)N%11.O=C(O)[CH](CC1=C(F)C(F)=C(F)C(F)=C1F)N%10	1.6_9

C%11(COC1=CC=C(Cl)C2=CC=CC=C21)=O.CSCC[CH](C%10=O)N%11.O=C(O)CN%10	1.7_1
C%11(COC1=CC=C(Cl)C2=CC=CC=C21)=O.CSCC[CH](C%10=O)N%11.CC(C)[CH](C(O)=O)N%10	1.7_2
C%11(COC1=CC=C(Cl)C2=CC=CC=C21)=O.CSCC[CH](C%10=O)N%11.CC(C)C[CH](C(O)=O)N%10	1.7_3
C%11(COC1=CC=C(Cl)C2=CC=CC=C21)=O.CSCC[CH](C%10=O)N%11.C[CH](C(O)=O)N%10	1.7_4
C%11(COC1=CC=C(Cl)C2=CC=CC=C21)=O.CSCC[CH](C%10=O)N%11.O=C(O)[CH](CC1=CC=CC=C1)N%10	1.7_5
C%11(COC1=CC=C(Cl)C2=CC=CC=C21)=O.CSCC[CH](C%10=O)N%11.CC[CH](C)[CH](C(O)=O)N%10	1.7_6
C%11(COC1=CC=C(Cl)C2=CC=CC=C21)=O.CSCC[CH](C%10=O)N%11.CSCC[CH](C(O)=O)N%10	1.7_7
C%11(COC1=CC=C(Cl)C2=CC=CC=C21)=O.CSCC[CH](C%10=O)N%11.OC(C=C1)=CC=C1C[CH](C(O)=O)N%10	1.7_8
C%11(COC1=CC=C(Cl)C2=CC=CC=C21)=O.CSCC[CH](C%10=O)N%11.O=C(O)[CH](CC1=C(F)C(F)=C(F)C(F)=C1F)N%10	1.7_9
C%11(COC1=CC=C(Cl)C2=CC=CC=C21)=O.OC(C=C1)=CC=C1C[CH](C%10=O)N%11.O=C(O)CN%10	1.8_1
C%11(COC1=CC=C(Cl)C2=CC=CC=C21)=O.OC(C=C1)=CC=C1C[CH](C%10=O)N%11.CC(C)[CH](C(O)=O)N%10	1.8_2
C%11(COC1=CC=C(Cl)C2=CC=CC=C21)=O.OC(C=C1)=CC=C1C[CH](C%10=O)N%11.CC(C)C[CH](C(O)=O)N%10	1.8_3
C%11(COC1=CC=C(Cl)C2=CC=CC=C21)=O.OC(C=C1)=CC=C1C[CH](C%10=O)N%11.C[CH](C(O)=O)N%10	1.8_4
C%11(COC1=CC=C(Cl)C2=CC=CC=C21)=O.OC(C=C1)=CC=C1C[CH](C%10=O)N%11.O=C(O)[CH](CC1=CC=CC=C1)N%10	1.8_5
C%11(COC1=CC=C(Cl)C2=CC=CC=C21)=O.OC(C=C1)=CC=C1C[CH](C%10=O)N%11.CC[CH](C)[CH](C(O)=O)N%10	1.8_6
C%11(COC1=CC=C(Cl)C2=CC=CC=C21)=O.OC(C=C1)=CC=C1C[CH](C%10=O)N%11.CSCC[CH](C(O)=O)N%10	1.8_7
C%11(COC1=CC=C(Cl)C2=CC=CC=C21)=O.OC(C=C1)=CC=C1C[CH](C%10=O)N%11.OC(C=C1)=CC=C1C[CH](C(O)=O)N%10	1.8_8
C%11(COC1=CC=C(Cl)C2=CC=CC=C21)=O.OC(C=C1)=CC=C1C[CH](C%10=O)N%11.O=C(O)[CH](CC1=C(F)C(F)=C(F)C(F)=C1F)N%10	1.8_9
C%10(COC1=CC=C(Cl)C2=CC=CC=C21)=O.O=C(O)CN%10	1.9_1
C%10(COC1=CC=C(Cl)C2=CC=CC=C21)=O.CC(C)[CH](C(O)=O)N%10	1.9_2
C%10(COC1=CC=C(Cl)C2=CC=CC=C21)=O.CC(C)C[CH](C(O)=O)N%10	1.9_3
C%10(COC1=CC=C(Cl)C2=CC=CC=C21)=O.C[CH](C(O)=O)N%10	1.9_4
C%10(COC1=CC=C(Cl)C2=CC=CC=C21)=O.O=C(O)[CH](CC1=CC=CC=C1)N%10	1.9_5
C%10(COC1=CC=C(Cl)C2=CC=CC=C21)=O.CC[CH](C)[CH](C(O)=O)N%10	1.9_6
C%10(COC1=CC=C(Cl)C2=CC=CC=C21)=O.CSCC[CH](C(O)=O)N%10	1.9_7
C%10(COC1=CC=C(Cl)C2=CC=CC=C21)=O.OC(C=C1)=CC=C1C[CH](C(O)=O)N%10	1.9_8
C%10(COC1=CC=C(Cl)C2=CC=CC=C21)=O.O=C(O)[CH](CC1=C(F)C(F)=C(F)C(F)=C1F)N%10	1.9_9
ClC1=CC(OCC%11=O)=CC2=CC=CC=C21.O=C%10CN%11.O=C(O)CN%10	2.1_1
ClC1=CC(OCC%11=O)=CC2=CC=CC=C21.O=C%10CN%11.CC(C)[CH](C(O)=O)N%10	2.1_2
ClC1=CC(OCC%11=O)=CC2=CC=CC=C21.O=C%10CN%11.CC(C)C[CH](C(O)=O)N%10	2.1_3
ClC1=CC(OCC%11=O)=CC2=CC=CC=C21.O=C%10CN%11.C[CH](C(O)=O)N%10	2.1_4

ClC1=CC(OCC%11=O)=CC2=CC=CC=C21.O=C%10CN%11.O=C(O)[CH](CC1=CC=CC=C1)N%10	2.1_5
ClC1=CC(OCC%11=O)=CC2=CC=CC=C21.O=C%10CN%11.CC[CH](C)[CH](C(O)=O)N%10	2.1_6
ClC1=CC(OCC%11=O)=CC2=CC=CC=C21.O=C%10CN%11.CSCC[CH](C(O)=O)N%10	2.1_7
ClC1=CC(OCC%11=O)=CC2=CC=CC=C21.O=C%10CN%11.OC(C=C1)=CC=C1C[CH](C(O)=O)N%10	2.1_8
ClC1=CC(OCC%11=O)=CC2=CC=CC=C21.O=C%10CN%11.O=C(O)[CH](CC1=C(F)C(F)=C(F)C(F)=C1F)N%10	2.1_9
ClC1=CC(OCC%11=O)=CC2=CC=CC=C21.CC(C)[CH](C%10=O)N%11.O=C(O)CN%10	2.2_1
ClC1=CC(OCC%11=O)=CC2=CC=CC=C21.CC(C)[CH](C%10=O)N%11.CC(C)[CH](C(O)=O)N%10	2.2_2
ClC1=CC(OCC%11=O)=CC2=CC=CC=C21.CC(C)[CH](C%10=O)N%11.CC(C)C[CH](C(O)=O)N%10	2.2_3
ClC1=CC(OCC%11=O)=CC2=CC=CC=C21.CC(C)[CH](C%10=O)N%11.C[CH](C(O)=O)N%10	2.2_4
ClC1=CC(OCC%11=O)=CC2=CC=CC=C21.CC(C)[CH](C%10=O)N%11.O=C(O)[CH](CC1=CC=CC=C1)N%10	2.2_5
ClC1=CC(OCC%11=O)=CC2=CC=CC=C21.CC(C)[CH](C%10=O)N%11.CC[CH](C)[CH](C(O)=O)N%10	2.2_6
ClC1=CC(OCC%11=O)=CC2=CC=CC=C21.CC(C)[CH](C%10=O)N%11.CSCC[CH](C(O)=O)N%10	2.2_7
ClC1=CC(OCC%11=O)=CC2=CC=CC=C21.CC(C)[CH](C%10=O)N%11.OC(C=C1)=CC=C1C[CH](C(O)=O)N%10	2.2_8
ClC1=CC(OCC%11=O)=CC2=CC=CC=C21.CC(C)[CH](C%10=O)N%11.O=C(O)[CH](CC1=C(F)C(F)=C(F)C(F)=C1F)N%10	2.2_9
ClC1=CC(OCC%11=O)=CC2=CC=CC=C21.CC(C)C[CH](C%10=O)N%11.O=C(O)CN%10	2.3_1
ClC1=CC(OCC%11=O)=CC2=CC=CC=C21.CC(C)C[CH](C%10=O)N%11.CC(C)[CH](C(O)=O)N%10	2.3_2
ClC1=CC(OCC%11=O)=CC2=CC=CC=C21.CC(C)C[CH](C%10=O)N%11.CC(C)C[CH](C(O)=O)N%10	2.3_3
ClC1=CC(OCC%11=O)=CC2=CC=CC=C21.CC(C)C[CH](C%10=O)N%11.C[CH](C(O)=O)N%10	2.3_4
ClC1=CC(OCC%11=O)=CC2=CC=CC=C21.CC(C)C[CH](C%10=O)N%11.O=C(O)[CH](CC1=CC=CC=C1)N%10	2.3_5
ClC1=CC(OCC%11=O)=CC2=CC=CC=C21.CC(C)C[CH](C%10=O)N%11.CC[CH](C)[CH](C(O)=O)N%10	2.3_6
ClC1=CC(OCC%11=O)=CC2=CC=CC=C21.CC(C)C[CH](C%10=O)N%11.CSCC[CH](C(O)=O)N%10	2.3_7
ClC1=CC(OCC%11=O)=CC2=CC=CC=C21.CC(C)C[CH](C%10=O)N%11.OC(C=C1)=CC=C1C[CH](C(O)=O)N%10	2.3_8
ClC1=CC(OCC%11=O)=CC2=CC=CC=C21.CC(C)C[CH](C%10=O)N%11.O=C(O)[CH](CC1=C(F)C(F)=C(F)C(F)=C1F)N%10	2.3_9
ClC1=CC(OCC%11=O)=CC2=CC=CC=C21.C[CH](C%10=O)N%11.O=C(O)CN%10	2.4_1
ClC1=CC(OCC%11=O)=CC2=CC=CC=C21.C[CH](C%10=O)N%11.CC(C)[CH](C(O)=O)N%10	2.4_2
ClC1=CC(OCC%11=O)=CC2=CC=CC=C21.C[CH](C%10=O)N%11.CC(C)C[CH](C(O)=O)N%10	2.4_3
ClC1=CC(OCC%11=O)=CC2=CC=CC=C21.C[CH](C%10=O)N%11.C[CH](C(O)=O)N%10	2.4_4
ClC1=CC(OCC%11=O)=CC2=CC=CC=C21.C[CH](C%10=O)N%11.O=C(O)[CH](CC1=CC=CC=C1)N%10	2.4_5
ClC1=CC(OCC%11=O)=CC2=CC=CC=C21.C[CH](C%10=O)N%11.CC[CH](C)[CH](C(O)=O)N%10	2.4_6

ClC1=CC(OCC%11=O)=CC2=CC=CC=C21.C[CH](C%10=O)N%11.CSCC[CH](C(O)=O)N%10	2.4_7
ClC1=CC(OCC%11=O)=CC2=CC=CC=C21.C[CH](C%10=O)N%11.OC(C=C1)=CC=C1C[CH](C(O)=O)N%10	2.4_8
ClC1=CC(OCC%11=O)=CC2=CC=CC=C21.C[CH](C%10=O)N%11.O=C(O)[CH](CC1=C(F)C(F)=C(F)C(F)=C1F)N%10	2.4_9
ClC1=CC(OCC%11=O)=CC2=CC=CC=C21.O=C%10[CH](CC1=CC=CC=C1)N%11.O=C(O)CN%10	2.5_1
ClC1=CC(OCC%11=O)=CC2=CC=CC=C21.O=C%10[CH](CC1=CC=CC=C1)N%11.CC(C)[CH](C(O)=O)N%10	2.5_2
ClC1=CC(OCC%11=O)=CC2=CC=CC=C21.O=C%10[CH](CC1=CC=CC=C1)N%11.CC(C)C[CH](C(O)=O)N%10	2.5_3
ClC1=CC(OCC%11=O)=CC2=CC=CC=C21.O=C%10[CH](CC1=CC=CC=C1)N%11.C[CH](C(O)=O)N%10	2.5_4
ClC1=CC(OCC%11=O)=CC2=CC=CC=C21.O=C%10[CH](CC1=CC=CC=C1)N%11.O=C(O)[CH](CC1=CC=CC=C1)N%10	2.5_5
ClC1=CC(OCC%11=O)=CC2=CC=CC=C21.O=C%10[CH](CC1=CC=CC=C1)N%11.CC[CH](C)[CH](C(O)=O)N%10	2.5_6
ClC1=CC(OCC%11=O)=CC2=CC=CC=C21.O=C%10[CH](CC1=CC=CC=C1)N%11.CSCC[CH](C(O)=O)N%10	2.5_7
ClC1=CC(OCC%11=O)=CC2=CC=CC=C21.O=C%10[CH](CC1=CC=CC=C1)N%11.OC(C=C1)=CC=C1C[CH](C(O)=O)N%10	2.5_8
ClC1=CC(OCC%11=O)=CC2=CC=CC=C21.O=C%10[CH](CC1=CC=CC=C1)N%11.O=C(O)[CH](CC1=C(F)C(F)=C(F)C(F)=C1F)N%10	2.5_9
ClC1=CC(OCC%11=O)=CC2=CC=CC=C21.CC[CH](C)[CH](C%10=O)N%11.O=C(O)CN%10	2.6_1
ClC1=CC(OCC%11=O)=CC2=CC=CC=C21.CC[CH](C)[CH](C%10=O)N%11.CC(C)[CH](C(O)=O)N%10	2.6_2
ClC1=CC(OCC%11=O)=CC2=CC=CC=C21.CC[CH](C)[CH](C%10=O)N%11.CC(C)C[CH](C(O)=O)N%10	2.6_3
ClC1=CC(OCC%11=O)=CC2=CC=CC=C21.CC[CH](C)[CH](C%10=O)N%11.C[CH](C(O)=O)N%10	2.6_4
ClC1=CC(OCC%11=O)=CC2=CC=CC=C21.CC[CH](C)[CH](C%10=O)N%11.O=C(O)[CH](CC1=CC=CC=C1)N%10	2.6_5
ClC1=CC(OCC%11=O)=CC2=CC=CC=C21.CC[CH](C)[CH](C%10=O)N%11.CC[CH](C)[CH](C(O)=O)N%10	2.6_6
ClC1=CC(OCC%11=O)=CC2=CC=CC=C21.CC[CH](C)[CH](C%10=O)N%11.CSCC[CH](C(O)=O)N%10	2.6_7
ClC1=CC(OCC%11=O)=CC2=CC=CC=C21.CC[CH](C)[CH](C%10=O)N%11.OC(C=C1)=CC=C1C[CH](C(O)=O)N%10	2.6_8
ClC1=CC(OCC%11=O)=CC2=CC=CC=C21.CC[CH](C)[CH](C%10=O)N%11.O=C(O)[CH](CC1=C(F)C(F)=C(F)C(F)=C1F)N%10	2.6_9
ClC1=CC(OCC%11=O)=CC2=CC=CC=C21.CSCC[CH](C%10=O)N%11.O=C(O)CN%10	2.7_1
ClC1=CC(OCC%11=O)=CC2=CC=CC=C21.CSCC[CH](C%10=O)N%11.CC(C)[CH](C(O)=O)N%10	2.7_2
ClC1=CC(OCC%11=O)=CC2=CC=CC=C21.CSCC[CH](C%10=O)N%11.CC(C)C[CH](C(O)=O)N%10	2.7_3
ClC1=CC(OCC%11=O)=CC2=CC=CC=C21.CSCC[CH](C%10=O)N%11.C[CH](C(O)=O)N%10	2.7_4
ClC1=CC(OCC%11=O)=CC2=CC=CC=C21.CSCC[CH](C%10=O)N%11.O=C(O)[CH](CC1=CC=CC=C1)N%10	2.7_5
ClC1=CC(OCC%11=O)=CC2=CC=CC=C21.CSCC[CH](C%10=O)N%11.CC[CH](C)[CH](C(O)=O)	2.7_6

N%10	
ClC1=CC(OCC%11=O)=CC2=CC=CC=C21.CSCC[CH](C%10=O)N%11.CSCC[CH](C(O)=O)N%10	2.7_7
ClC1=CC(OCC%11=O)=CC2=CC=CC=C21.CSCC[CH](C%10=O)N%11.OC(C=C1)=CC=C1C[CH](C(O)=O)N%10	2.7_8
ClC1=CC(OCC%11=O)=CC2=CC=CC=C21.CSCC[CH](C%10=O)N%11.O=C(O)[CH](CC1=C(F)C(F)=C(F)C(F)=C1F)N%10	2.7_9
ClC1=CC(OCC%11=O)=CC2=CC=CC=C21.OC(C=C1)=CC=C1C[CH](C%10=O)N%11.O=C(O)CN%10	2.8_1
ClC1=CC(OCC%11=O)=CC2=CC=CC=C21.OC(C=C1)=CC=C1C[CH](C%10=O)N%11.CC(C)[CH](C(O)=O)N%10	2.8_2
ClC1=CC(OCC%11=O)=CC2=CC=CC=C21.OC(C=C1)=CC=C1C[CH](C%10=O)N%11.CC(C)C[CH](C(O)=O)N%10	2.8_3
ClC1=CC(OCC%11=O)=CC2=CC=CC=C21.OC(C=C1)=CC=C1C[CH](C%10=O)N%11.C[CH](C(O)=O)N%10	2.8_4
ClC1=CC(OCC%11=O)=CC2=CC=CC=C21.OC(C=C1)=CC=C1C[CH](C%10=O)N%11.O=C(O)[CH](CC1=CC=CC=C1)N%10	2.8_5
ClC1=CC(OCC%11=O)=CC2=CC=CC=C21.OC(C=C1)=CC=C1C[CH](C%10=O)N%11.CC[CH](C)[CH](C(O)=O)N%10	2.8_6
ClC1=CC(OCC%11=O)=CC2=CC=CC=C21.OC(C=C1)=CC=C1C[CH](C%10=O)N%11.CSCC[CH](C(O)=O)N%10	2.8_7
ClC1=CC(OCC%11=O)=CC2=CC=CC=C21.OC(C=C1)=CC=C1C[CH](C%10=O)N%11.OC(C=C1)=CC=C1C[CH](C(O)=O)N%10	2.8_8
ClC1=CC(OCC%11=O)=CC2=CC=CC=C21.OC(C=C1)=CC=C1C[CH](C%10=O)N%11.O=C(O)[CH](CC1=C(F)C(F)=C(F)C(F)=C1F)N%10	2.8_9
ClC1=CC(OCC%10=O)=CC2=CC=CC=C21.O=C(O)CN%10	2.9_1
ClC1=CC(OCC%10=O)=CC2=CC=CC=C21.CC(C)[CH](C(O)=O)N%10	2.9_2
ClC1=CC(OCC%10=O)=CC2=CC=CC=C21.CC(C)C[CH](C(O)=O)N%10	2.9_3
ClC1=CC(OCC%10=O)=CC2=CC=CC=C21.C[CH](C(O)=O)N%10	2.9_4
ClC1=CC(OCC%10=O)=CC2=CC=CC=C21.O=C(O)[CH](CC1=CC=CC=C1)N%10	2.9_5
ClC1=CC(OCC%10=O)=CC2=CC=CC=C21.CC[CH](C)[CH](C(O)=O)N%10	2.9_6
ClC1=CC(OCC%10=O)=CC2=CC=CC=C21.CSCC[CH](C(O)=O)N%10	2.9_7
ClC1=CC(OCC%10=O)=CC2=CC=CC=C21.OC(C=C1)=CC=C1C[CH](C(O)=O)N%10	2.9_8
ClC1=CC(OCC%10=O)=CC2=CC=CC=C21.O=C(O)[CH](CC1=C(F)C(F)=C(F)C(F)=C1F)N%10	2.9_9
C%11(COC1=CC=CC2=CC=CC=C21)=O.O=C%10CN%11.O=C(O)CN%10	3.1_1
C%11(COC1=CC=CC2=CC=CC=C21)=O.O=C%10CN%11.CC(C)[CH](C(O)=O)N%10	3.1_2
C%11(COC1=CC=CC2=CC=CC=C21)=O.O=C%10CN%11.CC(C)C[CH](C(O)=O)N%10	3.1_3
C%11(COC1=CC=CC2=CC=CC=C21)=O.O=C%10CN%11.C[CH](C(O)=O)N%10	3.1_4
C%11(COC1=CC=CC2=CC=CC=C21)=O.O=C%10CN%11.O=C(O)[CH](CC1=CC=CC=C1)N%10	3.1_5
C%11(COC1=CC=CC2=CC=CC=C21)=O.O=C%10CN%11.CC[CH](C)[CH](C(O)=O)N%10	3.1_6
C%11(COC1=CC=CC2=CC=CC=C21)=O.O=C%10CN%11.CSCC[CH](C(O)=O)N%10	3.1_7
C%11(COC1=CC=CC2=CC=CC=C21)=O.O=C%10CN%11.OC(C=C1)=CC=C1C[CH](C(O)=O)N%10	3.1_8
C%11(COC1=CC=CC2=CC=CC=C21)=O.O=C%10CN%11.O=C(O)[CH](CC1=C(F)C(F)=C(F)C(F)=C1F)N%10	3.1_9
C%11(COC1=CC=CC2=CC=CC=C21)=O.CC(C)[CH](C%10=O)N%11.O=C(O)CN%10	3.2_1
C%11(COC1=CC=CC2=CC=CC=C21)=O.CC(C)[CH](C%10=O)N%11.CC(C)[CH](C(O)=O)N%10	3.2_2

C%11(COC1=CC=CC2=CC=CC=C21)=O.CC(C)[CH](C%10=O)N%11.CC(C)C[CH](C(O)=O)N%10	3.2_3
C%11(COC1=CC=CC2=CC=CC=C21)=O.CC(C)[CH](C%10=O)N%11.C[CH](C(O)=O)N%10	3.2_4
C%11(COC1=CC=CC2=CC=CC=C21)=O.CC(C)[CH](C%10=O)N%11.O=C(O)[CH](CC1=CC=CC=C1)N%10	3.2_5
C%11(COC1=CC=CC2=CC=CC=C21)=O.CC(C)[CH](C%10=O)N%11.CC[CH](C)[CH](C(O)=O)N%10	3.2_6
C%11(COC1=CC=CC2=CC=CC=C21)=O.CC(C)[CH](C%10=O)N%11.CSCC[CH](C(O)=O)N%10	3.2_7
C%11(COC1=CC=CC2=CC=CC=C21)=O.CC(C)[CH](C%10=O)N%11.OC(C=C1)=CC=C1C[CH](C(O)=O)N%10	3.2_8
C%11(COC1=CC=CC2=CC=CC=C21)=O.CC(C)[CH](C%10=O)N%11.O=C(O)[CH](CC1=C(F)C(F)=C(F)C(F)=C1F)N%10	3.2_9
C%11(COC1=CC=CC2=CC=CC=C21)=O.CC(C)C[CH](C%10=O)N%11.O=C(O)CN%10	3.3_1
C%11(COC1=CC=CC2=CC=CC=C21)=O.CC(C)C[CH](C%10=O)N%11.CC(C)[CH](C(O)=O)N%10	3.3_2
C%11(COC1=CC=CC2=CC=CC=C21)=O.CC(C)C[CH](C%10=O)N%11.CC(C)C[CH](C(O)=O)N%10	3.3_3
C%11(COC1=CC=CC2=CC=CC=C21)=O.CC(C)C[CH](C%10=O)N%11.C[CH](C(O)=O)N%10	3.3_4
C%11(COC1=CC=CC2=CC=CC=C21)=O.CC(C)C[CH](C%10=O)N%11.O=C(O)[CH](CC1=CC=CC=C1)N%10	3.3_5
C%11(COC1=CC=CC2=CC=CC=C21)=O.CC(C)C[CH](C%10=O)N%11.CC[CH](C)[CH](C(O)=O)N%10	3.3_6
C%11(COC1=CC=CC2=CC=CC=C21)=O.CC(C)C[CH](C%10=O)N%11.CSCC[CH](C(O)=O)N%10	3.3_7
C%11(COC1=CC=CC2=CC=CC=C21)=O.CC(C)C[CH](C%10=O)N%11.OC(C=C1)=CC=C1C[CH](C(O)=O)N%10	3.3_8
C%11(COC1=CC=CC2=CC=CC=C21)=O.CC(C)C[CH](C%10=O)N%11.O=C(O)[CH](CC1=C(F)C(F)=C(F)C(F)=C1F)N%10	3.3_9
C%11(COC1=CC=CC2=CC=CC=C21)=O.C[CH](C%10=O)N%11.O=C(O)CN%10	3.4_1
C%11(COC1=CC=CC2=CC=CC=C21)=O.C[CH](C%10=O)N%11.CC(C)[CH](C(O)=O)N%10	3.4_2
C%11(COC1=CC=CC2=CC=CC=C21)=O.C[CH](C%10=O)N%11.CC(C)C[CH](C(O)=O)N%10	3.4_3
C%11(COC1=CC=CC2=CC=CC=C21)=O.C[CH](C%10=O)N%11.C[CH](C(O)=O)N%10	3.4_4
C%11(COC1=CC=CC2=CC=CC=C21)=O.C[CH](C%10=O)N%11.O=C(O)[CH](CC1=CC=CC=C1)N%10	3.4_5
C%11(COC1=CC=CC2=CC=CC=C21)=O.C[CH](C%10=O)N%11.CC[CH](C)[CH](C(O)=O)N%10	3.4_6
C%11(COC1=CC=CC2=CC=CC=C21)=O.C[CH](C%10=O)N%11.CSCC[CH](C(O)=O)N%10	3.4_7
C%11(COC1=CC=CC2=CC=CC=C21)=O.C[CH](C%10=O)N%11.OC(C=C1)=CC=C1C[CH](C(O)=O)N%10	3.4_8
C%11(COC1=CC=CC2=CC=CC=C21)=O.C[CH](C%10=O)N%11.O=C(O)[CH](CC1=C(F)C(F)=C(F)C(F)=C1F)N%10	3.4_9
C%11(COC1=CC=CC2=CC=CC=C21)=O.O=C%10[CH](CC1=CC=CC=C1)N%11.O=C(O)CN%10	3.5_1
C%11(COC1=CC=CC2=CC=CC=C21)=O.O=C%10[CH](CC1=CC=CC=C1)N%11.CC(C)[CH](C(O)=O)N%10	3.5_2
C%11(COC1=CC=CC2=CC=CC=C21)=O.O=C%10[CH](CC1=CC=CC=C1)N%11.CC(C)C[CH](C(O)=O)N%10	3.5_3
C%11(COC1=CC=CC2=CC=CC=C21)=O.O=C%10[CH](CC1=CC=CC=C1)N%11.C[CH](C(O)=O)N%10	3.5_4
C%11(COC1=CC=CC2=CC=CC=C21)=O.O=C%10[CH](CC1=CC=CC=C1)N%11.O=C(O)[CH](CC	3.5_5

1=CC=CC=C1)N%10	
C%11(COC1=CC=CC2=CC=CC=C21)=O.O=C%10[CH](CC1=CC=CC=C1)N%11.CC[CH](C)[CH](C(O)=O)N%10	3.5_6
C%11(COC1=CC=CC2=CC=CC=C21)=O.O=C%10[CH](CC1=CC=CC=C1)N%11.CSCC[CH](C(O)=O)N%10	3.5_7
C%11(COC1=CC=CC2=CC=CC=C21)=O.O=C%10[CH](CC1=CC=CC=C1)N%11.OC(C=C1)=CC=C1C[CH](C(O)=O)N%10	3.5_8
C%11(COC1=CC=CC2=CC=CC=C21)=O.O=C%10[CH](CC1=CC=CC=C1)N%11.O=C(O)[CH](CC1=C(F)C(F)=C(F)C(F)=C1F)N%10	3.5_9
C%11(COC1=CC=CC2=CC=CC=C21)=O.CC[CH](C)[CH](C%10=O)N%11.O=C(O)CN%10	3.6_1
C%11(COC1=CC=CC2=CC=CC=C21)=O.CC[CH](C)[CH](C%10=O)N%11.CC(C)[CH](C(O)=O)N%10	3.6_2
C%11(COC1=CC=CC2=CC=CC=C21)=O.CC[CH](C)[CH](C%10=O)N%11.CC(C)C[CH](C(O)=O)N%10	3.6_3
C%11(COC1=CC=CC2=CC=CC=C21)=O.CC[CH](C)[CH](C%10=O)N%11.C[CH](C(O)=O)N%10	3.6_4
C%11(COC1=CC=CC2=CC=CC=C21)=O.CC[CH](C)[CH](C%10=O)N%11.O=C(O)[CH](CC1=CC=CC=C1)N%10	3.6_5
C%11(COC1=CC=CC2=CC=CC=C21)=O.CC[CH](C)[CH](C%10=O)N%11.CC[CH](C)[CH](C(O)=O)N%10	3.6_6
C%11(COC1=CC=CC2=CC=CC=C21)=O.CC[CH](C)[CH](C%10=O)N%11.CSCC[CH](C(O)=O)N%10	3.6_7
C%11(COC1=CC=CC2=CC=CC=C21)=O.CC[CH](C)[CH](C%10=O)N%11.OC(C=C1)=CC=C1C[CH](C(O)=O)N%10	3.6_8
C%11(COC1=CC=CC2=CC=CC=C21)=O.CC[CH](C)[CH](C%10=O)N%11.O=C(O)[CH](CC1=C(F)C(F)=C(F)C(F)=C1F)N%10	3.6_9
C%11(COC1=CC=CC2=CC=CC=C21)=O.CSCC[CH](C%10=O)N%11.O=C(O)CN%10	3.7_1
C%11(COC1=CC=CC2=CC=CC=C21)=O.CSCC[CH](C%10=O)N%11.CC(C)[CH](C(O)=O)N%10	3.7_2
C%11(COC1=CC=CC2=CC=CC=C21)=O.CSCC[CH](C%10=O)N%11.CC(C)C[CH](C(O)=O)N%10	3.7_3
C%11(COC1=CC=CC2=CC=CC=C21)=O.CSCC[CH](C%10=O)N%11.C[CH](C(O)=O)N%10	3.7_4
C%11(COC1=CC=CC2=CC=CC=C21)=O.CSCC[CH](C%10=O)N%11.O=C(O)[CH](CC1=CC=CC=C1)N%10	3.7_5
C%11(COC1=CC=CC2=CC=CC=C21)=O.CSCC[CH](C%10=O)N%11.CC[CH](C)[CH](C(O)=O)N%10	3.7_6
C%11(COC1=CC=CC2=CC=CC=C21)=O.CSCC[CH](C%10=O)N%11.CSCC[CH](C(O)=O)N%10	3.7_7
C%11(COC1=CC=CC2=CC=CC=C21)=O.CSCC[CH](C%10=O)N%11.OC(C=C1)=CC=C1C[CH](C(O)=O)N%10	3.7_8
C%11(COC1=CC=CC2=CC=CC=C21)=O.CSCC[CH](C%10=O)N%11.O=C(O)[CH](CC1=C(F)C(F)=C(F)C(F)=C1F)N%10	3.7_9
C%11(COC1=CC=CC2=CC=CC=C21)=O.OC(C=C1)=CC=C1C[CH](C%10=O)N%11.O=C(O)CN%10	3.8_1
C%11(COC1=CC=CC2=CC=CC=C21)=O.OC(C=C1)=CC=C1C[CH](C%10=O)N%11.CC(C)[CH](C(O)=O)N%10	3.8_2
C%11(COC1=CC=CC2=CC=CC=C21)=O.OC(C=C1)=CC=C1C[CH](C%10=O)N%11.CC(C)C[CH](C(O)=O)N%10	3.8_3
C%11(COC1=CC=CC2=CC=CC=C21)=O.OC(C=C1)=CC=C1C[CH](C%10=O)N%11.C[CH](C(O)=O)N%10	3.8_4
C%11(COC1=CC=CC2=CC=CC=C21)=O.OC(C=C1)=CC=C1C[CH](C%10=O)N%11.O=C(O)[CH]	3.8_5

(CC1=CC=CC=C1)N%10	
C%11(COC1=CC=CC2=CC=CC=C21)=O.OC(C=C1)=CC=C1C[CH](C%10=O)N%11.CC[CH](C)[CH](C(O)=O)N%10	3.8_6
C%11(COC1=CC=CC2=CC=CC=C21)=O.OC(C=C1)=CC=C1C[CH](C%10=O)N%11.CSCC[CH](C(O)=O)N%10	3.8_7
C%11(COC1=CC=CC2=CC=CC=C21)=O.OC(C=C1)=CC=C1C[CH](C%10=O)N%11.OC(C=C1)=CC=C1C[CH](C(O)=O)N%10	3.8_8
C%11(COC1=CC=CC2=CC=CC=C21)=O.OC(C=C1)=CC=C1C[CH](C%10=O)N%11.O=C(O)[CH](CC1=C(F)C(F)=C(F)C(F)=C1F)N%10	3.8_9
C%10(COC1=CC=CC2=CC=CC=C21)=O.O=C(O)CN%10	3.9_1
C%10(COC1=CC=CC2=CC=CC=C21)=O.CC(C)[CH](C(O)=O)N%10	3.9_2
C%10(COC1=CC=CC2=CC=CC=C21)=O.CC(C)C[CH](C(O)=O)N%10	3.9_3
C%10(COC1=CC=CC2=CC=CC=C21)=O.C[CH](C(O)=O)N%10	3.9_4
C%10(COC1=CC=CC2=CC=CC=C21)=O.O=C(O)[CH](CC1=CC=CC=C1)N%10	3.9_5
C%10(COC1=CC=CC2=CC=CC=C21)=O.CC[CH](C)[CH](C(O)=O)N%10	3.9_6
C%10(COC1=CC=CC2=CC=CC=C21)=O.CSCC[CH](C(O)=O)N%10	3.9_7
C%10(COC1=CC=CC2=CC=CC=C21)=O.OC(C=C1)=CC=C1C[CH](C(O)=O)N%10	3.9_8
C%10(COC1=CC=CC2=CC=CC=C21)=O.O=C(O)[CH](CC1=C(F)C(F)=C(F)C(F)=C1F)N%10	3.9_9
C%11(COC1=CC2=CC=CC=C2C=C1)=O.O=C%10CN%11.O=C(O)CN%10	4.1_1
C%11(COC1=CC2=CC=CC=C2C=C1)=O.O=C%10CN%11.CC(C)[CH](C(O)=O)N%10	4.1_2
C%11(COC1=CC2=CC=CC=C2C=C1)=O.O=C%10CN%11.CC(C)C[CH](C(O)=O)N%10	4.1_3
C%11(COC1=CC2=CC=CC=C2C=C1)=O.O=C%10CN%11.C[CH](C(O)=O)N%10	4.1_4
C%11(COC1=CC2=CC=CC=C2C=C1)=O.O=C%10CN%11.O=C(O)[CH](CC1=CC=CC=C1)N%10	4.1_5
C%11(COC1=CC2=CC=CC=C2C=C1)=O.O=C%10CN%11.CC[CH](C)[CH](C(O)=O)N%10	4.1_6
C%11(COC1=CC2=CC=CC=C2C=C1)=O.O=C%10CN%11.CSCC[CH](C(O)=O)N%10	4.1_7
C%11(COC1=CC2=CC=CC=C2C=C1)=O.O=C%10CN%11.OC(C=C1)=CC=C1C[CH](C(O)=O)N%10	4.1_8
C%11(COC1=CC2=CC=CC=C2C=C1)=O.O=C%10CN%11.O=C(O)[CH](CC1=C(F)C(F)=C(F)C(F)=C1F)N%10	4.1_9
C%11(COC1=CC2=CC=CC=C2C=C1)=O.CC(C)[CH](C%10=O)N%11.O=C(O)CN%10	4.2_1
C%11(COC1=CC2=CC=CC=C2C=C1)=O.CC(C)[CH](C%10=O)N%11.CC(C)[CH](C(O)=O)N%10	4.2_2
C%11(COC1=CC2=CC=CC=C2C=C1)=O.CC(C)[CH](C%10=O)N%11.CC(C)C[CH](C(O)=O)N%10	4.2_3
C%11(COC1=CC2=CC=CC=C2C=C1)=O.CC(C)[CH](C%10=O)N%11.C[CH](C(O)=O)N%10	4.2_4
C%11(COC1=CC2=CC=CC=C2C=C1)=O.CC(C)[CH](C%10=O)N%11.O=C(O)[CH](CC1=CC=CC=C1)N%10	4.2_5
C%11(COC1=CC2=CC=CC=C2C=C1)=O.CC(C)[CH](C%10=O)N%11.CC[CH](C)[CH](C(O)=O)N%10	4.2_6
C%11(COC1=CC2=CC=CC=C2C=C1)=O.CC(C)[CH](C%10=O)N%11.CSCC[CH](C(O)=O)N%10	4.2_7
C%11(COC1=CC2=CC=CC=C2C=C1)=O.CC(C)[CH](C%10=O)N%11.OC(C=C1)=CC=C1C[CH](C(O)=O)N%10	4.2_8
C%11(COC1=CC2=CC=CC=C2C=C1)=O.CC(C)[CH](C%10=O)N%11.O=C(O)[CH](CC1=C(F)C(F)=C(F)C(F)=C1F)N%10	4.2_9
C%11(COC1=CC2=CC=CC=C2C=C1)=O.CC(C)C[CH](C%10=O)N%11.O=C(O)CN%10	4.3_1
C%11(COC1=CC2=CC=CC=C2C=C1)=O.CC(C)C[CH](C%10=O)N%11.CC(C)[CH](C(O)=O)N%10	4.3_2

C%11(COC1=CC2=CC=CC=C2C=C1)=O.CC(C)C[CH](C%10=O)N%11.CC(C)C[CH](C(O)=O)N%10	4.3_3
C%11(COC1=CC2=CC=CC=C2C=C1)=O.CC(C)C[CH](C%10=O)N%11.C[CH](C(O)=O)N%10	4.3_4
C%11(COC1=CC2=CC=CC=C2C=C1)=O.CC(C)C[CH](C%10=O)N%11.O=C(O)[CH](CC1=CC=CC=C1)N%10	4.3_5
C%11(COC1=CC2=CC=CC=C2C=C1)=O.CC(C)C[CH](C%10=O)N%11.CC[CH](C)[CH](C(O)=O)N%10	4.3_6
C%11(COC1=CC2=CC=CC=C2C=C1)=O.CC(C)C[CH](C%10=O)N%11.CSCC[CH](C(O)=O)N%10	4.3_7
C%11(COC1=CC2=CC=CC=C2C=C1)=O.CC(C)C[CH](C%10=O)N%11.OC(C=C1)=CC=C1C[CH](C(O)=O)N%10	4.3_8
C%11(COC1=CC2=CC=CC=C2C=C1)=O.CC(C)C[CH](C%10=O)N%11.O=C(O)[CH](CC1=C(F)C(F)=C(F)C(F)=C1F)N%10	4.3_9
C%11(COC1=CC2=CC=CC=C2C=C1)=O.C[CH](C%10=O)N%11.O=C(O)CN%10	4.4_1
C%11(COC1=CC2=CC=CC=C2C=C1)=O.C[CH](C%10=O)N%11.CC(C)[CH](C(O)=O)N%10	4.4_2
C%11(COC1=CC2=CC=CC=C2C=C1)=O.C[CH](C%10=O)N%11.CC(C)C[CH](C(O)=O)N%10	4.4_3
C%11(COC1=CC2=CC=CC=C2C=C1)=O.C[CH](C%10=O)N%11.C[CH](C(O)=O)N%10	4.4_4
C%11(COC1=CC2=CC=CC=C2C=C1)=O.C[CH](C%10=O)N%11.O=C(O)[CH](CC1=CC=CC=C1)N%10	4.4_5
C%11(COC1=CC2=CC=CC=C2C=C1)=O.C[CH](C%10=O)N%11.CC[CH](C)[CH](C(O)=O)N%10	4.4_6
C%11(COC1=CC2=CC=CC=C2C=C1)=O.C[CH](C%10=O)N%11.CSCC[CH](C(O)=O)N%10	4.4_7
C%11(COC1=CC2=CC=CC=C2C=C1)=O.C[CH](C%10=O)N%11.OC(C=C1)=CC=C1C[CH](C(O)=O)N%10	4.4_8
C%11(COC1=CC2=CC=CC=C2C=C1)=O.C[CH](C%10=O)N%11.O=C(O)[CH](CC1=C(F)C(F)=C(F)C(F)=C1F)N%10	4.4_9
C%11(COC1=CC2=CC=CC=C2C=C1)=O.O=C%10[CH](CC1=CC=CC=C1)N%11.O=C(O)CN%10	4.5_1
C%11(COC1=CC2=CC=CC=C2C=C1)=O.O=C%10[CH](CC1=CC=CC=C1)N%11.CC(C)[CH](C(O)=O)N%10	4.5_2
C%11(COC1=CC2=CC=CC=C2C=C1)=O.O=C%10[CH](CC1=CC=CC=C1)N%11.CC(C)C[CH](C(O)=O)N%10	4.5_3
C%11(COC1=CC2=CC=CC=C2C=C1)=O.O=C%10[CH](CC1=CC=CC=C1)N%11.C[CH](C(O)=O)N%10	4.5_4
C%11(COC1=CC2=CC=CC=C2C=C1)=O.O=C%10[CH](CC1=CC=CC=C1)N%11.O=C(O)[CH](CC1=CC=CC=C1)N%10	4.5_5
C%11(COC1=CC2=CC=CC=C2C=C1)=O.O=C%10[CH](CC1=CC=CC=C1)N%11.CC[CH](C)[CH](C(O)=O)N%10	4.5_6
C%11(COC1=CC2=CC=CC=C2C=C1)=O.O=C%10[CH](CC1=CC=CC=C1)N%11.CSCC[CH](C(O)=O)N%10	4.5_7
C%11(COC1=CC2=CC=CC=C2C=C1)=O.O=C%10[CH](CC1=CC=CC=C1)N%11.OC(C=C1)=CC=C1C[CH](C(O)=O)N%10	4.5_8
C%11(COC1=CC2=CC=CC=C2C=C1)=O.O=C%10[CH](CC1=CC=CC=C1)N%11.O=C(O)[CH](CC1=C(F)C(F)=C(F)C(F)=C1F)N%10	4.5_9
C%11(COC1=CC2=CC=CC=C2C=C1)=O.CC[CH](C)[CH](C%10=O)N%11.O=C(O)CN%10	4.6_1
C%11(COC1=CC2=CC=CC=C2C=C1)=O.CC[CH](C)[CH](C%10=O)N%11.CC(C)[CH](C(O)=O)N%10	4.6_2
C%11(COC1=CC2=CC=CC=C2C=C1)=O.CC[CH](C)[CH](C%10=O)N%11.CC(C)C[CH](C(O)=O)N%10	4.6_3
C%11(COC1=CC2=CC=CC=C2C=C1)=O.CC[CH](C)[CH](C%10=O)N%11.C[CH](C(O)=O)N%10	4.6_4

C%11(COC1=CC2=CC=CC=C2C=C1)=O.CC[CH](C)[CH](C%10=O)N%11.O=C(O)[CH](CC1=CC=CC=C1)N%10	4.6_5
C%11(COC1=CC2=CC=CC=C2C=C1)=O.CC[CH](C)[CH](C%10=O)N%11.CC[CH](C)[CH](C(O)=O)N%10	4.6_6
C%11(COC1=CC2=CC=CC=C2C=C1)=O.CC[CH](C)[CH](C%10=O)N%11.CSCC[CH](C(O)=O)N%10	4.6_7
C%11(COC1=CC2=CC=CC=C2C=C1)=O.CC[CH](C)[CH](C%10=O)N%11.OC(C=C1)=CC=C1C[CH](C(O)=O)N%10	4.6_8
C%11(COC1=CC2=CC=CC=C2C=C1)=O.CC[CH](C)[CH](C%10=O)N%11.O=C(O)[CH](CC1=C(F)C(F)=C(F)C(F)=C1F)N%10	4.6_9
C%11(COC1=CC2=CC=CC=C2C=C1)=O.CSCC[CH](C%10=O)N%11.O=C(O)CN%10	4.7_1
C%11(COC1=CC2=CC=CC=C2C=C1)=O.CSCC[CH](C%10=O)N%11.CC(C)[CH](C(O)=O)N%10	4.7_2
C%11(COC1=CC2=CC=CC=C2C=C1)=O.CSCC[CH](C%10=O)N%11.CC(C)C[CH](C(O)=O)N%10	4.7_3
C%11(COC1=CC2=CC=CC=C2C=C1)=O.CSCC[CH](C%10=O)N%11.C[CH](C(O)=O)N%10	4.7_4
C%11(COC1=CC2=CC=CC=C2C=C1)=O.CSCC[CH](C%10=O)N%11.O=C(O)[CH](CC1=CC=CC=C1)N%10	4.7_5
C%11(COC1=CC2=CC=CC=C2C=C1)=O.CSCC[CH](C%10=O)N%11.CC[CH](C)[CH](C(O)=O)N%10	4.7_6
C%11(COC1=CC2=CC=CC=C2C=C1)=O.CSCC[CH](C%10=O)N%11.CSCC[CH](C(O)=O)N%10	4.7_7
C%11(COC1=CC2=CC=CC=C2C=C1)=O.CSCC[CH](C%10=O)N%11.OC(C=C1)=CC=C1C[CH](C(O)=O)N%10	4.7_8
C%11(COC1=CC2=CC=CC=C2C=C1)=O.CSCC[CH](C%10=O)N%11.O=C(O)[CH](CC1=C(F)C(F)=C(F)C(F)=C1F)N%10	4.7_9
C%11(COC1=CC2=CC=CC=C2C=C1)=O.OC(C=C1)=CC=C1C[CH](C%10=O)N%11.O=C(O)CN%10	4.8_1
C%11(COC1=CC2=CC=CC=C2C=C1)=O.OC(C=C1)=CC=C1C[CH](C%10=O)N%11.CC(C)[CH](C(O)=O)N%10	4.8_2
C%11(COC1=CC2=CC=CC=C2C=C1)=O.OC(C=C1)=CC=C1C[CH](C%10=O)N%11.CC(C)C[CH](C(O)=O)N%10	4.8_3
C%11(COC1=CC2=CC=CC=C2C=C1)=O.OC(C=C1)=CC=C1C[CH](C%10=O)N%11.C[CH](C(O)=O)N%10	4.8_4
C%11(COC1=CC2=CC=CC=C2C=C1)=O.OC(C=C1)=CC=C1C[CH](C%10=O)N%11.O=C(O)[CH](CC1=CC=CC=C1)N%10	4.8_5
C%11(COC1=CC2=CC=CC=C2C=C1)=O.OC(C=C1)=CC=C1C[CH](C%10=O)N%11.CC[CH](C)[CH](C(O)=O)N%10	4.8_6
C%11(COC1=CC2=CC=CC=C2C=C1)=O.OC(C=C1)=CC=C1C[CH](C%10=O)N%11.CSCC[CH](C(O)=O)N%10	4.8_7
C%11(COC1=CC2=CC=CC=C2C=C1)=O.OC(C=C1)=CC=C1C[CH](C%10=O)N%11.OC(C=C1)=CC=C1C[CH](C(O)=O)N%10	4.8_8
C%11(COC1=CC2=CC=CC=C2C=C1)=O.OC(C=C1)=CC=C1C[CH](C%10=O)N%11.O=C(O)[CH](CC1=C(F)C(F)=C(F)C(F)=C1F)N%10	4.8_9
C%10(COC1=CC2=CC=CC=C2C=C1)=O.O=C(O)CN%10	4.9_1
C%10(COC1=CC2=CC=CC=C2C=C1)=O.CC(C)[CH](C(O)=O)N%10	4.9_2
C%10(COC1=CC2=CC=CC=C2C=C1)=O.CC(C)C[CH](C(O)=O)N%10	4.9_3
C%10(COC1=CC2=CC=CC=C2C=C1)=O.C[CH](C(O)=O)N%10	4.9_4
C%10(COC1=CC2=CC=CC=C2C=C1)=O.O=C(O)[CH](CC1=CC=CC=C1)N%10	4.9_5
C%10(COC1=CC2=CC=CC=C2C=C1)=O.CC[CH](C)[CH](C(O)=O)N%10	4.9_6

C%10(COC1=CC2=CC=CC=C2C=C1)=O.CSCC[CH](C(O)=O)N%10	4.9_7
C%10(COC1=CC2=CC=CC=C2C=C1)=O.OC(C=C1)=CC=C1C[CH](C(O)=O)N%10	4.9_8
C%10(COC1=CC2=CC=CC=C2C=C1)=O.O=C(O)[CH](CC1=C(F)C(F)=C(F)C(F)=C1F)N%10	4.9_9
O=C%11CN1C2=C(C=CC=C2)C3=CC=CC=C31.O=C%10CN%11.O=C(O)CN%10	5.1_1
O=C%11CN1C2=C(C=CC=C2)C3=CC=CC=C31.O=C%10CN%11.CC(C)[CH](C(O)=O)N%10	5.1_2
O=C%11CN1C2=C(C=CC=C2)C3=CC=CC=C31.O=C%10CN%11.CC(C)C[CH](C(O)=O)N%10	5.1_3
O=C%11CN1C2=C(C=CC=C2)C3=CC=CC=C31.O=C%10CN%11.C[CH](C(O)=O)N%10	5.1_4
O=C%11CN1C2=C(C=CC=C2)C3=CC=CC=C31.O=C%10CN%11.O=C(O)[CH](CC1=CC=CC=C1)N%10	5.1_5
O=C%11CN1C2=C(C=CC=C2)C3=CC=CC=C31.O=C%10CN%11.CC[CH](C)[CH](C(O)=O)N%10	5.1_6
O=C%11CN1C2=C(C=CC=C2)C3=CC=CC=C31.O=C%10CN%11.CSCC[CH](C(O)=O)N%10	5.1_7
O=C%11CN1C2=C(C=CC=C2)C3=CC=CC=C31.O=C%10CN%11.OC(C=C1)=CC=C1C[CH](C(O)=O)N%10	5.1_8
O=C%11CN1C2=C(C=CC=C2)C3=CC=CC=C31.O=C%10CN%11.O=C(O)[CH](CC1=C(F)C(F)=C(F)C(F)=C1F)N%10	5.1_9
O=C%11CN1C2=C(C=CC=C2)C3=CC=CC=C31.CC(C)[CH](C%10=O)N%11.O=C(O)CN%10	5.2_1
O=C%11CN1C2=C(C=CC=C2)C3=CC=CC=C31.CC(C)[CH](C%10=O)N%11.CC(C)[CH](C(O)=O)N%10	5.2_2
O=C%11CN1C2=C(C=CC=C2)C3=CC=CC=C31.CC(C)[CH](C%10=O)N%11.CC(C)C[CH](C(O)=O)N%10	5.2_3
O=C%11CN1C2=C(C=CC=C2)C3=CC=CC=C31.CC(C)[CH](C%10=O)N%11.C[CH](C(O)=O)N%10	5.2_4
O=C%11CN1C2=C(C=CC=C2)C3=CC=CC=C31.CC(C)[CH](C%10=O)N%11.O=C(O)[CH](CC1=CC=CC=C1)N%10	5.2_5
O=C%11CN1C2=C(C=CC=C2)C3=CC=CC=C31.CC(C)[CH](C%10=O)N%11.CC[CH](C)[CH](C(O)=O)N%10	5.2_6
O=C%11CN1C2=C(C=CC=C2)C3=CC=CC=C31.CC(C)[CH](C%10=O)N%11.CSCC[CH](C(O)=O)N%10	5.2_7
O=C%11CN1C2=C(C=CC=C2)C3=CC=CC=C31.CC(C)[CH](C%10=O)N%11.OC(C=C1)=CC=C1C[CH](C(O)=O)N%10	5.2_8
O=C%11CN1C2=C(C=CC=C2)C3=CC=CC=C31.CC(C)[CH](C%10=O)N%11.O=C(O)[CH](CC1=C(F)C(F)=C(F)C(F)=C1F)N%10	5.2_9
O=C%11CN1C2=C(C=CC=C2)C3=CC=CC=C31.CC(C)C[CH](C%10=O)N%11.O=C(O)CN%10	5.3_1
O=C%11CN1C2=C(C=CC=C2)C3=CC=CC=C31.CC(C)C[CH](C%10=O)N%11.CC(C)[CH](C(O)=O)N%10	5.3_2
O=C%11CN1C2=C(C=CC=C2)C3=CC=CC=C31.CC(C)C[CH](C%10=O)N%11.CC(C)C[CH](C(O)=O)N%10	5.3_3
O=C%11CN1C2=C(C=CC=C2)C3=CC=CC=C31.CC(C)C[CH](C%10=O)N%11.C[CH](C(O)=O)N%10	5.3_4
O=C%11CN1C2=C(C=CC=C2)C3=CC=CC=C31.CC(C)C[CH](C%10=O)N%11.O=C(O)[CH](CC1=CC=CC=C1)N%10	5.3_5
O=C%11CN1C2=C(C=CC=C2)C3=CC=CC=C31.CC(C)C[CH](C%10=O)N%11.CC[CH](C)[CH](C(O)=O)N%10	5.3_6
O=C%11CN1C2=C(C=CC=C2)C3=CC=CC=C31.CC(C)C[CH](C%10=O)N%11.CSCC[CH](C(O)=O)N%10	5.3_7
O=C%11CN1C2=C(C=CC=C2)C3=CC=CC=C31.CC(C)C[CH](C%10=O)N%11.OC(C=C1)=CC=C1C[CH](C(O)=O)N%10	5.3_8

O=C%11CN1C2=C(C=CC=C2)C3=CC=CC=C31.CC(C)C[CH](C%10=O)N%11.O=C(O)[CH](CC1=C(F)C(F)=C(F)C(F)=C1F)N%10	5.3_9
O=C%11CN1C2=C(C=CC=C2)C3=CC=CC=C31.C[CH](C%10=O)N%11.O=C(O)CN%10	5.4_1
O=C%11CN1C2=C(C=CC=C2)C3=CC=CC=C31.C[CH](C%10=O)N%11.CC(C)[CH](C(O)=O)N%10	5.4_2
O=C%11CN1C2=C(C=CC=C2)C3=CC=CC=C31.C[CH](C%10=O)N%11.CC(C)C[CH](C(O)=O)N%10	5.4_3
O=C%11CN1C2=C(C=CC=C2)C3=CC=CC=C31.C[CH](C%10=O)N%11.C[CH](C(O)=O)N%10	5.4_4
O=C%11CN1C2=C(C=CC=C2)C3=CC=CC=C31.C[CH](C%10=O)N%11.O=C(O)[CH](CC1=CC=CC=C1)N%10	5.4_5
O=C%11CN1C2=C(C=CC=C2)C3=CC=CC=C31.C[CH](C%10=O)N%11.CC[CH](C)[CH](C(O)=O)N%10	5.4_6
O=C%11CN1C2=C(C=CC=C2)C3=CC=CC=C31.C[CH](C%10=O)N%11.CSCC[CH](C(O)=O)N%10	5.4_7
O=C%11CN1C2=C(C=CC=C2)C3=CC=CC=C31.C[CH](C%10=O)N%11.OC(C=C1)=CC=C1C[CH](C(O)=O)N%10	5.4_8
O=C%11CN1C2=C(C=CC=C2)C3=CC=CC=C31.C[CH](C%10=O)N%11.O=C(O)[CH](CC1=C(F)C(F)=C(F)C(F)=C1F)N%10	5.4_9
O=C%11CN1C2=C(C=CC=C2)C3=CC=CC=C31.O=C%10[CH](CC1=CC=CC=C1)N%11.O=C(O)CN%10	5.5_1
O=C%11CN1C2=C(C=CC=C2)C3=CC=CC=C31.O=C%10[CH](CC1=CC=CC=C1)N%11.CC(C)[CH](C(O)=O)N%10	5.5_2
O=C%11CN1C2=C(C=CC=C2)C3=CC=CC=C31.O=C%10[CH](CC1=CC=CC=C1)N%11.CC(C)C[CH](C(O)=O)N%10	5.5_3
O=C%11CN1C2=C(C=CC=C2)C3=CC=CC=C31.O=C%10[CH](CC1=CC=CC=C1)N%11.C[CH](C(O)=O)N%10	5.5_4
O=C%11CN1C2=C(C=CC=C2)C3=CC=CC=C31.O=C%10[CH](CC1=CC=CC=C1)N%11.O=C(O)[CH](CC1=CC=CC=C1)N%10	5.5_5
O=C%11CN1C2=C(C=CC=C2)C3=CC=CC=C31.O=C%10[CH](CC1=CC=CC=C1)N%11.CC[CH](C)[CH](C(O)=O)N%10	5.5_6
O=C%11CN1C2=C(C=CC=C2)C3=CC=CC=C31.O=C%10[CH](CC1=CC=CC=C1)N%11.CSCC[CH](C(O)=O)N%10	5.5_7
O=C%11CN1C2=C(C=CC=C2)C3=CC=CC=C31.O=C%10[CH](CC1=CC=CC=C1)N%11.OC(C=C1)=CC=C1C[CH](C(O)=O)N%10	5.5_8
O=C%11CN1C2=C(C=CC=C2)C3=CC=CC=C31.O=C%10[CH](CC1=CC=CC=C1)N%11.O=C(O)[CH](CC1=C(F)C(F)=C(F)C(F)=C1F)N%10	5.5_9
O=C%11CN1C2=C(C=CC=C2)C3=CC=CC=C31.CC[CH](C)[CH](C%10=O)N%11.O=C(O)CN%10	5.6_1
O=C%11CN1C2=C(C=CC=C2)C3=CC=CC=C31.CC[CH](C)[CH](C%10=O)N%11.CC(C)[CH](C(O)=O)N%10	5.6_2
O=C%11CN1C2=C(C=CC=C2)C3=CC=CC=C31.CC[CH](C)[CH](C%10=O)N%11.CC(C)C[CH](C(O)=O)N%10	5.6_3
O=C%11CN1C2=C(C=CC=C2)C3=CC=CC=C31.CC[CH](C)[CH](C%10=O)N%11.C[CH](C(O)=O)N%10	5.6_4
O=C%11CN1C2=C(C=CC=C2)C3=CC=CC=C31.CC[CH](C)[CH](C%10=O)N%11.O=C(O)[CH](CC1=CC=CC=C1)N%10	5.6_5
O=C%11CN1C2=C(C=CC=C2)C3=CC=CC=C31.CC[CH](C)[CH](C%10=O)N%11.CC[CH](C)[CH](C(O)=O)N%10	5.6_6
O=C%11CN1C2=C(C=CC=C2)C3=CC=CC=C31.CC[CH](C)[CH](C%10=O)N%11.CSCC[CH](C(O	5.6_7

)=O)N%10	
O=C%11CN1C2=C(C=CC=C2)C3=CC=CC=C31.CC[CH](C)[CH](C%10=O)N%11.OC(C=C1)=CC=C1C[CH](C(O)=O)N%10	5.6_8
O=C%11CN1C2=C(C=CC=C2)C3=CC=CC=C31.CC[CH](C)[CH](C%10=O)N%11.O=C(O)[CH](CC1=C(F)C(F)=C(F)C(F)=C1F)N%10	5.6_9
O=C%11CN1C2=C(C=CC=C2)C3=CC=CC=C31.CSCC[CH](C%10=O)N%11.O=C(O)CN%10	5.7_1
O=C%11CN1C2=C(C=CC=C2)C3=CC=CC=C31.CSCC[CH](C%10=O)N%11.CC(C)[CH](C(O)=O)N%10	5.7_2
O=C%11CN1C2=C(C=CC=C2)C3=CC=CC=C31.CSCC[CH](C%10=O)N%11.CC(C)C[CH](C(O)=O)N%10	5.7_3
O=C%11CN1C2=C(C=CC=C2)C3=CC=CC=C31.CSCC[CH](C%10=O)N%11.C[CH](C(O)=O)N%10	5.7_4
O=C%11CN1C2=C(C=CC=C2)C3=CC=CC=C31.CSCC[CH](C%10=O)N%11.O=C(O)[CH](CC1=CC=CC=C1)N%10	5.7_5
O=C%11CN1C2=C(C=CC=C2)C3=CC=CC=C31.CSCC[CH](C%10=O)N%11.CC[CH](C)[CH](C(O)=O)N%10	5.7_6
O=C%11CN1C2=C(C=CC=C2)C3=CC=CC=C31.CSCC[CH](C%10=O)N%11.CSCC[CH](C(O)=O)N%10	5.7_7
O=C%11CN1C2=C(C=CC=C2)C3=CC=CC=C31.CSCC[CH](C%10=O)N%11.OC(C=C1)=CC=C1C[CH](C(O)=O)N%10	5.7_8
O=C%11CN1C2=C(C=CC=C2)C3=CC=CC=C31.CSCC[CH](C%10=O)N%11.O=C(O)[CH](CC1=C(F)C(F)=C(F)C(F)=C1F)N%10	5.7_9
O=C%11CN1C2=C(C=CC=C2)C3=CC=CC=C31.OC(C=C1)=CC=C1C[CH](C%10=O)N%11.O=C(O)CN%10	5.8_1
O=C%11CN1C2=C(C=CC=C2)C3=CC=CC=C31.OC(C=C1)=CC=C1C[CH](C%10=O)N%11.CC(C)[CH](C(O)=O)N%10	5.8_2
O=C%11CN1C2=C(C=CC=C2)C3=CC=CC=C31.OC(C=C1)=CC=C1C[CH](C%10=O)N%11.CC(C)C[CH](C(O)=O)N%10	5.8_3
O=C%11CN1C2=C(C=CC=C2)C3=CC=CC=C31.OC(C=C1)=CC=C1C[CH](C%10=O)N%11.C[CH](C(O)=O)N%10	5.8_4
O=C%11CN1C2=C(C=CC=C2)C3=CC=CC=C31.OC(C=C1)=CC=C1C[CH](C%10=O)N%11.O=C(O)[CH](CC1=CC=CC=C1)N%10	5.8_5
O=C%11CN1C2=C(C=CC=C2)C3=CC=CC=C31.OC(C=C1)=CC=C1C[CH](C%10=O)N%11.CC[CH](C)[CH](C(O)=O)N%10	5.8_6
O=C%11CN1C2=C(C=CC=C2)C3=CC=CC=C31.OC(C=C1)=CC=C1C[CH](C%10=O)N%11.CSCC[CH](C(O)=O)N%10	5.8_7
O=C%11CN1C2=C(C=CC=C2)C3=CC=CC=C31.OC(C=C1)=CC=C1C[CH](C%10=O)N%11.OC(C=C1)=CC=C1C[CH](C(O)=O)N%10	5.8_8
O=C%11CN1C2=C(C=CC=C2)C3=CC=CC=C31.OC(C=C1)=CC=C1C[CH](C%10=O)N%11.O=C(O)[CH](CC1=C(F)C(F)=C(F)C(F)=C1F)N%10	5.8_9
O=C%10CN1C2=C(C=CC=C2)C3=CC=CC=C31.O=C(O)CN%10	5.9_1
O=C%10CN1C2=C(C=CC=C2)C3=CC=CC=C31.CC(C)[CH](C(O)=O)N%10	5.9_2
O=C%10CN1C2=C(C=CC=C2)C3=CC=CC=C31.CC(C)C[CH](C(O)=O)N%10	5.9_3
O=C%10CN1C2=C(C=CC=C2)C3=CC=CC=C31.C[CH](C(O)=O)N%10	5.9_4
O=C%10CN1C2=C(C=CC=C2)C3=CC=CC=C31.O=C(O)[CH](CC1=CC=CC=C1)N%10	5.9_5
O=C%10CN1C2=C(C=CC=C2)C3=CC=CC=C31.CC[CH](C)[CH](C(O)=O)N%10	5.9_6
O=C%10CN1C2=C(C=CC=C2)C3=CC=CC=C31.CSCC[CH](C(O)=O)N%10	5.9_7
O=C%10CN1C2=C(C=CC=C2)C3=CC=CC=C31.OC(C=C1)=CC=C1C[CH](C(O)=O)N%10	5.9_8

O=C%10CN1C2=C(C=CC=C2)C3=CC=CC=C31.O=C(O)[CH](CC1=C(F)C(F)=C(F)C(F)=C1F)N%10	5.9_9
O=C%11COC1=CC=C(C(F)(F)F)C=C1.O=C%10CN%11.O=C(O)CN%10	6.1_1
O=C%11COC1=CC=C(C(F)(F)F)C=C1.O=C%10CN%11.CC(C)[CH](C(O)=O)N%10	6.1_2
O=C%11COC1=CC=C(C(F)(F)F)C=C1.O=C%10CN%11.CC(C)C[CH](C(O)=O)N%10	6.1_3
O=C%11COC1=CC=C(C(F)(F)F)C=C1.O=C%10CN%11.C[CH](C(O)=O)N%10	6.1_4
O=C%11COC1=CC=C(C(F)(F)F)C=C1.O=C%10CN%11.O=C(O)[CH](CC1=CC=CC=C1)N%10	6.1_5
O=C%11COC1=CC=C(C(F)(F)F)C=C1.O=C%10CN%11.CC[CH](C)[CH](C(O)=O)N%10	6.1_6
O=C%11COC1=CC=C(C(F)(F)F)C=C1.O=C%10CN%11.CSCC[CH](C(O)=O)N%10	6.1_7
O=C%11COC1=CC=C(C(F)(F)F)C=C1.O=C%10CN%11.OC(C=C1)=CC=C1C[CH](C(O)=O)N%10	6.1_8
O=C%11COC1=CC=C(C(F)(F)F)C=C1.O=C%10CN%11.O=C(O)[CH](CC1=C(F)C(F)=C(F)C(F)=C1F)N%10	6.1_9
O=C%11COC1=CC=C(C(F)(F)F)C=C1.CC(C)[CH](C%10=O)N%11.O=C(O)CN%10	6.2_1
O=C%11COC1=CC=C(C(F)(F)F)C=C1.CC(C)[CH](C%10=O)N%11.CC(C)[CH](C(O)=O)N%10	6.2_2
O=C%11COC1=CC=C(C(F)(F)F)C=C1.CC(C)[CH](C%10=O)N%11.CC(C)C[CH](C(O)=O)N%10	6.2_3
O=C%11COC1=CC=C(C(F)(F)F)C=C1.CC(C)[CH](C%10=O)N%11.C[CH](C(O)=O)N%10	6.2_4
O=C%11COC1=CC=C(C(F)(F)F)C=C1.CC(C)[CH](C%10=O)N%11.O=C(O)[CH](CC1=CC=CC=C1)N%10	6.2_5
O=C%11COC1=CC=C(C(F)(F)F)C=C1.CC(C)[CH](C%10=O)N%11.CC[CH](C)[CH](C(O)=O)N%10	6.2_6
O=C%11COC1=CC=C(C(F)(F)F)C=C1.CC(C)[CH](C%10=O)N%11.CSCC[CH](C(O)=O)N%10	6.2_7
O=C%11COC1=CC=C(C(F)(F)F)C=C1.CC(C)[CH](C%10=O)N%11.OC(C=C1)=CC=C1C[CH](C(O)=O)N%10	6.2_8
O=C%11COC1=CC=C(C(F)(F)F)C=C1.CC(C)[CH](C%10=O)N%11.O=C(O)[CH](CC1=C(F)C(F)=C(F)C(F)=C1F)N%10	6.2_9
O=C%11COC1=CC=C(C(F)(F)F)C=C1.CC(C)C[CH](C%10=O)N%11.O=C(O)CN%10	6.3_1
O=C%11COC1=CC=C(C(F)(F)F)C=C1.CC(C)C[CH](C%10=O)N%11.CC(C)[CH](C(O)=O)N%10	6.3_2
O=C%11COC1=CC=C(C(F)(F)F)C=C1.CC(C)C[CH](C%10=O)N%11.CC(C)C[CH](C(O)=O)N%10	6.3_3
O=C%11COC1=CC=C(C(F)(F)F)C=C1.CC(C)C[CH](C%10=O)N%11.C[CH](C(O)=O)N%10	6.3_4
O=C%11COC1=CC=C(C(F)(F)F)C=C1.CC(C)C[CH](C%10=O)N%11.O=C(O)[CH](CC1=CC=CC=C1)N%10	6.3_5
O=C%11COC1=CC=C(C(F)(F)F)C=C1.CC(C)C[CH](C%10=O)N%11.CC[CH](C)[CH](C(O)=O)N%10	6.3_6
O=C%11COC1=CC=C(C(F)(F)F)C=C1.CC(C)C[CH](C%10=O)N%11.CSCC[CH](C(O)=O)N%10	6.3_7
O=C%11COC1=CC=C(C(F)(F)F)C=C1.CC(C)C[CH](C%10=O)N%11.OC(C=C1)=CC=C1C[CH](C(O)=O)N%10	6.3_8
O=C%11COC1=CC=C(C(F)(F)F)C=C1.CC(C)C[CH](C%10=O)N%11.O=C(O)[CH](CC1=C(F)C(F)=C(F)C(F)=C1F)N%10	6.3_9
O=C%11COC1=CC=C(C(F)(F)F)C=C1.C[CH](C%10=O)N%11.O=C(O)CN%10	6.4_1
O=C%11COC1=CC=C(C(F)(F)F)C=C1.C[CH](C%10=O)N%11.CC(C)[CH](C(O)=O)N%10	6.4_2
O=C%11COC1=CC=C(C(F)(F)F)C=C1.C[CH](C%10=O)N%11.CC(C)C[CH](C(O)=O)N%10	6.4_3
O=C%11COC1=CC=C(C(F)(F)F)C=C1.C[CH](C%10=O)N%11.C[CH](C(O)=O)N%10	6.4_4
O=C%11COC1=CC=C(C(F)(F)F)C=C1.C[CH](C%10=O)N%11.O=C(O)[CH](CC1=CC=CC=C1)N%10	6.4_5
O=C%11COC1=CC=C(C(F)(F)F)C=C1.C[CH](C%10=O)N%11.CC[CH](C)[CH](C(O)=O)N%10	6.4_6
O=C%11COC1=CC=C(C(F)(F)F)C=C1.C[CH](C%10=O)N%11.CSCC[CH](C(O)=O)N%10	6.4_7

O=C%11COC1=CC=C(C(F)(F)F)C=C1.C[CH](C%10=O)N%11.OC(C=C1)=CC=C1C[CH](C(O)=O)N%10	6.4_8
O=C%11COC1=CC=C(C(F)(F)F)C=C1.C[CH](C%10=O)N%11.O=C(O)[CH](CC1=C(F)C(F)=C(F)C(F)=C1F)N%10	6.4_9
O=C%11COC1=CC=C(C(F)(F)F)C=C1.O=C%10[CH](CC1=CC=CC=C1)N%11.O=C(O)CN%10	6.5_1
O=C%11COC1=CC=C(C(F)(F)F)C=C1.O=C%10[CH](CC1=CC=CC=C1)N%11.CC(C)[CH](C(O)=O)N%10	6.5_2
O=C%11COC1=CC=C(C(F)(F)F)C=C1.O=C%10[CH](CC1=CC=CC=C1)N%11.CC(C)C[CH](C(O)=O)N%10	6.5_3
O=C%11COC1=CC=C(C(F)(F)F)C=C1.O=C%10[CH](CC1=CC=CC=C1)N%11.C[CH](C(O)=O)N%10	6.5_4
O=C%11COC1=CC=C(C(F)(F)F)C=C1.O=C%10[CH](CC1=CC=CC=C1)N%11.O=C(O)[CH](CC1=CC=CC=C1)N%10	6.5_5
O=C%11COC1=CC=C(C(F)(F)F)C=C1.O=C%10[CH](CC1=CC=CC=C1)N%11.CC[CH](C)[CH](C(O)=O)N%10	6.5_6
O=C%11COC1=CC=C(C(F)(F)F)C=C1.O=C%10[CH](CC1=CC=CC=C1)N%11.CSCC[CH](C(O)=O)N%10	6.5_7
O=C%11COC1=CC=C(C(F)(F)F)C=C1.O=C%10[CH](CC1=CC=CC=C1)N%11.OC(C=C1)=CC=C1C[CH](C(O)=O)N%10	6.5_8
O=C%11COC1=CC=C(C(F)(F)F)C=C1.O=C%10[CH](CC1=CC=CC=C1)N%11.O=C(O)[CH](CC1=C(F)C(F)=C(F)C(F)=C1F)N%10	6.5_9
O=C%11COC1=CC=C(C(F)(F)F)C=C1.CC[CH](C)[CH](C%10=O)N%11.O=C(O)CN%10	6.6_1
O=C%11COC1=CC=C(C(F)(F)F)C=C1.CC[CH](C)[CH](C%10=O)N%11.CC(C)[CH](C(O)=O)N%10	6.6_2
O=C%11COC1=CC=C(C(F)(F)F)C=C1.CC[CH](C)[CH](C%10=O)N%11.CC(C)C[CH](C(O)=O)N%10	6.6_3
O=C%11COC1=CC=C(C(F)(F)F)C=C1.CC[CH](C)[CH](C%10=O)N%11.C[CH](C(O)=O)N%10	6.6_4
O=C%11COC1=CC=C(C(F)(F)F)C=C1.CC[CH](C)[CH](C%10=O)N%11.O=C(O)[CH](CC1=CC=CC=C1)N%10	6.6_5
O=C%11COC1=CC=C(C(F)(F)F)C=C1.CC[CH](C)[CH](C%10=O)N%11.CC[CH](C)[CH](C(O)=O)N%10	6.6_6
O=C%11COC1=CC=C(C(F)(F)F)C=C1.CC[CH](C)[CH](C%10=O)N%11.CSCC[CH](C(O)=O)N%10	6.6_7
O=C%11COC1=CC=C(C(F)(F)F)C=C1.CC[CH](C)[CH](C%10=O)N%11.OC(C=C1)=CC=C1C[CH](C(O)=O)N%10	6.6_8
O=C%11COC1=CC=C(C(F)(F)F)C=C1.CC[CH](C)[CH](C%10=O)N%11.O=C(O)[CH](CC1=C(F)C(F)=C(F)C(F)=C1F)N%10	6.6_9
O=C%11COC1=CC=C(C(F)(F)F)C=C1.CSCC[CH](C%10=O)N%11.O=C(O)CN%10	6.7_1
O=C%11COC1=CC=C(C(F)(F)F)C=C1.CSCC[CH](C%10=O)N%11.CC(C)[CH](C(O)=O)N%10	6.7_2
O=C%11COC1=CC=C(C(F)(F)F)C=C1.CSCC[CH](C%10=O)N%11.CC(C)C[CH](C(O)=O)N%10	6.7_3
O=C%11COC1=CC=C(C(F)(F)F)C=C1.CSCC[CH](C%10=O)N%11.C[CH](C(O)=O)N%10	6.7_4
O=C%11COC1=CC=C(C(F)(F)F)C=C1.CSCC[CH](C%10=O)N%11.O=C(O)[CH](CC1=CC=CC=C1)N%10	6.7_5
O=C%11COC1=CC=C(C(F)(F)F)C=C1.CSCC[CH](C%10=O)N%11.CC[CH](C)[CH](C(O)=O)N%10	6.7_6
O=C%11COC1=CC=C(C(F)(F)F)C=C1.CSCC[CH](C%10=O)N%11.CSCC[CH](C(O)=O)N%10	6.7_7
O=C%11COC1=CC=C(C(F)(F)F)C=C1.CSCC[CH](C%10=O)N%11.OC(C=C1)=CC=C1C[CH](C(O)=O)N%10	6.7_8

O=C%11COC1=CC=C(C(F)(F)F)C=C1.CSCC[CH](C%10=O)N%11.O=C(O)[CH](CC1=C(F)C(F)=C(F)C(F)=C1F)N%10	6.7_9
O=C%11COC1=CC=C(C(F)(F)F)C=C1.OC(C=C1)=CC=C1C[CH](C%10=O)N%11.O=C(O)CN%10	6.8_1
O=C%11COC1=CC=C(C(F)(F)F)C=C1.OC(C=C1)=CC=C1C[CH](C%10=O)N%11.CC(C)[CH](C(O)=O)N%10	6.8_2
O=C%11COC1=CC=C(C(F)(F)F)C=C1.OC(C=C1)=CC=C1C[CH](C%10=O)N%11.CC(C)C[CH](C(O)=O)N%10	6.8_3
O=C%11COC1=CC=C(C(F)(F)F)C=C1.OC(C=C1)=CC=C1C[CH](C%10=O)N%11.C[CH](C(O)=O)N%10	6.8_4
O=C%11COC1=CC=C(C(F)(F)F)C=C1.OC(C=C1)=CC=C1C[CH](C%10=O)N%11.O=C(O)[CH](CC1=CC=CC=C1)N%10	6.8_5
O=C%11COC1=CC=C(C(F)(F)F)C=C1.OC(C=C1)=CC=C1C[CH](C%10=O)N%11.CC[CH](C)[CH](C(O)=O)N%10	6.8_6
O=C%11COC1=CC=C(C(F)(F)F)C=C1.OC(C=C1)=CC=C1C[CH](C%10=O)N%11.CSCC[CH](C(O)=O)N%10	6.8_7
O=C%11COC1=CC=C(C(F)(F)F)C=C1.OC(C=C1)=CC=C1C[CH](C%10=O)N%11.OC(C=C1)=CC=C1C[CH](C(O)=O)N%10	6.8_8
O=C%11COC1=CC=C(C(F)(F)F)C=C1.OC(C=C1)=CC=C1C[CH](C%10=O)N%11.O=C(O)[CH](CC1=C(F)C(F)=C(F)C(F)=C1F)N%10	6.8_9
O=C%10COC1=CC=C(C(F)(F)F)C=C1.O=C(O)CN%10	6.9_1
O=C%10COC1=CC=C(C(F)(F)F)C=C1.CC(C)[CH](C(O)=O)N%10	6.9_2
O=C%10COC1=CC=C(C(F)(F)F)C=C1.CC(C)C[CH](C(O)=O)N%10	6.9_3
O=C%10COC1=CC=C(C(F)(F)F)C=C1.C[CH](C(O)=O)N%10	6.9_4
O=C%10COC1=CC=C(C(F)(F)F)C=C1.O=C(O)[CH](CC1=CC=CC=C1)N%10	6.9_5
O=C%10COC1=CC=C(C(F)(F)F)C=C1.CC[CH](C)[CH](C(O)=O)N%10	6.9_6
O=C%10COC1=CC=C(C(F)(F)F)C=C1.CSCC[CH](C(O)=O)N%10	6.9_7
O=C%10COC1=CC=C(C(F)(F)F)C=C1.OC(C=C1)=CC=C1C[CH](C(O)=O)N%10	6.9_8
O=C%10COC1=CC=C(C(F)(F)F)C=C1.O=C(O)[CH](CC1=C(F)C(F)=C(F)C(F)=C1F)N%10	6.9_9
C%11(COC1=CC(CCCC2)=C2C=C1)=O.O=C%10CN%11.O=C(O)CN%10	7.1_1
C%11(COC1=CC(CCCC2)=C2C=C1)=O.O=C%10CN%11.CC(C)[CH](C(O)=O)N%10	7.1_2
C%11(COC1=CC(CCCC2)=C2C=C1)=O.O=C%10CN%11.CC(C)C[CH](C(O)=O)N%10	7.1_3
C%11(COC1=CC(CCCC2)=C2C=C1)=O.O=C%10CN%11.C[CH](C(O)=O)N%10	7.1_4
C%11(COC1=CC(CCCC2)=C2C=C1)=O.O=C%10CN%11.O=C(O)[CH](CC1=CC=CC=C1)N%10	7.1_5
C%11(COC1=CC(CCCC2)=C2C=C1)=O.O=C%10CN%11.CC[CH](C)[CH](C(O)=O)N%10	7.1_6
C%11(COC1=CC(CCCC2)=C2C=C1)=O.O=C%10CN%11.CSCC[CH](C(O)=O)N%10	7.1_7
C%11(COC1=CC(CCCC2)=C2C=C1)=O.O=C%10CN%11.OC(C=C1)=CC=C1C[CH](C(O)=O)N%10	7.1_8
C%11(COC1=CC(CCCC2)=C2C=C1)=O.O=C%10CN%11.O=C(O)[CH](CC1=C(F)C(F)=C(F)C(F)=C1F)N%10	7.1_9
C%11(COC1=CC(CCCC2)=C2C=C1)=O.CC(C)[CH](C%10=O)N%11.O=C(O)CN%10	7.2_1
C%11(COC1=CC(CCCC2)=C2C=C1)=O.CC(C)[CH](C%10=O)N%11.CC(C)[CH](C(O)=O)N%10	7.2_2
C%11(COC1=CC(CCCC2)=C2C=C1)=O.CC(C)[CH](C%10=O)N%11.CC(C)C[CH](C(O)=O)N%10	7.2_3
C%11(COC1=CC(CCCC2)=C2C=C1)=O.CC(C)[CH](C%10=O)N%11.C[CH](C(O)=O)N%10	7.2_4
C%11(COC1=CC(CCCC2)=C2C=C1)=O.CC(C)[CH](C%10=O)N%11.O=C(O)[CH](CC1=CC=CC=C1)N%10	7.2_5
C%11(COC1=CC(CCCC2)=C2C=C1)=O.CC(C)[CH](C%10=O)N%11.CC[CH](C)[CH](C(O)=O)N%10	7.2_6

C%11(COC1=CC(CCCC2)=C2C=C1)=O.CC(C)[CH](C%10=O)N%11.CSCC[CH](C(O)=O)N%10	7.2_7
C%11(COC1=CC(CCCC2)=C2C=C1)=O.CC(C)[CH](C%10=O)N%11.OC(C=C1)=CC=C1C[CH](C(O)=O)N%10	7.2_8
C%11(COC1=CC(CCCC2)=C2C=C1)=O.CC(C)[CH](C%10=O)N%11.O=C(O)[CH](CC1=C(F)C(F)=C(F)C(F)=C1F)N%10	7.2_9
C%11(COC1=CC(CCCC2)=C2C=C1)=O.CC(C)C[CH](C%10=O)N%11.O=C(O)CN%10	7.3_1
C%11(COC1=CC(CCCC2)=C2C=C1)=O.CC(C)C[CH](C%10=O)N%11.CC(C)[CH](C(O)=O)N%10	7.3_2
C%11(COC1=CC(CCCC2)=C2C=C1)=O.CC(C)C[CH](C%10=O)N%11.CC(C)C[CH](C(O)=O)N%10	7.3_3
C%11(COC1=CC(CCCC2)=C2C=C1)=O.CC(C)C[CH](C%10=O)N%11.C[CH](C(O)=O)N%10	7.3_4
C%11(COC1=CC(CCCC2)=C2C=C1)=O.CC(C)C[CH](C%10=O)N%11.O=C(O)[CH](CC1=CC=CC=C1)N%10	7.3_5
C%11(COC1=CC(CCCC2)=C2C=C1)=O.CC(C)C[CH](C%10=O)N%11.CC[CH](C)[CH](C(O)=O)N%10	7.3_6
C%11(COC1=CC(CCCC2)=C2C=C1)=O.CC(C)C[CH](C%10=O)N%11.CSCC[CH](C(O)=O)N%10	7.3_7
C%11(COC1=CC(CCCC2)=C2C=C1)=O.CC(C)C[CH](C%10=O)N%11.OC(C=C1)=CC=C1C[CH](C(O)=O)N%10	7.3_8
C%11(COC1=CC(CCCC2)=C2C=C1)=O.CC(C)C[CH](C%10=O)N%11.O=C(O)[CH](CC1=C(F)C(F)=C(F)C(F)=C1F)N%10	7.3_9
C%11(COC1=CC(CCCC2)=C2C=C1)=O.C[CH](C%10=O)N%11.O=C(O)CN%10	7.4_1
C%11(COC1=CC(CCCC2)=C2C=C1)=O.C[CH](C%10=O)N%11.CC(C)[CH](C(O)=O)N%10	7.4_2
C%11(COC1=CC(CCCC2)=C2C=C1)=O.C[CH](C%10=O)N%11.CC(C)C[CH](C(O)=O)N%10	7.4_3
C%11(COC1=CC(CCCC2)=C2C=C1)=O.C[CH](C%10=O)N%11.C[CH](C(O)=O)N%10	7.4_4
C%11(COC1=CC(CCCC2)=C2C=C1)=O.C[CH](C%10=O)N%11.O=C(O)[CH](CC1=CC=CC=C1)N%10	7.4_5
C%11(COC1=CC(CCCC2)=C2C=C1)=O.C[CH](C%10=O)N%11.CC[CH](C)[CH](C(O)=O)N%10	7.4_6
C%11(COC1=CC(CCCC2)=C2C=C1)=O.C[CH](C%10=O)N%11.CSCC[CH](C(O)=O)N%10	7.4_7
C%11(COC1=CC(CCCC2)=C2C=C1)=O.C[CH](C%10=O)N%11.OC(C=C1)=CC=C1C[CH](C(O)=O)N%10	7.4_8
C%11(COC1=CC(CCCC2)=C2C=C1)=O.C[CH](C%10=O)N%11.O=C(O)[CH](CC1=C(F)C(F)=C(F)C(F)=C1F)N%10	7.4_9
C%11(COC1=CC(CCCC2)=C2C=C1)=O.O=C%10[CH](CC1=CC=CC=C1)N%11.O=C(O)CN%10	7.5_1
C%11(COC1=CC(CCCC2)=C2C=C1)=O.O=C%10[CH](CC1=CC=CC=C1)N%11.CC(C)[CH](C(O)=O)N%10	7.5_2
C%11(COC1=CC(CCCC2)=C2C=C1)=O.O=C%10[CH](CC1=CC=CC=C1)N%11.CC(C)C[CH](C(O)=O)N%10	7.5_3
C%11(COC1=CC(CCCC2)=C2C=C1)=O.O=C%10[CH](CC1=CC=CC=C1)N%11.C[CH](C(O)=O)N%10	7.5_4
C%11(COC1=CC(CCCC2)=C2C=C1)=O.O=C%10[CH](CC1=CC=CC=C1)N%11.O=C(O)[CH](CC1=CC=CC=C1)N%10	7.5_5
C%11(COC1=CC(CCCC2)=C2C=C1)=O.O=C%10[CH](CC1=CC=CC=C1)N%11.CC[CH](C)[CH](C(O)=O)N%10	7.5_6
C%11(COC1=CC(CCCC2)=C2C=C1)=O.O=C%10[CH](CC1=CC=CC=C1)N%11.CSCC[CH](C(O)=O)N%10	7.5_7
C%11(COC1=CC(CCCC2)=C2C=C1)=O.O=C%10[CH](CC1=CC=CC=C1)N%11.OC(C=C1)=CC=C1C[CH](C(O)=O)N%10	7.5_8
C%11(COC1=CC(CCCC2)=C2C=C1)=O.O=C%10[CH](CC1=CC=CC=C1)N%11.O=C(O)[CH](CC1=C(F)C(F)=C(F)C(F)=C1F)N%10	7.5_9

C%11(COC1=CC(CCCC2)=C2C=C1)=O.CC[CH](C)[CH](C%10=O)N%11.O=C(O)CN%10	7.6_1
C%11(COC1=CC(CCCC2)=C2C=C1)=O.CC[CH](C)[CH](C%10=O)N%11.CC(C)[CH](C(O)=O)N%10	7.6_2
C%11(COC1=CC(CCCC2)=C2C=C1)=O.CC[CH](C)[CH](C%10=O)N%11.CC(C)C[CH](C(O)=O)N%10	7.6_3
C%11(COC1=CC(CCCC2)=C2C=C1)=O.CC[CH](C)[CH](C%10=O)N%11.C[CH](C(O)=O)N%10	7.6_4
C%11(COC1=CC(CCCC2)=C2C=C1)=O.CC[CH](C)[CH](C%10=O)N%11.O=C(O)[CH](CC1=CC=CC=C1)N%10	7.6_5
C%11(COC1=CC(CCCC2)=C2C=C1)=O.CC[CH](C)[CH](C%10=O)N%11.CC[CH](C)[CH](C(O)=O)N%10	7.6_6
C%11(COC1=CC(CCCC2)=C2C=C1)=O.CC[CH](C)[CH](C%10=O)N%11.CSCC[CH](C(O)=O)N%10	7.6_7
C%11(COC1=CC(CCCC2)=C2C=C1)=O.CC[CH](C)[CH](C%10=O)N%11.OC(C=C1)=CC=C1C[CH](C(O)=O)N%10	7.6_8
C%11(COC1=CC(CCCC2)=C2C=C1)=O.CC[CH](C)[CH](C%10=O)N%11.O=C(O)[CH](CC1=C(F)C(F)=C(F)C(F)=C1F)N%10	7.6_9
C%11(COC1=CC(CCCC2)=C2C=C1)=O.CSCC[CH](C%10=O)N%11.O=C(O)CN%10	7.7_1
C%11(COC1=CC(CCCC2)=C2C=C1)=O.CSCC[CH](C%10=O)N%11.CC(C)[CH](C(O)=O)N%10	7.7_2
C%11(COC1=CC(CCCC2)=C2C=C1)=O.CSCC[CH](C%10=O)N%11.CC(C)C[CH](C(O)=O)N%10	7.7_3
C%11(COC1=CC(CCCC2)=C2C=C1)=O.CSCC[CH](C%10=O)N%11.C[CH](C(O)=O)N%10	7.7_4
C%11(COC1=CC(CCCC2)=C2C=C1)=O.CSCC[CH](C%10=O)N%11.O=C(O)[CH](CC1=CC=CC=C1)N%10	7.7_5
C%11(COC1=CC(CCCC2)=C2C=C1)=O.CSCC[CH](C%10=O)N%11.CC[CH](C)[CH](C(O)=O)N%10	7.7_6
C%11(COC1=CC(CCCC2)=C2C=C1)=O.CSCC[CH](C%10=O)N%11.CSCC[CH](C(O)=O)N%10	7.7_7
C%11(COC1=CC(CCCC2)=C2C=C1)=O.CSCC[CH](C%10=O)N%11.OC(C=C1)=CC=C1C[CH](C(O)=O)N%10	7.7_8
C%11(COC1=CC(CCCC2)=C2C=C1)=O.CSCC[CH](C%10=O)N%11.O=C(O)[CH](CC1=C(F)C(F)=C(F)C(F)=C1F)N%10	7.7_9
C%11(COC1=CC(CCCC2)=C2C=C1)=O.OC(C=C1)=CC=C1C[CH](C%10=O)N%11.O=C(O)CN%10	7.8_1
C%11(COC1=CC(CCCC2)=C2C=C1)=O.OC(C=C1)=CC=C1C[CH](C%10=O)N%11.CC(C)[CH](C(O)=O)N%10	7.8_2
C%11(COC1=CC(CCCC2)=C2C=C1)=O.OC(C=C1)=CC=C1C[CH](C%10=O)N%11.CC(C)C[CH](C(O)=O)N%10	7.8_3
C%11(COC1=CC(CCCC2)=C2C=C1)=O.OC(C=C1)=CC=C1C[CH](C%10=O)N%11.C[CH](C(O)=O)N%10	7.8_4
C%11(COC1=CC(CCCC2)=C2C=C1)=O.OC(C=C1)=CC=C1C[CH](C%10=O)N%11.O=C(O)[CH](CC1=CC=CC=C1)N%10	7.8_5
C%11(COC1=CC(CCCC2)=C2C=C1)=O.OC(C=C1)=CC=C1C[CH](C%10=O)N%11.CC[CH](C)[CH](C(O)=O)N%10	7.8_6
C%11(COC1=CC(CCCC2)=C2C=C1)=O.OC(C=C1)=CC=C1C[CH](C%10=O)N%11.CSCC[CH](C(O)=O)N%10	7.8_7
C%11(COC1=CC(CCCC2)=C2C=C1)=O.OC(C=C1)=CC=C1C[CH](C%10=O)N%11.OC(C=C1)=CC=C1C[CH](C(O)=O)N%10	7.8_8
C%11(COC1=CC(CCCC2)=C2C=C1)=O.OC(C=C1)=CC=C1C[CH](C%10=O)N%11.O=C(O)[CH](CC1=C(F)C(F)=C(F)C(F)=C1F)N%10	7.8_9
C%10(COC1=CC(CCCC2)=C2C=C1)=O.O=C(O)CN%10	7.9_1

C%10(COC1=CC(CCCC2)=C2C=C1)=O.CC(C)[CH](C(O)=O)N%10	7.9_2
C%10(COC1=CC(CCCC2)=C2C=C1)=O.CC(C)C[CH](C(O)=O)N%10	7.9_3
C%10(COC1=CC(CCCC2)=C2C=C1)=O.C[CH](C(O)=O)N%10	7.9_4
C%10(COC1=CC(CCCC2)=C2C=C1)=O.O=C(O)[CH](CC1=CC=CC=C1)N%10	7.9_5
C%10(COC1=CC(CCCC2)=C2C=C1)=O.CC[CH](C)[CH](C(O)=O)N%10	7.9_6
C%10(COC1=CC(CCCC2)=C2C=C1)=O.CSCC[CH](C(O)=O)N%10	7.9_7
C%10(COC1=CC(CCCC2)=C2C=C1)=O.OC(C=C1)=CC=C1C[CH](C(O)=O)N%10	7.9_8
C%10(COC1=CC(CCCC2)=C2C=C1)=O.O=C(O)[CH](CC1=C(F)C(F)=C(F)C(F)=C1F)N%10	7.9_9
O=C%11COC1=CC=CC2=C1CCCC2.O=C%10CN%11.O=C(O)CN%10	8.1_1
O=C%11COC1=CC=CC2=C1CCCC2.O=C%10CN%11.CC(C)[CH](C(O)=O)N%10	8.1_2
O=C%11COC1=CC=CC2=C1CCCC2.O=C%10CN%11.CC(C)C[CH](C(O)=O)N%10	8.1_3
O=C%11COC1=CC=CC2=C1CCCC2.O=C%10CN%11.C[CH](C(O)=O)N%10	8.1_4
O=C%11COC1=CC=CC2=C1CCCC2.O=C%10CN%11.O=C(O)[CH](CC1=CC=CC=C1)N%10	8.1_5
O=C%11COC1=CC=CC2=C1CCCC2.O=C%10CN%11.CC[CH](C)[CH](C(O)=O)N%10	8.1_6
O=C%11COC1=CC=CC2=C1CCCC2.O=C%10CN%11.CSCC[CH](C(O)=O)N%10	8.1_7
O=C%11COC1=CC=CC2=C1CCCC2.O=C%10CN%11.OC(C=C1)=CC=C1C[CH](C(O)=O)N%10	8.1_8
O=C%11COC1=CC=CC2=C1CCCC2.O=C%10CN%11.O=C(O)[CH](CC1=C(F)C(F)=C(F)C(F)=C1 F)N%10	8.1_9
O=C%11COC1=CC=CC2=C1CCCC2.CC(C)[CH](C%10=O)N%11.O=C(O)CN%10	8.2_1
O=C%11COC1=CC=CC2=C1CCCC2.CC(C)[CH](C%10=O)N%11.CC(C)[CH](C(O)=O)N%10	8.2_2
O=C%11COC1=CC=CC2=C1CCCC2.CC(C)[CH](C%10=O)N%11.CC(C)C[CH](C(O)=O)N%10	8.2_3
O=C%11COC1=CC=CC2=C1CCCC2.CC(C)[CH](C%10=O)N%11.C[CH](C(O)=O)N%10	8.2_4
O=C%11COC1=CC=CC2=C1CCCC2.CC(C)[CH](C%10=O)N%11.O=C(O)[CH](CC1=CC=CC=C1) N%10	8.2_5
O=C%11COC1=CC=CC2=C1CCCC2.CC(C)[CH](C%10=O)N%11.CC[CH](C)[CH](C(O)=O)N%10	8.2_6
O=C%11COC1=CC=CC2=C1CCCC2.CC(C)[CH](C%10=O)N%11.CSCC[CH](C(O)=O)N%10	8.2_7
O=C%11COC1=CC=CC2=C1CCCC2.CC(C)[CH](C%10=O)N%11.OC(C=C1)=CC=C1C[CH](C(O) =O)N%10	8.2_8
O=C%11COC1=CC=CC2=C1CCCC2.CC(C)[CH](C%10=O)N%11.O=C(O)[CH](CC1=C(F)C(F)=C(F)C(F)=C1F)N%10	8.2_9
O=C%11COC1=CC=CC2=C1CCCC2.CC(C)C[CH](C%10=O)N%11.O=C(O)CN%10	8.3_1
O=C%11COC1=CC=CC2=C1CCCC2.CC(C)C[CH](C%10=O)N%11.CC(C)[CH](C(O)=O)N%10	8.3_2
O=C%11COC1=CC=CC2=C1CCCC2.CC(C)C[CH](C%10=O)N%11.CC(C)C[CH](C(O)=O)N%10	8.3_3
O=C%11COC1=CC=CC2=C1CCCC2.CC(C)C[CH](C%10=O)N%11.C[CH](C(O)=O)N%10	8.3_4
O=C%11COC1=CC=CC2=C1CCCC2.CC(C)C[CH](C%10=O)N%11.O=C(O)[CH](CC1=CC=CC=C 1)N%10	8.3_5
O=C%11COC1=CC=CC2=C1CCCC2.CC(C)C[CH](C%10=O)N%11.CC[CH](C)[CH](C(O)=O)N%1 0	8.3_6
O=C%11COC1=CC=CC2=C1CCCC2.CC(C)C[CH](C%10=O)N%11.CSCC[CH](C(O)=O)N%10	8.3_7
O=C%11COC1=CC=CC2=C1CCCC2.CC(C)C[CH](C%10=O)N%11.OC(C=C1)=CC=C1C[CH](C(O)=O)N%10	8.3_8
O=C%11COC1=CC=CC2=C1CCCC2.CC(C)C[CH](C%10=O)N%11.O=C(O)[CH](CC1=C(F)C(F)= C(F)C(F)=C1F)N%10	8.3_9
O=C%11COC1=CC=CC2=C1CCCC2.C[CH](C%10=O)N%11.O=C(O)CN%10	8.4_1
O=C%11COC1=CC=CC2=C1CCCC2.C[CH](C%10=O)N%11.CC(C)[CH](C(O)=O)N%10	8.4_2

O=C%11COC1=CC=CC2=C1CCCC2.C[CH](C%10=O)N%11.CC(C)C[CH](C(O)=O)N%10	8.4_3
O=C%11COC1=CC=CC2=C1CCCC2.C[CH](C%10=O)N%11.C[CH](C(O)=O)N%10	8.4_4
O=C%11COC1=CC=CC2=C1CCCC2.C[CH](C%10=O)N%11.O=C(O)[CH](CC1=CC=CC=C1)N%10	8.4_5
O=C%11COC1=CC=CC2=C1CCCC2.C[CH](C%10=O)N%11.CC[CH](C)[CH](C(O)=O)N%10	8.4_6
O=C%11COC1=CC=CC2=C1CCCC2.C[CH](C%10=O)N%11.CSCC[CH](C(O)=O)N%10	8.4_7
O=C%11COC1=CC=CC2=C1CCCC2.C[CH](C%10=O)N%11.OC(C=C1)=CC=C1C[CH](C(O)=O)N%10	8.4_8
O=C%11COC1=CC=CC2=C1CCCC2.C[CH](C%10=O)N%11.O=C(O)[CH](CC1=C(F)C(F)=C(F)C(F)=C1F)N%10	8.4_9
O=C%11COC1=CC=CC2=C1CCCC2.O=C%10[CH](CC1=CC=CC=C1)N%11.O=C(O)CN%10	8.5_1
O=C%11COC1=CC=CC2=C1CCCC2.O=C%10[CH](CC1=CC=CC=C1)N%11.CC(C)[CH](C(O)=O)N%10	8.5_2
O=C%11COC1=CC=CC2=C1CCCC2.O=C%10[CH](CC1=CC=CC=C1)N%11.CC(C)C[CH](C(O)=O)N%10	8.5_3
O=C%11COC1=CC=CC2=C1CCCC2.O=C%10[CH](CC1=CC=CC=C1)N%11.C[CH](C(O)=O)N%10	8.5_4
O=C%11COC1=CC=CC2=C1CCCC2.O=C%10[CH](CC1=CC=CC=C1)N%11.O=C(O)[CH](CC1=CC=CC=C1)N%10	8.5_5
O=C%11COC1=CC=CC2=C1CCCC2.O=C%10[CH](CC1=CC=CC=C1)N%11.CC[CH](C)[CH](C(O)=O)N%10	8.5_6
O=C%11COC1=CC=CC2=C1CCCC2.O=C%10[CH](CC1=CC=CC=C1)N%11.CSCC[CH](C(O)=O)N%10	8.5_7
O=C%11COC1=CC=CC2=C1CCCC2.O=C%10[CH](CC1=CC=CC=C1)N%11.OC(C=C1)=CC=C1C[CH](C(O)=O)N%10	8.5_8
O=C%11COC1=CC=CC2=C1CCCC2.O=C%10[CH](CC1=CC=CC=C1)N%11.O=C(O)[CH](CC1=C(F)C(F)=C(F)C(F)=C1F)N%10	8.5_9
O=C%11COC1=CC=CC2=C1CCCC2.CC[CH](C)[CH](C%10=O)N%11.O=C(O)CN%10	8.6_1
O=C%11COC1=CC=CC2=C1CCCC2.CC[CH](C)[CH](C%10=O)N%11.CC(C)[CH](C(O)=O)N%10	8.6_2
O=C%11COC1=CC=CC2=C1CCCC2.CC[CH](C)[CH](C%10=O)N%11.CC(C)C[CH](C(O)=O)N%10	8.6_3
O=C%11COC1=CC=CC2=C1CCCC2.CC[CH](C)[CH](C%10=O)N%11.C[CH](C(O)=O)N%10	8.6_4
O=C%11COC1=CC=CC2=C1CCCC2.CC[CH](C)[CH](C%10=O)N%11.O=C(O)[CH](CC1=CC=CC=C1)N%10	8.6_5
O=C%11COC1=CC=CC2=C1CCCC2.CC[CH](C)[CH](C%10=O)N%11.CC[CH](C)[CH](C(O)=O)N%10	8.6_6
O=C%11COC1=CC=CC2=C1CCCC2.CC[CH](C)[CH](C%10=O)N%11.CSCC[CH](C(O)=O)N%10	8.6_7
O=C%11COC1=CC=CC2=C1CCCC2.CC[CH](C)[CH](C%10=O)N%11.OC(C=C1)=CC=C1C[CH](C(O)=O)N%10	8.6_8
O=C%11COC1=CC=CC2=C1CCCC2.CC[CH](C)[CH](C%10=O)N%11.O=C(O)[CH](CC1=C(F)C(F)=C(F)C(F)=C1F)N%10	8.6_9
O=C%11COC1=CC=CC2=C1CCCC2.CSCC[CH](C%10=O)N%11.O=C(O)CN%10	8.7_1
O=C%11COC1=CC=CC2=C1CCCC2.CSCC[CH](C%10=O)N%11.CC(C)[CH](C(O)=O)N%10	8.7_2
O=C%11COC1=CC=CC2=C1CCCC2.CSCC[CH](C%10=O)N%11.CC(C)C[CH](C(O)=O)N%10	8.7_3
O=C%11COC1=CC=CC2=C1CCCC2.CSCC[CH](C%10=O)N%11.C[CH](C(O)=O)N%10	8.7_4
O=C%11COC1=CC=CC2=C1CCCC2.CSCC[CH](C%10=O)N%11.O=C(O)[CH](CC1=CC=CC=C1)N%10	8.7_5
O=C%11COC1=CC=CC2=C1CCCC2.CSCC[CH](C%10=O)N%11.CC[CH](C)[CH](C(O)=O)N%10	8.7_6

O=C%11COC1=CC=CC2=C1CCCC2.CSCC[CH](C%10=O)N%11.CSCC[CH](C(O)=O)N%10	8.7_7
O=C%11COC1=CC=CC2=C1CCCC2.CSCC[CH](C%10=O)N%11.OC(C=C1)=CC=C1C[CH](C(O)=O)N%10	8.7_8
O=C%11COC1=CC=CC2=C1CCCC2.CSCC[CH](C%10=O)N%11.O=C(O)[CH](CC1=C(F)C(F)=C(F)C(F)=C1F)N%10	8.7_9
O=C%11COC1=CC=CC2=C1CCCC2.OC(C=C1)=CC=C1C[CH](C%10=O)N%11.O=C(O)CN%10	8.8_1
O=C%11COC1=CC=CC2=C1CCCC2.OC(C=C1)=CC=C1C[CH](C%10=O)N%11.CC(C)[CH](C(O)=O)N%10	8.8_2
O=C%11COC1=CC=CC2=C1CCCC2.OC(C=C1)=CC=C1C[CH](C%10=O)N%11.CC(C)C[CH](C(O)=O)N%10	8.8_3
O=C%11COC1=CC=CC2=C1CCCC2.OC(C=C1)=CC=C1C[CH](C%10=O)N%11.C[CH](C(O)=O)N%10	8.8_4
O=C%11COC1=CC=CC2=C1CCCC2.OC(C=C1)=CC=C1C[CH](C%10=O)N%11.O=C(O)[CH](CC1=CC=CC=C1)N%10	8.8_5
O=C%11COC1=CC=CC2=C1CCCC2.OC(C=C1)=CC=C1C[CH](C%10=O)N%11.CC[CH](C)[CH](C(O)=O)N%10	8.8_6
O=C%11COC1=CC=CC2=C1CCCC2.OC(C=C1)=CC=C1C[CH](C%10=O)N%11.CSCC[CH](C(O)=O)N%10	8.8_7
O=C%11COC1=CC=CC2=C1CCCC2.OC(C=C1)=CC=C1C[CH](C%10=O)N%11.OC(C=C1)=CC=C1C[CH](C(O)=O)N%10	8.8_8
O=C%11COC1=CC=CC2=C1CCCC2.OC(C=C1)=CC=C1C[CH](C%10=O)N%11.O=C(O)[CH](CC1=C(F)C(F)=C(F)C(F)=C1F)N%10	8.8_9
O=C%10COC1=CC=CC2=C1CCCC2.O=C(O)CN%10	8.9_1
O=C%10COC1=CC=CC2=C1CCCC2.CC(C)[CH](C(O)=O)N%10	8.9_2
O=C%10COC1=CC=CC2=C1CCCC2.CC(C)C[CH](C(O)=O)N%10	8.9_3
O=C%10COC1=CC=CC2=C1CCCC2.C[CH](C(O)=O)N%10	8.9_4
O=C%10COC1=CC=CC2=C1CCCC2.O=C(O)[CH](CC1=CC=CC=C1)N%10	8.9_5
O=C%10COC1=CC=CC2=C1CCCC2.CC[CH](C)[CH](C(O)=O)N%10	8.9_6
O=C%10COC1=CC=CC2=C1CCCC2.CSCC[CH](C(O)=O)N%10	8.9_7
O=C%10COC1=CC=CC2=C1CCCC2.OC(C=C1)=CC=C1C[CH](C(O)=O)N%10	8.9_8
O=C%10COC1=CC=CC2=C1CCCC2.O=C(O)[CH](CC1=C(F)C(F)=C(F)C(F)=C1F)N%10	8.9_9
C%11(COC1=CC=CC2=CC(Br)=CC=C21)=O.O=C%10CN%11.O=C(O)CN%10	9.1_1
C%11(COC1=CC=CC2=CC(Br)=CC=C21)=O.O=C%10CN%11.CC(C)[CH](C(O)=O)N%10	9.1_2
C%11(COC1=CC=CC2=CC(Br)=CC=C21)=O.O=C%10CN%11.CC(C)C[CH](C(O)=O)N%10	9.1_3
C%11(COC1=CC=CC2=CC(Br)=CC=C21)=O.O=C%10CN%11.C[CH](C(O)=O)N%10	9.1_4
C%11(COC1=CC=CC2=CC(Br)=CC=C21)=O.O=C%10CN%11.O=C(O)[CH](CC1=CC=CC=C1)N%10	9.1_5
C%11(COC1=CC=CC2=CC(Br)=CC=C21)=O.O=C%10CN%11.CC[CH](C)[CH](C(O)=O)N%10	9.1_6
C%11(COC1=CC=CC2=CC(Br)=CC=C21)=O.O=C%10CN%11.CSCC[CH](C(O)=O)N%10	9.1_7
C%11(COC1=CC=CC2=CC(Br)=CC=C21)=O.O=C%10CN%11.OC(C=C1)=CC=C1C[CH](C(O)=O)N%10	9.1_8
C%11(COC1=CC=CC2=CC(Br)=CC=C21)=O.O=C%10CN%11.O=C(O)[CH](CC1=C(F)C(F)=C(F)C(F)=C1F)N%10	9.1_9
C%11(COC1=CC=CC2=CC(Br)=CC=C21)=O.CC(C)[CH](C%10=O)N%11.O=C(O)CN%10	9.2_1
C%11(COC1=CC=CC2=CC(Br)=CC=C21)=O.CC(C)[CH](C%10=O)N%11.CC(C)[CH](C(O)=O)N%10	9.2_2
C%11(COC1=CC=CC2=CC(Br)=CC=C21)=O.CC(C)[CH](C%10=O)N%11.CC(C)C[CH](C(O)=O)	9.2_3

N%10	
C%11(COC1=CC=CC2=CC(Br)=CC=C21)=O.CC(C)[CH](C%10=O)N%11.C[CH](C(O)=O)N%10	9.2_4
C%11(COC1=CC=CC2=CC(Br)=CC=C21)=O.CC(C)[CH](C%10=O)N%11.O=C(O)[CH](CC1=CC=CC=C1)N%10	9.2_5
C%11(COC1=CC=CC2=CC(Br)=CC=C21)=O.CC(C)[CH](C%10=O)N%11.CC[CH](C)[CH](C(O)=O)N%10	9.2_6
C%11(COC1=CC=CC2=CC(Br)=CC=C21)=O.CC(C)[CH](C%10=O)N%11.CSCC[CH](C(O)=O)N%10	9.2_7
C%11(COC1=CC=CC2=CC(Br)=CC=C21)=O.CC(C)[CH](C%10=O)N%11.OC(C=C1)=CC=C1C[CH](C(O)=O)N%10	9.2_8
C%11(COC1=CC=CC2=CC(Br)=CC=C21)=O.CC(C)[CH](C%10=O)N%11.O=C(O)[CH](CC1=C(F)C(F)=C(F)C(F)=C1F)N%10	9.2_9
C%11(COC1=CC=CC2=CC(Br)=CC=C21)=O.CC(C)C[CH](C%10=O)N%11.O=C(O)CN%10	9.3_1
C%11(COC1=CC=CC2=CC(Br)=CC=C21)=O.CC(C)C[CH](C%10=O)N%11.CC(C)[CH](C(O)=O)N%10	9.3_2
C%11(COC1=CC=CC2=CC(Br)=CC=C21)=O.CC(C)C[CH](C%10=O)N%11.CC(C)C[CH](C(O)=O)N%10	9.3_3
C%11(COC1=CC=CC2=CC(Br)=CC=C21)=O.CC(C)C[CH](C%10=O)N%11.C[CH](C(O)=O)N%10	9.3_4
C%11(COC1=CC=CC2=CC(Br)=CC=C21)=O.CC(C)C[CH](C%10=O)N%11.O=C(O)[CH](CC1=CC=CC=C1)N%10	9.3_5
C%11(COC1=CC=CC2=CC(Br)=CC=C21)=O.CC(C)C[CH](C%10=O)N%11.CC[CH](C)[CH](C(O)=O)N%10	9.3_6
C%11(COC1=CC=CC2=CC(Br)=CC=C21)=O.CC(C)C[CH](C%10=O)N%11.CSCC[CH](C(O)=O)N%10	9.3_7
C%11(COC1=CC=CC2=CC(Br)=CC=C21)=O.CC(C)C[CH](C%10=O)N%11.OC(C=C1)=CC=C1C[CH](C(O)=O)N%10	9.3_8
C%11(COC1=CC=CC2=CC(Br)=CC=C21)=O.CC(C)C[CH](C%10=O)N%11.O=C(O)[CH](CC1=C(F)C(F)=C(F)C(F)=C1F)N%10	9.3_9
C%11(COC1=CC=CC2=CC(Br)=CC=C21)=O.C[CH](C%10=O)N%11.O=C(O)CN%10	9.4_1
C%11(COC1=CC=CC2=CC(Br)=CC=C21)=O.C[CH](C%10=O)N%11.CC(C)[CH](C(O)=O)N%10	9.4_2
C%11(COC1=CC=CC2=CC(Br)=CC=C21)=O.C[CH](C%10=O)N%11.CC(C)C[CH](C(O)=O)N%10	9.4_3
C%11(COC1=CC=CC2=CC(Br)=CC=C21)=O.C[CH](C%10=O)N%11.C[CH](C(O)=O)N%10	9.4_4
C%11(COC1=CC=CC2=CC(Br)=CC=C21)=O.C[CH](C%10=O)N%11.O=C(O)[CH](CC1=CC=CC=C1)N%10	9.4_5
C%11(COC1=CC=CC2=CC(Br)=CC=C21)=O.C[CH](C%10=O)N%11.CC[CH](C)[CH](C(O)=O)N%10	9.4_6
C%11(COC1=CC=CC2=CC(Br)=CC=C21)=O.C[CH](C%10=O)N%11.CSCC[CH](C(O)=O)N%10	9.4_7
C%11(COC1=CC=CC2=CC(Br)=CC=C21)=O.C[CH](C%10=O)N%11.OC(C=C1)=CC=C1C[CH](C(O)=O)N%10	9.4_8
C%11(COC1=CC=CC2=CC(Br)=CC=C21)=O.C[CH](C%10=O)N%11.O=C(O)[CH](CC1=C(F)C(F)=C(F)C(F)=C1F)N%10	9.4_9
C%11(COC1=CC=CC2=CC(Br)=CC=C21)=O.O=C%10[CH](CC1=CC=CC=C1)N%11.O=C(O)CN%10	9.5_1
C%11(COC1=CC=CC2=CC(Br)=CC=C21)=O.O=C%10[CH](CC1=CC=CC=C1)N%11.CC(C)[CH](C(O)=O)N%10	9.5_2
C%11(COC1=CC=CC2=CC(Br)=CC=C21)=O.O=C%10[CH](CC1=CC=CC=C1)N%11.CC(C)C[CH]	9.5_3

(C(O)=O)N%10	
C%11(COC1=CC=CC2=CC(Br)=CC=C21)=O.O=C%10[CH](CC1=CC=CC=C1)N%11.C[CH](C(O)=O)N%10	9.5_4
C%11(COC1=CC=CC2=CC(Br)=CC=C21)=O.O=C%10[CH](CC1=CC=CC=C1)N%11.O=C(O)[CH](CC1=CC=CC=C1)N%10	9.5_5
C%11(COC1=CC=CC2=CC(Br)=CC=C21)=O.O=C%10[CH](CC1=CC=CC=C1)N%11.CC[CH](C)[CH](C(O)=O)N%10	9.5_6
C%11(COC1=CC=CC2=CC(Br)=CC=C21)=O.O=C%10[CH](CC1=CC=CC=C1)N%11.CSCC[CH](C(O)=O)N%10	9.5_7
C%11(COC1=CC=CC2=CC(Br)=CC=C21)=O.O=C%10[CH](CC1=CC=CC=C1)N%11.OC(C=C1)=CC=C1C[CH](C(O)=O)N%10	9.5_8
C%11(COC1=CC=CC2=CC(Br)=CC=C21)=O.O=C%10[CH](CC1=CC=CC=C1)N%11.O=C(O)[CH](CC1=C(F)C(F)=C(F)C(F)=C1F)N%10	9.5_9
C%11(COC1=CC=CC2=CC(Br)=CC=C21)=O.CC[CH](C)[CH](C%10=O)N%11.O=C(O)CN%10	9.6_1
C%11(COC1=CC=CC2=CC(Br)=CC=C21)=O.CC[CH](C)[CH](C%10=O)N%11.CC(C)[CH](C(O)=O)N%10	9.6_2
C%11(COC1=CC=CC2=CC(Br)=CC=C21)=O.CC[CH](C)[CH](C%10=O)N%11.CC(C)C[CH](C(O)=O)N%10	9.6_3
C%11(COC1=CC=CC2=CC(Br)=CC=C21)=O.CC[CH](C)[CH](C%10=O)N%11.C[CH](C(O)=O)N%10	9.6_4
C%11(COC1=CC=CC2=CC(Br)=CC=C21)=O.CC[CH](C)[CH](C%10=O)N%11.O=C(O)[CH](CC1=CC=CC=C1)N%10	9.6_5
C%11(COC1=CC=CC2=CC(Br)=CC=C21)=O.CC[CH](C)[CH](C%10=O)N%11.CC[CH](C)[CH](C(O)=O)N%10	9.6_6
C%11(COC1=CC=CC2=CC(Br)=CC=C21)=O.CC[CH](C)[CH](C%10=O)N%11.CSCC[CH](C(O)=O)N%10	9.6_7
C%11(COC1=CC=CC2=CC(Br)=CC=C21)=O.CC[CH](C)[CH](C%10=O)N%11.OC(C=C1)=CC=C1C[CH](C(O)=O)N%10	9.6_8
C%11(COC1=CC=CC2=CC(Br)=CC=C21)=O.CC[CH](C)[CH](C%10=O)N%11.O=C(O)[CH](CC1=C(F)C(F)=C(F)C(F)=C1F)N%10	9.6_9
C%11(COC1=CC=CC2=CC(Br)=CC=C21)=O.CSCC[CH](C%10=O)N%11.O=C(O)CN%10	9.7_1
C%11(COC1=CC=CC2=CC(Br)=CC=C21)=O.CSCC[CH](C%10=O)N%11.CC(C)[CH](C(O)=O)N%10	9.7_2
C%11(COC1=CC=CC2=CC(Br)=CC=C21)=O.CSCC[CH](C%10=O)N%11.CC(C)C[CH](C(O)=O)N%10	9.7_3
C%11(COC1=CC=CC2=CC(Br)=CC=C21)=O.CSCC[CH](C%10=O)N%11.C[CH](C(O)=O)N%10	9.7_4
C%11(COC1=CC=CC2=CC(Br)=CC=C21)=O.CSCC[CH](C%10=O)N%11.O=C(O)[CH](CC1=CC=CC=C1)N%10	9.7_5
C%11(COC1=CC=CC2=CC(Br)=CC=C21)=O.CSCC[CH](C%10=O)N%11.CC[CH](C)[CH](C(O)=O)N%10	9.7_6
C%11(COC1=CC=CC2=CC(Br)=CC=C21)=O.CSCC[CH](C%10=O)N%11.CSCC[CH](C(O)=O)N%10	9.7_7
C%11(COC1=CC=CC2=CC(Br)=CC=C21)=O.CSCC[CH](C%10=O)N%11.OC(C=C1)=CC=C1C[CH](C(O)=O)N%10	9.7_8
C%11(COC1=CC=CC2=CC(Br)=CC=C21)=O.CSCC[CH](C%10=O)N%11.O=C(O)[CH](CC1=C(F)C(F)=C(F)C(F)=C1F)N%10	9.7_9
C%11(COC1=CC=CC2=CC(Br)=CC=C21)=O.OC(C=C1)=CC=C1C[CH](C%10=O)N%11.O=C(O)CN%10	9.8_1
C%11(COC1=CC=CC2=CC(Br)=CC=C21)=O.OC(C=C1)=CC=C1C[CH](C%10=O)N%11.CC(C)[C	9.8_2

H](C(O)=O)N%10	
C%11(COC1=CC=CC2=CC(Br)=CC=C21)=O.OC(C=C1)=CC=C1C[CH](C%10=O)N%11.CC(C)C[CH](C(O)=O)N%10	9.8_3
C%11(COC1=CC=CC2=CC(Br)=CC=C21)=O.OC(C=C1)=CC=C1C[CH](C%10=O)N%11.C[CH](C(O)=O)N%10	9.8_4
C%11(COC1=CC=CC2=CC(Br)=CC=C21)=O.OC(C=C1)=CC=C1C[CH](C%10=O)N%11.O=C(O)[CH](CC1=CC=CC=C1)N%10	9.8_5
C%11(COC1=CC=CC2=CC(Br)=CC=C21)=O.OC(C=C1)=CC=C1C[CH](C%10=O)N%11.CC[CH](C)[CH](C(O)=O)N%10	9.8_6
C%11(COC1=CC=CC2=CC(Br)=CC=C21)=O.OC(C=C1)=CC=C1C[CH](C%10=O)N%11.CSCC[CH](C(O)=O)N%10	9.8_7
C%11(COC1=CC=CC2=CC(Br)=CC=C21)=O.OC(C=C1)=CC=C1C[CH](C%10=O)N%11.OC(C=C1)=CC=C1C[CH](C(O)=O)N%10	9.8_8
C%11(COC1=CC=CC2=CC(Br)=CC=C21)=O.OC(C=C1)=CC=C1C[CH](C%10=O)N%11.O=C(O)[CH](CC1=C(F)C(F)=C(F)C(F)=C1F)N%10	9.8_9
C%10(COC1=CC=CC2=CC(Br)=CC=C21)=O.O=C(O)CN%10	9.9_1
C%10(COC1=CC=CC2=CC(Br)=CC=C21)=O.CC(C)[CH](C(O)=O)N%10	9.9_2
C%10(COC1=CC=CC2=CC(Br)=CC=C21)=O.CC(C)C[CH](C(O)=O)N%10	9.9_3
C%10(COC1=CC=CC2=CC(Br)=CC=C21)=O.C[CH](C(O)=O)N%10	9.9_4
C%10(COC1=CC=CC2=CC(Br)=CC=C21)=O.O=C(O)[CH](CC1=CC=CC=C1)N%10	9.9_5
C%10(COC1=CC=CC2=CC(Br)=CC=C21)=O.CC[CH](C)[CH](C(O)=O)N%10	9.9_6
C%10(COC1=CC=CC2=CC(Br)=CC=C21)=O.CSCC[CH](C(O)=O)N%10	9.9_7
C%10(COC1=CC=CC2=CC(Br)=CC=C21)=O.OC(C=C1)=CC=C1C[CH](C(O)=O)N%10	9.9_8
C%10(COC1=CC=CC2=CC(Br)=CC=C21)=O.O=C(O)[CH](CC1=C(F)C(F)=C(F)C(F)=C1F)N%10	9.9_9
C%11(COC1=CC=CC2=CC=CC=C21)=O.O=C%10CN%11.O=C(O)CN%10	10.1_1
C%11(COC1=CC=CC2=CC=CC=C21)=O.O=C%10CN%11.CC(C)[CH](C(O)=O)N%10	10.1_2
C%11(COC1=CC=CC2=CC=CC=C21)=O.O=C%10CN%11.CC(C)C[CH](C(O)=O)N%10	10.1_3
C%11(COC1=CC=CC2=CC=CC=C21)=O.O=C%10CN%11.C[CH](C(O)=O)N%10	10.1_4
C%11(COC1=CC=CC2=CC=CC=C21)=O.O=C%10CN%11.O=C(O)[CH](CC1=CC=CC=C1)N%10	10.1_5
C%11(COC1=CC=CC2=CC=CC=C21)=O.O=C%10CN%11.CC[CH](C)[CH](C(O)=O)N%10	10.1_6
C%11(COC1=CC=CC2=CC=CC=C21)=O.O=C%10CN%11.CSCC[CH](C(O)=O)N%10	10.1_7
C%11(COC1=CC=CC2=CC=CC=C21)=O.O=C%10CN%11.OC(C=C1)=CC=C1C[CH](C(O)=O)N%10	10.1_8
C%11(COC1=CC=CC2=CC=CC=C21)=O.O=C%10CN%11.O=C(O)[CH](CC1=C(F)C(F)=C(F)C(F)=C1F)N%10	10.1_9
C%11(COC1=CC=CC2=CC=CC=C21)=O.CC(C)[CH](C%10=O)N%11.O=C(O)CN%10	10.2_1
C%11(COC1=CC=CC2=CC=CC=C21)=O.CC(C)[CH](C%10=O)N%11.CC(C)[CH](C(O)=O)N%10	10.2_2
C%11(COC1=CC=CC2=CC=CC=C21)=O.CC(C)[CH](C%10=O)N%11.CC(C)C[CH](C(O)=O)N%10	10.2_3
C%11(COC1=CC=CC2=CC=CC=C21)=O.CC(C)[CH](C%10=O)N%11.C[CH](C(O)=O)N%10	10.2_4
C%11(COC1=CC=CC2=CC=CC=C21)=O.CC(C)[CH](C%10=O)N%11.O=C(O)[CH](CC1=CC=CC=C1)N%10	10.2_5
C%11(COC1=CC=CC2=CC=CC=C21)=O.CC(C)[CH](C%10=O)N%11.CC[CH](C)[CH](C(O)=O)N%10	10.2_6
C%11(COC1=CC=CC2=CC=CC=C21)=O.CC(C)[CH](C%10=O)N%11.CSCC[CH](C(O)=O)N%10	10.2_7
C%11(COC1=CC=CC2=CC=CC=C21)=O.CC(C)[CH](C%10=O)N%11.OC(C=C1)=CC=C1C[CH](C(O)=O)N%10	10.2_8

C%11(COC1=CC=CC2=CC=CC=C21)=O.CC(C)[CH](C%10=O)N%11.O=C(O)[CH](CC1=C(F)C(F)=C(F)C(F)=C1F)N%10	10.2_9
C%11(COC1=CC=CC2=CC=CC=C21)=O.CC(C)C[CH](C%10=O)N%11.O=C(O)CN%10	10.3_1
C%11(COC1=CC=CC2=CC=CC=C21)=O.CC(C)C[CH](C%10=O)N%11.CC(C)[CH](C(O)=O)N%10	10.3_2
C%11(COC1=CC=CC2=CC=CC=C21)=O.CC(C)C[CH](C%10=O)N%11.CC(C)C[CH](C(O)=O)N%10	10.3_3
C%11(COC1=CC=CC2=CC=CC=C21)=O.CC(C)C[CH](C%10=O)N%11.C[CH](C(O)=O)N%10	10.3_4
C%11(COC1=CC=CC2=CC=CC=C21)=O.CC(C)C[CH](C%10=O)N%11.O=C(O)[CH](CC1=CC=CC=C1)N%10	10.3_5
C%11(COC1=CC=CC2=CC=CC=C21)=O.CC(C)C[CH](C%10=O)N%11.CC[CH](C)[CH](C(O)=O)N%10	10.3_6
C%11(COC1=CC=CC2=CC=CC=C21)=O.CC(C)C[CH](C%10=O)N%11.CSCC[CH](C(O)=O)N%10	10.3_7
C%11(COC1=CC=CC2=CC=CC=C21)=O.CC(C)C[CH](C%10=O)N%11.OC(C=C1)=CC=C1C[CH](C(O)=O)N%10	10.3_8
C%11(COC1=CC=CC2=CC=CC=C21)=O.CC(C)C[CH](C%10=O)N%11.O=C(O)[CH](CC1=C(F)C(F)=C(F)C(F)=C1F)N%10	10.3_9
C%11(COC1=CC=CC2=CC=CC=C21)=O.C[CH](C%10=O)N%11.O=C(O)CN%10	10.4_1
C%11(COC1=CC=CC2=CC=CC=C21)=O.C[CH](C%10=O)N%11.CC(C)[CH](C(O)=O)N%10	10.4_2
C%11(COC1=CC=CC2=CC=CC=C21)=O.C[CH](C%10=O)N%11.CC(C)C[CH](C(O)=O)N%10	10.4_3
C%11(COC1=CC=CC2=CC=CC=C21)=O.C[CH](C%10=O)N%11.C[CH](C(O)=O)N%10	10.4_4
C%11(COC1=CC=CC2=CC=CC=C21)=O.C[CH](C%10=O)N%11.O=C(O)[CH](CC1=CC=CC=C1)N%10	10.4_5
C%11(COC1=CC=CC2=CC=CC=C21)=O.C[CH](C%10=O)N%11.CC[CH](C)[CH](C(O)=O)N%10	10.4_6
C%11(COC1=CC=CC2=CC=CC=C21)=O.C[CH](C%10=O)N%11.CSCC[CH](C(O)=O)N%10	10.4_7
C%11(COC1=CC=CC2=CC=CC=C21)=O.C[CH](C%10=O)N%11.OC(C=C1)=CC=C1C[CH](C(O)=O)N%10	10.4_8
C%11(COC1=CC=CC2=CC=CC=C21)=O.C[CH](C%10=O)N%11.O=C(O)[CH](CC1=C(F)C(F)=C(F)C(F)=C1F)N%10	10.4_9
C%11(COC1=CC=CC2=CC=CC=C21)=O.O=C%10[CH](CC1=CC=CC=C1)N%11.O=C(O)CN%10	10.5_1
C%11(COC1=CC=CC2=CC=CC=C21)=O.O=C%10[CH](CC1=CC=CC=C1)N%11.CC(C)[CH](C(O)=O)N%10	10.5_2
C%11(COC1=CC=CC2=CC=CC=C21)=O.O=C%10[CH](CC1=CC=CC=C1)N%11.CC(C)C[CH](C(O)=O)N%10	10.5_3
C%11(COC1=CC=CC2=CC=CC=C21)=O.O=C%10[CH](CC1=CC=CC=C1)N%11.C[CH](C(O)=O)N%10	10.5_4
C%11(COC1=CC=CC2=CC=CC=C21)=O.O=C%10[CH](CC1=CC=CC=C1)N%11.O=C(O)[CH](CC1=CC=CC=C1)N%10	10.5_5
C%11(COC1=CC=CC2=CC=CC=C21)=O.O=C%10[CH](CC1=CC=CC=C1)N%11.CC[CH](C)[CH](C(O)=O)N%10	10.5_6
C%11(COC1=CC=CC2=CC=CC=C21)=O.O=C%10[CH](CC1=CC=CC=C1)N%11.CSCC[CH](C(O)=O)N%10	10.5_7
C%11(COC1=CC=CC2=CC=CC=C21)=O.O=C%10[CH](CC1=CC=CC=C1)N%11.OC(C=C1)=CC=C1C[CH](C(O)=O)N%10	10.5_8
C%11(COC1=CC=CC2=CC=CC=C21)=O.O=C%10[CH](CC1=CC=CC=C1)N%11.O=C(O)[CH](CC1=C(F)C(F)=C(F)C(F)=C1F)N%10	10.5_9
C%11(COC1=CC=CC2=CC=CC=C21)=O.CC[CH](C)[CH](C%10=O)N%11.O=C(O)CN%10	10.6_1

C%11(COC1=CC=CC2=CC=CC=C21)=O.CC[CH](C)[CH](C%10=O)N%11.CC(C)[CH](C(O)=O)N%10	10.6_2
C%11(COC1=CC=CC2=CC=CC=C21)=O.CC[CH](C)[CH](C%10=O)N%11.CC(C)C[CH](C(O)=O)N%10	10.6_3
C%11(COC1=CC=CC2=CC=CC=C21)=O.CC[CH](C)[CH](C%10=O)N%11.C[CH](C(O)=O)N%10	10.6_4
C%11(COC1=CC=CC2=CC=CC=C21)=O.CC[CH](C)[CH](C%10=O)N%11.O=C(O)[CH](CC1=CC=CC=C1)N%10	10.6_5
C%11(COC1=CC=CC2=CC=CC=C21)=O.CC[CH](C)[CH](C%10=O)N%11.CC[CH](C)[CH](C(O)=O)N%10	10.6_6
C%11(COC1=CC=CC2=CC=CC=C21)=O.CC[CH](C)[CH](C%10=O)N%11.CSCC[CH](C(O)=O)N%10	10.6_7
C%11(COC1=CC=CC2=CC=CC=C21)=O.CC[CH](C)[CH](C%10=O)N%11.OC(C=C1)=CC=C1C[CH](C(O)=O)N%10	10.6_8
C%11(COC1=CC=CC2=CC=CC=C21)=O.CC[CH](C)[CH](C%10=O)N%11.O=C(O)[CH](CC1=C(F)C(F)=C(F)C(F)=C1F)N%10	10.6_9
C%11(COC1=CC=CC2=CC=CC=C21)=O.CSCC[CH](C%10=O)N%11.O=C(O)CN%10	10.7_1
C%11(COC1=CC=CC2=CC=CC=C21)=O.CSCC[CH](C%10=O)N%11.CC(C)[CH](C(O)=O)N%10	10.7_2
C%11(COC1=CC=CC2=CC=CC=C21)=O.CSCC[CH](C%10=O)N%11.CC(C)C[CH](C(O)=O)N%10	10.7_3
C%11(COC1=CC=CC2=CC=CC=C21)=O.CSCC[CH](C%10=O)N%11.C[CH](C(O)=O)N%10	10.7_4
C%11(COC1=CC=CC2=CC=CC=C21)=O.CSCC[CH](C%10=O)N%11.O=C(O)[CH](CC1=CC=CC=C1)N%10	10.7_5
C%11(COC1=CC=CC2=CC=CC=C21)=O.CSCC[CH](C%10=O)N%11.CC[CH](C)[CH](C(O)=O)N%10	10.7_6
C%11(COC1=CC=CC2=CC=CC=C21)=O.CSCC[CH](C%10=O)N%11.CSCC[CH](C(O)=O)N%10	10.7_7
C%11(COC1=CC=CC2=CC=CC=C21)=O.CSCC[CH](C%10=O)N%11.OC(C=C1)=CC=C1C[CH](C(O)=O)N%10	10.7_8
C%11(COC1=CC=CC2=CC=CC=C21)=O.CSCC[CH](C%10=O)N%11.O=C(O)[CH](CC1=C(F)C(F)=C(F)C(F)=C1F)N%10	10.7_9
C%11(COC1=CC=CC2=CC=CC=C21)=O.OC(C=C1)=CC=C1C[CH](C%10=O)N%11.O=C(O)CN%10	10.8_1
C%11(COC1=CC=CC2=CC=CC=C21)=O.OC(C=C1)=CC=C1C[CH](C%10=O)N%11.CC(C)[CH](C(O)=O)N%10	10.8_2
C%11(COC1=CC=CC2=CC=CC=C21)=O.OC(C=C1)=CC=C1C[CH](C%10=O)N%11.CC(C)C[CH](C(O)=O)N%10	10.8_3
C%11(COC1=CC=CC2=CC=CC=C21)=O.OC(C=C1)=CC=C1C[CH](C%10=O)N%11.C[CH](C(O)=O)N%10	10.8_4
C%11(COC1=CC=CC2=CC=CC=C21)=O.OC(C=C1)=CC=C1C[CH](C%10=O)N%11.O=C(O)[CH](CC1=CC=CC=C1)N%10	10.8_5
C%11(COC1=CC=CC2=CC=CC=C21)=O.OC(C=C1)=CC=C1C[CH](C%10=O)N%11.CC[CH](C)[CH](C(O)=O)N%10	10.8_6
C%11(COC1=CC=CC2=CC=CC=C21)=O.OC(C=C1)=CC=C1C[CH](C%10=O)N%11.CSCC[CH](C(O)=O)N%10	10.8_7
C%11(COC1=CC=CC2=CC=CC=C21)=O.OC(C=C1)=CC=C1C[CH](C%10=O)N%11.OC(C=C1)=CC=C1C[CH](C(O)=O)N%10	10.8_8
C%11(COC1=CC=CC2=CC=CC=C21)=O.OC(C=C1)=CC=C1C[CH](C%10=O)N%11.O=C(O)[CH](CC1=C(F)C(F)=C(F)C(F)=C1F)N%10	10.8_9
C%10(COC1=CC=CC2=CC=CC=C21)=O.O=C(O)CN%10	10.9_1

C%10(COC1=CC=CC2=CC=CC=C21)=O.CC(C)[CH](C(O)=O)N%10	10.9_2
C%10(COC1=CC=CC2=CC=CC=C21)=O.CC(C)C[CH](C(O)=O)N%10	10.9_3
C%10(COC1=CC=CC2=CC=CC=C21)=O.C[CH](C(O)=O)N%10	10.9_4
C%10(COC1=CC=CC2=CC=CC=C21)=O.O=C(O)[CH](CC1=CC=CC=C1)N%10	10.9_5
C%10(COC1=CC=CC2=CC=CC=C21)=O.CC[CH](C)[CH](C(O)=O)N%10	10.9_6
C%10(COC1=CC=CC2=CC=CC=C21)=O.CSCC[CH](C(O)=O)N%10	10.9_7
C%10(COC1=CC=CC2=CC=CC=C21)=O.OC(C=C1)=CC=C1C[CH](C(O)=O)N%10	10.9_8
C%10(COC1=CC=CC2=CC=CC=C21)=O.O=C(O)[CH](CC1=C(F)C(F)=C(F)C(F)=C1F)N%10	10.9_9
C%11(COC1=CC=CC2=CC(C#N)=CC=C21)=O.O=C%10CN%11.O=C(O)CN%10	11.1_1
C%11(COC1=CC=CC2=CC(C#N)=CC=C21)=O.O=C%10CN%11.CC(C)[CH](C(O)=O)N%10	11.1_2
C%11(COC1=CC=CC2=CC(C#N)=CC=C21)=O.O=C%10CN%11.CC(C)C[CH](C(O)=O)N%10	11.1_3
C%11(COC1=CC=CC2=CC(C#N)=CC=C21)=O.O=C%10CN%11.C[CH](C(O)=O)N%10	11.1_4
C%11(COC1=CC=CC2=CC(C#N)=CC=C21)=O.O=C%10CN%11.O=C(O)[CH](CC1=CC=CC=C1)<br>N%10	11.1_5
C%11(COC1=CC=CC2=CC(C#N)=CC=C21)=O.O=C%10CN%11.CC[CH](C)[CH](C(O)=O)N%10	11.1_6
C%11(COC1=CC=CC2=CC(C#N)=CC=C21)=O.O=C%10CN%11.CSCC[CH](C(O)=O)N%10	11.1_7
C%11(COC1=CC=CC2=CC(C#N)=CC=C21)=O.O=C%10CN%11.OC(C=C1)=CC=C1C[CH](C(O)=<br>O)N%10	11.1_8
C%11(COC1=CC=CC2=CC(C#N)=CC=C21)=O.O=C%10CN%11.O=C(O)[CH](CC1=C(F)C(F)=C(<br>F)C(F)=C1F)N%10	11.1_9
C%11(COC1=CC=CC2=CC(C#N)=CC=C21)=O.CC(C)[CH](C%10=O)N%11.O=C(O)CN%10	11.2_1
C%11(COC1=CC=CC2=CC(C#N)=CC=C21)=O.CC(C)[CH](C%10=O)N%11.CC(C)[CH](C(O)=O)<br>N%10	11.2_2
C%11(COC1=CC=CC2=CC(C#N)=CC=C21)=O.CC(C)[CH](C%10=O)N%11.CC(C)C[CH](C(O)=O<br>)N%10	11.2_3
C%11(COC1=CC=CC2=CC(C#N)=CC=C21)=O.CC(C)[CH](C%10=O)N%11.C[CH](C(O)=O)N%1<br>0	11.2_4
C%11(COC1=CC=CC2=CC(C#N)=CC=C21)=O.CC(C)[CH](C%10=O)N%11.O=C(O)[CH](CC1=C<br>C=CC=C1)N%10	11.2_5
C%11(COC1=CC=CC2=CC(C#N)=CC=C21)=O.CC(C)[CH](C%10=O)N%11.CC[CH](C)[CH](C(O)<br>=O)N%10	11.2_6
C%11(COC1=CC=CC2=CC(C#N)=CC=C21)=O.CC(C)[CH](C%10=O)N%11.CSCC[CH](C(O)=O)<br>N%10	11.2_7
C%11(COC1=CC=CC2=CC(C#N)=CC=C21)=O.CC(C)[CH](C%10=O)N%11.OC(C=C1)=CC=C1C[<br>CH](C(O)=O)N%10	11.2_8
C%11(COC1=CC=CC2=CC(C#N)=CC=C21)=O.CC(C)[CH](C%10=O)N%11.O=C(O)[CH](CC1=C<br>(F)C(F)=C(F)C(F)=C1F)N%10	11.2_9
C%11(COC1=CC=CC2=CC(C#N)=CC=C21)=O.CC(C)C[CH](C%10=O)N%11.O=C(O)CN%10	11.3_1
C%11(COC1=CC=CC2=CC(C#N)=CC=C21)=O.CC(C)C[CH](C%10=O)N%11.CC(C)[CH](C(O)=O<br>)N%10	11.3_2
C%11(COC1=CC=CC2=CC(C#N)=CC=C21)=O.CC(C)C[CH](C%10=O)N%11.CC(C)C[CH](C(O)=<br>O)N%10	11.3_3
C%11(COC1=CC=CC2=CC(C#N)=CC=C21)=O.CC(C)C[CH](C%10=O)N%11.C[CH](C(O)=O)N%<br>10	11.3_4
C%11(COC1=CC=CC2=CC(C#N)=CC=C21)=O.CC(C)C[CH](C%10=O)N%11.O=C(O)[CH](CC1=<br>CC=CC=C1)N%10	11.3_5
C%11(COC1=CC=CC2=CC(C#N)=CC=C21)=O.CC(C)C[CH](C%10=O)N%11.CC[CH](C)[CH](C(	11.3_6

O)=O)N%10	
C%11(COC1=CC=CC2=CC(C#N)=CC=C21)=O.CC(C)C[CH](C%10=O)N%11.CSCC[CH](C(O)=O)N%10	11.3_7
C%11(COC1=CC=CC2=CC(C#N)=CC=C21)=O.CC(C)C[CH](C%10=O)N%11.OC(C=C1)=CC=C1C[CH](C(O)=O)N%10	11.3_8
C%11(COC1=CC=CC2=CC(C#N)=CC=C21)=O.CC(C)C[CH](C%10=O)N%11.O=C(O)[CH](CC1=C(F)C(F)=C(F)C(F)=C1F)N%10	11.3_9
C%11(COC1=CC=CC2=CC(C#N)=CC=C21)=O.C[CH](C%10=O)N%11.O=C(O)CN%10	11.4_1
C%11(COC1=CC=CC2=CC(C#N)=CC=C21)=O.C[CH](C%10=O)N%11.CC(C)[CH](C(O)=O)N%10	11.4_2
C%11(COC1=CC=CC2=CC(C#N)=CC=C21)=O.C[CH](C%10=O)N%11.CC(C)C[CH](C(O)=O)N%10	11.4_3
C%11(COC1=CC=CC2=CC(C#N)=CC=C21)=O.C[CH](C%10=O)N%11.C[CH](C(O)=O)N%10	11.4_4
C%11(COC1=CC=CC2=CC(C#N)=CC=C21)=O.C[CH](C%10=O)N%11.O=C(O)[CH](CC1=CC=CC=C1)N%10	11.4_5
C%11(COC1=CC=CC2=CC(C#N)=CC=C21)=O.C[CH](C%10=O)N%11.CC[CH](C)[CH](C(O)=O)N%10	11.4_6
C%11(COC1=CC=CC2=CC(C#N)=CC=C21)=O.C[CH](C%10=O)N%11.CSCC[CH](C(O)=O)N%10	11.4_7
C%11(COC1=CC=CC2=CC(C#N)=CC=C21)=O.C[CH](C%10=O)N%11.OC(C=C1)=CC=C1C[CH](C(O)=O)N%10	11.4_8
C%11(COC1=CC=CC2=CC(C#N)=CC=C21)=O.C[CH](C%10=O)N%11.O=C(O)[CH](CC1=C(F)C(F)=C(F)C(F)=C1F)N%10	11.4_9
C%11(COC1=CC=CC2=CC(C#N)=CC=C21)=O.O=C%10[CH](CC1=CC=CC=C1)N%11.O=C(O)CN%10	11.5_1
C%11(COC1=CC=CC2=CC(C#N)=CC=C21)=O.O=C%10[CH](CC1=CC=CC=C1)N%11.CC(C)[CH](C(O)=O)N%10	11.5_2
C%11(COC1=CC=CC2=CC(C#N)=CC=C21)=O.O=C%10[CH](CC1=CC=CC=C1)N%11.CC(C)C[CH](C(O)=O)N%10	11.5_3
C%11(COC1=CC=CC2=CC(C#N)=CC=C21)=O.O=C%10[CH](CC1=CC=CC=C1)N%11.C[CH](C(O)=O)N%10	11.5_4
C%11(COC1=CC=CC2=CC(C#N)=CC=C21)=O.O=C%10[CH](CC1=CC=CC=C1)N%11.O=C(O)[CH](CC1=CC=CC=C1)N%10	11.5_5
C%11(COC1=CC=CC2=CC(C#N)=CC=C21)=O.O=C%10[CH](CC1=CC=CC=C1)N%11.CC[CH](C)[CH](C(O)=O)N%10	11.5_6
C%11(COC1=CC=CC2=CC(C#N)=CC=C21)=O.O=C%10[CH](CC1=CC=CC=C1)N%11.CSCC[CH](C(O)=O)N%10	11.5_7
C%11(COC1=CC=CC2=CC(C#N)=CC=C21)=O.O=C%10[CH](CC1=CC=CC=C1)N%11.OC(C=C1)=CC=C1C[CH](C(O)=O)N%10	11.5_8
C%11(COC1=CC=CC2=CC(C#N)=CC=C21)=O.O=C%10[CH](CC1=CC=CC=C1)N%11.O=C(O)[CH](CC1=C(F)C(F)=C(F)C(F)=C1F)N%10	11.5_9
C%11(COC1=CC=CC2=CC(C#N)=CC=C21)=O.CC[CH](C)[CH](C%10=O)N%11.O=C(O)CN%10	11.6_1
C%11(COC1=CC=CC2=CC(C#N)=CC=C21)=O.CC[CH](C)[CH](C%10=O)N%11.CC(C)[CH](C(O)=O)N%10	11.6_2
C%11(COC1=CC=CC2=CC(C#N)=CC=C21)=O.CC[CH](C)[CH](C%10=O)N%11.CC(C)C[CH](C(O)=O)N%10	11.6_3
C%11(COC1=CC=CC2=CC(C#N)=CC=C21)=O.CC[CH](C)[CH](C%10=O)N%11.C[CH](C(O)=O)N%10	11.6_4
C%11(COC1=CC=CC2=CC(C#N)=CC=C21)=O.CC[CH](C)[CH](C%10=O)N%11.O=C(O)[CH](CC	11.6_5

1=CC=CC=C1)N%10	
C%11(COC1=CC=CC2=CC(C#N)=CC=C21)=O.CC[CH](C)[CH](C%10=O)N%11.CC[CH](C)[CH](C(O)=O)N%10	11.6_6
C%11(COC1=CC=CC2=CC(C#N)=CC=C21)=O.CC[CH](C)[CH](C%10=O)N%11.CSCC[CH](C(O)=O)N%10	11.6_7
C%11(COC1=CC=CC2=CC(C#N)=CC=C21)=O.CC[CH](C)[CH](C%10=O)N%11.OC(C=C1)=CC=C1C[CH](C(O)=O)N%10	11.6_8
C%11(COC1=CC=CC2=CC(C#N)=CC=C21)=O.CC[CH](C)[CH](C%10=O)N%11.O=C(O)[CH](CC1=C(F)C(F)=C(F)C(F)=C1F)N%10	11.6_9
C%11(COC1=CC=CC2=CC(C#N)=CC=C21)=O.CSCC[CH](C%10=O)N%11.O=C(O)CN%10	11.7_1
C%11(COC1=CC=CC2=CC(C#N)=CC=C21)=O.CSCC[CH](C%10=O)N%11.CC(C)[CH](C(O)=O)N%10	11.7_2
C%11(COC1=CC=CC2=CC(C#N)=CC=C21)=O.CSCC[CH](C%10=O)N%11.CC(C)C[CH](C(O)=O)N%10	11.7_3
C%11(COC1=CC=CC2=CC(C#N)=CC=C21)=O.CSCC[CH](C%10=O)N%11.C[CH](C(O)=O)N%10	11.7_4
C%11(COC1=CC=CC2=CC(C#N)=CC=C21)=O.CSCC[CH](C%10=O)N%11.O=C(O)[CH](CC1=CC=CC=C1)N%10	11.7_5
C%11(COC1=CC=CC2=CC(C#N)=CC=C21)=O.CSCC[CH](C%10=O)N%11.CC[CH](C)[CH](C(O)=O)N%10	11.7_6
C%11(COC1=CC=CC2=CC(C#N)=CC=C21)=O.CSCC[CH](C%10=O)N%11.CSCC[CH](C(O)=O)N%10	11.7_7
C%11(COC1=CC=CC2=CC(C#N)=CC=C21)=O.CSCC[CH](C%10=O)N%11.OC(C=C1)=CC=C1C[CH](C(O)=O)N%10	11.7_8
C%11(COC1=CC=CC2=CC(C#N)=CC=C21)=O.CSCC[CH](C%10=O)N%11.O=C(O)[CH](CC1=C(F)C(F)=C(F)C(F)=C1F)N%10	11.7_9
C%11(COC1=CC=CC2=CC(C#N)=CC=C21)=O.OC(C=C1)=CC=C1C[CH](C%10=O)N%11.O=C(O)CN%10	11.8_1
C%11(COC1=CC=CC2=CC(C#N)=CC=C21)=O.OC(C=C1)=CC=C1C[CH](C%10=O)N%11.CC(C)[CH](C(O)=O)N%10	11.8_2
C%11(COC1=CC=CC2=CC(C#N)=CC=C21)=O.OC(C=C1)=CC=C1C[CH](C%10=O)N%11.CC(C)C[CH](C(O)=O)N%10	11.8_3
C%11(COC1=CC=CC2=CC(C#N)=CC=C21)=O.OC(C=C1)=CC=C1C[CH](C%10=O)N%11.C[CH](C(O)=O)N%10	11.8_4
C%11(COC1=CC=CC2=CC(C#N)=CC=C21)=O.OC(C=C1)=CC=C1C[CH](C%10=O)N%11.O=C(O)[CH](CC1=CC=CC=C1)N%10	11.8_5
C%11(COC1=CC=CC2=CC(C#N)=CC=C21)=O.OC(C=C1)=CC=C1C[CH](C%10=O)N%11.CC[CH](C)[CH](C(O)=O)N%10	11.8_6
C%11(COC1=CC=CC2=CC(C#N)=CC=C21)=O.OC(C=C1)=CC=C1C[CH](C%10=O)N%11.CSCC[CH](C(O)=O)N%10	11.8_7
C%11(COC1=CC=CC2=CC(C#N)=CC=C21)=O.OC(C=C1)=CC=C1C[CH](C%10=O)N%11.OC(C=C1)=CC=C1C[CH](C(O)=O)N%10	11.8_8
C%11(COC1=CC=CC2=CC(C#N)=CC=C21)=O.OC(C=C1)=CC=C1C[CH](C%10=O)N%11.O=C(O)[CH](CC1=C(F)C(F)=C(F)C(F)=C1F)N%10	11.8_9
C%10(COC1=CC=CC2=CC(C#N)=CC=C21)=O.O=C(O)CN%10	11.9_1
C%10(COC1=CC=CC2=CC(C#N)=CC=C21)=O.CC(C)[CH](C(O)=O)N%10	11.9_2
C%10(COC1=CC=CC2=CC(C#N)=CC=C21)=O.CC(C)C[CH](C(O)=O)N%10	11.9_3
C%10(COC1=CC=CC2=CC(C#N)=CC=C21)=O.C[CH](C(O)=O)N%10	11.9_4

C%10(COC1=CC=CC2=CC(C#N)=CC=C21)=O.O=C(O)[CH](CC1=CC=CC=C1)N%10	11.9_5
C%10(COC1=CC=CC2=CC(C#N)=CC=C21)=O.CC[CH](C)[CH](C(O)=O)N%10	11.9_6
C%10(COC1=CC=CC2=CC(C#N)=CC=C21)=O.CSCC[CH](C(O)=O)N%10	11.9_7
C%10(COC1=CC=CC2=CC(C#N)=CC=C21)=O.OC(C=C1)=CC=C1C[CH](C(O)=O)N%10	11.9_8
C%10(COC1=CC=CC2=CC(C#N)=CC=C21)=O.O=C(O)[CH](CC1=C(F)C(F)=C(F)C(F)=C1F)N%1 0	11.9_9
C%11(COC1=CC2=CC=CC=C2C=C1OC)=O.O=C%10CN%11.O=C(O)CN%10	12.1_1
C%11(COC1=CC2=CC=CC=C2C=C1OC)=O.O=C%10CN%11.CC(C)[CH](C(O)=O)N%10	12.1_2
C%11(COC1=CC2=CC=CC=C2C=C1OC)=O.O=C%10CN%11.CC(C)C[CH](C(O)=O)N%10	12.1_3
C%11(COC1=CC2=CC=CC=C2C=C1OC)=O.O=C%10CN%11.C[CH](C(O)=O)N%10	12.1_4
C%11(COC1=CC2=CC=CC=C2C=C1OC)=O.O=C%10CN%11.O=C(O)[CH](CC1=CC=CC=C1)N% 10	12.1_5
C%11(COC1=CC2=CC=CC=C2C=C1OC)=O.O=C%10CN%11.CC[CH](C)[CH](C(O)=O)N%10	12.1_6
C%11(COC1=CC2=CC=CC=C2C=C1OC)=O.O=C%10CN%11.CSCC[CH](C(O)=O)N%10	12.1_7
C%11(COC1=CC2=CC=CC=C2C=C1OC)=O.O=C%10CN%11.OC(C=C1)=CC=C1C[CH](C(O)=O) N%10	12.1_8
C%11(COC1=CC2=CC=CC=C2C=C1OC)=O.O=C%10CN%11.O=C(O)[CH](CC1=C(F)C(F)=C(F)C (F)=C1F)N%10	12.1_9
C%11(COC1=CC2=CC=CC=C2C=C1OC)=O.CC(C)[CH](C%10=O)N%11.O=C(O)CN%10	12.2_1
C%11(COC1=CC2=CC=CC=C2C=C1OC)=O.CC(C)[CH](C%10=O)N%11.CC(C)[CH](C(O)=O)N% 10	12.2_2
C%11(COC1=CC2=CC=CC=C2C=C1OC)=O.CC(C)[CH](C%10=O)N%11.CC(C)C[CH](C(O)=O)N %10	12.2_3
C%11(COC1=CC2=CC=CC=C2C=C1OC)=O.CC(C)[CH](C%10=O)N%11.C[CH](C(O)=O)N%10	12.2_4
C%11(COC1=CC2=CC=CC=C2C=C1OC)=O.CC(C)[CH](C%10=O)N%11.O=C(O)[CH](CC1=CC= CC=C1)N%10	12.2_5
C%11(COC1=CC2=CC=CC=C2C=C1OC)=O.CC(C)[CH](C%10=O)N%11.CC[CH](C)[CH](C(O)= O)N%10	12.2_6
C%11(COC1=CC2=CC=CC=C2C=C1OC)=O.CC(C)[CH](C%10=O)N%11.CSCC[CH](C(O)=O)N% 10	12.2_7
C%11(COC1=CC2=CC=CC=C2C=C1OC)=O.CC(C)[CH](C%10=O)N%11.OC(C=C1)=CC=C1C[CH](C(O)=O)N%10	12.2_8
C%11(COC1=CC2=CC=CC=C2C=C1OC)=O.CC(C)[CH](C%10=O)N%11.O=C(O)[CH](CC1=C(F) C(F)=C(F)C(F)=C1F)N%10	12.2_9
C%11(COC1=CC2=CC=CC=C2C=C1OC)=O.CC(C)C[CH](C%10=O)N%11.O=C(O)CN%10	12.3_1
C%11(COC1=CC2=CC=CC=C2C=C1OC)=O.CC(C)C[CH](C%10=O)N%11.CC(C)[CH](C(O)=O)N %10	12.3_2
C%11(COC1=CC2=CC=CC=C2C=C1OC)=O.CC(C)C[CH](C%10=O)N%11.CC(C)C[CH](C(O)=O) N%10	12.3_3
C%11(COC1=CC2=CC=CC=C2C=C1OC)=O.CC(C)C[CH](C%10=O)N%11.C[CH](C(O)=O)N%10	12.3_4
C%11(COC1=CC2=CC=CC=C2C=C1OC)=O.CC(C)C[CH](C%10=O)N%11.O=C(O)[CH](CC1=CC =CC=C1)N%10	12.3_5
C%11(COC1=CC2=CC=CC=C2C=C1OC)=O.CC(C)C[CH](C%10=O)N%11.CC[CH](C)[CH](C(O)= O)N%10	12.3_6
C%11(COC1=CC2=CC=CC=C2C=C1OC)=O.CC(C)C[CH](C%10=O)N%11.CSCC[CH](C(O)=O)N %10	12.3_7
C%11(COC1=CC2=CC=CC=C2C=C1OC)=O.CC(C)C[CH](C%10=O)N%11.OC(C=C1)=CC=C1C[C H](C(O)=O)N%10	12.3_8

C%11(COC1=CC2=CC=CC=C2C=C1OC)=O.CC(C)C[CH](C%10=O)N%11.O=C(O)[CH](CC1=C(F)C(F)=C(F)C(F)=C1F)N%10	12.3_9
C%11(COC1=CC2=CC=CC=C2C=C1OC)=O.C[CH](C%10=O)N%11.O=C(O)CN%10	12.4_1
C%11(COC1=CC2=CC=CC=C2C=C1OC)=O.C[CH](C%10=O)N%11.CC(C)[CH](C(O)=O)N%10	12.4_2
C%11(COC1=CC2=CC=CC=C2C=C1OC)=O.C[CH](C%10=O)N%11.CC(C)C[CH](C(O)=O)N%10	12.4_3
C%11(COC1=CC2=CC=CC=C2C=C1OC)=O.C[CH](C%10=O)N%11.C[CH](C(O)=O)N%10	12.4_4
C%11(COC1=CC2=CC=CC=C2C=C1OC)=O.C[CH](C%10=O)N%11.O=C(O)[CH](CC1=CC=CC=C1)N%10	12.4_5
C%11(COC1=CC2=CC=CC=C2C=C1OC)=O.C[CH](C%10=O)N%11.CC[CH](C)[CH](C(O)=O)N%10	12.4_6
C%11(COC1=CC2=CC=CC=C2C=C1OC)=O.C[CH](C%10=O)N%11.CSCC[CH](C(O)=O)N%10	12.4_7
C%11(COC1=CC2=CC=CC=C2C=C1OC)=O.C[CH](C%10=O)N%11.OC(C=C1)=CC=C1C[CH](C(O)=O)N%10	12.4_8
C%11(COC1=CC2=CC=CC=C2C=C1OC)=O.C[CH](C%10=O)N%11.O=C(O)[CH](CC1=C(F)C(F)=C(F)C(F)=C1F)N%10	12.4_9
C%11(COC1=CC2=CC=CC=C2C=C1OC)=O.O=C%10[CH](CC1=CC=CC=C1)N%11.O=C(O)CN%10	12.5_1
C%11(COC1=CC2=CC=CC=C2C=C1OC)=O.O=C%10[CH](CC1=CC=CC=C1)N%11.CC(C)[CH](C(O)=O)N%10	12.5_2
C%11(COC1=CC2=CC=CC=C2C=C1OC)=O.O=C%10[CH](CC1=CC=CC=C1)N%11.CC(C)C[CH](C(O)=O)N%10	12.5_3
C%11(COC1=CC2=CC=CC=C2C=C1OC)=O.O=C%10[CH](CC1=CC=CC=C1)N%11.C[CH](C(O)=O)N%10	12.5_4
C%11(COC1=CC2=CC=CC=C2C=C1OC)=O.O=C%10[CH](CC1=CC=CC=C1)N%11.O=C(O)[CH](CC1=CC=CC=C1)N%10	12.5_5
C%11(COC1=CC2=CC=CC=C2C=C1OC)=O.O=C%10[CH](CC1=CC=CC=C1)N%11.CC[CH](C)[CH](C(O)=O)N%10	12.5_6
C%11(COC1=CC2=CC=CC=C2C=C1OC)=O.O=C%10[CH](CC1=CC=CC=C1)N%11.CSCC[CH](C(O)=O)N%10	12.5_7
C%11(COC1=CC2=CC=CC=C2C=C1OC)=O.O=C%10[CH](CC1=CC=CC=C1)N%11.OC(C=C1)=CC=C1C[CH](C(O)=O)N%10	12.5_8
C%11(COC1=CC2=CC=CC=C2C=C1OC)=O.O=C%10[CH](CC1=CC=CC=C1)N%11.O=C(O)[CH](CC1=C(F)C(F)=C(F)C(F)=C1F)N%10	12.5_9
C%11(COC1=CC2=CC=CC=C2C=C1OC)=O.CC[CH](C)[CH](C%10=O)N%11.O=C(O)CN%10	12.6_1
C%11(COC1=CC2=CC=CC=C2C=C1OC)=O.CC[CH](C)[CH](C%10=O)N%11.CC(C)[CH](C(O)=O)N%10	12.6_2
C%11(COC1=CC2=CC=CC=C2C=C1OC)=O.CC[CH](C)[CH](C%10=O)N%11.CC(C)C[CH](C(O)=O)N%10	12.6_3
C%11(COC1=CC2=CC=CC=C2C=C1OC)=O.CC[CH](C)[CH](C%10=O)N%11.C[CH](C(O)=O)N%10	12.6_4
C%11(COC1=CC2=CC=CC=C2C=C1OC)=O.CC[CH](C)[CH](C%10=O)N%11.O=C(O)[CH](CC1=CC=CC=C1)N%10	12.6_5
C%11(COC1=CC2=CC=CC=C2C=C1OC)=O.CC[CH](C)[CH](C%10=O)N%11.CC[CH](C)[CH](C(O)=O)N%10	12.6_6
C%11(COC1=CC2=CC=CC=C2C=C1OC)=O.CC[CH](C)[CH](C%10=O)N%11.CSCC[CH](C(O)=O)N%10	12.6_7
C%11(COC1=CC2=CC=CC=C2C=C1OC)=O.CC[CH](C)[CH](C%10=O)N%11.OC(C=C1)=CC=C1C[CH](C(O)=O)N%10	12.6_8

C%11(COC1=CC2=CC=CC=C2C=C1OC)=O.CC[CH](C)[CH](C%10=O)N%11.O=C(O)[CH](CC1=C(F)C(F)=C(F)C(F)=C1F)N%10	12.6_9
C%11(COC1=CC2=CC=CC=C2C=C1OC)=O.CSCC[CH](C%10=O)N%11.O=C(O)CN%10	12.7_1
C%11(COC1=CC2=CC=CC=C2C=C1OC)=O.CSCC[CH](C%10=O)N%11.CC(C)[CH](C(O)=O)N%10	12.7_2
C%11(COC1=CC2=CC=CC=C2C=C1OC)=O.CSCC[CH](C%10=O)N%11.CC(C)C[CH](C(O)=O)N%10	12.7_3
C%11(COC1=CC2=CC=CC=C2C=C1OC)=O.CSCC[CH](C%10=O)N%11.C[CH](C(O)=O)N%10	12.7_4
C%11(COC1=CC2=CC=CC=C2C=C1OC)=O.CSCC[CH](C%10=O)N%11.O=C(O)[CH](CC1=CC=CC=C1)N%10	12.7_5
C%11(COC1=CC2=CC=CC=C2C=C1OC)=O.CSCC[CH](C%10=O)N%11.CC[CH](C)[CH](C(O)=O)N%10	12.7_6
C%11(COC1=CC2=CC=CC=C2C=C1OC)=O.CSCC[CH](C%10=O)N%11.CSCC[CH](C(O)=O)N%10	12.7_7
C%11(COC1=CC2=CC=CC=C2C=C1OC)=O.CSCC[CH](C%10=O)N%11.OC(C=C1)=CC=C1C[CH](C(O)=O)N%10	12.7_8
C%11(COC1=CC2=CC=CC=C2C=C1OC)=O.CSCC[CH](C%10=O)N%11.O=C(O)[CH](CC1=C(F)C(F)=C(F)C(F)=C1F)N%10	12.7_9
C%11(COC1=CC2=CC=CC=C2C=C1OC)=O.OC(C=C1)=CC=C1C[CH](C%10=O)N%11.O=C(O)CN%10	12.8_1
C%11(COC1=CC2=CC=CC=C2C=C1OC)=O.OC(C=C1)=CC=C1C[CH](C%10=O)N%11.CC(C)[CH](C(O)=O)N%10	12.8_2
C%11(COC1=CC2=CC=CC=C2C=C1OC)=O.OC(C=C1)=CC=C1C[CH](C%10=O)N%11.CC(C)C[CH](C(O)=O)N%10	12.8_3
C%11(COC1=CC2=CC=CC=C2C=C1OC)=O.OC(C=C1)=CC=C1C[CH](C%10=O)N%11.C[CH](C(O)=O)N%10	12.8_4
C%11(COC1=CC2=CC=CC=C2C=C1OC)=O.OC(C=C1)=CC=C1C[CH](C%10=O)N%11.O=C(O)[CH](CC1=CC=CC=C1)N%10	12.8_5
C%11(COC1=CC2=CC=CC=C2C=C1OC)=O.OC(C=C1)=CC=C1C[CH](C%10=O)N%11.CC[CH](C)[CH](C(O)=O)N%10	12.8_6
C%11(COC1=CC2=CC=CC=C2C=C1OC)=O.OC(C=C1)=CC=C1C[CH](C%10=O)N%11.CSCC[CH](C(O)=O)N%10	12.8_7
C%11(COC1=CC2=CC=CC=C2C=C1OC)=O.OC(C=C1)=CC=C1C[CH](C%10=O)N%11.OC(C=C1)=CC=C1C[CH](C(O)=O)N%10	12.8_8
C%11(COC1=CC2=CC=CC=C2C=C1OC)=O.OC(C=C1)=CC=C1C[CH](C%10=O)N%11.O=C(O)[CH](CC1=C(F)C(F)=C(F)C(F)=C1F)N%10	12.8_9
C%10(COC1=CC2=CC=CC=C2C=C1OC)=O.O=C(O)CN%10	12.9_1
C%10(COC1=CC2=CC=CC=C2C=C1OC)=O.CC(C)[CH](C(O)=O)N%10	12.9_2
C%10(COC1=CC2=CC=CC=C2C=C1OC)=O.CC(C)C[CH](C(O)=O)N%10	12.9_3
C%10(COC1=CC2=CC=CC=C2C=C1OC)=O.C[CH](C(O)=O)N%10	12.9_4
C%10(COC1=CC2=CC=CC=C2C=C1OC)=O.O=C(O)[CH](CC1=CC=CC=C1)N%10	12.9_5
C%10(COC1=CC2=CC=CC=C2C=C1OC)=O.CC[CH](C)[CH](C(O)=O)N%10	12.9_6
C%10(COC1=CC2=CC=CC=C2C=C1OC)=O.CSCC[CH](C(O)=O)N%10	12.9_7
C%10(COC1=CC2=CC=CC=C2C=C1OC)=O.OC(C=C1)=CC=C1C[CH](C(O)=O)N%10	12.9_8
C%10(COC1=CC2=CC=CC=C2C=C1OC)=O.O=C(O)[CH](CC1=C(F)C(F)=C(F)C(F)=C1F)N%10	12.9_9
C%11(COC1=CC=CC2=CC=CC=C21)=O.O=C%10CN%11.O=C(O)CN%10	13.1_1
C%11(COC1=CC=CC2=CC=CC=C21)=O.O=C%10CN%11.CC(C)[CH](C(O)=O)N%10	13.1_2
C%11(COC1=CC=CC2=CC=CC=C21)=O.O=C%10CN%11.CC(C)C[CH](C(O)=O)N%10	13.1_3

C%11(COC1=CC=CC2=CC=CC=C21)=O.O=C%10CN%11.C[CH](C(O)=O)N%10	13.1_4
C%11(COC1=CC=CC2=CC=CC=C21)=O.O=C%10CN%11.O=C(O)[CH](CC1=CC=CC=C1)N%10	13.1_5
C%11(COC1=CC=CC2=CC=CC=C21)=O.O=C%10CN%11.CC[CH](C)[CH](C(O)=O)N%10	13.1_6
C%11(COC1=CC=CC2=CC=CC=C21)=O.O=C%10CN%11.CSCC[CH](C(O)=O)N%10	13.1_7
C%11(COC1=CC=CC2=CC=CC=C21)=O.O=C%10CN%11.OC(C=C1)=CC=C1C[CH](C(O)=O)N%10	13.1_8
C%11(COC1=CC=CC2=CC=CC=C21)=O.O=C%10CN%11.O=C(O)[CH](CC1=C(F)C(F)=C(F)C(F)=C1F)N%10	13.1_9
C%11(COC1=CC=CC2=CC=CC=C21)=O.CC(C)[CH](C%10=O)N%11.O=C(O)CN%10	13.2_1
C%11(COC1=CC=CC2=CC=CC=C21)=O.CC(C)[CH](C%10=O)N%11.CC(C)[CH](C(O)=O)N%10	13.2_2
C%11(COC1=CC=CC2=CC=CC=C21)=O.CC(C)[CH](C%10=O)N%11.CC(C)C[CH](C(O)=O)N%10	13.2_3
C%11(COC1=CC=CC2=CC=CC=C21)=O.CC(C)[CH](C%10=O)N%11.C[CH](C(O)=O)N%10	13.2_4
C%11(COC1=CC=CC2=CC=CC=C21)=O.CC(C)[CH](C%10=O)N%11.O=C(O)[CH](CC1=CC=CC=C1)N%10	13.2_5
C%11(COC1=CC=CC2=CC=CC=C21)=O.CC(C)[CH](C%10=O)N%11.CC[CH](C)[CH](C(O)=O)N%10	13.2_6
C%11(COC1=CC=CC2=CC=CC=C21)=O.CC(C)[CH](C%10=O)N%11.CSCC[CH](C(O)=O)N%10	13.2_7
C%11(COC1=CC=CC2=CC=CC=C21)=O.CC(C)[CH](C%10=O)N%11.OC(C=C1)=CC=C1C[CH](C(O)=O)N%10	13.2_8
C%11(COC1=CC=CC2=CC=CC=C21)=O.CC(C)[CH](C%10=O)N%11.O=C(O)[CH](CC1=C(F)C(F)=C(F)C(F)=C1F)N%10	13.2_9
C%11(COC1=CC=CC2=CC=CC=C21)=O.CC(C)C[CH](C%10=O)N%11.O=C(O)CN%10	13.3_1
C%11(COC1=CC=CC2=CC=CC=C21)=O.CC(C)C[CH](C%10=O)N%11.CC(C)[CH](C(O)=O)N%10	13.3_2
C%11(COC1=CC=CC2=CC=CC=C21)=O.CC(C)C[CH](C%10=O)N%11.CC(C)C[CH](C(O)=O)N%10	13.3_3
C%11(COC1=CC=CC2=CC=CC=C21)=O.CC(C)C[CH](C%10=O)N%11.C[CH](C(O)=O)N%10	13.3_4
C%11(COC1=CC=CC2=CC=CC=C21)=O.CC(C)C[CH](C%10=O)N%11.O=C(O)[CH](CC1=CC=CC=C1)N%10	13.3_5
C%11(COC1=CC=CC2=CC=CC=C21)=O.CC(C)C[CH](C%10=O)N%11.CC[CH](C)[CH](C(O)=O)N%10	13.3_6
C%11(COC1=CC=CC2=CC=CC=C21)=O.CC(C)C[CH](C%10=O)N%11.CSCC[CH](C(O)=O)N%10	13.3_7
C%11(COC1=CC=CC2=CC=CC=C21)=O.CC(C)C[CH](C%10=O)N%11.OC(C=C1)=CC=C1C[CH](C(O)=O)N%10	13.3_8
C%11(COC1=CC=CC2=CC=CC=C21)=O.CC(C)C[CH](C%10=O)N%11.O=C(O)[CH](CC1=C(F)C(F)=C(F)C(F)=C1F)N%10	13.3_9
C%11(COC1=CC=CC2=CC=CC=C21)=O.C[CH](C%10=O)N%11.O=C(O)CN%10	13.4_1
C%11(COC1=CC=CC2=CC=CC=C21)=O.C[CH](C%10=O)N%11.CC(C)[CH](C(O)=O)N%10	13.4_2
C%11(COC1=CC=CC2=CC=CC=C21)=O.C[CH](C%10=O)N%11.CC(C)C[CH](C(O)=O)N%10	13.4_3
C%11(COC1=CC=CC2=CC=CC=C21)=O.C[CH](C%10=O)N%11.C[CH](C(O)=O)N%10	13.4_4
C%11(COC1=CC=CC2=CC=CC=C21)=O.C[CH](C%10=O)N%11.O=C(O)[CH](CC1=CC=CC=C1)N%10	13.4_5
C%11(COC1=CC=CC2=CC=CC=C21)=O.C[CH](C%10=O)N%11.CC[CH](C)[CH](C(O)=O)N%10	13.4_6
C%11(COC1=CC=CC2=CC=CC=C21)=O.C[CH](C%10=O)N%11.CSCC[CH](C(O)=O)N%10	13.4_7
C%11(COC1=CC=CC2=CC=CC=C21)=O.C[CH](C%10=O)N%11.OC(C=C1)=CC=C1C[CH](C(O)	13.4_8

=O)N%10	
C%11(COC1=CC=CC2=CC=CC=C21)=O.C[CH](C%10=O)N%11.O=C(O)[CH](CC1=C(F)C(F)=C(F)C(F)=C1F)N%10	13.4_9
C%11(COC1=CC=CC2=CC=CC=C21)=O.O=C%10[CH](CC1=CC=CC=C1)N%11.O=C(O)CN%10	13.5_1
C%11(COC1=CC=CC2=CC=CC=C21)=O.O=C%10[CH](CC1=CC=CC=C1)N%11.CC(C)[CH](C(O)=O)N%10	13.5_2
C%11(COC1=CC=CC2=CC=CC=C21)=O.O=C%10[CH](CC1=CC=CC=C1)N%11.CC(C)C[CH](C(O)=O)N%10	13.5_3
C%11(COC1=CC=CC2=CC=CC=C21)=O.O=C%10[CH](CC1=CC=CC=C1)N%11.C[CH](C(O)=O)N%10	13.5_4
C%11(COC1=CC=CC2=CC=CC=C21)=O.O=C%10[CH](CC1=CC=CC=C1)N%11.O=C(O)[CH](CC1=CC=CC=C1)N%10	13.5_5
C%11(COC1=CC=CC2=CC=CC=C21)=O.O=C%10[CH](CC1=CC=CC=C1)N%11.CC[CH](C)[CH](C(O)=O)N%10	13.5_6
C%11(COC1=CC=CC2=CC=CC=C21)=O.O=C%10[CH](CC1=CC=CC=C1)N%11.CSCC[CH](C(O)=O)N%10	13.5_7
C%11(COC1=CC=CC2=CC=CC=C21)=O.O=C%10[CH](CC1=CC=CC=C1)N%11.OC(C=C1)=CC=C1C[CH](C(O)=O)N%10	13.5_8
C%11(COC1=CC=CC2=CC=CC=C21)=O.O=C%10[CH](CC1=CC=CC=C1)N%11.O=C(O)[CH](CC1=C(F)C(F)=C(F)C(F)=C1F)N%10	13.5_9
C%11(COC1=CC=CC2=CC=CC=C21)=O.CC[CH](C)[CH](C%10=O)N%11.O=C(O)CN%10	13.6_1
C%11(COC1=CC=CC2=CC=CC=C21)=O.CC[CH](C)[CH](C%10=O)N%11.CC(C)[CH](C(O)=O)N%10	13.6_2
C%11(COC1=CC=CC2=CC=CC=C21)=O.CC[CH](C)[CH](C%10=O)N%11.CC(C)C[CH](C(O)=O)N%10	13.6_3
C%11(COC1=CC=CC2=CC=CC=C21)=O.CC[CH](C)[CH](C%10=O)N%11.C[CH](C(O)=O)N%10	13.6_4
C%11(COC1=CC=CC2=CC=CC=C21)=O.CC[CH](C)[CH](C%10=O)N%11.O=C(O)[CH](CC1=CC=CC=C1)N%10	13.6_5
C%11(COC1=CC=CC2=CC=CC=C21)=O.CC[CH](C)[CH](C%10=O)N%11.CC[CH](C)[CH](C(O)=O)N%10	13.6_6
C%11(COC1=CC=CC2=CC=CC=C21)=O.CC[CH](C)[CH](C%10=O)N%11.CSCC[CH](C(O)=O)N%10	13.6_7
C%11(COC1=CC=CC2=CC=CC=C21)=O.CC[CH](C)[CH](C%10=O)N%11.OC(C=C1)=CC=C1C[CH](C(O)=O)N%10	13.6_8
C%11(COC1=CC=CC2=CC=CC=C21)=O.CC[CH](C)[CH](C%10=O)N%11.O=C(O)[CH](CC1=C(F)C(F)=C(F)C(F)=C1F)N%10	13.6_9
C%11(COC1=CC=CC2=CC=CC=C21)=O.CSCC[CH](C%10=O)N%11.O=C(O)CN%10	13.7_1
C%11(COC1=CC=CC2=CC=CC=C21)=O.CSCC[CH](C%10=O)N%11.CC(C)[CH](C(O)=O)N%10	13.7_2
C%11(COC1=CC=CC2=CC=CC=C21)=O.CSCC[CH](C%10=O)N%11.CC(C)C[CH](C(O)=O)N%10	13.7_3
C%11(COC1=CC=CC2=CC=CC=C21)=O.CSCC[CH](C%10=O)N%11.C[CH](C(O)=O)N%10	13.7_4
C%11(COC1=CC=CC2=CC=CC=C21)=O.CSCC[CH](C%10=O)N%11.O=C(O)[CH](CC1=CC=CC=C1)N%10	13.7_5
C%11(COC1=CC=CC2=CC=CC=C21)=O.CSCC[CH](C%10=O)N%11.CC[CH](C)[CH](C(O)=O)N%10	13.7_6
C%11(COC1=CC=CC2=CC=CC=C21)=O.CSCC[CH](C%10=O)N%11.CSCC[CH](C(O)=O)N%10	13.7_7
C%11(COC1=CC=CC2=CC=CC=C21)=O.CSCC[CH](C%10=O)N%11.OC(C=C1)=CC=C1C[CH](C(O)=O)N%10	13.7_8

C%11(COC1=CC=CC2=CC=CC=C21)=O.CSCC[CH](C%10=O)N%11.O=C(O)[CH](CC1=C(F)C(F)=C(F)C(F)=C1F)N%10	13.7_9
C%11(COC1=CC=CC2=CC=CC=C21)=O.OC(C=C1)=CC=C1C[CH](C%10=O)N%11.O=C(O)CN%10	13.8_1
C%11(COC1=CC=CC2=CC=CC=C21)=O.OC(C=C1)=CC=C1C[CH](C%10=O)N%11.CC(C)[CH](C(O)=O)N%10	13.8_2
C%11(COC1=CC=CC2=CC=CC=C21)=O.OC(C=C1)=CC=C1C[CH](C%10=O)N%11.CC(C)C[CH](C(O)=O)N%10	13.8_3
C%11(COC1=CC=CC2=CC=CC=C21)=O.OC(C=C1)=CC=C1C[CH](C%10=O)N%11.C[CH](C(O)=O)N%10	13.8_4
C%11(COC1=CC=CC2=CC=CC=C21)=O.OC(C=C1)=CC=C1C[CH](C%10=O)N%11.O=C(O)[CH](CC1=CC=CC=C1)N%10	13.8_5
C%11(COC1=CC=CC2=CC=CC=C21)=O.OC(C=C1)=CC=C1C[CH](C%10=O)N%11.CC[CH](C)[CH](C(O)=O)N%10	13.8_6
C%11(COC1=CC=CC2=CC=CC=C21)=O.OC(C=C1)=CC=C1C[CH](C%10=O)N%11.CSCC[CH](C(O)=O)N%10	13.8_7
C%11(COC1=CC=CC2=CC=CC=C21)=O.OC(C=C1)=CC=C1C[CH](C%10=O)N%11.OC(C=C1)=CC=C1C[CH](C(O)=O)N%10	13.8_8
C%11(COC1=CC=CC2=CC=CC=C21)=O.OC(C=C1)=CC=C1C[CH](C%10=O)N%11.O=C(O)[CH](CC1=C(F)C(F)=C(F)C(F)=C1F)N%10	13.8_9
C%10(COC1=CC=CC2=CC=CC=C21)=O.O=C(O)CN%10	13.9_1
C%10(COC1=CC=CC2=CC=CC=C21)=O.CC(C)[CH](C(O)=O)N%10	13.9_2
C%10(COC1=CC=CC2=CC=CC=C21)=O.CC(C)C[CH](C(O)=O)N%10	13.9_3
C%10(COC1=CC=CC2=CC=CC=C21)=O.C[CH](C(O)=O)N%10	13.9_4
C%10(COC1=CC=CC2=CC=CC=C21)=O.O=C(O)[CH](CC1=CC=CC=C1)N%10	13.9_5
C%10(COC1=CC=CC2=CC=CC=C21)=O.CC[CH](C)[CH](C(O)=O)N%10	13.9_6
C%10(COC1=CC=CC2=CC=CC=C21)=O.CSCC[CH](C(O)=O)N%10	13.9_7
C%10(COC1=CC=CC2=CC=CC=C21)=O.OC(C=C1)=CC=C1C[CH](C(O)=O)N%10	13.9_8
C%10(COC1=CC=CC2=CC=CC=C21)=O.O=C(O)[CH](CC1=C(F)C(F)=C(F)C(F)=C1F)N%10	13.9_9
C%11(COC1=CC=CC2=CC(C#N)=CC=C21)=O.O=C%10CN%11.O=C(O)CN%10	14.1_1
C%11(COC1=CC=CC2=CC(C#N)=CC=C21)=O.O=C%10CN%11.CC(C)[CH](C(O)=O)N%10	14.1_2
C%11(COC1=CC=CC2=CC(C#N)=CC=C21)=O.O=C%10CN%11.CC(C)C[CH](C(O)=O)N%10	14.1_3
C%11(COC1=CC=CC2=CC(C#N)=CC=C21)=O.O=C%10CN%11.C[CH](C(O)=O)N%10	14.1_4
C%11(COC1=CC=CC2=CC(C#N)=CC=C21)=O.O=C%10CN%11.O=C(O)[CH](CC1=CC=CC=C1)N%10	14.1_5
C%11(COC1=CC=CC2=CC(C#N)=CC=C21)=O.O=C%10CN%11.CC[CH](C)[CH](C(O)=O)N%10	14.1_6
C%11(COC1=CC=CC2=CC(C#N)=CC=C21)=O.O=C%10CN%11.CSCC[CH](C(O)=O)N%10	14.1_7
C%11(COC1=CC=CC2=CC(C#N)=CC=C21)=O.O=C%10CN%11.OC(C=C1)=CC=C1C[CH](C(O)=O)N%10	14.1_8
C%11(COC1=CC=CC2=CC(C#N)=CC=C21)=O.O=C%10CN%11.O=C(O)[CH](CC1=C(F)C(F)=C(F)C(F)=C1F)N%10	14.1_9
C%11(COC1=CC=CC2=CC(C#N)=CC=C21)=O.CC(C)[CH](C%10=O)N%11.O=C(O)CN%10	14.2_1
C%11(COC1=CC=CC2=CC(C#N)=CC=C21)=O.CC(C)[CH](C%10=O)N%11.CC(C)[CH](C(O)=O)N%10	14.2_2
C%11(COC1=CC=CC2=CC(C#N)=CC=C21)=O.CC(C)[CH](C%10=O)N%11.CC(C)C[CH](C(O)=O)N%10	14.2_3
C%11(COC1=CC=CC2=CC(C#N)=CC=C21)=O.CC(C)[CH](C%10=O)N%11.C[CH](C(O)=O)N%1	14.2_4

0	
C%11(COC1=CC=CC2=CC(C#N)=CC=C21)=O.CC(C)[CH](C%10=O)N%11.O=C(O)[CH](CC1=CC=CC=C1)N%10	14.2_5
C%11(COC1=CC=CC2=CC(C#N)=CC=C21)=O.CC(C)[CH](C%10=O)N%11.CC[CH](C)[CH](C(O)=O)N%10	14.2_6
C%11(COC1=CC=CC2=CC(C#N)=CC=C21)=O.CC(C)[CH](C%10=O)N%11.CSCC[CH](C(O)=O)N%10	14.2_7
C%11(COC1=CC=CC2=CC(C#N)=CC=C21)=O.CC(C)[CH](C%10=O)N%11.OC(C=C1)=CC=C1C[CH](C(O)=O)N%10	14.2_8
C%11(COC1=CC=CC2=CC(C#N)=CC=C21)=O.CC(C)[CH](C%10=O)N%11.O=C(O)[CH](CC1=C(F)C(F)=C(F)C(F)=C1F)N%10	14.2_9
C%11(COC1=CC=CC2=CC(C#N)=CC=C21)=O.CC(C)C[CH](C%10=O)N%11.O=C(O)CN%10	14.3_1
C%11(COC1=CC=CC2=CC(C#N)=CC=C21)=O.CC(C)C[CH](C%10=O)N%11.CC(C)[CH](C(O)=O)N%10	14.3_2
C%11(COC1=CC=CC2=CC(C#N)=CC=C21)=O.CC(C)C[CH](C%10=O)N%11.CC(C)C[CH](C(O)=O)N%10	14.3_3
C%11(COC1=CC=CC2=CC(C#N)=CC=C21)=O.CC(C)C[CH](C%10=O)N%11.C[CH](C(O)=O)N%10	14.3_4
C%11(COC1=CC=CC2=CC(C#N)=CC=C21)=O.CC(C)C[CH](C%10=O)N%11.O=C(O)[CH](CC1=CC=CC=C1)N%10	14.3_5
C%11(COC1=CC=CC2=CC(C#N)=CC=C21)=O.CC(C)C[CH](C%10=O)N%11.CC[CH](C)[CH](C(O)=O)N%10	14.3_6
C%11(COC1=CC=CC2=CC(C#N)=CC=C21)=O.CC(C)C[CH](C%10=O)N%11.CSCC[CH](C(O)=O)N%10	14.3_7
C%11(COC1=CC=CC2=CC(C#N)=CC=C21)=O.CC(C)C[CH](C%10=O)N%11.OC(C=C1)=CC=C1C[CH](C(O)=O)N%10	14.3_8
C%11(COC1=CC=CC2=CC(C#N)=CC=C21)=O.CC(C)C[CH](C%10=O)N%11.O=C(O)[CH](CC1=C(F)C(F)=C(F)C(F)=C1F)N%10	14.3_9
C%11(COC1=CC=CC2=CC(C#N)=CC=C21)=O.C[CH](C%10=O)N%11.O=C(O)CN%10	14.4_1
C%11(COC1=CC=CC2=CC(C#N)=CC=C21)=O.C[CH](C%10=O)N%11.CC(C)[CH](C(O)=O)N%10	14.4_2
C%11(COC1=CC=CC2=CC(C#N)=CC=C21)=O.C[CH](C%10=O)N%11.CC(C)C[CH](C(O)=O)N%10	14.4_3
C%11(COC1=CC=CC2=CC(C#N)=CC=C21)=O.C[CH](C%10=O)N%11.C[CH](C(O)=O)N%10	14.4_4
C%11(COC1=CC=CC2=CC(C#N)=CC=C21)=O.C[CH](C%10=O)N%11.O=C(O)[CH](CC1=CC=CC=C1)N%10	14.4_5
C%11(COC1=CC=CC2=CC(C#N)=CC=C21)=O.C[CH](C%10=O)N%11.CC[CH](C)[CH](C(O)=O)N%10	14.4_6
C%11(COC1=CC=CC2=CC(C#N)=CC=C21)=O.C[CH](C%10=O)N%11.CSCC[CH](C(O)=O)N%10	14.4_7
C%11(COC1=CC=CC2=CC(C#N)=CC=C21)=O.C[CH](C%10=O)N%11.OC(C=C1)=CC=C1C[CH](C(O)=O)N%10	14.4_8
C%11(COC1=CC=CC2=CC(C#N)=CC=C21)=O.C[CH](C%10=O)N%11.O=C(O)[CH](CC1=C(F)C(F)=C(F)C(F)=C1F)N%10	14.4_9
C%11(COC1=CC=CC2=CC(C#N)=CC=C21)=O.O=C%10[CH](CC1=CC=CC=C1)N%11.O=C(O)CN%10	14.5_1
C%11(COC1=CC=CC2=CC(C#N)=CC=C21)=O.O=C%10[CH](CC1=CC=CC=C1)N%11.CC(C)[CH](C(O)=O)N%10	14.5_2
C%11(COC1=CC=CC2=CC(C#N)=CC=C21)=O.O=C%10[CH](CC1=CC=CC=C1)N%11.CC(C)C[C	14.5_3

H](C(O)=O)N%10	
C%11(COC1=CC=CC2=CC(C#N)=CC=C21)=O.O=C%10[CH](CC1=CC=CC=C1)N%11.C[CH](C(O)=O)N%10	14.5_4
C%11(COC1=CC=CC2=CC(C#N)=CC=C21)=O.O=C%10[CH](CC1=CC=CC=C1)N%11.O=C(O)[CH](CC1=CC=CC=C1)N%10	14.5_5
C%11(COC1=CC=CC2=CC(C#N)=CC=C21)=O.O=C%10[CH](CC1=CC=CC=C1)N%11.CC[CH](C)[CH](C(O)=O)N%10	14.5_6
C%11(COC1=CC=CC2=CC(C#N)=CC=C21)=O.O=C%10[CH](CC1=CC=CC=C1)N%11.CSCC[CH](C(O)=O)N%10	14.5_7
C%11(COC1=CC=CC2=CC(C#N)=CC=C21)=O.O=C%10[CH](CC1=CC=CC=C1)N%11.OC(C=C1)=CC=C1C[CH](C(O)=O)N%10	14.5_8
C%11(COC1=CC=CC2=CC(C#N)=CC=C21)=O.O=C%10[CH](CC1=CC=CC=C1)N%11.O=C(O)[CH](CC1=C(F)C(F)=C(F)C(F)=C1F)N%10	14.5_9
C%11(COC1=CC=CC2=CC(C#N)=CC=C21)=O.CC[CH](C)[CH](C%10=O)N%11.O=C(O)CN%10	14.6_1
C%11(COC1=CC=CC2=CC(C#N)=CC=C21)=O.CC[CH](C)[CH](C%10=O)N%11.CC(C)[CH](C(O)=O)N%10	14.6_2
C%11(COC1=CC=CC2=CC(C#N)=CC=C21)=O.CC[CH](C)[CH](C%10=O)N%11.CC(C)C[CH](C(O)=O)N%10	14.6_3
C%11(COC1=CC=CC2=CC(C#N)=CC=C21)=O.CC[CH](C)[CH](C%10=O)N%11.C[CH](C(O)=O)N%10	14.6_4
C%11(COC1=CC=CC2=CC(C#N)=CC=C21)=O.CC[CH](C)[CH](C%10=O)N%11.O=C(O)[CH](CC1=CC=CC=C1)N%10	14.6_5
C%11(COC1=CC=CC2=CC(C#N)=CC=C21)=O.CC[CH](C)[CH](C%10=O)N%11.CC[CH](C)[CH](C(O)=O)N%10	14.6_6
C%11(COC1=CC=CC2=CC(C#N)=CC=C21)=O.CC[CH](C)[CH](C%10=O)N%11.CSCC[CH](C(O)=O)N%10	14.6_7
C%11(COC1=CC=CC2=CC(C#N)=CC=C21)=O.CC[CH](C)[CH](C%10=O)N%11.OC(C=C1)=CC=C1C[CH](C(O)=O)N%10	14.6_8
C%11(COC1=CC=CC2=CC(C#N)=CC=C21)=O.CC[CH](C)[CH](C%10=O)N%11.O=C(O)[CH](CC1=C(F)C(F)=C(F)C(F)=C1F)N%10	14.6_9
C%11(COC1=CC=CC2=CC(C#N)=CC=C21)=O.CSCC[CH](C%10=O)N%11.O=C(O)CN%10	14.7_1
C%11(COC1=CC=CC2=CC(C#N)=CC=C21)=O.CSCC[CH](C%10=O)N%11.CC(C)[CH](C(O)=O)N%10	14.7_2
C%11(COC1=CC=CC2=CC(C#N)=CC=C21)=O.CSCC[CH](C%10=O)N%11.CC(C)C[CH](C(O)=O)N%10	14.7_3
C%11(COC1=CC=CC2=CC(C#N)=CC=C21)=O.CSCC[CH](C%10=O)N%11.C[CH](C(O)=O)N%10	14.7_4
C%11(COC1=CC=CC2=CC(C#N)=CC=C21)=O.CSCC[CH](C%10=O)N%11.O=C(O)[CH](CC1=CC=CC=C1)N%10	14.7_5
C%11(COC1=CC=CC2=CC(C#N)=CC=C21)=O.CSCC[CH](C%10=O)N%11.CC[CH](C)[CH](C(O)=O)N%10	14.7_6
C%11(COC1=CC=CC2=CC(C#N)=CC=C21)=O.CSCC[CH](C%10=O)N%11.CSCC[CH](C(O)=O)N%10	14.7_7
C%11(COC1=CC=CC2=CC(C#N)=CC=C21)=O.CSCC[CH](C%10=O)N%11.OC(C=C1)=CC=C1C[CH](C(O)=O)N%10	14.7_8
C%11(COC1=CC=CC2=CC(C#N)=CC=C21)=O.CSCC[CH](C%10=O)N%11.O=C(O)[CH](CC1=C(F)C(F)=C(F)C(F)=C1F)N%10	14.7_9
C%11(COC1=CC=CC2=CC(C#N)=CC=C21)=O.OC(C=C1)=CC=C1C[CH](C%10=O)N%11.O=C(O)CN%10	14.8_1

C%11(COC1=CC=CC2=CC(C#N)=CC=C21)=O.OC(C=C1)=CC=C1C[CH](C%10=O)N%11.CC(C)[CH](C(O)=O)N%10	14.8_2
C%11(COC1=CC=CC2=CC(C#N)=CC=C21)=O.OC(C=C1)=CC=C1C[CH](C%10=O)N%11.CC(C)C[CH](C(O)=O)N%10	14.8_3
C%11(COC1=CC=CC2=CC(C#N)=CC=C21)=O.OC(C=C1)=CC=C1C[CH](C%10=O)N%11.C[CH](C(O)=O)N%10	14.8_4
C%11(COC1=CC=CC2=CC(C#N)=CC=C21)=O.OC(C=C1)=CC=C1C[CH](C%10=O)N%11.O=C(O)[CH](CC1=CC=CC=C1)N%10	14.8_5
C%11(COC1=CC=CC2=CC(C#N)=CC=C21)=O.OC(C=C1)=CC=C1C[CH](C%10=O)N%11.CC[CH](C)[CH](C(O)=O)N%10	14.8_6
C%11(COC1=CC=CC2=CC(C#N)=CC=C21)=O.OC(C=C1)=CC=C1C[CH](C%10=O)N%11.CSCC[CH](C(O)=O)N%10	14.8_7
C%11(COC1=CC=CC2=CC(C#N)=CC=C21)=O.OC(C=C1)=CC=C1C[CH](C%10=O)N%11.OC(C=C1)=CC=C1C[CH](C(O)=O)N%10	14.8_8
C%11(COC1=CC=CC2=CC(C#N)=CC=C21)=O.OC(C=C1)=CC=C1C[CH](C%10=O)N%11.O=C(O)[CH](CC1=C(F)C(F)=C(F)C(F)=C1F)N%10	14.8_9
C%10(COC1=CC=CC2=CC(C#N)=CC=C21)=O.O=C(O)CN%10	14.9_1
C%10(COC1=CC=CC2=CC(C#N)=CC=C21)=O.CC(C)[CH](C(O)=O)N%10	14.9_2
C%10(COC1=CC=CC2=CC(C#N)=CC=C21)=O.CC(C)C[CH](C(O)=O)N%10	14.9_3
C%10(COC1=CC=CC2=CC(C#N)=CC=C21)=O.C[CH](C(O)=O)N%10	14.9_4
C%10(COC1=CC=CC2=CC(C#N)=CC=C21)=O.O=C(O)[CH](CC1=CC=CC=C1)N%10	14.9_5
C%10(COC1=CC=CC2=CC(C#N)=CC=C21)=O.CC[CH](C)[CH](C(O)=O)N%10	14.9_6
C%10(COC1=CC=CC2=CC(C#N)=CC=C21)=O.CSCC[CH](C(O)=O)N%10	14.9_7
C%10(COC1=CC=CC2=CC(C#N)=CC=C21)=O.OC(C=C1)=CC=C1C[CH](C(O)=O)N%10	14.9_8
C%10(COC1=CC=CC2=CC(C#N)=CC=C21)=O.O=C(O)[CH](CC1=C(F)C(F)=C(F)C(F)=C1F)N%10	14.9_9
C%11(COC1=CC2=CC=CC=C2C=C1OC)=O.O=C%10CN%11.O=C(O)CN%10	15.1_1
C%11(COC1=CC2=CC=CC=C2C=C1OC)=O.O=C%10CN%11.CC(C)[CH](C(O)=O)N%10	15.1_2
C%11(COC1=CC2=CC=CC=C2C=C1OC)=O.O=C%10CN%11.CC(C)C[CH](C(O)=O)N%10	15.1_3
C%11(COC1=CC2=CC=CC=C2C=C1OC)=O.O=C%10CN%11.C[CH](C(O)=O)N%10	15.1_4
C%11(COC1=CC2=CC=CC=C2C=C1OC)=O.O=C%10CN%11.O=C(O)[CH](CC1=CC=CC=C1)N%10	15.1_5
C%11(COC1=CC2=CC=CC=C2C=C1OC)=O.O=C%10CN%11.CC[CH](C)[CH](C(O)=O)N%10	15.1_6
C%11(COC1=CC2=CC=CC=C2C=C1OC)=O.O=C%10CN%11.CSCC[CH](C(O)=O)N%10	15.1_7
C%11(COC1=CC2=CC=CC=C2C=C1OC)=O.O=C%10CN%11.OC(C=C1)=CC=C1C[CH](C(O)=O)N%10	15.1_8
C%11(COC1=CC2=CC=CC=C2C=C1OC)=O.O=C%10CN%11.O=C(O)[CH](CC1=C(F)C(F)=C(F)C(F)=C1F)N%10	15.1_9
C%11(COC1=CC2=CC=CC=C2C=C1OC)=O.CC(C)[CH](C%10=O)N%11.O=C(O)CN%10	15.2_1
C%11(COC1=CC2=CC=CC=C2C=C1OC)=O.CC(C)[CH](C%10=O)N%11.CC(C)[CH](C(O)=O)N%10	15.2_2
C%11(COC1=CC2=CC=CC=C2C=C1OC)=O.CC(C)[CH](C%10=O)N%11.CC(C)C[CH](C(O)=O)N%10	15.2_3
C%11(COC1=CC2=CC=CC=C2C=C1OC)=O.CC(C)[CH](C%10=O)N%11.C[CH](C(O)=O)N%10	15.2_4
C%11(COC1=CC2=CC=CC=C2C=C1OC)=O.CC(C)[CH](C%10=O)N%11.O=C(O)[CH](CC1=CC=CC=C1)N%10	15.2_5
C%11(COC1=CC2=CC=CC=C2C=C1OC)=O.CC(C)[CH](C%10=O)N%11.CC[CH](C)[CH](C(O)=	15.2_6

O)N%10	
C%11(COC1=CC2=CC=CC=C2C=C1OC)=O.CC(C)[CH](C%10=O)N%11.CSCC[CH](C(O)=O)N%10	15.2_7
C%11(COC1=CC2=CC=CC=C2C=C1OC)=O.CC(C)[CH](C%10=O)N%11.OC(C=C1)=CC=C1C[CH](C(O)=O)N%10	15.2_8
C%11(COC1=CC2=CC=CC=C2C=C1OC)=O.CC(C)[CH](C%10=O)N%11.O=C(O)[CH](CC1=C(F)C(F)=C(F)C(F)=C1F)N%10	15.2_9
C%11(COC1=CC2=CC=CC=C2C=C1OC)=O.CC(C)C[CH](C%10=O)N%11.O=C(O)CN%10	15.3_1
C%11(COC1=CC2=CC=CC=C2C=C1OC)=O.CC(C)C[CH](C%10=O)N%11.CC(C)[CH](C(O)=O)N%10	15.3_2
C%11(COC1=CC2=CC=CC=C2C=C1OC)=O.CC(C)C[CH](C%10=O)N%11.CC(C)C[CH](C(O)=O)N%10	15.3_3
C%11(COC1=CC2=CC=CC=C2C=C1OC)=O.CC(C)C[CH](C%10=O)N%11.C[CH](C(O)=O)N%10	15.3_4
C%11(COC1=CC2=CC=CC=C2C=C1OC)=O.CC(C)C[CH](C%10=O)N%11.O=C(O)[CH](CC1=CC=CC=C1)N%10	15.3_5
C%11(COC1=CC2=CC=CC=C2C=C1OC)=O.CC(C)C[CH](C%10=O)N%11.CC[CH](C)[CH](C(O)=O)N%10	15.3_6
C%11(COC1=CC2=CC=CC=C2C=C1OC)=O.CC(C)C[CH](C%10=O)N%11.CSCC[CH](C(O)=O)N%10	15.3_7
C%11(COC1=CC2=CC=CC=C2C=C1OC)=O.CC(C)C[CH](C%10=O)N%11.OC(C=C1)=CC=C1C[CH](C(O)=O)N%10	15.3_8
C%11(COC1=CC2=CC=CC=C2C=C1OC)=O.CC(C)C[CH](C%10=O)N%11.O=C(O)[CH](CC1=C(F)C(F)=C(F)C(F)=C1F)N%10	15.3_9
C%11(COC1=CC2=CC=CC=C2C=C1OC)=O.C[CH](C%10=O)N%11.O=C(O)CN%10	15.4_1
C%11(COC1=CC2=CC=CC=C2C=C1OC)=O.C[CH](C%10=O)N%11.CC(C)[CH](C(O)=O)N%10	15.4_2
C%11(COC1=CC2=CC=CC=C2C=C1OC)=O.C[CH](C%10=O)N%11.CC(C)C[CH](C(O)=O)N%10	15.4_3
C%11(COC1=CC2=CC=CC=C2C=C1OC)=O.C[CH](C%10=O)N%11.C[CH](C(O)=O)N%10	15.4_4
C%11(COC1=CC2=CC=CC=C2C=C1OC)=O.C[CH](C%10=O)N%11.O=C(O)[CH](CC1=CC=CC=C1)N%10	15.4_5
C%11(COC1=CC2=CC=CC=C2C=C1OC)=O.C[CH](C%10=O)N%11.CC[CH](C)[CH](C(O)=O)N%10	15.4_6
C%11(COC1=CC2=CC=CC=C2C=C1OC)=O.C[CH](C%10=O)N%11.CSCC[CH](C(O)=O)N%10	15.4_7
C%11(COC1=CC2=CC=CC=C2C=C1OC)=O.C[CH](C%10=O)N%11.OC(C=C1)=CC=C1C[CH](C(O)=O)N%10	15.4_8
C%11(COC1=CC2=CC=CC=C2C=C1OC)=O.C[CH](C%10=O)N%11.O=C(O)[CH](CC1=C(F)C(F)=C(F)C(F)=C1F)N%10	15.4_9
C%11(COC1=CC2=CC=CC=C2C=C1OC)=O.O=C%10[CH](CC1=CC=CC=C1)N%11.O=C(O)CN%10	15.5_1
C%11(COC1=CC2=CC=CC=C2C=C1OC)=O.O=C%10[CH](CC1=CC=CC=C1)N%11.CC(C)[CH](C(O)=O)N%10	15.5_2
C%11(COC1=CC2=CC=CC=C2C=C1OC)=O.O=C%10[CH](CC1=CC=CC=C1)N%11.CC(C)C[CH](C(O)=O)N%10	15.5_3
C%11(COC1=CC2=CC=CC=C2C=C1OC)=O.O=C%10[CH](CC1=CC=CC=C1)N%11.C[CH](C(O)=O)N%10	15.5_4
C%11(COC1=CC2=CC=CC=C2C=C1OC)=O.O=C%10[CH](CC1=CC=CC=C1)N%11.O=C(O)[CH](CC1=CC=CC=C1)N%10	15.5_5
C%11(COC1=CC2=CC=CC=C2C=C1OC)=O.O=C%10[CH](CC1=CC=CC=C1)N%11.CC[CH](C)[CH](C(O)=O)N%10	15.5_6

C%11(COC1=CC2=CC=CC=C2C=C1OC)=O.O=C%10[CH](CC1=CC=CC=C1)N%11.CSCC[CH](C(O)=O)N%10	15.5_7
C%11(COC1=CC2=CC=CC=C2C=C1OC)=O.O=C%10[CH](CC1=CC=CC=C1)N%11.OC(C=C1)=CC=C1C[CH](C(O)=O)N%10	15.5_8
C%11(COC1=CC2=CC=CC=C2C=C1OC)=O.O=C%10[CH](CC1=CC=CC=C1)N%11.O=C(O)[CH](CC1=C(F)C(F)=C(F)C(F)=C1F)N%10	15.5_9
C%11(COC1=CC2=CC=CC=C2C=C1OC)=O.CC[CH](C)[CH](C%10=O)N%11.O=C(O)CN%10	15.6_1
C%11(COC1=CC2=CC=CC=C2C=C1OC)=O.CC[CH](C)[CH](C%10=O)N%11.CC(C)[CH](C(O)=O)N%10	15.6_2
C%11(COC1=CC2=CC=CC=C2C=C1OC)=O.CC[CH](C)[CH](C%10=O)N%11.CC(C)C[CH](C(O)=O)N%10	15.6_3
C%11(COC1=CC2=CC=CC=C2C=C1OC)=O.CC[CH](C)[CH](C%10=O)N%11.C[CH](C(O)=O)N%10	15.6_4
C%11(COC1=CC2=CC=CC=C2C=C1OC)=O.CC[CH](C)[CH](C%10=O)N%11.O=C(O)[CH](CC1=CC=CC=C1)N%10	15.6_5
C%11(COC1=CC2=CC=CC=C2C=C1OC)=O.CC[CH](C)[CH](C%10=O)N%11.CC[CH](C)[CH](C(O)=O)N%10	15.6_6
C%11(COC1=CC2=CC=CC=C2C=C1OC)=O.CC[CH](C)[CH](C%10=O)N%11.CSCC[CH](C(O)=O)N%10	15.6_7
C%11(COC1=CC2=CC=CC=C2C=C1OC)=O.CC[CH](C)[CH](C%10=O)N%11.OC(C=C1)=CC=C1C[CH](C(O)=O)N%10	15.6_8
C%11(COC1=CC2=CC=CC=C2C=C1OC)=O.CC[CH](C)[CH](C%10=O)N%11.O=C(O)[CH](CC1=C(F)C(F)=C(F)C(F)=C1F)N%10	15.6_9
C%11(COC1=CC2=CC=CC=C2C=C1OC)=O.CSCC[CH](C%10=O)N%11.O=C(O)CN%10	15.7_1
C%11(COC1=CC2=CC=CC=C2C=C1OC)=O.CSCC[CH](C%10=O)N%11.CC(C)[CH](C(O)=O)N%10	15.7_2
C%11(COC1=CC2=CC=CC=C2C=C1OC)=O.CSCC[CH](C%10=O)N%11.CC(C)C[CH](C(O)=O)N%10	15.7_3
C%11(COC1=CC2=CC=CC=C2C=C1OC)=O.CSCC[CH](C%10=O)N%11.C[CH](C(O)=O)N%10	15.7_4
C%11(COC1=CC2=CC=CC=C2C=C1OC)=O.CSCC[CH](C%10=O)N%11.O=C(O)[CH](CC1=CC=CC=C1)N%10	15.7_5
C%11(COC1=CC2=CC=CC=C2C=C1OC)=O.CSCC[CH](C%10=O)N%11.CC[CH](C)[CH](C(O)=O)N%10	15.7_6
C%11(COC1=CC2=CC=CC=C2C=C1OC)=O.CSCC[CH](C%10=O)N%11.CSCC[CH](C(O)=O)N%10	15.7_7
C%11(COC1=CC2=CC=CC=C2C=C1OC)=O.CSCC[CH](C%10=O)N%11.OC(C=C1)=CC=C1C[CH](C(O)=O)N%10	15.7_8
C%11(COC1=CC2=CC=CC=C2C=C1OC)=O.CSCC[CH](C%10=O)N%11.O=C(O)[CH](CC1=C(F)C(F)=C(F)C(F)=C1F)N%10	15.7_9
C%11(COC1=CC2=CC=CC=C2C=C1OC)=O.OC(C=C1)=CC=C1C[CH](C%10=O)N%11.O=C(O)CN%10	15.8_1
C%11(COC1=CC2=CC=CC=C2C=C1OC)=O.OC(C=C1)=CC=C1C[CH](C%10=O)N%11.CC(C)[CH](C(O)=O)N%10	15.8_2
C%11(COC1=CC2=CC=CC=C2C=C1OC)=O.OC(C=C1)=CC=C1C[CH](C%10=O)N%11.CC(C)C[CH](C(O)=O)N%10	15.8_3
C%11(COC1=CC2=CC=CC=C2C=C1OC)=O.OC(C=C1)=CC=C1C[CH](C%10=O)N%11.C[CH](C(O)=O)N%10	15.8_4
C%11(COC1=CC2=CC=CC=C2C=C1OC)=O.OC(C=C1)=CC=C1C[CH](C%10=O)N%11.O=C(O)[CH](CC1=CC=CC=C1)N%10	15.8_5

C%11(COC1=CC2=CC=CC=C2C=C1OC)=O.OC(C=C1)=CC=C1C[CH](C%10=O)N%11.CC[CH](C)[CH](C(O)=O)N%10	15.8_6
C%11(COC1=CC2=CC=CC=C2C=C1OC)=O.OC(C=C1)=CC=C1C[CH](C%10=O)N%11.CSCC[CH](C(O)=O)N%10	15.8_7
C%11(COC1=CC2=CC=CC=C2C=C1OC)=O.OC(C=C1)=CC=C1C[CH](C%10=O)N%11.OC(C=C1)=CC=C1C[CH](C(O)=O)N%10	15.8_8
C%11(COC1=CC2=CC=CC=C2C=C1OC)=O.OC(C=C1)=CC=C1C[CH](C%10=O)N%11.O=C(O)[CH](CC1=C(F)C(F)=C(F)C(F)=C1F)N%10	15.8_9
C%10(COC1=CC2=CC=CC=C2C=C1OC)=O.O=C(O)CN%10	15.9_1
C%10(COC1=CC2=CC=CC=C2C=C1OC)=O.CC(C)[CH](C(O)=O)N%10	15.9_2
C%10(COC1=CC2=CC=CC=C2C=C1OC)=O.CC(C)C[CH](C(O)=O)N%10	15.9_3
C%10(COC1=CC2=CC=CC=C2C=C1OC)=O.C[CH](C(O)=O)N%10	15.9_4
C%10(COC1=CC2=CC=CC=C2C=C1OC)=O.O=C(O)[CH](CC1=CC=CC=C1)N%10	15.9_5
C%10(COC1=CC2=CC=CC=C2C=C1OC)=O.CC[CH](C)[CH](C(O)=O)N%10	15.9_6
C%10(COC1=CC2=CC=CC=C2C=C1OC)=O.CSCC[CH](C(O)=O)N%10	15.9_7
C%10(COC1=CC2=CC=CC=C2C=C1OC)=O.OC(C=C1)=CC=C1C[CH](C(O)=O)N%10	15.9_8
C%10(COC1=CC2=CC=CC=C2C=C1OC)=O.O=C(O)[CH](CC1=C(F)C(F)=C(F)C(F)=C1F)N%10	15.9_9
C%11(COC1=CC2=CC(OC)=CC=C2C=C1)=O.O=C%10CN%11.O=C(O)CN%10	16.1_1
C%11(COC1=CC2=CC(OC)=CC=C2C=C1)=O.O=C%10CN%11.CC(C)[CH](C(O)=O)N%10	16.1_2
C%11(COC1=CC2=CC(OC)=CC=C2C=C1)=O.O=C%10CN%11.CC(C)C[CH](C(O)=O)N%10	16.1_3
C%11(COC1=CC2=CC(OC)=CC=C2C=C1)=O.O=C%10CN%11.C[CH](C(O)=O)N%10	16.1_4
C%11(COC1=CC2=CC(OC)=CC=C2C=C1)=O.O=C%10CN%11.O=C(O)[CH](CC1=CC=CC=C1)N%10	16.1_5
C%11(COC1=CC2=CC(OC)=CC=C2C=C1)=O.O=C%10CN%11.CC[CH](C)[CH](C(O)=O)N%10	16.1_6
C%11(COC1=CC2=CC(OC)=CC=C2C=C1)=O.O=C%10CN%11.CSCC[CH](C(O)=O)N%10	16.1_7
C%11(COC1=CC2=CC(OC)=CC=C2C=C1)=O.O=C%10CN%11.OC(C=C1)=CC=C1C[CH](C(O)=O)N%10	16.1_8
C%11(COC1=CC2=CC(OC)=CC=C2C=C1)=O.O=C%10CN%11.O=C(O)[CH](CC1=C(F)C(F)=C(F)C(F)=C1F)N%10	16.1_9
C%11(COC1=CC2=CC(OC)=CC=C2C=C1)=O.CC(C)[CH](C%10=O)N%11.O=C(O)CN%10	16.2_1
C%11(COC1=CC2=CC(OC)=CC=C2C=C1)=O.CC(C)[CH](C%10=O)N%11.CC(C)[CH](C(O)=O)N%10	16.2_2
C%11(COC1=CC2=CC(OC)=CC=C2C=C1)=O.CC(C)[CH](C%10=O)N%11.CC(C)C[CH](C(O)=O)N%10	16.2_3
C%11(COC1=CC2=CC(OC)=CC=C2C=C1)=O.CC(C)[CH](C%10=O)N%11.C[CH](C(O)=O)N%10	16.2_4
C%11(COC1=CC2=CC(OC)=CC=C2C=C1)=O.CC(C)[CH](C%10=O)N%11.O=C(O)[CH](CC1=CC=CC=C1)N%10	16.2_5
C%11(COC1=CC2=CC(OC)=CC=C2C=C1)=O.CC(C)[CH](C%10=O)N%11.CC[CH](C)[CH](C(O)=O)N%10	16.2_6
C%11(COC1=CC2=CC(OC)=CC=C2C=C1)=O.CC(C)[CH](C%10=O)N%11.CSCC[CH](C(O)=O)N%10	16.2_7
C%11(COC1=CC2=CC(OC)=CC=C2C=C1)=O.CC(C)[CH](C%10=O)N%11.OC(C=C1)=CC=C1C[CH](C(O)=O)N%10	16.2_8
C%11(COC1=CC2=CC(OC)=CC=C2C=C1)=O.CC(C)[CH](C%10=O)N%11.O=C(O)[CH](CC1=C(F)C(F)=C(F)C(F)=C1F)N%10	16.2_9
C%11(COC1=CC2=CC(OC)=CC=C2C=C1)=O.CC(C)C[CH](C%10=O)N%11.O=C(O)CN%10	16.3_1
C%11(COC1=CC2=CC(OC)=CC=C2C=C1)=O.CC(C)C[CH](C%10=O)N%11.CC(C)[CH](C(O)=O)	16.3_2

N%10	
C%11(COC1=CC2=CC(OC)=CC=C2C=C1)=O.CC(C)C[CH](C%10=O)N%11.CC(C)C[CH](C(O)=O)N%10	16.3_3
C%11(COC1=CC2=CC(OC)=CC=C2C=C1)=O.CC(C)C[CH](C%10=O)N%11.C[CH](C(O)=O)N%10	16.3_4
C%11(COC1=CC2=CC(OC)=CC=C2C=C1)=O.CC(C)C[CH](C%10=O)N%11.O=C(O)[CH](CC1=CC=CC=C1)N%10	16.3_5
C%11(COC1=CC2=CC(OC)=CC=C2C=C1)=O.CC(C)C[CH](C%10=O)N%11.CC[CH](C)[CH](C(O)=O)N%10	16.3_6
C%11(COC1=CC2=CC(OC)=CC=C2C=C1)=O.CC(C)C[CH](C%10=O)N%11.CSCC[CH](C(O)=O)N%10	16.3_7
C%11(COC1=CC2=CC(OC)=CC=C2C=C1)=O.CC(C)C[CH](C%10=O)N%11.OC(C=C1)=CC=C1C[CH](C(O)=O)N%10	16.3_8
C%11(COC1=CC2=CC(OC)=CC=C2C=C1)=O.CC(C)C[CH](C%10=O)N%11.O=C(O)[CH](CC1=C(F)C(F)=C(F)C(F)=C1F)N%10	16.3_9
C%11(COC1=CC2=CC(OC)=CC=C2C=C1)=O.C[CH](C%10=O)N%11.O=C(O)CN%10	16.4_1
C%11(COC1=CC2=CC(OC)=CC=C2C=C1)=O.C[CH](C%10=O)N%11.CC(C)[CH](C(O)=O)N%10	16.4_2
C%11(COC1=CC2=CC(OC)=CC=C2C=C1)=O.C[CH](C%10=O)N%11.CC(C)C[CH](C(O)=O)N%10	16.4_3
C%11(COC1=CC2=CC(OC)=CC=C2C=C1)=O.C[CH](C%10=O)N%11.C[CH](C(O)=O)N%10	16.4_4
C%11(COC1=CC2=CC(OC)=CC=C2C=C1)=O.C[CH](C%10=O)N%11.O=C(O)[CH](CC1=CC=CC=C1)N%10	16.4_5
C%11(COC1=CC2=CC(OC)=CC=C2C=C1)=O.C[CH](C%10=O)N%11.CC[CH](C)[CH](C(O)=O)N%10	16.4_6
C%11(COC1=CC2=CC(OC)=CC=C2C=C1)=O.C[CH](C%10=O)N%11.CSCC[CH](C(O)=O)N%10	16.4_7
C%11(COC1=CC2=CC(OC)=CC=C2C=C1)=O.C[CH](C%10=O)N%11.OC(C=C1)=CC=C1C[CH](C(O)=O)N%10	16.4_8
C%11(COC1=CC2=CC(OC)=CC=C2C=C1)=O.C[CH](C%10=O)N%11.O=C(O)[CH](CC1=C(F)C(F)=C(F)C(F)=C1F)N%10	16.4_9
C%11(COC1=CC2=CC(OC)=CC=C2C=C1)=O.O=C%10[CH](CC1=CC=CC=C1)N%11.O=C(O)CN%10	16.5_1
C%11(COC1=CC2=CC(OC)=CC=C2C=C1)=O.O=C%10[CH](CC1=CC=CC=C1)N%11.CC(C)[CH](C(O)=O)N%10	16.5_2
C%11(COC1=CC2=CC(OC)=CC=C2C=C1)=O.O=C%10[CH](CC1=CC=CC=C1)N%11.CC(C)C[CH](C(O)=O)N%10	16.5_3
C%11(COC1=CC2=CC(OC)=CC=C2C=C1)=O.O=C%10[CH](CC1=CC=CC=C1)N%11.C[CH](C(O)=O)N%10	16.5_4
C%11(COC1=CC2=CC(OC)=CC=C2C=C1)=O.O=C%10[CH](CC1=CC=CC=C1)N%11.O=C(O)[CH](CC1=CC=CC=C1)N%10	16.5_5
C%11(COC1=CC2=CC(OC)=CC=C2C=C1)=O.O=C%10[CH](CC1=CC=CC=C1)N%11.CC[CH](C)[CH](C(O)=O)N%10	16.5_6
C%11(COC1=CC2=CC(OC)=CC=C2C=C1)=O.O=C%10[CH](CC1=CC=CC=C1)N%11.CSCC[CH](C(O)=O)N%10	16.5_7
C%11(COC1=CC2=CC(OC)=CC=C2C=C1)=O.O=C%10[CH](CC1=CC=CC=C1)N%11.OC(C=C1)=CC=C1C[CH](C(O)=O)N%10	16.5_8
C%11(COC1=CC2=CC(OC)=CC=C2C=C1)=O.O=C%10[CH](CC1=CC=CC=C1)N%11.O=C(O)[CH](CC1=C(F)C(F)=C(F)C(F)=C1F)N%10	16.5_9
C%11(COC1=CC2=CC(OC)=CC=C2C=C1)=O.CC[CH](C)[CH](C%10=O)N%11.O=C(O)CN%10	16.6_1

C%11(COC1=CC2=CC(OC)=CC=C2C=C1)=O.CC[CH](C)[CH](C%10=O)N%11.CC(C)[CH](C(O)=O)N%10	16.6_2
C%11(COC1=CC2=CC(OC)=CC=C2C=C1)=O.CC[CH](C)[CH](C%10=O)N%11.CC(C)C[CH](C(O)=O)N%10	16.6_3
C%11(COC1=CC2=CC(OC)=CC=C2C=C1)=O.CC[CH](C)[CH](C%10=O)N%11.C[CH](C(O)=O)N%10	16.6_4
C%11(COC1=CC2=CC(OC)=CC=C2C=C1)=O.CC[CH](C)[CH](C%10=O)N%11.O=C(O)[CH](CC1=CC=CC=C1)N%10	16.6_5
C%11(COC1=CC2=CC(OC)=CC=C2C=C1)=O.CC[CH](C)[CH](C%10=O)N%11.CC[CH](C)[CH](C(O)=O)N%10	16.6_6
C%11(COC1=CC2=CC(OC)=CC=C2C=C1)=O.CC[CH](C)[CH](C%10=O)N%11.CSCC[CH](C(O)=O)N%10	16.6_7
C%11(COC1=CC2=CC(OC)=CC=C2C=C1)=O.CC[CH](C)[CH](C%10=O)N%11.OC(C=C1)=CC=C1C[CH](C(O)=O)N%10	16.6_8
C%11(COC1=CC2=CC(OC)=CC=C2C=C1)=O.CC[CH](C)[CH](C%10=O)N%11.O=C(O)[CH](CC1=C(F)C(F)=C(F)C(F)=C1F)N%10	16.6_9
C%11(COC1=CC2=CC(OC)=CC=C2C=C1)=O.CSCC[CH](C%10=O)N%11.O=C(O)CN%10	16.7_1
C%11(COC1=CC2=CC(OC)=CC=C2C=C1)=O.CSCC[CH](C%10=O)N%11.CC(C)[CH](C(O)=O)N%10	16.7_2
C%11(COC1=CC2=CC(OC)=CC=C2C=C1)=O.CSCC[CH](C%10=O)N%11.CC(C)C[CH](C(O)=O)N%10	16.7_3
C%11(COC1=CC2=CC(OC)=CC=C2C=C1)=O.CSCC[CH](C%10=O)N%11.C[CH](C(O)=O)N%10	16.7_4
C%11(COC1=CC2=CC(OC)=CC=C2C=C1)=O.CSCC[CH](C%10=O)N%11.O=C(O)[CH](CC1=CC=CC=C1)N%10	16.7_5
C%11(COC1=CC2=CC(OC)=CC=C2C=C1)=O.CSCC[CH](C%10=O)N%11.CC[CH](C)[CH](C(O)=O)N%10	16.7_6
C%11(COC1=CC2=CC(OC)=CC=C2C=C1)=O.CSCC[CH](C%10=O)N%11.CSCC[CH](C(O)=O)N%10	16.7_7
C%11(COC1=CC2=CC(OC)=CC=C2C=C1)=O.CSCC[CH](C%10=O)N%11.OC(C=C1)=CC=C1C[CH](C(O)=O)N%10	16.7_8
C%11(COC1=CC2=CC(OC)=CC=C2C=C1)=O.CSCC[CH](C%10=O)N%11.O=C(O)[CH](CC1=C(F)C(F)=C(F)C(F)=C1F)N%10	16.7_9
C%11(COC1=CC2=CC(OC)=CC=C2C=C1)=O.OC(C=C1)=CC=C1C[CH](C%10=O)N%11.O=C(O)CN%10	16.8_1
C%11(COC1=CC2=CC(OC)=CC=C2C=C1)=O.OC(C=C1)=CC=C1C[CH](C%10=O)N%11.CC(C)[CH](C(O)=O)N%10	16.8_2
C%11(COC1=CC2=CC(OC)=CC=C2C=C1)=O.OC(C=C1)=CC=C1C[CH](C%10=O)N%11.CC(C)C[CH](C(O)=O)N%10	16.8_3
C%11(COC1=CC2=CC(OC)=CC=C2C=C1)=O.OC(C=C1)=CC=C1C[CH](C%10=O)N%11.C[CH](C(O)=O)N%10	16.8_4
C%11(COC1=CC2=CC(OC)=CC=C2C=C1)=O.OC(C=C1)=CC=C1C[CH](C%10=O)N%11.O=C(O)[CH](CC1=CC=CC=C1)N%10	16.8_5
C%11(COC1=CC2=CC(OC)=CC=C2C=C1)=O.OC(C=C1)=CC=C1C[CH](C%10=O)N%11.CC[CH](C)[CH](C(O)=O)N%10	16.8_6
C%11(COC1=CC2=CC(OC)=CC=C2C=C1)=O.OC(C=C1)=CC=C1C[CH](C%10=O)N%11.CSCC[CH](C(O)=O)N%10	16.8_7
C%11(COC1=CC2=CC(OC)=CC=C2C=C1)=O.OC(C=C1)=CC=C1C[CH](C%10=O)N%11.OC(C=C1)=CC=C1C[CH](C(O)=O)N%10	16.8_8
C%11(COC1=CC2=CC(OC)=CC=C2C=C1)=O.OC(C=C1)=CC=C1C[CH](C%10=O)N%11.O=C(O)	16.8_9

[CH](CC1=C(F)C(F)=C(F)C(F)=C1F)N%10	
C%10(COC1=CC2=CC(OC)=CC=C2C=C1)=O.O=C(O)CN%10	16.9_1
C%10(COC1=CC2=CC(OC)=CC=C2C=C1)=O.CC(C)[CH](C(O)=O)N%10	16.9_2
C%10(COC1=CC2=CC(OC)=CC=C2C=C1)=O.CC(C)C[CH](C(O)=O)N%10	16.9_3
C%10(COC1=CC2=CC(OC)=CC=C2C=C1)=O.C[CH](C(O)=O)N%10	16.9_4
C%10(COC1=CC2=CC(OC)=CC=C2C=C1)=O.O=C(O)[CH](CC1=CC=CC=C1)N%10	16.9_5
C%10(COC1=CC2=CC(OC)=CC=C2C=C1)=O.CC[CH](C)[CH](C(O)=O)N%10	16.9_6
C%10(COC1=CC2=CC(OC)=CC=C2C=C1)=O.CSCC[CH](C(O)=O)N%10	16.9_7
C%10(COC1=CC2=CC(OC)=CC=C2C=C1)=O.OC(C=C1)=CC=C1C[CH](C(O)=O)N%10	16.9_8
C%10(COC1=CC2=CC(OC)=CC=C2C=C1)=O.O=C(O)[CH](CC1=C(F)C(F)=C(F)C(F)=C1F)N%10	16.9_9
C%11(COC1=CC2=CC=CC=C2C=C1)=O.O=C%10CN%11.O=C(O)CN%10	17.1_1
C%11(COC1=CC2=CC=CC=C2C=C1)=O.O=C%10CN%11.CC(C)[CH](C(O)=O)N%10	17.1_2
C%11(COC1=CC2=CC=CC=C2C=C1)=O.O=C%10CN%11.CC(C)C[CH](C(O)=O)N%10	17.1_3
C%11(COC1=CC2=CC=CC=C2C=C1)=O.O=C%10CN%11.C[CH](C(O)=O)N%10	17.1_4
C%11(COC1=CC2=CC=CC=C2C=C1)=O.O=C%10CN%11.O=C(O)[CH](CC1=CC=CC=C1)N%10	17.1_5
C%11(COC1=CC2=CC=CC=C2C=C1)=O.O=C%10CN%11.CC[CH](C)[CH](C(O)=O)N%10	17.1_6
C%11(COC1=CC2=CC=CC=C2C=C1)=O.O=C%10CN%11.CSCC[CH](C(O)=O)N%10	17.1_7
C%11(COC1=CC2=CC=CC=C2C=C1)=O.O=C%10CN%11.OC(C=C1)=CC=C1C[CH](C(O)=O)N%<br>10	17.1_8
C%11(COC1=CC2=CC=CC=C2C=C1)=O.O=C%10CN%11.O=C(O)[CH](CC1=C(F)C(F)=C(F)C(F)<br>=C1F)N%10	17.1_9
C%11(COC1=CC2=CC=CC=C2C=C1)=O.CC(C)[CH](C%10=O)N%11.O=C(O)CN%10	17.2_1
C%11(COC1=CC2=CC=CC=C2C=C1)=O.CC(C)[CH](C%10=O)N%11.CC(C)[CH](C(O)=O)N%10	17.2_2
C%11(COC1=CC2=CC=CC=C2C=C1)=O.CC(C)[CH](C%10=O)N%11.CC(C)C[CH](C(O)=O)N%1<br>0	17.2_3
C%11(COC1=CC2=CC=CC=C2C=C1)=O.CC(C)[CH](C%10=O)N%11.C[CH](C(O)=O)N%10	17.2_4
C%11(COC1=CC2=CC=CC=C2C=C1)=O.CC(C)[CH](C%10=O)N%11.O=C(O)[CH](CC1=CC=CC<br>=C1)N%10	17.2_5
C%11(COC1=CC2=CC=CC=C2C=C1)=O.CC(C)[CH](C%10=O)N%11.CC[CH](C)[CH](C(O)=O)N<br>%10	17.2_6
C%11(COC1=CC2=CC=CC=C2C=C1)=O.CC(C)[CH](C%10=O)N%11.CSCC[CH](C(O)=O)N%10	17.2_7
C%11(COC1=CC2=CC=CC=C2C=C1)=O.CC(C)[CH](C%10=O)N%11.OC(C=C1)=CC=C1C[CH](C<br>(O)=O)N%10	17.2_8
C%11(COC1=CC2=CC=CC=C2C=C1)=O.CC(C)[CH](C%10=O)N%11.O=C(O)[CH](CC1=C(F)C(F<br>)=C(F)C(F)=C1F)N%10	17.2_9
C%11(COC1=CC2=CC=CC=C2C=C1)=O.CC(C)C[CH](C%10=O)N%11.O=C(O)CN%10	17.3_1
C%11(COC1=CC2=CC=CC=C2C=C1)=O.CC(C)C[CH](C%10=O)N%11.CC(C)[CH](C(O)=O)N%1<br>0	17.3_2
C%11(COC1=CC2=CC=CC=C2C=C1)=O.CC(C)C[CH](C%10=O)N%11.CC(C)C[CH](C(O)=O)N%<br>10	17.3_3
C%11(COC1=CC2=CC=CC=C2C=C1)=O.CC(C)C[CH](C%10=O)N%11.C[CH](C(O)=O)N%10	17.3_4
C%11(COC1=CC2=CC=CC=C2C=C1)=O.CC(C)C[CH](C%10=O)N%11.O=C(O)[CH](CC1=CC=C<br>C=C1)N%10	17.3_5
C%11(COC1=CC2=CC=CC=C2C=C1)=O.CC(C)C[CH](C%10=O)N%11.CC[CH](C)[CH](C(O)=O)<br>N%10	17.3_6
C%11(COC1=CC2=CC=CC=C2C=C1)=O.CC(C)C[CH](C%10=O)N%11.CSCC[CH](C(O)=O)N%1	17.3_7

0	
C%11(COC1=CC2=CC=CC=C2C=C1)=O.CC(C)C[CH](C%10=O)N%11.OC(C=C1)=CC=C1C[CH](C(O)=O)N%10	17.3_8
C%11(COC1=CC2=CC=CC=C2C=C1)=O.CC(C)C[CH](C%10=O)N%11.O=C(O)[CH](CC1=C(F)C(F)=C(F)C(F)=C1F)N%10	17.3_9
C%11(COC1=CC2=CC=CC=C2C=C1)=O.C[CH](C%10=O)N%11.O=C(O)CN%10	17.4_1
C%11(COC1=CC2=CC=CC=C2C=C1)=O.C[CH](C%10=O)N%11.CC(C)[CH](C(O)=O)N%10	17.4_2
C%11(COC1=CC2=CC=CC=C2C=C1)=O.C[CH](C%10=O)N%11.CC(C)C[CH](C(O)=O)N%10	17.4_3
C%11(COC1=CC2=CC=CC=C2C=C1)=O.C[CH](C%10=O)N%11.C[CH](C(O)=O)N%10	17.4_4
C%11(COC1=CC2=CC=CC=C2C=C1)=O.C[CH](C%10=O)N%11.O=C(O)[CH](CC1=CC=CC=C1)N%10	17.4_5
C%11(COC1=CC2=CC=CC=C2C=C1)=O.C[CH](C%10=O)N%11.CC[CH](C)[CH](C(O)=O)N%10	17.4_6
C%11(COC1=CC2=CC=CC=C2C=C1)=O.C[CH](C%10=O)N%11.CSCC[CH](C(O)=O)N%10	17.4_7
C%11(COC1=CC2=CC=CC=C2C=C1)=O.C[CH](C%10=O)N%11.OC(C=C1)=CC=C1C[CH](C(O)=O)N%10	17.4_8
C%11(COC1=CC2=CC=CC=C2C=C1)=O.C[CH](C%10=O)N%11.O=C(O)[CH](CC1=C(F)C(F)=C(F)C(F)=C1F)N%10	17.4_9
C%11(COC1=CC2=CC=CC=C2C=C1)=O.O=C%10[CH](CC1=CC=CC=C1)N%11.O=C(O)CN%10	17.5_1
C%11(COC1=CC2=CC=CC=C2C=C1)=O.O=C%10[CH](CC1=CC=CC=C1)N%11.CC(C)[CH](C(O)=O)N%10	17.5_2
C%11(COC1=CC2=CC=CC=C2C=C1)=O.O=C%10[CH](CC1=CC=CC=C1)N%11.CC(C)C[CH](C(O)=O)N%10	17.5_3
C%11(COC1=CC2=CC=CC=C2C=C1)=O.O=C%10[CH](CC1=CC=CC=C1)N%11.C[CH](C(O)=O)N%10	17.5_4
C%11(COC1=CC2=CC=CC=C2C=C1)=O.O=C%10[CH](CC1=CC=CC=C1)N%11.O=C(O)[CH](CC1=CC=CC=C1)N%10	17.5_5
C%11(COC1=CC2=CC=CC=C2C=C1)=O.O=C%10[CH](CC1=CC=CC=C1)N%11.CC[CH](C)[CH](C(O)=O)N%10	17.5_6
C%11(COC1=CC2=CC=CC=C2C=C1)=O.O=C%10[CH](CC1=CC=CC=C1)N%11.CSCC[CH](C(O)=O)N%10	17.5_7
C%11(COC1=CC2=CC=CC=C2C=C1)=O.O=C%10[CH](CC1=CC=CC=C1)N%11.OC(C=C1)=CC=C1C[CH](C(O)=O)N%10	17.5_8
C%11(COC1=CC2=CC=CC=C2C=C1)=O.O=C%10[CH](CC1=CC=CC=C1)N%11.O=C(O)[CH](CC1=C(F)C(F)=C(F)C(F)=C1F)N%10	17.5_9
C%11(COC1=CC2=CC=CC=C2C=C1)=O.CC[CH](C)[CH](C%10=O)N%11.O=C(O)CN%10	17.6_1
C%11(COC1=CC2=CC=CC=C2C=C1)=O.CC[CH](C)[CH](C%10=O)N%11.CC(C)[CH](C(O)=O)N%10	17.6_2
C%11(COC1=CC2=CC=CC=C2C=C1)=O.CC[CH](C)[CH](C%10=O)N%11.CC(C)C[CH](C(O)=O)N%10	17.6_3
C%11(COC1=CC2=CC=CC=C2C=C1)=O.CC[CH](C)[CH](C%10=O)N%11.C[CH](C(O)=O)N%10	17.6_4
C%11(COC1=CC2=CC=CC=C2C=C1)=O.CC[CH](C)[CH](C%10=O)N%11.O=C(O)[CH](CC1=CC=CC=C1)N%10	17.6_5
C%11(COC1=CC2=CC=CC=C2C=C1)=O.CC[CH](C)[CH](C%10=O)N%11.CC[CH](C)[CH](C(O)=O)N%10	17.6_6
C%11(COC1=CC2=CC=CC=C2C=C1)=O.CC[CH](C)[CH](C%10=O)N%11.CSCC[CH](C(O)=O)N%10	17.6_7
C%11(COC1=CC2=CC=CC=C2C=C1)=O.CC[CH](C)[CH](C%10=O)N%11.OC(C=C1)=CC=C1C[CH](C(O)=O)N%10	17.6_8

C%11(COC1=CC2=CC=CC=C2C=C1)=O.CC[CH](C)[CH](C%10=O)N%11.O=C(O)[CH](CC1=C(F)C(F)=C(F)C(F)=C1F)N%10	17.6_9
C%11(COC1=CC2=CC=CC=C2C=C1)=O.CSCC[CH](C%10=O)N%11.O=C(O)CN%10	17.7_1
C%11(COC1=CC2=CC=CC=C2C=C1)=O.CSCC[CH](C%10=O)N%11.CC(C)[CH](C(O)=O)N%10	17.7_2
C%11(COC1=CC2=CC=CC=C2C=C1)=O.CSCC[CH](C%10=O)N%11.CC(C)C[CH](C(O)=O)N%10	17.7_3
C%11(COC1=CC2=CC=CC=C2C=C1)=O.CSCC[CH](C%10=O)N%11.C[CH](C(O)=O)N%10	17.7_4
C%11(COC1=CC2=CC=CC=C2C=C1)=O.CSCC[CH](C%10=O)N%11.O=C(O)[CH](CC1=CC=CC=C1)N%10	17.7_5
C%11(COC1=CC2=CC=CC=C2C=C1)=O.CSCC[CH](C%10=O)N%11.CC[CH](C)[CH](C(O)=O)N%10	17.7_6
C%11(COC1=CC2=CC=CC=C2C=C1)=O.CSCC[CH](C%10=O)N%11.CSCC[CH](C(O)=O)N%10	17.7_7
C%11(COC1=CC2=CC=CC=C2C=C1)=O.CSCC[CH](C%10=O)N%11.OC(C=C1)=CC=C1C[CH](C(O)=O)N%10	17.7_8
C%11(COC1=CC2=CC=CC=C2C=C1)=O.CSCC[CH](C%10=O)N%11.O=C(O)[CH](CC1=C(F)C(F)=C(F)C(F)=C1F)N%10	17.7_9
C%11(COC1=CC2=CC=CC=C2C=C1)=O.OC(C=C1)=CC=C1C[CH](C%10=O)N%11.O=C(O)CN%10	17.8_1
C%11(COC1=CC2=CC=CC=C2C=C1)=O.OC(C=C1)=CC=C1C[CH](C%10=O)N%11.CC(C)[CH](C(O)=O)N%10	17.8_2
C%11(COC1=CC2=CC=CC=C2C=C1)=O.OC(C=C1)=CC=C1C[CH](C%10=O)N%11.CC(C)C[CH](C(O)=O)N%10	17.8_3
C%11(COC1=CC2=CC=CC=C2C=C1)=O.OC(C=C1)=CC=C1C[CH](C%10=O)N%11.C[CH](C(O)=O)N%10	17.8_4
C%11(COC1=CC2=CC=CC=C2C=C1)=O.OC(C=C1)=CC=C1C[CH](C%10=O)N%11.O=C(O)[CH](CC1=CC=CC=C1)N%10	17.8_5
C%11(COC1=CC2=CC=CC=C2C=C1)=O.OC(C=C1)=CC=C1C[CH](C%10=O)N%11.CC[CH](C)[CH](C(O)=O)N%10	17.8_6
C%11(COC1=CC2=CC=CC=C2C=C1)=O.OC(C=C1)=CC=C1C[CH](C%10=O)N%11.CSCC[CH](C(O)=O)N%10	17.8_7
C%11(COC1=CC2=CC=CC=C2C=C1)=O.OC(C=C1)=CC=C1C[CH](C%10=O)N%11.OC(C=C1)=CC=C1C[CH](C(O)=O)N%10	17.8_8
C%11(COC1=CC2=CC=CC=C2C=C1)=O.OC(C=C1)=CC=C1C[CH](C%10=O)N%11.O=C(O)[CH](CC1=C(F)C(F)=C(F)C(F)=C1F)N%10	17.8_9
C%10(COC1=CC2=CC=CC=C2C=C1)=O.O=C(O)CN%10	17.9_1
C%10(COC1=CC2=CC=CC=C2C=C1)=O.CC(C)[CH](C(O)=O)N%10	17.9_2
C%10(COC1=CC2=CC=CC=C2C=C1)=O.CC(C)C[CH](C(O)=O)N%10	17.9_3
C%10(COC1=CC2=CC=CC=C2C=C1)=O.C[CH](C(O)=O)N%10	17.9_4
C%10(COC1=CC2=CC=CC=C2C=C1)=O.O=C(O)[CH](CC1=CC=CC=C1)N%10	17.9_5
C%10(COC1=CC2=CC=CC=C2C=C1)=O.CC[CH](C)[CH](C(O)=O)N%10	17.9_6
C%10(COC1=CC2=CC=CC=C2C=C1)=O.CSCC[CH](C(O)=O)N%10	17.9_7
C%10(COC1=CC2=CC=CC=C2C=C1)=O.OC(C=C1)=CC=C1C[CH](C(O)=O)N%10	17.9_8
C%10(COC1=CC2=CC=CC=C2C=C1)=O.O=C(O)[CH](CC1=C(F)C(F)=C(F)C(F)=C1F)N%10	17.9_9
C%11(COC1=CC=CC2=CC=CC=C21)=O.O=C%10CN%11.O=C(O)CN%10	18.1_1
C%11(COC1=CC=CC2=CC=CC=C21)=O.O=C%10CN%11.CC(C)[CH](C(O)=O)N%10	18.1_2
C%11(COC1=CC=CC2=CC=CC=C21)=O.O=C%10CN%11.CC(C)C[CH](C(O)=O)N%10	18.1_3
C%11(COC1=CC=CC2=CC=CC=C21)=O.O=C%10CN%11.C[CH](C(O)=O)N%10	18.1_4

C%11(COC1=CC=CC2=CC=CC=C21)=O.O=C%10CN%11.O=C(O)[CH](CC1=CC=CC=C1)N%10	18.1_5
C%11(COC1=CC=CC2=CC=CC=C21)=O.O=C%10CN%11.CC[CH](C)[CH](C(O)=O)N%10	18.1_6
C%11(COC1=CC=CC2=CC=CC=C21)=O.O=C%10CN%11.CSCC[CH](C(O)=O)N%10	18.1_7
C%11(COC1=CC=CC2=CC=CC=C21)=O.O=C%10CN%11.OC(C=C1)=CC=C1C[CH](C(O)=O)N%10	18.1_8
C%11(COC1=CC=CC2=CC=CC=C21)=O.O=C%10CN%11.O=C(O)[CH](CC1=C(F)C(F)=C(F)C(F)=C1F)N%10	18.1_9
C%11(COC1=CC=CC2=CC=CC=C21)=O.CC(C)[CH](C%10=O)N%11.O=C(O)CN%10	18.2_1
C%11(COC1=CC=CC2=CC=CC=C21)=O.CC(C)[CH](C%10=O)N%11.CC(C)[CH](C(O)=O)N%10	18.2_2
C%11(COC1=CC=CC2=CC=CC=C21)=O.CC(C)[CH](C%10=O)N%11.CC(C)C[CH](C(O)=O)N%10	18.2_3
C%11(COC1=CC=CC2=CC=CC=C21)=O.CC(C)[CH](C%10=O)N%11.C[CH](C(O)=O)N%10	18.2_4
C%11(COC1=CC=CC2=CC=CC=C21)=O.CC(C)[CH](C%10=O)N%11.O=C(O)[CH](CC1=CC=CC=C1)N%10	18.2_5
C%11(COC1=CC=CC2=CC=CC=C21)=O.CC(C)[CH](C%10=O)N%11.CC[CH](C)[CH](C(O)=O)N%10	18.2_6
C%11(COC1=CC=CC2=CC=CC=C21)=O.CC(C)[CH](C%10=O)N%11.CSCC[CH](C(O)=O)N%10	18.2_7
C%11(COC1=CC=CC2=CC=CC=C21)=O.CC(C)[CH](C%10=O)N%11.OC(C=C1)=CC=C1C[CH](C(O)=O)N%10	18.2_8
C%11(COC1=CC=CC2=CC=CC=C21)=O.CC(C)[CH](C%10=O)N%11.O=C(O)[CH](CC1=C(F)C(F)=C(F)C(F)=C1F)N%10	18.2_9
C%11(COC1=CC=CC2=CC=CC=C21)=O.CC(C)C[CH](C%10=O)N%11.O=C(O)CN%10	18.3_1
C%11(COC1=CC=CC2=CC=CC=C21)=O.CC(C)C[CH](C%10=O)N%11.CC(C)[CH](C(O)=O)N%10	18.3_2
C%11(COC1=CC=CC2=CC=CC=C21)=O.CC(C)C[CH](C%10=O)N%11.CC(C)C[CH](C(O)=O)N%10	18.3_3
C%11(COC1=CC=CC2=CC=CC=C21)=O.CC(C)C[CH](C%10=O)N%11.C[CH](C(O)=O)N%10	18.3_4
C%11(COC1=CC=CC2=CC=CC=C21)=O.CC(C)C[CH](C%10=O)N%11.O=C(O)[CH](CC1=CC=CC=C1)N%10	18.3_5
C%11(COC1=CC=CC2=CC=CC=C21)=O.CC(C)C[CH](C%10=O)N%11.CC[CH](C)[CH](C(O)=O)N%10	18.3_6
C%11(COC1=CC=CC2=CC=CC=C21)=O.CC(C)C[CH](C%10=O)N%11.CSCC[CH](C(O)=O)N%10	18.3_7
C%11(COC1=CC=CC2=CC=CC=C21)=O.CC(C)C[CH](C%10=O)N%11.OC(C=C1)=CC=C1C[CH](C(O)=O)N%10	18.3_8
C%11(COC1=CC=CC2=CC=CC=C21)=O.CC(C)C[CH](C%10=O)N%11.O=C(O)[CH](CC1=C(F)C(F)=C(F)C(F)=C1F)N%10	18.3_9
C%11(COC1=CC=CC2=CC=CC=C21)=O.C[CH](C%10=O)N%11.O=C(O)CN%10	18.4_1
C%11(COC1=CC=CC2=CC=CC=C21)=O.C[CH](C%10=O)N%11.CC(C)[CH](C(O)=O)N%10	18.4_2
C%11(COC1=CC=CC2=CC=CC=C21)=O.C[CH](C%10=O)N%11.CC(C)C[CH](C(O)=O)N%10	18.4_3
C%11(COC1=CC=CC2=CC=CC=C21)=O.C[CH](C%10=O)N%11.C[CH](C(O)=O)N%10	18.4_4
C%11(COC1=CC=CC2=CC=CC=C21)=O.C[CH](C%10=O)N%11.O=C(O)[CH](CC1=CC=CC=C1)N%10	18.4_5
C%11(COC1=CC=CC2=CC=CC=C21)=O.C[CH](C%10=O)N%11.CC[CH](C)[CH](C(O)=O)N%10	18.4_6
C%11(COC1=CC=CC2=CC=CC=C21)=O.C[CH](C%10=O)N%11.CSCC[CH](C(O)=O)N%10	18.4_7
C%11(COC1=CC=CC2=CC=CC=C21)=O.C[CH](C%10=O)N%11.OC(C=C1)=CC=C1C[CH](C(O)=O)N%10	18.4_8

C%11(COC1=CC=CC2=CC=CC=C21)=O.C[CH](C%10=O)N%11.O=C(O)[CH](CC1=C(F)C(F)=C(F)C(F)=C1F)N%10	18.4_9
C%11(COC1=CC=CC2=CC=CC=C21)=O.O=C%10[CH](CC1=CC=CC=C1)N%11.O=C(O)CN%10	18.5_1
C%11(COC1=CC=CC2=CC=CC=C21)=O.O=C%10[CH](CC1=CC=CC=C1)N%11.CC(C)[CH](C(O)=O)N%10	18.5_2
C%11(COC1=CC=CC2=CC=CC=C21)=O.O=C%10[CH](CC1=CC=CC=C1)N%11.CC(C)C[CH](C(O)=O)N%10	18.5_3
C%11(COC1=CC=CC2=CC=CC=C21)=O.O=C%10[CH](CC1=CC=CC=C1)N%11.C[CH](C(O)=O)N%10	18.5_4
C%11(COC1=CC=CC2=CC=CC=C21)=O.O=C%10[CH](CC1=CC=CC=C1)N%11.O=C(O)[CH](CC1=CC=CC=C1)N%10	18.5_5
C%11(COC1=CC=CC2=CC=CC=C21)=O.O=C%10[CH](CC1=CC=CC=C1)N%11.CC[CH](C)[CH](C(O)=O)N%10	18.5_6
C%11(COC1=CC=CC2=CC=CC=C21)=O.O=C%10[CH](CC1=CC=CC=C1)N%11.CSCC[CH](C(O)=O)N%10	18.5_7
C%11(COC1=CC=CC2=CC=CC=C21)=O.O=C%10[CH](CC1=CC=CC=C1)N%11.OC(C=C1)=CC=C1C[CH](C(O)=O)N%10	18.5_8
C%11(COC1=CC=CC2=CC=CC=C21)=O.O=C%10[CH](CC1=CC=CC=C1)N%11.O=C(O)[CH](CC1=C(F)C(F)=C(F)C(F)=C1F)N%10	18.5_9
C%11(COC1=CC=CC2=CC=CC=C21)=O.CC[CH](C)[CH](C%10=O)N%11.O=C(O)CN%10	18.6_1
C%11(COC1=CC=CC2=CC=CC=C21)=O.CC[CH](C)[CH](C%10=O)N%11.CC(C)[CH](C(O)=O)N%10	18.6_2
C%11(COC1=CC=CC2=CC=CC=C21)=O.CC[CH](C)[CH](C%10=O)N%11.CC(C)C[CH](C(O)=O)N%10	18.6_3
C%11(COC1=CC=CC2=CC=CC=C21)=O.CC[CH](C)[CH](C%10=O)N%11.C[CH](C(O)=O)N%10	18.6_4
C%11(COC1=CC=CC2=CC=CC=C21)=O.CC[CH](C)[CH](C%10=O)N%11.O=C(O)[CH](CC1=CC=CC=C1)N%10	18.6_5
C%11(COC1=CC=CC2=CC=CC=C21)=O.CC[CH](C)[CH](C%10=O)N%11.CC[CH](C)[CH](C(O)=O)N%10	18.6_6
C%11(COC1=CC=CC2=CC=CC=C21)=O.CC[CH](C)[CH](C%10=O)N%11.CSCC[CH](C(O)=O)N%10	18.6_7
C%11(COC1=CC=CC2=CC=CC=C21)=O.CC[CH](C)[CH](C%10=O)N%11.OC(C=C1)=CC=C1C[CH](C(O)=O)N%10	18.6_8
C%11(COC1=CC=CC2=CC=CC=C21)=O.CC[CH](C)[CH](C%10=O)N%11.O=C(O)[CH](CC1=C(F)C(F)=C(F)C(F)=C1F)N%10	18.6_9
C%11(COC1=CC=CC2=CC=CC=C21)=O.CSCC[CH](C%10=O)N%11.O=C(O)CN%10	18.7_1
C%11(COC1=CC=CC2=CC=CC=C21)=O.CSCC[CH](C%10=O)N%11.CC(C)[CH](C(O)=O)N%10	18.7_2
C%11(COC1=CC=CC2=CC=CC=C21)=O.CSCC[CH](C%10=O)N%11.CC(C)C[CH](C(O)=O)N%10	18.7_3
C%11(COC1=CC=CC2=CC=CC=C21)=O.CSCC[CH](C%10=O)N%11.C[CH](C(O)=O)N%10	18.7_4
C%11(COC1=CC=CC2=CC=CC=C21)=O.CSCC[CH](C%10=O)N%11.O=C(O)[CH](CC1=CC=CC=C1)N%10	18.7_5
C%11(COC1=CC=CC2=CC=CC=C21)=O.CSCC[CH](C%10=O)N%11.CC[CH](C)[CH](C(O)=O)N%10	18.7_6
C%11(COC1=CC=CC2=CC=CC=C21)=O.CSCC[CH](C%10=O)N%11.CSCC[CH](C(O)=O)N%10	18.7_7
C%11(COC1=CC=CC2=CC=CC=C21)=O.CSCC[CH](C%10=O)N%11.OC(C=C1)=CC=C1C[CH](C(O)=O)N%10	18.7_8
C%11(COC1=CC=CC2=CC=CC=C21)=O.CSCC[CH](C%10=O)N%11.O=C(O)[CH](CC1=C(F)C(F)	18.7_9

=C(F)C(F)=C1F)N%10	
C%11(COC1=CC=CC2=CC=CC=C21)=O.OC(C=C1)=CC=C1C[CH](C%10=O)N%11.O=C(O)CN%10	18.8_1
C%11(COC1=CC=CC2=CC=CC=C21)=O.OC(C=C1)=CC=C1C[CH](C%10=O)N%11.CC(C)[CH](C(O)=O)N%10	18.8_2
C%11(COC1=CC=CC2=CC=CC=C21)=O.OC(C=C1)=CC=C1C[CH](C%10=O)N%11.CC(C)C[CH](C(O)=O)N%10	18.8_3
C%11(COC1=CC=CC2=CC=CC=C21)=O.OC(C=C1)=CC=C1C[CH](C%10=O)N%11.C[CH](C(O)=O)N%10	18.8_4
C%11(COC1=CC=CC2=CC=CC=C21)=O.OC(C=C1)=CC=C1C[CH](C%10=O)N%11.O=C(O)[CH](CC1=CC=CC=C1)N%10	18.8_5
C%11(COC1=CC=CC2=CC=CC=C21)=O.OC(C=C1)=CC=C1C[CH](C%10=O)N%11.CC[CH](C)[CH](C(O)=O)N%10	18.8_6
C%11(COC1=CC=CC2=CC=CC=C21)=O.OC(C=C1)=CC=C1C[CH](C%10=O)N%11.CSCC[CH](C(O)=O)N%10	18.8_7
C%11(COC1=CC=CC2=CC=CC=C21)=O.OC(C=C1)=CC=C1C[CH](C%10=O)N%11.OC(C=C1)=CC=C1C[CH](C(O)=O)N%10	18.8_8
C%11(COC1=CC=CC2=CC=CC=C21)=O.OC(C=C1)=CC=C1C[CH](C%10=O)N%11.O=C(O)[CH](CC1=C(F)C(F)=C(F)C(F)=C1F)N%10	18.8_9
C%10(COC1=CC=CC2=CC=CC=C21)=O.O=C(O)CN%10	18.9_1
C%10(COC1=CC=CC2=CC=CC=C21)=O.CC(C)[CH](C(O)=O)N%10	18.9_2
C%10(COC1=CC=CC2=CC=CC=C21)=O.CC(C)C[CH](C(O)=O)N%10	18.9_3
C%10(COC1=CC=CC2=CC=CC=C21)=O.C[CH](C(O)=O)N%10	18.9_4
C%10(COC1=CC=CC2=CC=CC=C21)=O.O=C(O)[CH](CC1=CC=CC=C1)N%10	18.9_5
C%10(COC1=CC=CC2=CC=CC=C21)=O.CC[CH](C)[CH](C(O)=O)N%10	18.9_6
C%10(COC1=CC=CC2=CC=CC=C21)=O.CSCC[CH](C(O)=O)N%10	18.9_7
C%10(COC1=CC=CC2=CC=CC=C21)=O.OC(C=C1)=CC=C1C[CH](C(O)=O)N%10	18.9_8
C%10(COC1=CC=CC2=CC=CC=C21)=O.O=C(O)[CH](CC1=C(F)C(F)=C(F)C(F)=C1F)N%10	18.9_9
C%11(CCCCCCCCCCCCCCC)=O.O=C%10CN%11.O=C(O)CN%10	19.1_1
C%11(CCCCCCCCCCCCCCC)=O.O=C%10CN%11.CC(C)[CH](C(O)=O)N%10	19.1_2
C%11(CCCCCCCCCCCCCCC)=O.O=C%10CN%11.CC(C)C[CH](C(O)=O)N%10	19.1_3
C%11(CCCCCCCCCCCCCCC)=O.O=C%10CN%11.C[CH](C(O)=O)N%10	19.1_4
C%11(CCCCCCCCCCCCCCC)=O.O=C%10CN%11.O=C(O)[CH](CC1=CC=CC=C1)N%10	19.1_5
C%11(CCCCCCCCCCCCCCC)=O.O=C%10CN%11.CC[CH](C)[CH](C(O)=O)N%10	19.1_6
C%11(CCCCCCCCCCCCCCC)=O.O=C%10CN%11.CSCC[CH](C(O)=O)N%10	19.1_7
C%11(CCCCCCCCCCCCCCC)=O.O=C%10CN%11.OC(C=C1)=CC=C1C[CH](C(O)=O)N%10	19.1_8
C%11(CCCCCCCCCCCCCCC)=O.O=C%10CN%11.O=C(O)[CH](CC1=C(F)C(F)=C(F)C(F)=C1F)N%10	19.1_9
C%11(CCCCCCCCCCCCCCC)=O.CC(C)[CH](C%10=O)N%11.O=C(O)CN%10	19.2_1
C%11(CCCCCCCCCCCCCCC)=O.CC(C)[CH](C%10=O)N%11.CC(C)[CH](C(O)=O)N%10	19.2_2
C%11(CCCCCCCCCCCCCCC)=O.CC(C)[CH](C%10=O)N%11.CC(C)C[CH](C(O)=O)N%10	19.2_3
C%11(CCCCCCCCCCCCCCC)=O.CC(C)[CH](C%10=O)N%11.C[CH](C(O)=O)N%10	19.2_4
C%11(CCCCCCCCCCCCCCC)=O.CC(C)[CH](C%10=O)N%11.O=C(O)[CH](CC1=CC=CC=C1)N%10	19.2_5
C%11(CCCCCCCCCCCCCCC)=O.CC(C)[CH](C%10=O)N%11.CC[CH](C)[CH](C(O)=O)N%10	19.2_6
C%11(CCCCCCCCCCCCCCC)=O.CC(C)[CH](C%10=O)N%11.CSCC[CH](C(O)=O)N%10	19.2_7

C%11(CCCCCCCCCCCCCCC)=O.CC(C)[CH](C%10=O)N%11.OC(C=C1)=CC=C1C[CH](C(O)=O)N%10	19.2_8
C%11(CCCCCCCCCCCCCCC)=O.CC(C)[CH](C%10=O)N%11.O=C(O)[CH](CC1=C(F)C(F)=C(F)C(F)=C1F)N%10	19.2_9
C%11(CCCCCCCCCCCCCCC)=O.CC(C)C[CH](C%10=O)N%11.O=C(O)CN%10	19.3_1
C%11(CCCCCCCCCCCCCCC)=O.CC(C)C[CH](C%10=O)N%11.CC(C)[CH](C(O)=O)N%10	19.3_2
C%11(CCCCCCCCCCCCCCC)=O.CC(C)C[CH](C%10=O)N%11.CC(C)C[CH](C(O)=O)N%10	19.3_3
C%11(CCCCCCCCCCCCCCC)=O.CC(C)C[CH](C%10=O)N%11.C[CH](C(O)=O)N%10	19.3_4
C%11(CCCCCCCCCCCCCCC)=O.CC(C)C[CH](C%10=O)N%11.O=C(O)[CH](CC1=CC=CC=C1)N%10	19.3_5
C%11(CCCCCCCCCCCCCCC)=O.CC(C)C[CH](C%10=O)N%11.CC[CH](C)[CH](C(O)=O)N%10	19.3_6
C%11(CCCCCCCCCCCCCCC)=O.CC(C)C[CH](C%10=O)N%11.CSCC[CH](C(O)=O)N%10	19.3_7
C%11(CCCCCCCCCCCCCCC)=O.CC(C)C[CH](C%10=O)N%11.OC(C=C1)=CC=C1C[CH](C(O)=O)N%10	19.3_8
C%11(CCCCCCCCCCCCCCC)=O.CC(C)C[CH](C%10=O)N%11.O=C(O)[CH](CC1=C(F)C(F)=C(F)C(F)=C1F)N%10	19.3_9
C%11(CCCCCCCCCCCCCCC)=O.C[CH](C%10=O)N%11.O=C(O)CN%10	19.4_1
C%11(CCCCCCCCCCCCCCC)=O.C[CH](C%10=O)N%11.CC(C)[CH](C(O)=O)N%10	19.4_2
C%11(CCCCCCCCCCCCCCC)=O.C[CH](C%10=O)N%11.CC(C)C[CH](C(O)=O)N%10	19.4_3
C%11(CCCCCCCCCCCCCCC)=O.C[CH](C%10=O)N%11.C[CH](C(O)=O)N%10	19.4_4
C%11(CCCCCCCCCCCCCCC)=O.C[CH](C%10=O)N%11.O=C(O)[CH](CC1=CC=CC=C1)N%10	19.4_5
C%11(CCCCCCCCCCCCCCC)=O.C[CH](C%10=O)N%11.CC[CH](C)[CH](C(O)=O)N%10	19.4_6
C%11(CCCCCCCCCCCCCCC)=O.C[CH](C%10=O)N%11.CSCC[CH](C(O)=O)N%10	19.4_7
C%11(CCCCCCCCCCCCCCC)=O.C[CH](C%10=O)N%11.OC(C=C1)=CC=C1C[CH](C(O)=O)N%10	19.4_8
C%11(CCCCCCCCCCCCCCC)=O.C[CH](C%10=O)N%11.O=C(O)[CH](CC1=C(F)C(F)=C(F)C(F)=C1F)N%10	19.4_9
C%11(CCCCCCCCCCCCCCC)=O.O=C%10[CH](CC1=CC=CC=C1)N%11.O=C(O)CN%10	19.5_1
C%11(CCCCCCCCCCCCCCC)=O.O=C%10[CH](CC1=CC=CC=C1)N%11.CC(C)[CH](C(O)=O)N%10	19.5_2
C%11(CCCCCCCCCCCCCCC)=O.O=C%10[CH](CC1=CC=CC=C1)N%11.CC(C)C[CH](C(O)=O)N%10	19.5_3
C%11(CCCCCCCCCCCCCCC)=O.O=C%10[CH](CC1=CC=CC=C1)N%11.C[CH](C(O)=O)N%10	19.5_4
C%11(CCCCCCCCCCCCCCC)=O.O=C%10[CH](CC1=CC=CC=C1)N%11.O=C(O)[CH](CC1=CC=CC=C1)N%10	19.5_5
C%11(CCCCCCCCCCCCCCC)=O.O=C%10[CH](CC1=CC=CC=C1)N%11.CC[CH](C)[CH](C(O)=O)N%10	19.5_6
C%11(CCCCCCCCCCCCCCC)=O.O=C%10[CH](CC1=CC=CC=C1)N%11.CSCC[CH](C(O)=O)N%10	19.5_7
C%11(CCCCCCCCCCCCCCC)=O.O=C%10[CH](CC1=CC=CC=C1)N%11.OC(C=C1)=CC=C1C[CH](C(O)=O)N%10	19.5_8
C%11(CCCCCCCCCCCCCCC)=O.O=C%10[CH](CC1=CC=CC=C1)N%11.O=C(O)[CH](CC1=C(F)C(F)=C(F)C(F)=C1F)N%10	19.5_9
C%11(CCCCCCCCCCCCCCC)=O.CC[CH](C)[CH](C%10=O)N%11.O=C(O)CN%10	19.6_1
C%11(CCCCCCCCCCCCCCC)=O.CC[CH](C)[CH](C%10=O)N%11.CC(C)[CH](C(O)=O)N%10	19.6_2
C%11(CCCCCCCCCCCCCCC)=O.CC[CH](C)[CH](C%10=O)N%11.CC(C)C[CH](C(O)=O)N%10	19.6_3
C%11(CCCCCCCCCCCCCCC)=O.CC[CH](C)[CH](C%10=O)N%11.C[CH](C(O)=O)N%10	19.6_4

C%11(CCCCCCCCCCCCCCC)=O.CC[CH](C)[CH](C%10=O)N%11.O=C(O)[CH](CC1=CC=CC=C1)N%10	19.6_5
C%11(CCCCCCCCCCCCCCC)=O.CC[CH](C)[CH](C%10=O)N%11.CC[CH](C)[CH](C(O)=O)N%10	19.6_6
C%11(CCCCCCCCCCCCCCC)=O.CC[CH](C)[CH](C%10=O)N%11.CSCC[CH](C(O)=O)N%10	19.6_7
C%11(CCCCCCCCCCCCCCC)=O.CC[CH](C)[CH](C%10=O)N%11.OC(C=C1)=CC=C1C[CH](C(O)=O)N%10	19.6_8
C%11(CCCCCCCCCCCCCCC)=O.CC[CH](C)[CH](C%10=O)N%11.O=C(O)[CH](CC1=C(F)C(F)=C(F)C(F)=C1F)N%10	19.6_9
C%11(CCCCCCCCCCCCCCC)=O.CSCC[CH](C%10=O)N%11.O=C(O)CN%10	19.7_1
C%11(CCCCCCCCCCCCCCC)=O.CSCC[CH](C%10=O)N%11.CC(C)[CH](C(O)=O)N%10	19.7_2
C%11(CCCCCCCCCCCCCCC)=O.CSCC[CH](C%10=O)N%11.CC(C)C[CH](C(O)=O)N%10	19.7_3
C%11(CCCCCCCCCCCCCCC)=O.CSCC[CH](C%10=O)N%11.C[CH](C(O)=O)N%10	19.7_4
C%11(CCCCCCCCCCCCCCC)=O.CSCC[CH](C%10=O)N%11.O=C(O)[CH](CC1=CC=CC=C1)N%10	19.7_5
C%11(CCCCCCCCCCCCCCC)=O.CSCC[CH](C%10=O)N%11.CC[CH](C)[CH](C(O)=O)N%10	19.7_6
C%11(CCCCCCCCCCCCCCC)=O.CSCC[CH](C%10=O)N%11.CSCC[CH](C(O)=O)N%10	19.7_7
C%11(CCCCCCCCCCCCCCC)=O.CSCC[CH](C%10=O)N%11.OC(C=C1)=CC=C1C[CH](C(O)=O)N%10	19.7_8
C%11(CCCCCCCCCCCCCCC)=O.CSCC[CH](C%10=O)N%11.O=C(O)[CH](CC1=C(F)C(F)=C(F)C(F)=C1F)N%10	19.7_9
C%11(CCCCCCCCCCCCCCC)=O.OC(C=C1)=CC=C1C[CH](C%10=O)N%11.O=C(O)CN%10	19.8_1
C%11(CCCCCCCCCCCCCCC)=O.OC(C=C1)=CC=C1C[CH](C%10=O)N%11.CC(C)[CH](C(O)=O)N%10	19.8_2
C%11(CCCCCCCCCCCCCCC)=O.OC(C=C1)=CC=C1C[CH](C%10=O)N%11.CC(C)C[CH](C(O)=O)N%10	19.8_3
C%11(CCCCCCCCCCCCCCC)=O.OC(C=C1)=CC=C1C[CH](C%10=O)N%11.C[CH](C(O)=O)N%10	19.8_4
C%11(CCCCCCCCCCCCCCC)=O.OC(C=C1)=CC=C1C[CH](C%10=O)N%11.O=C(O)[CH](CC1=CC=CC=C1)N%10	19.8_5
C%11(CCCCCCCCCCCCCCC)=O.OC(C=C1)=CC=C1C[CH](C%10=O)N%11.CC[CH](C)[CH](C(O)=O)N%10	19.8_6
C%11(CCCCCCCCCCCCCCC)=O.OC(C=C1)=CC=C1C[CH](C%10=O)N%11.CSCC[CH](C(O)=O)N%10	19.8_7
C%11(CCCCCCCCCCCCCCC)=O.OC(C=C1)=CC=C1C[CH](C%10=O)N%11.OC(C=C1)=CC=C1C[CH](C(O)=O)N%10	19.8_8
C%11(CCCCCCCCCCCCCCC)=O.OC(C=C1)=CC=C1C[CH](C%10=O)N%11.O=C(O)[CH](CC1=C(F)C(F)=C(F)C(F)=C1F)N%10	19.8_9
C%10(CCCCCCCCCCCCCCC)=O.O=C(O)CN%10	19.9_1
C%10(CCCCCCCCCCCCCCC)=O.CC(C)[CH](C(O)=O)N%10	19.9_2
C%10(CCCCCCCCCCCCCCC)=O.CC(C)C[CH](C(O)=O)N%10	19.9_3
C%10(CCCCCCCCCCCCCCC)=O.C[CH](C(O)=O)N%10	19.9_4
C%10(CCCCCCCCCCCCCCC)=O.O=C(O)[CH](CC1=CC=CC=C1)N%10	19.9_5
C%10(CCCCCCCCCCCCCCC)=O.CC[CH](C)[CH](C(O)=O)N%10	19.9_6
C%10(CCCCCCCCCCCCCCC)=O.CSCC[CH](C(O)=O)N%10	19.9_7
C%10(CCCCCCCCCCCCCCC)=O.OC(C=C1)=CC=C1C[CH](C(O)=O)N%10	19.9_8
C%10(CCCCCCCCCCCCCCC)=O.O=C(O)[CH](CC1=C(F)C(F)=C(F)C(F)=C1F)N%10	19.9_9

CCCCCCCCCCCCCC%11=O.O=C%10CN%11.O=C(O)CN%10	20.1_1
CCCCCCCCCCCCCC%11=O.O=C%10CN%11.CC(C)[CH](C(O)=O)N%10	20.1_2
CCCCCCCCCCCCCC%11=O.O=C%10CN%11.CC(C)C[CH](C(O)=O)N%10	20.1_3
CCCCCCCCCCCCCC%11=O.O=C%10CN%11.C[CH](C(O)=O)N%10	20.1_4
CCCCCCCCCCCCCC%11=O.O=C%10CN%11.O=C(O)[CH](CC1=CC=CC=C1)N%10	20.1_5
CCCCCCCCCCCCCC%11=O.O=C%10CN%11.CC[CH](C)[CH](C(O)=O)N%10	20.1_6
CCCCCCCCCCCCCC%11=O.O=C%10CN%11.CSCC[CH](C(O)=O)N%10	20.1_7
CCCCCCCCCCCCCC%11=O.O=C%10CN%11.OC(C=C1)=CC=C1C[CH](C(O)=O)N%10	20.1_8
CCCCCCCCCCCCCC%11=O.O=C%10CN%11.O=C(O)[CH](CC1=C(F)C(F)=C(F)C(F)=C1F)N%10	20.1_9
CCCCCCCCCCCCCC%11=O.CC(C)[CH](C%10=O)N%11.O=C(O)CN%10	20.2_1
CCCCCCCCCCCCCC%11=O.CC(C)[CH](C%10=O)N%11.CC(C)[CH](C(O)=O)N%10	20.2_2
CCCCCCCCCCCCCC%11=O.CC(C)[CH](C%10=O)N%11.CC(C)C[CH](C(O)=O)N%10	20.2_3
CCCCCCCCCCCCCC%11=O.CC(C)[CH](C%10=O)N%11.C[CH](C(O)=O)N%10	20.2_4
CCCCCCCCCCCCCC%11=O.CC(C)[CH](C%10=O)N%11.O=C(O)[CH](CC1=CC=CC=C1)N%10	20.2_5
CCCCCCCCCCCCCC%11=O.CC(C)[CH](C%10=O)N%11.CC[CH](C)[CH](C(O)=O)N%10	20.2_6
CCCCCCCCCCCCCC%11=O.CC(C)[CH](C%10=O)N%11.CSCC[CH](C(O)=O)N%10	20.2_7
CCCCCCCCCCCCCC%11=O.CC(C)[CH](C%10=O)N%11.OC(C=C1)=CC=C1C[CH](C(O)=O)N%1<br>0	20.2_8
CCCCCCCCCCCCCC%11=O.CC(C)[CH](C%10=O)N%11.O=C(O)[CH](CC1=C(F)C(F)=C(F)C(F)=<br>C1F)N%10	20.2_9
CCCCCCCCCCCCCC%11=O.CC(C)C[CH](C%10=O)N%11.O=C(O)CN%10	20.3_1
CCCCCCCCCCCCCC%11=O.CC(C)C[CH](C%10=O)N%11.CC(C)[CH](C(O)=O)N%10	20.3_2
CCCCCCCCCCCCCC%11=O.CC(C)C[CH](C%10=O)N%11.CC(C)C[CH](C(O)=O)N%10	20.3_3
CCCCCCCCCCCCCC%11=O.CC(C)C[CH](C%10=O)N%11.C[CH](C(O)=O)N%10	20.3_4
CCCCCCCCCCCCCC%11=O.CC(C)C[CH](C%10=O)N%11.O=C(O)[CH](CC1=CC=CC=C1)N%10	20.3_5
CCCCCCCCCCCCCC%11=O.CC(C)C[CH](C%10=O)N%11.CC[CH](C)[CH](C(O)=O)N%10	20.3_6
CCCCCCCCCCCCCC%11=O.CC(C)C[CH](C%10=O)N%11.CSCC[CH](C(O)=O)N%10	20.3_7
CCCCCCCCCCCCCC%11=O.CC(C)C[CH](C%10=O)N%11.OC(C=C1)=CC=C1C[CH](C(O)=O)N%<br>10	20.3_8
CCCCCCCCCCCCCC%11=O.CC(C)C[CH](C%10=O)N%11.O=C(O)[CH](CC1=C(F)C(F)=C(F)C(F)<br>=C1F)N%10	20.3_9
CCCCCCCCCCCCCC%11=O.C[CH](C%10=O)N%11.O=C(O)CN%10	20.4_1
CCCCCCCCCCCCCC%11=O.C[CH](C%10=O)N%11.CC(C)[CH](C(O)=O)N%10	20.4_2
CCCCCCCCCCCCCC%11=O.C[CH](C%10=O)N%11.CC(C)C[CH](C(O)=O)N%10	20.4_3
CCCCCCCCCCCCCC%11=O.C[CH](C%10=O)N%11.C[CH](C(O)=O)N%10	20.4_4
CCCCCCCCCCCCCC%11=O.C[CH](C%10=O)N%11.O=C(O)[CH](CC1=CC=CC=C1)N%10	20.4_5
CCCCCCCCCCCCCC%11=O.C[CH](C%10=O)N%11.CC[CH](C)[CH](C(O)=O)N%10	20.4_6
CCCCCCCCCCCCCC%11=O.C[CH](C%10=O)N%11.CSCC[CH](C(O)=O)N%10	20.4_7
CCCCCCCCCCCCCC%11=O.C[CH](C%10=O)N%11.OC(C=C1)=CC=C1C[CH](C(O)=O)N%10	20.4_8
CCCCCCCCCCCCCC%11=O.C[CH](C%10=O)N%11.O=C(O)[CH](CC1=C(F)C(F)=C(F)C(F)=C1F)<br>N%10	20.4_9
CCCCCCCCCCCCCC%11=O.O=C%10[CH](CC1=CC=CC=C1)N%11.O=C(O)CN%10	20.5_1
CCCCCCCCCCCCCC%11=O.O=C%10[CH](CC1=CC=CC=C1)N%11.CC(C)[CH](C(O)=O)N%10	20.5_2
CCCCCCCCCCCCCC%11=O.O=C%10[CH](CC1=CC=CC=C1)N%11.CC(C)C[CH](C(O)=O)N%10	20.5_3
CCCCCCCCCCCCCC%11=O.O=C%10[CH](CC1=CC=CC=C1)N%11.C[CH](C(O)=O)N%10	20.5_4

CCCCCCCCCCCCCC%11=O.O=C%10[CH](CC1=CC=CC=C1)N%11.O=C(O)[CH](CC1=CC=CC=C1)N%10	20.5_5
CCCCCCCCCCCCCC%11=O.O=C%10[CH](CC1=CC=CC=C1)N%11.CC[CH](C)[CH](C(O)=O)N%10	20.5_6
CCCCCCCCCCCCCC%11=O.O=C%10[CH](CC1=CC=CC=C1)N%11.CSCC[CH](C(O)=O)N%10	20.5_7
CCCCCCCCCCCCCC%11=O.O=C%10[CH](CC1=CC=CC=C1)N%11.OC(C=C1)=CC=C1C[CH](C(O)=O)N%10	20.5_8
CCCCCCCCCCCCCC%11=O.O=C%10[CH](CC1=CC=CC=C1)N%11.O=C(O)[CH](CC1=C(F)C(F)=C(F)C(F)=C1F)N%10	20.5_9
CCCCCCCCCCCCCC%11=O.CC[CH](C)[CH](C%10=O)N%11.O=C(O)CN%10	20.6_1
CCCCCCCCCCCCCC%11=O.CC[CH](C)[CH](C%10=O)N%11.CC(C)[CH](C(O)=O)N%10	20.6_2
CCCCCCCCCCCCCC%11=O.CC[CH](C)[CH](C%10=O)N%11.CC(C)C[CH](C(O)=O)N%10	20.6_3
CCCCCCCCCCCCCC%11=O.CC[CH](C)[CH](C%10=O)N%11.C[CH](C(O)=O)N%10	20.6_4
CCCCCCCCCCCCCC%11=O.CC[CH](C)[CH](C%10=O)N%11.O=C(O)[CH](CC1=CC=CC=C1)N%10	20.6_5
CCCCCCCCCCCCCC%11=O.CC[CH](C)[CH](C%10=O)N%11.CC[CH](C)[CH](C(O)=O)N%10	20.6_6
CCCCCCCCCCCCCC%11=O.CC[CH](C)[CH](C%10=O)N%11.CSCC[CH](C(O)=O)N%10	20.6_7
CCCCCCCCCCCCCC%11=O.CC[CH](C)[CH](C%10=O)N%11.OC(C=C1)=CC=C1C[CH](C(O)=O)N%10	20.6_8
CCCCCCCCCCCCCC%11=O.CC[CH](C)[CH](C%10=O)N%11.O=C(O)[CH](CC1=C(F)C(F)=C(F)C(F)=C1F)N%10	20.6_9
CCCCCCCCCCCCCC%11=O.CSCC[CH](C%10=O)N%11.O=C(O)CN%10	20.7_1
CCCCCCCCCCCCCC%11=O.CSCC[CH](C%10=O)N%11.CC(C)[CH](C(O)=O)N%10	20.7_2
CCCCCCCCCCCCCC%11=O.CSCC[CH](C%10=O)N%11.CC(C)C[CH](C(O)=O)N%10	20.7_3
CCCCCCCCCCCCCC%11=O.CSCC[CH](C%10=O)N%11.C[CH](C(O)=O)N%10	20.7_4
CCCCCCCCCCCCCC%11=O.CSCC[CH](C%10=O)N%11.O=C(O)[CH](CC1=CC=CC=C1)N%10	20.7_5
CCCCCCCCCCCCCC%11=O.CSCC[CH](C%10=O)N%11.CC[CH](C)[CH](C(O)=O)N%10	20.7_6
CCCCCCCCCCCCCC%11=O.CSCC[CH](C%10=O)N%11.CSCC[CH](C(O)=O)N%10	20.7_7
CCCCCCCCCCCCCC%11=O.CSCC[CH](C%10=O)N%11.OC(C=C1)=CC=C1C[CH](C(O)=O)N%10	20.7_8
CCCCCCCCCCCCCC%11=O.CSCC[CH](C%10=O)N%11.O=C(O)[CH](CC1=C(F)C(F)=C(F)C(F)=C1F)N%10	20.7_9
CCCCCCCCCCCCCC%11=O.OC(C=C1)=CC=C1C[CH](C%10=O)N%11.O=C(O)CN%10	20.8_1
CCCCCCCCCCCCCC%11=O.OC(C=C1)=CC=C1C[CH](C%10=O)N%11.CC(C)[CH](C(O)=O)N%10	20.8_2
CCCCCCCCCCCCCC%11=O.OC(C=C1)=CC=C1C[CH](C%10=O)N%11.CC(C)C[CH](C(O)=O)N%10	20.8_3
CCCCCCCCCCCCCC%11=O.OC(C=C1)=CC=C1C[CH](C%10=O)N%11.C[CH](C(O)=O)N%10	20.8_4
CCCCCCCCCCCCCC%11=O.OC(C=C1)=CC=C1C[CH](C%10=O)N%11.O=C(O)[CH](CC1=CC=CC=C1)N%10	20.8_5
CCCCCCCCCCCCCC%11=O.OC(C=C1)=CC=C1C[CH](C%10=O)N%11.CC[CH](C)[CH](C(O)=O)N%10	20.8_6
CCCCCCCCCCCCCC%11=O.OC(C=C1)=CC=C1C[CH](C%10=O)N%11.CSCC[CH](C(O)=O)N%10	20.8_7
CCCCCCCCCCCCCC%11=O.OC(C=C1)=CC=C1C[CH](C%10=O)N%11.OC(C=C1)=CC=C1C[CH](C(O)=O)N%10	20.8_8
CCCCCCCCCCCCCC%11=O.OC(C=C1)=CC=C1C[CH](C%10=O)N%11.O=C(O)[CH](CC1=C(F)C(	20.8_9

F)=C(F)C(F)=C1F)N%10	
CCCCCCCCCCCCCC%10=O.O=C(O)CN%10	20.9_1
CCCCCCCCCCCCCC%10=O.CC(C)[CH](C(O)=O)N%10	20.9_2
CCCCCCCCCCCCCC%10=O.CC(C)C[CH](C(O)=O)N%10	20.9_3
CCCCCCCCCCCCCC%10=O.C[CH](C(O)=O)N%10	20.9_4
CCCCCCCCCCCCCC%10=O.O=C(O)[CH](CC1=CC=CC=C1)N%10	20.9_5
CCCCCCCCCCCCCC%10=O.CC[CH](C)[CH](C(O)=O)N%10	20.9_6
CCCCCCCCCCCCCC%10=O.CSCC[CH](C(O)=O)N%10	20.9_7
CCCCCCCCCCCCCC%10=O.OC(C=C1)=CC=C1C[CH](C(O)=O)N%10	20.9_8
CCCCCCCCCCCCCC%10=O.O=C(O)[CH](CC1=C(F)C(F)=C(F)C(F)=C1F)N%10	20.9_9
C%11(COC1=CC=CC=C1)=O.O=C%10CN%11.O=C(O)CN%10	21.1_1
C%11(COC1=CC=CC=C1)=O.O=C%10CN%11.CC(C)[CH](C(O)=O)N%10	21.1_2
C%11(COC1=CC=CC=C1)=O.O=C%10CN%11.CC(C)C[CH](C(O)=O)N%10	21.1_3
C%11(COC1=CC=CC=C1)=O.O=C%10CN%11.C[CH](C(O)=O)N%10	21.1_4
C%11(COC1=CC=CC=C1)=O.O=C%10CN%11.O=C(O)[CH](CC1=CC=CC=C1)N%10	21.1_5
C%11(COC1=CC=CC=C1)=O.O=C%10CN%11.CC[CH](C)[CH](C(O)=O)N%10	21.1_6
C%11(COC1=CC=CC=C1)=O.O=C%10CN%11.CSCC[CH](C(O)=O)N%10	21.1_7
C%11(COC1=CC=CC=C1)=O.O=C%10CN%11.OC(C=C1)=CC=C1C[CH](C(O)=O)N%10	21.1_8
C%11(COC1=CC=CC=C1)=O.O=C%10CN%11.O=C(O)[CH](CC1=C(F)C(F)=C(F)C(F)=C1F)N%1<br>0	21.1_9
C%11(COC1=CC=CC=C1)=O.CC(C)[CH](C%10=O)N%11.O=C(O)CN%10	21.2_1
C%11(COC1=CC=CC=C1)=O.CC(C)[CH](C%10=O)N%11.CC(C)[CH](C(O)=O)N%10	21.2_2
C%11(COC1=CC=CC=C1)=O.CC(C)[CH](C%10=O)N%11.CC(C)C[CH](C(O)=O)N%10	21.2_3
C%11(COC1=CC=CC=C1)=O.CC(C)[CH](C%10=O)N%11.C[CH](C(O)=O)N%10	21.2_4
C%11(COC1=CC=CC=C1)=O.CC(C)[CH](C%10=O)N%11.O=C(O)[CH](CC1=CC=CC=C1)N%10	21.2_5
C%11(COC1=CC=CC=C1)=O.CC(C)[CH](C%10=O)N%11.CC[CH](C)[CH](C(O)=O)N%10	21.2_6
C%11(COC1=CC=CC=C1)=O.CC(C)[CH](C%10=O)N%11.CSCC[CH](C(O)=O)N%10	21.2_7
C%11(COC1=CC=CC=C1)=O.CC(C)[CH](C%10=O)N%11.OC(C=C1)=CC=C1C[CH](C(O)=O)N%<br>10	21.2_8
C%11(COC1=CC=CC=C1)=O.CC(C)[CH](C%10=O)N%11.O=C(O)[CH](CC1=C(F)C(F)=C(F)C(F)<br>=C1F)N%10	21.2_9
C%11(COC1=CC=CC=C1)=O.CC(C)C[CH](C%10=O)N%11.O=C(O)CN%10	21.3_1
C%11(COC1=CC=CC=C1)=O.CC(C)C[CH](C%10=O)N%11.CC(C)[CH](C(O)=O)N%10	21.3_2
C%11(COC1=CC=CC=C1)=O.CC(C)C[CH](C%10=O)N%11.CC(C)C[CH](C(O)=O)N%10	21.3_3
C%11(COC1=CC=CC=C1)=O.CC(C)C[CH](C%10=O)N%11.C[CH](C(O)=O)N%10	21.3_4
C%11(COC1=CC=CC=C1)=O.CC(C)C[CH](C%10=O)N%11.O=C(O)[CH](CC1=CC=CC=C1)N%1<br>0	21.3_5
C%11(COC1=CC=CC=C1)=O.CC(C)C[CH](C%10=O)N%11.CC[CH](C)[CH](C(O)=O)N%10	21.3_6
C%11(COC1=CC=CC=C1)=O.CC(C)C[CH](C%10=O)N%11.CSCC[CH](C(O)=O)N%10	21.3_7
C%11(COC1=CC=CC=C1)=O.CC(C)C[CH](C%10=O)N%11.OC(C=C1)=CC=C1C[CH](C(O)=O)N<br>%10	21.3_8
C%11(COC1=CC=CC=C1)=O.CC(C)C[CH](C%10=O)N%11.O=C(O)[CH](CC1=C(F)C(F)=C(F)C(F<br>)=C1F)N%10	21.3_9
C%11(COC1=CC=CC=C1)=O.C[CH](C%10=O)N%11.O=C(O)CN%10	21.4_1
C%11(COC1=CC=CC=C1)=O.C[CH](C%10=O)N%11.CC(C)[CH](C(O)=O)N%10	21.4_2

C%11(COC1=CC=CC=C1)=O.C[CH](C%10=O)N%11.CC(C)C[CH](C(O)=O)N%10	21.4_3
C%11(COC1=CC=CC=C1)=O.C[CH](C%10=O)N%11.C[CH](C(O)=O)N%10	21.4_4
C%11(COC1=CC=CC=C1)=O.C[CH](C%10=O)N%11.O=C(O)[CH](CC1=CC=CC=C1)N%10	21.4_5
C%11(COC1=CC=CC=C1)=O.C[CH](C%10=O)N%11.CC[CH](C)[CH](C(O)=O)N%10	21.4_6
C%11(COC1=CC=CC=C1)=O.C[CH](C%10=O)N%11.CSCC[CH](C(O)=O)N%10	21.4_7
C%11(COC1=CC=CC=C1)=O.C[CH](C%10=O)N%11.OC(C=C1)=CC=C1C[CH](C(O)=O)N%10	21.4_8
C%11(COC1=CC=CC=C1)=O.C[CH](C%10=O)N%11.O=C(O)[CH](CC1=C(F)C(F)=C(F)C(F)=C1 F)N%10	21.4_9
C%11(COC1=CC=CC=C1)=O.O=C%10[CH](CC1=CC=CC=C1)N%11.O=C(O)CN%10	21.5_1
C%11(COC1=CC=CC=C1)=O.O=C%10[CH](CC1=CC=CC=C1)N%11.CC(C)[CH](C(O)=O)N%10	21.5_2
C%11(COC1=CC=CC=C1)=O.O=C%10[CH](CC1=CC=CC=C1)N%11.CC(C)C[CH](C(O)=O)N%1 0	21.5_3
C%11(COC1=CC=CC=C1)=O.O=C%10[CH](CC1=CC=CC=C1)N%11.C[CH](C(O)=O)N%10	21.5_4
C%11(COC1=CC=CC=C1)=O.O=C%10[CH](CC1=CC=CC=C1)N%11.O=C(O)[CH](CC1=CC=CC= C1)N%10	21.5_5
C%11(COC1=CC=CC=C1)=O.O=C%10[CH](CC1=CC=CC=C1)N%11.CC[CH](C)[CH](C(O)=O)N %10	21.5_6
C%11(COC1=CC=CC=C1)=O.O=C%10[CH](CC1=CC=CC=C1)N%11.CSCC[CH](C(O)=O)N%10	21.5_7
C%11(COC1=CC=CC=C1)=O.O=C%10[CH](CC1=CC=CC=C1)N%11.OC(C=C1)=CC=C1C[CH](C (O)=O)N%10	21.5_8
C%11(COC1=CC=CC=C1)=O.O=C%10[CH](CC1=CC=CC=C1)N%11.O=C(O)[CH](CC1=C(F)C(F)=C(F)C(F)=C1F)N%10	21.5_9
C%11(COC1=CC=CC=C1)=O.CC[CH](C)[CH](C%10=O)N%11.O=C(O)CN%10	21.6_1
C%11(COC1=CC=CC=C1)=O.CC[CH](C)[CH](C%10=O)N%11.CC(C)[CH](C(O)=O)N%10	21.6_2
C%11(COC1=CC=CC=C1)=O.CC[CH](C)[CH](C%10=O)N%11.CC(C)C[CH](C(O)=O)N%10	21.6_3
C%11(COC1=CC=CC=C1)=O.CC[CH](C)[CH](C%10=O)N%11.C[CH](C(O)=O)N%10	21.6_4
C%11(COC1=CC=CC=C1)=O.CC[CH](C)[CH](C%10=O)N%11.O=C(O)[CH](CC1=CC=CC=C1)N %10	21.6_5
C%11(COC1=CC=CC=C1)=O.CC[CH](C)[CH](C%10=O)N%11.CC[CH](C)[CH](C(O)=O)N%10	21.6_6
C%11(COC1=CC=CC=C1)=O.CC[CH](C)[CH](C%10=O)N%11.CSCC[CH](C(O)=O)N%10	21.6_7
C%11(COC1=CC=CC=C1)=O.CC[CH](C)[CH](C%10=O)N%11.OC(C=C1)=CC=C1C[CH](C(O)= O)N%10	21.6_8
C%11(COC1=CC=CC=C1)=O.CC[CH](C)[CH](C%10=O)N%11.O=C(O)[CH](CC1=C(F)C(F)=C(F) C(F)=C1F)N%10	21.6_9
C%11(COC1=CC=CC=C1)=O.CSCC[CH](C%10=O)N%11.O=C(O)CN%10	21.7_1
C%11(COC1=CC=CC=C1)=O.CSCC[CH](C%10=O)N%11.CC(C)[CH](C(O)=O)N%10	21.7_2
C%11(COC1=CC=CC=C1)=O.CSCC[CH](C%10=O)N%11.CC(C)C[CH](C(O)=O)N%10	21.7_3
C%11(COC1=CC=CC=C1)=O.CSCC[CH](C%10=O)N%11.C[CH](C(O)=O)N%10	21.7_4
C%11(COC1=CC=CC=C1)=O.CSCC[CH](C%10=O)N%11.O=C(O)[CH](CC1=CC=CC=C1)N%10	21.7_5
C%11(COC1=CC=CC=C1)=O.CSCC[CH](C%10=O)N%11.CC[CH](C)[CH](C(O)=O)N%10	21.7_6
C%11(COC1=CC=CC=C1)=O.CSCC[CH](C%10=O)N%11.CSCC[CH](C(O)=O)N%10	21.7_7
C%11(COC1=CC=CC=C1)=O.CSCC[CH](C%10=O)N%11.OC(C=C1)=CC=C1C[CH](C(O)=O)N% 10	21.7_8
C%11(COC1=CC=CC=C1)=O.CSCC[CH](C%10=O)N%11.O=C(O)[CH](CC1=C(F)C(F)=C(F)C(F) =C1F)N%10	21.7_9
C%11(COC1=CC=CC=C1)=O.OC(C=C1)=CC=C1C[CH](C%10=O)N%11.O=C(O)CN%10	21.8_1

C%11(COC1=CC=CC=C1)=O.OC(C=C1)=CC=C1C[CH](C%10=O)N%11.CC(C)[CH](C(O)=O)N%10	21.8_2
C%11(COC1=CC=CC=C1)=O.OC(C=C1)=CC=C1C[CH](C%10=O)N%11.CC(C)C[CH](C(O)=O)N%10	21.8_3
C%11(COC1=CC=CC=C1)=O.OC(C=C1)=CC=C1C[CH](C%10=O)N%11.C[CH](C(O)=O)N%10	21.8_4
C%11(COC1=CC=CC=C1)=O.OC(C=C1)=CC=C1C[CH](C%10=O)N%11.O=C(O)[CH](CC1=CC=CC=C1)N%10	21.8_5
C%11(COC1=CC=CC=C1)=O.OC(C=C1)=CC=C1C[CH](C%10=O)N%11.CC[CH](C)[CH](C(O)=O)N%10	21.8_6
C%11(COC1=CC=CC=C1)=O.OC(C=C1)=CC=C1C[CH](C%10=O)N%11.CSCC[CH](C(O)=O)N%10	21.8_7
C%11(COC1=CC=CC=C1)=O.OC(C=C1)=CC=C1C[CH](C%10=O)N%11.OC(C=C1)=CC=C1C[CH](C(O)=O)N%10	21.8_8
C%11(COC1=CC=CC=C1)=O.OC(C=C1)=CC=C1C[CH](C%10=O)N%11.O=C(O)[CH](CC1=C(F)C(F)=C(F)C(F)=C1F)N%10	21.8_9
C%10(COC1=CC=CC=C1)=O.O=C(O)CN%10	21.9_1
C%10(COC1=CC=CC=C1)=O.CC(C)[CH](C(O)=O)N%10	21.9_2
C%10(COC1=CC=CC=C1)=O.CC(C)C[CH](C(O)=O)N%10	21.9_3
C%10(COC1=CC=CC=C1)=O.C[CH](C(O)=O)N%10	21.9_4
C%10(COC1=CC=CC=C1)=O.O=C(O)[CH](CC1=CC=CC=C1)N%10	21.9_5
C%10(COC1=CC=CC=C1)=O.CC[CH](C)[CH](C(O)=O)N%10	21.9_6
C%10(COC1=CC=CC=C1)=O.CSCC[CH](C(O)=O)N%10	21.9_7
C%10(COC1=CC=CC=C1)=O.OC(C=C1)=CC=C1C[CH](C(O)=O)N%10	21.9_8
C%10(COC1=CC=CC=C1)=O.O=C(O)[CH](CC1=C(F)C(F)=C(F)C(F)=C1F)N%10	21.9_9
C%11(OCC1C2=C(C=CC=C2)C3=CC=CC=C31)=O.O=C%10CN%11.O=C(O)CN%10	22.1_1
C%11(OCC1C2=C(C=CC=C2)C3=CC=CC=C31)=O.O=C%10CN%11.CC(C)[CH](C(O)=O)N%10	22.1_2
C%11(OCC1C2=C(C=CC=C2)C3=CC=CC=C31)=O.O=C%10CN%11.CC(C)C[CH](C(O)=O)N%10	22.1_3
C%11(OCC1C2=C(C=CC=C2)C3=CC=CC=C31)=O.O=C%10CN%11.C[CH](C(O)=O)N%10	22.1_4
C%11(OCC1C2=C(C=CC=C2)C3=CC=CC=C31)=O.O=C%10CN%11.O=C(O)[CH](CC1=CC=CC=C1)N%10	22.1_5
C%11(OCC1C2=C(C=CC=C2)C3=CC=CC=C31)=O.O=C%10CN%11.CC[CH](C)[CH](C(O)=O)N%10	22.1_6
C%11(OCC1C2=C(C=CC=C2)C3=CC=CC=C31)=O.O=C%10CN%11.CSCC[CH](C(O)=O)N%10	22.1_7
C%11(OCC1C2=C(C=CC=C2)C3=CC=CC=C31)=O.O=C%10CN%11.OC(C=C1)=CC=C1C[CH](C(O)=O)N%10	22.1_8
C%11(OCC1C2=C(C=CC=C2)C3=CC=CC=C31)=O.O=C%10CN%11.O=C(O)[CH](CC1=C(F)C(F)=C(F)C(F)=C1F)N%10	22.1_9
C%11(OCC1C2=C(C=CC=C2)C3=CC=CC=C31)=O.CC(C)[CH](C%10=O)N%11.O=C(O)CN%10	22.2_1
C%11(OCC1C2=C(C=CC=C2)C3=CC=CC=C31)=O.CC(C)[CH](C%10=O)N%11.CC(C)[CH](C(O)=O)N%10	22.2_2
C%11(OCC1C2=C(C=CC=C2)C3=CC=CC=C31)=O.CC(C)[CH](C%10=O)N%11.CC(C)C[CH](C(O)=O)N%10	22.2_3
C%11(OCC1C2=C(C=CC=C2)C3=CC=CC=C31)=O.CC(C)[CH](C%10=O)N%11.C[CH](C(O)=O)N%10	22.2_4
C%11(OCC1C2=C(C=CC=C2)C3=CC=CC=C31)=O.CC(C)[CH](C%10=O)N%11.O=C(O)[CH](CC1=CC=CC=C1)N%10	22.2_5
C%11(OCC1C2=C(C=CC=C2)C3=CC=CC=C31)=O.CC(C)[CH](C%10=O)N%11.CC[CH](C)[CH](	22.2_6

C(O)=O)N%10	
C%11(OCC1C2=C(C=CC=C2)C3=CC=CC=C31)=O.CC(C)[CH](C%10=O)N%11.CSCC[CH](C(O)=O)N%10	22.2_7
C%11(OCC1C2=C(C=CC=C2)C3=CC=CC=C31)=O.CC(C)[CH](C%10=O)N%11.OC(C=C1)=CC=C1C[CH](C(O)=O)N%10	22.2_8
C%11(OCC1C2=C(C=CC=C2)C3=CC=CC=C31)=O.CC(C)[CH](C%10=O)N%11.O=C(O)[CH](CC1=C(F)C(F)=C(F)C(F)=C1F)N%10	22.2_9
C%11(OCC1C2=C(C=CC=C2)C3=CC=CC=C31)=O.CC(C)C[CH](C%10=O)N%11.O=C(O)CN%10	22.3_1
C%11(OCC1C2=C(C=CC=C2)C3=CC=CC=C31)=O.CC(C)C[CH](C%10=O)N%11.CC(C)[CH](C(O)=O)N%10	22.3_2
C%11(OCC1C2=C(C=CC=C2)C3=CC=CC=C31)=O.CC(C)C[CH](C%10=O)N%11.CC(C)C[CH](C(O)=O)N%10	22.3_3
C%11(OCC1C2=C(C=CC=C2)C3=CC=CC=C31)=O.CC(C)C[CH](C%10=O)N%11.C[CH](C(O)=O)N%10	22.3_4
C%11(OCC1C2=C(C=CC=C2)C3=CC=CC=C31)=O.CC(C)C[CH](C%10=O)N%11.O=C(O)[CH](CC1=CC=CC=C1)N%10	22.3_5
C%11(OCC1C2=C(C=CC=C2)C3=CC=CC=C31)=O.CC(C)C[CH](C%10=O)N%11.CC[CH](C)[CH](C(O)=O)N%10	22.3_6
C%11(OCC1C2=C(C=CC=C2)C3=CC=CC=C31)=O.CC(C)C[CH](C%10=O)N%11.CSCC[CH](C(O)=O)N%10	22.3_7
C%11(OCC1C2=C(C=CC=C2)C3=CC=CC=C31)=O.CC(C)C[CH](C%10=O)N%11.OC(C=C1)=CC=C1C[CH](C(O)=O)N%10	22.3_8
C%11(OCC1C2=C(C=CC=C2)C3=CC=CC=C31)=O.CC(C)C[CH](C%10=O)N%11.O=C(O)[CH](CC1=C(F)C(F)=C(F)C(F)=C1F)N%10	22.3_9
C%11(OCC1C2=C(C=CC=C2)C3=CC=CC=C31)=O.C[CH](C%10=O)N%11.O=C(O)CN%10	22.4_1
C%11(OCC1C2=C(C=CC=C2)C3=CC=CC=C31)=O.C[CH](C%10=O)N%11.CC(C)[CH](C(O)=O)N%10	22.4_2
C%11(OCC1C2=C(C=CC=C2)C3=CC=CC=C31)=O.C[CH](C%10=O)N%11.CC(C)C[CH](C(O)=O)N%10	22.4_3
C%11(OCC1C2=C(C=CC=C2)C3=CC=CC=C31)=O.C[CH](C%10=O)N%11.C[CH](C(O)=O)N%10	22.4_4
C%11(OCC1C2=C(C=CC=C2)C3=CC=CC=C31)=O.C[CH](C%10=O)N%11.O=C(O)[CH](CC1=CC=CC=C1)N%10	22.4_5
C%11(OCC1C2=C(C=CC=C2)C3=CC=CC=C31)=O.C[CH](C%10=O)N%11.CC[CH](C)[CH](C(O)=O)N%10	22.4_6
C%11(OCC1C2=C(C=CC=C2)C3=CC=CC=C31)=O.C[CH](C%10=O)N%11.CSCC[CH](C(O)=O)N%10	22.4_7
C%11(OCC1C2=C(C=CC=C2)C3=CC=CC=C31)=O.C[CH](C%10=O)N%11.OC(C=C1)=CC=C1C[CH](C(O)=O)N%10	22.4_8
C%11(OCC1C2=C(C=CC=C2)C3=CC=CC=C31)=O.C[CH](C%10=O)N%11.O=C(O)[CH](CC1=C(F)C(F)=C(F)C(F)=C1F)N%10	22.4_9
C%11(OCC1C2=C(C=CC=C2)C3=CC=CC=C31)=O.O=C%10[CH](CC1=CC=CC=C1)N%11.O=C(O)CN%10	22.5_1
C%11(OCC1C2=C(C=CC=C2)C3=CC=CC=C31)=O.O=C%10[CH](CC1=CC=CC=C1)N%11.CC(C)[CH](C(O)=O)N%10	22.5_2
C%11(OCC1C2=C(C=CC=C2)C3=CC=CC=C31)=O.O=C%10[CH](CC1=CC=CC=C1)N%11.CC(C)C[CH](C(O)=O)N%10	22.5_3
C%11(OCC1C2=C(C=CC=C2)C3=CC=CC=C31)=O.O=C%10[CH](CC1=CC=CC=C1)N%11.C[CH](C(O)=O)N%10	22.5_4

C%11(OCC1C2=C(C=CC=C2)C3=CC=CC=C31)=O.O=C%10[CH](CC1=CC=CC=C1)N%11.O=C(O)[CH](CC1=CC=CC=C1)N%10	22.5_5
C%11(OCC1C2=C(C=CC=C2)C3=CC=CC=C31)=O.O=C%10[CH](CC1=CC=CC=C1)N%11.CC[CH](C)[CH](C(O)=O)N%10	22.5_6
C%11(OCC1C2=C(C=CC=C2)C3=CC=CC=C31)=O.O=C%10[CH](CC1=CC=CC=C1)N%11.CSCC[CH](C(O)=O)N%10	22.5_7
C%11(OCC1C2=C(C=CC=C2)C3=CC=CC=C31)=O.O=C%10[CH](CC1=CC=CC=C1)N%11.OC(C=C1)=CC=C1C[CH](C(O)=O)N%10	22.5_8
C%11(OCC1C2=C(C=CC=C2)C3=CC=CC=C31)=O.O=C%10[CH](CC1=CC=CC=C1)N%11.O=C(O)[CH](CC1=C(F)C(F)=C(F)C(F)=C1F)N%10	22.5_9
C%11(OCC1C2=C(C=CC=C2)C3=CC=CC=C31)=O.CC[CH](C)[CH](C%10=O)N%11.O=C(O)CN%10	22.6_1
C%11(OCC1C2=C(C=CC=C2)C3=CC=CC=C31)=O.CC[CH](C)[CH](C%10=O)N%11.CC(C)[CH](C(O)=O)N%10	22.6_2
C%11(OCC1C2=C(C=CC=C2)C3=CC=CC=C31)=O.CC[CH](C)[CH](C%10=O)N%11.CC(C)C[CH](C(O)=O)N%10	22.6_3
C%11(OCC1C2=C(C=CC=C2)C3=CC=CC=C31)=O.CC[CH](C)[CH](C%10=O)N%11.C[CH](C(O)=O)N%10	22.6_4
C%11(OCC1C2=C(C=CC=C2)C3=CC=CC=C31)=O.CC[CH](C)[CH](C%10=O)N%11.O=C(O)[CH](CC1=CC=CC=C1)N%10	22.6_5
C%11(OCC1C2=C(C=CC=C2)C3=CC=CC=C31)=O.CC[CH](C)[CH](C%10=O)N%11.CC[CH](C)[CH](C(O)=O)N%10	22.6_6
C%11(OCC1C2=C(C=CC=C2)C3=CC=CC=C31)=O.CC[CH](C)[CH](C%10=O)N%11.CSCC[CH](C(O)=O)N%10	22.6_7
C%11(OCC1C2=C(C=CC=C2)C3=CC=CC=C31)=O.CC[CH](C)[CH](C%10=O)N%11.OC(C=C1)=CC=C1C[CH](C(O)=O)N%10	22.6_8
C%11(OCC1C2=C(C=CC=C2)C3=CC=CC=C31)=O.CC[CH](C)[CH](C%10=O)N%11.O=C(O)[CH](CC1=C(F)C(F)=C(F)C(F)=C1F)N%10	22.6_9
C%11(OCC1C2=C(C=CC=C2)C3=CC=CC=C31)=O.CSCC[CH](C%10=O)N%11.O=C(O)CN%10	22.7_1
C%11(OCC1C2=C(C=CC=C2)C3=CC=CC=C31)=O.CSCC[CH](C%10=O)N%11.CC(C)[CH](C(O)=O)N%10	22.7_2
C%11(OCC1C2=C(C=CC=C2)C3=CC=CC=C31)=O.CSCC[CH](C%10=O)N%11.CC(C)C[CH](C(O)=O)N%10	22.7_3
C%11(OCC1C2=C(C=CC=C2)C3=CC=CC=C31)=O.CSCC[CH](C%10=O)N%11.C[CH](C(O)=O)N%10	22.7_4
C%11(OCC1C2=C(C=CC=C2)C3=CC=CC=C31)=O.CSCC[CH](C%10=O)N%11.O=C(O)[CH](CC1=CC=CC=C1)N%10	22.7_5
C%11(OCC1C2=C(C=CC=C2)C3=CC=CC=C31)=O.CSCC[CH](C%10=O)N%11.CC[CH](C)[CH](C(O)=O)N%10	22.7_6
C%11(OCC1C2=C(C=CC=C2)C3=CC=CC=C31)=O.CSCC[CH](C%10=O)N%11.CSCC[CH](C(O)=O)N%10	22.7_7
C%11(OCC1C2=C(C=CC=C2)C3=CC=CC=C31)=O.CSCC[CH](C%10=O)N%11.OC(C=C1)=CC=C1C[CH](C(O)=O)N%10	22.7_8
C%11(OCC1C2=C(C=CC=C2)C3=CC=CC=C31)=O.CSCC[CH](C%10=O)N%11.O=C(O)[CH](CC1=C(F)C(F)=C(F)C(F)=C1F)N%10	22.7_9
C%11(OCC1C2=C(C=CC=C2)C3=CC=CC=C31)=O.OC(C=C1)=CC=C1C[CH](C%10=O)N%11.O=C(O)CN%10	22.8_1
C%11(OCC1C2=C(C=CC=C2)C3=CC=CC=C31)=O.OC(C=C1)=CC=C1C[CH](C%10=O)N%11.CC(C)[CH](C(O)=O)N%10	22.8_2

C%11(OCC1C2=C(C=CC=C2)C3=CC=CC=C31)=O.OC(C=C1)=CC=C1C[CH](C%10=O)N%11.CC(C)C[CH](C(O)=O)N%10	22.8_3
C%11(OCC1C2=C(C=CC=C2)C3=CC=CC=C31)=O.OC(C=C1)=CC=C1C[CH](C%10=O)N%11.C[CH](C(O)=O)N%10	22.8_4
C%11(OCC1C2=C(C=CC=C2)C3=CC=CC=C31)=O.OC(C=C1)=CC=C1C[CH](C%10=O)N%11.O=C(O)[CH](CC1=CC=CC=C1)N%10	22.8_5
C%11(OCC1C2=C(C=CC=C2)C3=CC=CC=C31)=O.OC(C=C1)=CC=C1C[CH](C%10=O)N%11.CC[CH](C)[CH](C(O)=O)N%10	22.8_6
C%11(OCC1C2=C(C=CC=C2)C3=CC=CC=C31)=O.OC(C=C1)=CC=C1C[CH](C%10=O)N%11.CSCC[CH](C(O)=O)N%10	22.8_7
C%11(OCC1C2=C(C=CC=C2)C3=CC=CC=C31)=O.OC(C=C1)=CC=C1C[CH](C%10=O)N%11.OC(C=C1)=CC=C1C[CH](C(O)=O)N%10	22.8_8
C%11(OCC1C2=C(C=CC=C2)C3=CC=CC=C31)=O.OC(C=C1)=CC=C1C[CH](C%10=O)N%11.O=C(O)[CH](CC1=C(F)C(F)=C(F)C(F)=C1F)N%10	22.8_9
C%10(OCC1C2=C(C=CC=C2)C3=CC=CC=C31)=O.O=C(O)CN%10	22.9_1
C%10(OCC1C2=C(C=CC=C2)C3=CC=CC=C31)=O.CC(C)[CH](C(O)=O)N%10	22.9_2
C%10(OCC1C2=C(C=CC=C2)C3=CC=CC=C31)=O.CC(C)C[CH](C(O)=O)N%10	22.9_3
C%10(OCC1C2=C(C=CC=C2)C3=CC=CC=C31)=O.C[CH](C(O)=O)N%10	22.9_4
C%10(OCC1C2=C(C=CC=C2)C3=CC=CC=C31)=O.O=C(O)[CH](CC1=CC=CC=C1)N%10	22.9_5
C%10(OCC1C2=C(C=CC=C2)C3=CC=CC=C31)=O.CC[CH](C)[CH](C(O)=O)N%10	22.9_6
C%10(OCC1C2=C(C=CC=C2)C3=CC=CC=C31)=O.CSCC[CH](C(O)=O)N%10	22.9_7
C%10(OCC1C2=C(C=CC=C2)C3=CC=CC=C31)=O.OC(C=C1)=CC=C1C[CH](C(O)=O)N%10	22.9_8
C%10(OCC1C2=C(C=CC=C2)C3=CC=CC=C31)=O.O=C(O)[CH](CC1=C(F)C(F)=C(F)C(F)=C1F)N%10	22.9_9
C%11(COC1=CC2=CC=C(C#N)C=C2C=C1)=O.O=C%10CN%11.O=C(O)CN%10	23.1_1
C%11(COC1=CC2=CC=C(C#N)C=C2C=C1)=O.O=C%10CN%11.CC(C)[CH](C(O)=O)N%10	23.1_2
C%11(COC1=CC2=CC=C(C#N)C=C2C=C1)=O.O=C%10CN%11.CC(C)C[CH](C(O)=O)N%10	23.1_3
C%11(COC1=CC2=CC=C(C#N)C=C2C=C1)=O.O=C%10CN%11.C[CH](C(O)=O)N%10	23.1_4
C%11(COC1=CC2=CC=C(C#N)C=C2C=C1)=O.O=C%10CN%11.O=C(O)[CH](CC1=CC=CC=C1)N%10	23.1_5
C%11(COC1=CC2=CC=C(C#N)C=C2C=C1)=O.O=C%10CN%11.CC[CH](C)[CH](C(O)=O)N%10	23.1_6
C%11(COC1=CC2=CC=C(C#N)C=C2C=C1)=O.O=C%10CN%11.CSCC[CH](C(O)=O)N%10	23.1_7
C%11(COC1=CC2=CC=C(C#N)C=C2C=C1)=O.O=C%10CN%11.OC(C=C1)=CC=C1C[CH](C(O)=O)N%10	23.1_8
C%11(COC1=CC2=CC=C(C#N)C=C2C=C1)=O.O=C%10CN%11.O=C(O)[CH](CC1=C(F)C(F)=C(F)C(F)=C1F)N%10	23.1_9
C%11(COC1=CC2=CC=C(C#N)C=C2C=C1)=O.CC(C)[CH](C%10=O)N%11.O=C(O)CN%10	23.2_1
C%11(COC1=CC2=CC=C(C#N)C=C2C=C1)=O.CC(C)[CH](C%10=O)N%11.CC(C)[CH](C(O)=O)N%10	23.2_2
C%11(COC1=CC2=CC=C(C#N)C=C2C=C1)=O.CC(C)[CH](C%10=O)N%11.CC(C)C[CH](C(O)=O)N%10	23.2_3
C%11(COC1=CC2=CC=C(C#N)C=C2C=C1)=O.CC(C)[CH](C%10=O)N%11.C[CH](C(O)=O)N%10	23.2_4
C%11(COC1=CC2=CC=C(C#N)C=C2C=C1)=O.CC(C)[CH](C%10=O)N%11.O=C(O)[CH](CC1=CC=CC=C1)N%10	23.2_5
C%11(COC1=CC2=CC=C(C#N)C=C2C=C1)=O.CC(C)[CH](C%10=O)N%11.CC[CH](C)[CH](C(O)=O)N%10	23.2_6

C%11(COC1=CC2=CC=C(C#N)C=C2C=C1)=O.CC(C)[CH](C%10=O)N%11.CSCC[CH](C(O)=O)N%10	23.2_7
C%11(COC1=CC2=CC=C(C#N)C=C2C=C1)=O.CC(C)[CH](C%10=O)N%11.OC(C=C1)=CC=C1C[CH](C(O)=O)N%10	23.2_8
C%11(COC1=CC2=CC=C(C#N)C=C2C=C1)=O.CC(C)[CH](C%10=O)N%11.O=C(O)[CH](CC1=C(F)C(F)=C(F)C(F)=C1F)N%10	23.2_9
C%11(COC1=CC2=CC=C(C#N)C=C2C=C1)=O.CC(C)C[CH](C%10=O)N%11.O=C(O)CN%10	23.3_1
C%11(COC1=CC2=CC=C(C#N)C=C2C=C1)=O.CC(C)C[CH](C%10=O)N%11.CC(C)[CH](C(O)=O)N%10	23.3_2
C%11(COC1=CC2=CC=C(C#N)C=C2C=C1)=O.CC(C)C[CH](C%10=O)N%11.CC(C)C[CH](C(O)=O)N%10	23.3_3
C%11(COC1=CC2=CC=C(C#N)C=C2C=C1)=O.CC(C)C[CH](C%10=O)N%11.C[CH](C(O)=O)N%10	23.3_4
C%11(COC1=CC2=CC=C(C#N)C=C2C=C1)=O.CC(C)C[CH](C%10=O)N%11.O=C(O)[CH](CC1=CC=CC=C1)N%10	23.3_5
C%11(COC1=CC2=CC=C(C#N)C=C2C=C1)=O.CC(C)C[CH](C%10=O)N%11.CC[CH](C)[CH](C(O)=O)N%10	23.3_6
C%11(COC1=CC2=CC=C(C#N)C=C2C=C1)=O.CC(C)C[CH](C%10=O)N%11.CSCC[CH](C(O)=O)N%10	23.3_7
C%11(COC1=CC2=CC=C(C#N)C=C2C=C1)=O.CC(C)C[CH](C%10=O)N%11.OC(C=C1)=CC=C1C[CH](C(O)=O)N%10	23.3_8
C%11(COC1=CC2=CC=C(C#N)C=C2C=C1)=O.CC(C)C[CH](C%10=O)N%11.O=C(O)[CH](CC1=C(F)C(F)=C(F)C(F)=C1F)N%10	23.3_9
C%11(COC1=CC2=CC=C(C#N)C=C2C=C1)=O.C[CH](C%10=O)N%11.O=C(O)CN%10	23.4_1
C%11(COC1=CC2=CC=C(C#N)C=C2C=C1)=O.C[CH](C%10=O)N%11.CC(C)[CH](C(O)=O)N%10	23.4_2
C%11(COC1=CC2=CC=C(C#N)C=C2C=C1)=O.C[CH](C%10=O)N%11.CC(C)C[CH](C(O)=O)N%10	23.4_3
C%11(COC1=CC2=CC=C(C#N)C=C2C=C1)=O.C[CH](C%10=O)N%11.C[CH](C(O)=O)N%10	23.4_4
C%11(COC1=CC2=CC=C(C#N)C=C2C=C1)=O.C[CH](C%10=O)N%11.O=C(O)[CH](CC1=CC=CC=C1)N%10	23.4_5
C%11(COC1=CC2=CC=C(C#N)C=C2C=C1)=O.C[CH](C%10=O)N%11.CC[CH](C)[CH](C(O)=O)N%10	23.4_6
C%11(COC1=CC2=CC=C(C#N)C=C2C=C1)=O.C[CH](C%10=O)N%11.CSCC[CH](C(O)=O)N%10	23.4_7
C%11(COC1=CC2=CC=C(C#N)C=C2C=C1)=O.C[CH](C%10=O)N%11.OC(C=C1)=CC=C1C[CH](C(O)=O)N%10	23.4_8
C%11(COC1=CC2=CC=C(C#N)C=C2C=C1)=O.C[CH](C%10=O)N%11.O=C(O)[CH](CC1=C(F)C(F)=C(F)C(F)=C1F)N%10	23.4_9
C%11(COC1=CC2=CC=C(C#N)C=C2C=C1)=O.O=C%10[CH](CC1=CC=CC=C1)N%11.O=C(O)CN%10	23.5_1
C%11(COC1=CC2=CC=C(C#N)C=C2C=C1)=O.O=C%10[CH](CC1=CC=CC=C1)N%11.CC(C)[CH](C(O)=O)N%10	23.5_2
C%11(COC1=CC2=CC=C(C#N)C=C2C=C1)=O.O=C%10[CH](CC1=CC=CC=C1)N%11.CC(C)C[CH](C(O)=O)N%10	23.5_3
C%11(COC1=CC2=CC=C(C#N)C=C2C=C1)=O.O=C%10[CH](CC1=CC=CC=C1)N%11.C[CH](C(O)=O)N%10	23.5_4
C%11(COC1=CC2=CC=C(C#N)C=C2C=C1)=O.O=C%10[CH](CC1=CC=CC=C1)N%11.O=C(O)[CH](CC1=CC=CC=C1)N%10	23.5_5

C%11(COC1=CC2=CC=C(C#N)C=C2C=C1)=O.O=C%10[CH](CC1=CC=CC=C1)N%11.CC[CH](C)[CH](C(O)=O)N%10	23.5_6
C%11(COC1=CC2=CC=C(C#N)C=C2C=C1)=O.O=C%10[CH](CC1=CC=CC=C1)N%11.CSCC[CH](C(O)=O)N%10	23.5_7
C%11(COC1=CC2=CC=C(C#N)C=C2C=C1)=O.O=C%10[CH](CC1=CC=CC=C1)N%11.OC(C=C1)=CC=C1C[CH](C(O)=O)N%10	23.5_8
C%11(COC1=CC2=CC=C(C#N)C=C2C=C1)=O.O=C%10[CH](CC1=CC=CC=C1)N%11.O=C(O)[CH](CC1=C(F)C(F)=C(F)C(F)=C1F)N%10	23.5_9
C%11(COC1=CC2=CC=C(C#N)C=C2C=C1)=O.CC[CH](C)[CH](C%10=O)N%11.O=C(O)CN%10	23.6_1
C%11(COC1=CC2=CC=C(C#N)C=C2C=C1)=O.CC[CH](C)[CH](C%10=O)N%11.CC(C)[CH](C(O)=O)N%10	23.6_2
C%11(COC1=CC2=CC=C(C#N)C=C2C=C1)=O.CC[CH](C)[CH](C%10=O)N%11.CC(C)C[CH](C(O)=O)N%10	23.6_3
C%11(COC1=CC2=CC=C(C#N)C=C2C=C1)=O.CC[CH](C)[CH](C%10=O)N%11.C[CH](C(O)=O)N%10	23.6_4
C%11(COC1=CC2=CC=C(C#N)C=C2C=C1)=O.CC[CH](C)[CH](C%10=O)N%11.O=C(O)[CH](CC1=CC=CC=C1)N%10	23.6_5
C%11(COC1=CC2=CC=C(C#N)C=C2C=C1)=O.CC[CH](C)[CH](C%10=O)N%11.CC[CH](C)[CH](C(O)=O)N%10	23.6_6
C%11(COC1=CC2=CC=C(C#N)C=C2C=C1)=O.CC[CH](C)[CH](C%10=O)N%11.CSCC[CH](C(O)=O)N%10	23.6_7
C%11(COC1=CC2=CC=C(C#N)C=C2C=C1)=O.CC[CH](C)[CH](C%10=O)N%11.OC(C=C1)=CC=C1C[CH](C(O)=O)N%10	23.6_8
C%11(COC1=CC2=CC=C(C#N)C=C2C=C1)=O.CC[CH](C)[CH](C%10=O)N%11.O=C(O)[CH](CC1=C(F)C(F)=C(F)C(F)=C1F)N%10	23.6_9
C%11(COC1=CC2=CC=C(C#N)C=C2C=C1)=O.CSCC[CH](C%10=O)N%11.O=C(O)CN%10	23.7_1
C%11(COC1=CC2=CC=C(C#N)C=C2C=C1)=O.CSCC[CH](C%10=O)N%11.CC(C)[CH](C(O)=O)N%10	23.7_2
C%11(COC1=CC2=CC=C(C#N)C=C2C=C1)=O.CSCC[CH](C%10=O)N%11.CC(C)C[CH](C(O)=O)N%10	23.7_3
C%11(COC1=CC2=CC=C(C#N)C=C2C=C1)=O.CSCC[CH](C%10=O)N%11.C[CH](C(O)=O)N%10	23.7_4
C%11(COC1=CC2=CC=C(C#N)C=C2C=C1)=O.CSCC[CH](C%10=O)N%11.O=C(O)[CH](CC1=CC=CC=C1)N%10	23.7_5
C%11(COC1=CC2=CC=C(C#N)C=C2C=C1)=O.CSCC[CH](C%10=O)N%11.CC[CH](C)[CH](C(O)=O)N%10	23.7_6
C%11(COC1=CC2=CC=C(C#N)C=C2C=C1)=O.CSCC[CH](C%10=O)N%11.CSCC[CH](C(O)=O)N%10	23.7_7
C%11(COC1=CC2=CC=C(C#N)C=C2C=C1)=O.CSCC[CH](C%10=O)N%11.OC(C=C1)=CC=C1C[CH](C(O)=O)N%10	23.7_8
C%11(COC1=CC2=CC=C(C#N)C=C2C=C1)=O.CSCC[CH](C%10=O)N%11.O=C(O)[CH](CC1=C(F)C(F)=C(F)C(F)=C1F)N%10	23.7_9
C%11(COC1=CC2=CC=C(C#N)C=C2C=C1)=O.OC(C=C1)=CC=C1C[CH](C%10=O)N%11.O=C(O)CN%10	23.8_1
C%11(COC1=CC2=CC=C(C#N)C=C2C=C1)=O.OC(C=C1)=CC=C1C[CH](C%10=O)N%11.CC(C)[CH](C(O)=O)N%10	23.8_2
C%11(COC1=CC2=CC=C(C#N)C=C2C=C1)=O.OC(C=C1)=CC=C1C[CH](C%10=O)N%11.CC(C)C[CH](C(O)=O)N%10	23.8_3
C%11(COC1=CC2=CC=C(C#N)C=C2C=C1)=O.OC(C=C1)=CC=C1C[CH](C%10=O)N%11.C[CH]	23.8_4

(C(O)=O)N%10	
C%11(COC1=CC2=CC=C(C#N)C=C2C=C1)=O.OC(C=C1)=CC=C1C[CH](C%10=O)N%11.O=C(O)[CH](CC1=CC=CC=C1)N%10	23.8_5
C%11(COC1=CC2=CC=C(C#N)C=C2C=C1)=O.OC(C=C1)=CC=C1C[CH](C%10=O)N%11.CC[CH](C)[CH](C(O)=O)N%10	23.8_6
C%11(COC1=CC2=CC=C(C#N)C=C2C=C1)=O.OC(C=C1)=CC=C1C[CH](C%10=O)N%11.CSCC[CH](C(O)=O)N%10	23.8_7
C%11(COC1=CC2=CC=C(C#N)C=C2C=C1)=O.OC(C=C1)=CC=C1C[CH](C%10=O)N%11.OC(C=C1)=CC=C1C[CH](C(O)=O)N%10	23.8_8
C%11(COC1=CC2=CC=C(C#N)C=C2C=C1)=O.OC(C=C1)=CC=C1C[CH](C%10=O)N%11.O=C(O)[CH](CC1=C(F)C(F)=C(F)C(F)=C1F)N%10	23.8_9
C%10(COC1=CC2=CC=C(C#N)C=C2C=C1)=O.O=C(O)CN%10	23.9_1
C%10(COC1=CC2=CC=C(C#N)C=C2C=C1)=O.CC(C)[CH](C(O)=O)N%10	23.9_2
C%10(COC1=CC2=CC=C(C#N)C=C2C=C1)=O.CC(C)C[CH](C(O)=O)N%10	23.9_3
C%10(COC1=CC2=CC=C(C#N)C=C2C=C1)=O.C[CH](C(O)=O)N%10	23.9_4
C%10(COC1=CC2=CC=C(C#N)C=C2C=C1)=O.O=C(O)[CH](CC1=CC=CC=C1)N%10	23.9_5
C%10(COC1=CC2=CC=C(C#N)C=C2C=C1)=O.CC[CH](C)[CH](C(O)=O)N%10	23.9_6
C%10(COC1=CC2=CC=C(C#N)C=C2C=C1)=O.CSCC[CH](C(O)=O)N%10	23.9_7
C%10(COC1=CC2=CC=C(C#N)C=C2C=C1)=O.OC(C=C1)=CC=C1C[CH](C(O)=O)N%10	23.9_8
C%10(COC1=CC2=CC=C(C#N)C=C2C=C1)=O.O=C(O)[CH](CC1=C(F)C(F)=C(F)C(F)=C1F)N%10	23.9_9
C%11(COC1=CC2=CC=C(OC)C=C2C=C1)=O.O=C%10CN%11.O=C(O)CN%10	24.1_1
C%11(COC1=CC2=CC=C(OC)C=C2C=C1)=O.O=C%10CN%11.CC(C)[CH](C(O)=O)N%10	24.1_2
C%11(COC1=CC2=CC=C(OC)C=C2C=C1)=O.O=C%10CN%11.CC(C)C[CH](C(O)=O)N%10	24.1_3
C%11(COC1=CC2=CC=C(OC)C=C2C=C1)=O.O=C%10CN%11.C[CH](C(O)=O)N%10	24.1_4
C%11(COC1=CC2=CC=C(OC)C=C2C=C1)=O.O=C%10CN%11.O=C(O)[CH](CC1=CC=CC=C1)N%10	24.1_5
C%11(COC1=CC2=CC=C(OC)C=C2C=C1)=O.O=C%10CN%11.CC[CH](C)[CH](C(O)=O)N%10	24.1_6
C%11(COC1=CC2=CC=C(OC)C=C2C=C1)=O.O=C%10CN%11.CSCC[CH](C(O)=O)N%10	24.1_7
C%11(COC1=CC2=CC=C(OC)C=C2C=C1)=O.O=C%10CN%11.OC(C=C1)=CC=C1C[CH](C(O)=O)N%10	24.1_8
C%11(COC1=CC2=CC=C(OC)C=C2C=C1)=O.O=C%10CN%11.O=C(O)[CH](CC1=C(F)C(F)=C(F)C(F)=C1F)N%10	24.1_9
C%11(COC1=CC2=CC=C(OC)C=C2C=C1)=O.CC(C)[CH](C%10=O)N%11.O=C(O)CN%10	24.2_1
C%11(COC1=CC2=CC=C(OC)C=C2C=C1)=O.CC(C)[CH](C%10=O)N%11.CC(C)[CH](C(O)=O)N%10	24.2_2
C%11(COC1=CC2=CC=C(OC)C=C2C=C1)=O.CC(C)[CH](C%10=O)N%11.CC(C)C[CH](C(O)=O)N%10	24.2_3
C%11(COC1=CC2=CC=C(OC)C=C2C=C1)=O.CC(C)[CH](C%10=O)N%11.C[CH](C(O)=O)N%10	24.2_4
C%11(COC1=CC2=CC=C(OC)C=C2C=C1)=O.CC(C)[CH](C%10=O)N%11.O=C(O)[CH](CC1=CC=CC=C1)N%10	24.2_5
C%11(COC1=CC2=CC=C(OC)C=C2C=C1)=O.CC(C)[CH](C%10=O)N%11.CC[CH](C)[CH](C(O)=O)N%10	24.2_6
C%11(COC1=CC2=CC=C(OC)C=C2C=C1)=O.CC(C)[CH](C%10=O)N%11.CSCC[CH](C(O)=O)N%10	24.2_7
C%11(COC1=CC2=CC=C(OC)C=C2C=C1)=O.CC(C)[CH](C%10=O)N%11.OC(C=C1)=CC=C1C[CH](C(O)=O)N%10	24.2_8

C%11(COC1=CC2=CC=C(OC)C=C2C=C1)=O.CC(C)[CH](C%10=O)N%11.O=C(O)[CH](CC1=C(F)C(F)=C(F)C(F)=C1F)N%10	24.2_9
C%11(COC1=CC2=CC=C(OC)C=C2C=C1)=O.CC(C)C[CH](C%10=O)N%11.O=C(O)CN%10	24.3_1
C%11(COC1=CC2=CC=C(OC)C=C2C=C1)=O.CC(C)C[CH](C%10=O)N%11.CC(C)[CH](C(O)=O)N%10	24.3_2
C%11(COC1=CC2=CC=C(OC)C=C2C=C1)=O.CC(C)C[CH](C%10=O)N%11.CC(C)C[CH](C(O)=O)N%10	24.3_3
C%11(COC1=CC2=CC=C(OC)C=C2C=C1)=O.CC(C)C[CH](C%10=O)N%11.C[CH](C(O)=O)N%10	24.3_4
C%11(COC1=CC2=CC=C(OC)C=C2C=C1)=O.CC(C)C[CH](C%10=O)N%11.O=C(O)[CH](CC1=CC=CC=C1)N%10	24.3_5
C%11(COC1=CC2=CC=C(OC)C=C2C=C1)=O.CC(C)C[CH](C%10=O)N%11.CC[CH](C)[CH](C(O)=O)N%10	24.3_6
C%11(COC1=CC2=CC=C(OC)C=C2C=C1)=O.CC(C)C[CH](C%10=O)N%11.CSCC[CH](C(O)=O)N%10	24.3_7
C%11(COC1=CC2=CC=C(OC)C=C2C=C1)=O.CC(C)C[CH](C%10=O)N%11.OC(C=C1)=CC=C1C[CH](C(O)=O)N%10	24.3_8
C%11(COC1=CC2=CC=C(OC)C=C2C=C1)=O.CC(C)C[CH](C%10=O)N%11.O=C(O)[CH](CC1=C(F)C(F)=C(F)C(F)=C1F)N%10	24.3_9
C%11(COC1=CC2=CC=C(OC)C=C2C=C1)=O.C[CH](C%10=O)N%11.O=C(O)CN%10	24.4_1
C%11(COC1=CC2=CC=C(OC)C=C2C=C1)=O.C[CH](C%10=O)N%11.CC(C)[CH](C(O)=O)N%10	24.4_2
C%11(COC1=CC2=CC=C(OC)C=C2C=C1)=O.C[CH](C%10=O)N%11.CC(C)C[CH](C(O)=O)N%10	24.4_3
C%11(COC1=CC2=CC=C(OC)C=C2C=C1)=O.C[CH](C%10=O)N%11.C[CH](C(O)=O)N%10	24.4_4
C%11(COC1=CC2=CC=C(OC)C=C2C=C1)=O.C[CH](C%10=O)N%11.O=C(O)[CH](CC1=CC=CC=C1)N%10	24.4_5
C%11(COC1=CC2=CC=C(OC)C=C2C=C1)=O.C[CH](C%10=O)N%11.CC[CH](C)[CH](C(O)=O)N%10	24.4_6
C%11(COC1=CC2=CC=C(OC)C=C2C=C1)=O.C[CH](C%10=O)N%11.CSCC[CH](C(O)=O)N%10	24.4_7
C%11(COC1=CC2=CC=C(OC)C=C2C=C1)=O.C[CH](C%10=O)N%11.OC(C=C1)=CC=C1C[CH](C(O)=O)N%10	24.4_8
C%11(COC1=CC2=CC=C(OC)C=C2C=C1)=O.C[CH](C%10=O)N%11.O=C(O)[CH](CC1=C(F)C(F)=C(F)C(F)=C1F)N%10	24.4_9
C%11(COC1=CC2=CC=C(OC)C=C2C=C1)=O.O=C%10[CH](CC1=CC=CC=C1)N%11.O=C(O)CN%10	24.5_1
C%11(COC1=CC2=CC=C(OC)C=C2C=C1)=O.O=C%10[CH](CC1=CC=CC=C1)N%11.CC(C)[CH](C(O)=O)N%10	24.5_2
C%11(COC1=CC2=CC=C(OC)C=C2C=C1)=O.O=C%10[CH](CC1=CC=CC=C1)N%11.CC(C)C[CH](C(O)=O)N%10	24.5_3
C%11(COC1=CC2=CC=C(OC)C=C2C=C1)=O.O=C%10[CH](CC1=CC=CC=C1)N%11.C[CH](C(O)=O)N%10	24.5_4
C%11(COC1=CC2=CC=C(OC)C=C2C=C1)=O.O=C%10[CH](CC1=CC=CC=C1)N%11.O=C(O)[CH](CC1=CC=CC=C1)N%10	24.5_5
C%11(COC1=CC2=CC=C(OC)C=C2C=C1)=O.O=C%10[CH](CC1=CC=CC=C1)N%11.CC[CH](C)[CH](C(O)=O)N%10	24.5_6
C%11(COC1=CC2=CC=C(OC)C=C2C=C1)=O.O=C%10[CH](CC1=CC=CC=C1)N%11.CSCC[CH](C(O)=O)N%10	24.5_7
C%11(COC1=CC2=CC=C(OC)C=C2C=C1)=O.O=C%10[CH](CC1=CC=CC=C1)N%11.OC(C=C1)=CC=C1C[CH](C(O)=O)N%10	24.5_8

C%11(COC1=CC2=CC=C(OC)C=C2C=C1)=O.O=C%10[CH](CC1=CC=CC=C1)N%11.O=C(O)[CH](CC1=C(F)C(F)=C(F)C(F)=C1F)N%10	24.5_9
C%11(COC1=CC2=CC=C(OC)C=C2C=C1)=O.CC[CH](C)[CH](C%10=O)N%11.O=C(O)CN%10	24.6_1
C%11(COC1=CC2=CC=C(OC)C=C2C=C1)=O.CC[CH](C)[CH](C%10=O)N%11.CC(C)[CH](C(O)=O)N%10	24.6_2
C%11(COC1=CC2=CC=C(OC)C=C2C=C1)=O.CC[CH](C)[CH](C%10=O)N%11.CC(C)C[CH](C(O)=O)N%10	24.6_3
C%11(COC1=CC2=CC=C(OC)C=C2C=C1)=O.CC[CH](C)[CH](C%10=O)N%11.C[CH](C(O)=O)N%10	24.6_4
C%11(COC1=CC2=CC=C(OC)C=C2C=C1)=O.CC[CH](C)[CH](C%10=O)N%11.O=C(O)[CH](CC1=CC=CC=C1)N%10	24.6_5
C%11(COC1=CC2=CC=C(OC)C=C2C=C1)=O.CC[CH](C)[CH](C%10=O)N%11.CC[CH](C)[CH](C(O)=O)N%10	24.6_6
C%11(COC1=CC2=CC=C(OC)C=C2C=C1)=O.CC[CH](C)[CH](C%10=O)N%11.CSCC[CH](C(O)=O)N%10	24.6_7
C%11(COC1=CC2=CC=C(OC)C=C2C=C1)=O.CC[CH](C)[CH](C%10=O)N%11.OC(C=C1)=CC=C1C[CH](C(O)=O)N%10	24.6_8
C%11(COC1=CC2=CC=C(OC)C=C2C=C1)=O.CC[CH](C)[CH](C%10=O)N%11.O=C(O)[CH](CC1=C(F)C(F)=C(F)C(F)=C1F)N%10	24.6_9
C%11(COC1=CC2=CC=C(OC)C=C2C=C1)=O.CSCC[CH](C%10=O)N%11.O=C(O)CN%10	24.7_1
C%11(COC1=CC2=CC=C(OC)C=C2C=C1)=O.CSCC[CH](C%10=O)N%11.CC(C)[CH](C(O)=O)N%10	24.7_2
C%11(COC1=CC2=CC=C(OC)C=C2C=C1)=O.CSCC[CH](C%10=O)N%11.CC(C)C[CH](C(O)=O)N%10	24.7_3
C%11(COC1=CC2=CC=C(OC)C=C2C=C1)=O.CSCC[CH](C%10=O)N%11.C[CH](C(O)=O)N%10	24.7_4
C%11(COC1=CC2=CC=C(OC)C=C2C=C1)=O.CSCC[CH](C%10=O)N%11.O=C(O)[CH](CC1=CC=CC=C1)N%10	24.7_5
C%11(COC1=CC2=CC=C(OC)C=C2C=C1)=O.CSCC[CH](C%10=O)N%11.CC[CH](C)[CH](C(O)=O)N%10	24.7_6
C%11(COC1=CC2=CC=C(OC)C=C2C=C1)=O.CSCC[CH](C%10=O)N%11.CSCC[CH](C(O)=O)N%10	24.7_7
C%11(COC1=CC2=CC=C(OC)C=C2C=C1)=O.CSCC[CH](C%10=O)N%11.OC(C=C1)=CC=C1C[CH](C(O)=O)N%10	24.7_8
C%11(COC1=CC2=CC=C(OC)C=C2C=C1)=O.CSCC[CH](C%10=O)N%11.O=C(O)[CH](CC1=C(F)C(F)=C(F)C(F)=C1F)N%10	24.7_9
C%11(COC1=CC2=CC=C(OC)C=C2C=C1)=O.OC(C=C1)=CC=C1C[CH](C%10=O)N%11.O=C(O)CN%10	24.8_1
C%11(COC1=CC2=CC=C(OC)C=C2C=C1)=O.OC(C=C1)=CC=C1C[CH](C%10=O)N%11.CC(C)[CH](C(O)=O)N%10	24.8_2
C%11(COC1=CC2=CC=C(OC)C=C2C=C1)=O.OC(C=C1)=CC=C1C[CH](C%10=O)N%11.CC(C)C[CH](C(O)=O)N%10	24.8_3
C%11(COC1=CC2=CC=C(OC)C=C2C=C1)=O.OC(C=C1)=CC=C1C[CH](C%10=O)N%11.C[CH](C(O)=O)N%10	24.8_4
C%11(COC1=CC2=CC=C(OC)C=C2C=C1)=O.OC(C=C1)=CC=C1C[CH](C%10=O)N%11.O=C(O)[CH](CC1=CC=CC=C1)N%10	24.8_5
C%11(COC1=CC2=CC=C(OC)C=C2C=C1)=O.OC(C=C1)=CC=C1C[CH](C%10=O)N%11.CC[CH](C)[CH](C(O)=O)N%10	24.8_6
C%11(COC1=CC2=CC=C(OC)C=C2C=C1)=O.OC(C=C1)=CC=C1C[CH](C%10=O)N%11.CSCC[CH](C(O)=O)N%10	24.8_7

C%11(COC1=CC2=CC=C(OC)C=C2C=C1)=O.OC(C=C1)=CC=C1C[CH](C%10=O)N%11.OC(C=C1)=CC=C1C[CH](C(O)=O)N%10	24.8_8
C%11(COC1=CC2=CC=C(OC)C=C2C=C1)=O.OC(C=C1)=CC=C1C[CH](C%10=O)N%11.O=C(O)[CH](CC1=C(F)C(F)=C(F)C(F)=C1F)N%10	24.8_9
C%10(COC1=CC2=CC=C(OC)C=C2C=C1)=O.O=C(O)CN%10	24.9_1
C%10(COC1=CC2=CC=C(OC)C=C2C=C1)=O.CC(C)[CH](C(O)=O)N%10	24.9_2
C%10(COC1=CC2=CC=C(OC)C=C2C=C1)=O.CC(C)C[CH](C(O)=O)N%10	24.9_3
C%10(COC1=CC2=CC=C(OC)C=C2C=C1)=O.C[CH](C(O)=O)N%10	24.9_4
C%10(COC1=CC2=CC=C(OC)C=C2C=C1)=O.O=C(O)[CH](CC1=CC=CC=C1)N%10	24.9_5
C%10(COC1=CC2=CC=C(OC)C=C2C=C1)=O.CC[CH](C)[CH](C(O)=O)N%10	24.9_6
C%10(COC1=CC2=CC=C(OC)C=C2C=C1)=O.CSCC[CH](C(O)=O)N%10	24.9_7
C%10(COC1=CC2=CC=C(OC)C=C2C=C1)=O.OC(C=C1)=CC=C1C[CH](C(O)=O)N%10	24.9_8
C%10(COC1=CC2=CC=C(OC)C=C2C=C1)=O.O=C(O)[CH](CC1=C(F)C(F)=C(F)C(F)=C1F)N%10	24.9_9
C%11(COC1=CC2=CC=C(Br)C=C2C=C1)=O.O=C%10CN%11.O=C(O)CN%10	25.1_1
C%11(COC1=CC2=CC=C(Br)C=C2C=C1)=O.O=C%10CN%11.CC(C)[CH](C(O)=O)N%10	25.1_2
C%11(COC1=CC2=CC=C(Br)C=C2C=C1)=O.O=C%10CN%11.CC(C)C[CH](C(O)=O)N%10	25.1_3
C%11(COC1=CC2=CC=C(Br)C=C2C=C1)=O.O=C%10CN%11.C[CH](C(O)=O)N%10	25.1_4
C%11(COC1=CC2=CC=C(Br)C=C2C=C1)=O.O=C%10CN%11.O=C(O)[CH](CC1=CC=CC=C1)N%10	25.1_5
C%11(COC1=CC2=CC=C(Br)C=C2C=C1)=O.O=C%10CN%11.CC[CH](C)[CH](C(O)=O)N%10	25.1_6
C%11(COC1=CC2=CC=C(Br)C=C2C=C1)=O.O=C%10CN%11.CSCC[CH](C(O)=O)N%10	25.1_7
C%11(COC1=CC2=CC=C(Br)C=C2C=C1)=O.O=C%10CN%11.OC(C=C1)=CC=C1C[CH](C(O)=O)N%10	25.1_8
C%11(COC1=CC2=CC=C(Br)C=C2C=C1)=O.O=C%10CN%11.O=C(O)[CH](CC1=C(F)C(F)=C(F)C(F)=C1F)N%10	25.1_9
C%11(COC1=CC2=CC=C(Br)C=C2C=C1)=O.CC(C)[CH](C%10=O)N%11.O=C(O)CN%10	25.2_1
C%11(COC1=CC2=CC=C(Br)C=C2C=C1)=O.CC(C)[CH](C%10=O)N%11.CC(C)[CH](C(O)=O)N%10	25.2_2
C%11(COC1=CC2=CC=C(Br)C=C2C=C1)=O.CC(C)[CH](C%10=O)N%11.CC(C)C[CH](C(O)=O)N%10	25.2_3
C%11(COC1=CC2=CC=C(Br)C=C2C=C1)=O.CC(C)[CH](C%10=O)N%11.C[CH](C(O)=O)N%10	25.2_4
C%11(COC1=CC2=CC=C(Br)C=C2C=C1)=O.CC(C)[CH](C%10=O)N%11.O=C(O)[CH](CC1=CC=CC=C1)N%10	25.2_5
C%11(COC1=CC2=CC=C(Br)C=C2C=C1)=O.CC(C)[CH](C%10=O)N%11.CC[CH](C)[CH](C(O)=O)N%10	25.2_6
C%11(COC1=CC2=CC=C(Br)C=C2C=C1)=O.CC(C)[CH](C%10=O)N%11.CSCC[CH](C(O)=O)N%10	25.2_7
C%11(COC1=CC2=CC=C(Br)C=C2C=C1)=O.CC(C)[CH](C%10=O)N%11.OC(C=C1)=CC=C1C[CH](C(O)=O)N%10	25.2_8
C%11(COC1=CC2=CC=C(Br)C=C2C=C1)=O.CC(C)[CH](C%10=O)N%11.O=C(O)[CH](CC1=C(F)C(F)=C(F)C(F)=C1F)N%10	25.2_9
C%11(COC1=CC2=CC=C(Br)C=C2C=C1)=O.CC(C)C[CH](C%10=O)N%11.O=C(O)CN%10	25.3_1
C%11(COC1=CC2=CC=C(Br)C=C2C=C1)=O.CC(C)C[CH](C%10=O)N%11.CC(C)[CH](C(O)=O)N%10	25.3_2
C%11(COC1=CC2=CC=C(Br)C=C2C=C1)=O.CC(C)C[CH](C%10=O)N%11.CC(C)C[CH](C(O)=O)N%10	25.3_3
C%11(COC1=CC2=CC=C(Br)C=C2C=C1)=O.CC(C)C[CH](C%10=O)N%11.C[CH](C(O)=O)N%1	25.3_4

0	
C%11(COC1=CC2=CC=C(Br)C=C2C=C1)=O.CC(C)C[CH](C%10=O)N%11.O=C(O)[CH](CC1=CC=CC=C1)N%10	25.3_5
C%11(COC1=CC2=CC=C(Br)C=C2C=C1)=O.CC(C)C[CH](C%10=O)N%11.CC[CH](C)[CH](C(O)=O)N%10	25.3_6
C%11(COC1=CC2=CC=C(Br)C=C2C=C1)=O.CC(C)C[CH](C%10=O)N%11.CSCC[CH](C(O)=O)N%10	25.3_7
C%11(COC1=CC2=CC=C(Br)C=C2C=C1)=O.CC(C)C[CH](C%10=O)N%11.OC(C=C1)=CC=C1C[CH](C(O)=O)N%10	25.3_8
C%11(COC1=CC2=CC=C(Br)C=C2C=C1)=O.CC(C)C[CH](C%10=O)N%11.O=C(O)[CH](CC1=C(F)C(F)=C(F)C(F)=C1F)N%10	25.3_9
C%11(COC1=CC2=CC=C(Br)C=C2C=C1)=O.C[CH](C%10=O)N%11.O=C(O)CN%10	25.4_1
C%11(COC1=CC2=CC=C(Br)C=C2C=C1)=O.C[CH](C%10=O)N%11.CC(C)[CH](C(O)=O)N%10	25.4_2
C%11(COC1=CC2=CC=C(Br)C=C2C=C1)=O.C[CH](C%10=O)N%11.CC(C)C[CH](C(O)=O)N%10	25.4_3
C%11(COC1=CC2=CC=C(Br)C=C2C=C1)=O.C[CH](C%10=O)N%11.C[CH](C(O)=O)N%10	25.4_4
C%11(COC1=CC2=CC=C(Br)C=C2C=C1)=O.C[CH](C%10=O)N%11.O=C(O)[CH](CC1=CC=CC=C1)N%10	25.4_5
C%11(COC1=CC2=CC=C(Br)C=C2C=C1)=O.C[CH](C%10=O)N%11.CC[CH](C)[CH](C(O)=O)N%10	25.4_6
C%11(COC1=CC2=CC=C(Br)C=C2C=C1)=O.C[CH](C%10=O)N%11.CSCC[CH](C(O)=O)N%10	25.4_7
C%11(COC1=CC2=CC=C(Br)C=C2C=C1)=O.C[CH](C%10=O)N%11.OC(C=C1)=CC=C1C[CH](C(O)=O)N%10	25.4_8
C%11(COC1=CC2=CC=C(Br)C=C2C=C1)=O.C[CH](C%10=O)N%11.O=C(O)[CH](CC1=C(F)C(F)=C(F)C(F)=C1F)N%10	25.4_9
C%11(COC1=CC2=CC=C(Br)C=C2C=C1)=O.O=C%10[CH](CC1=CC=CC=C1)N%11.O=C(O)CN%10	25.5_1
C%11(COC1=CC2=CC=C(Br)C=C2C=C1)=O.O=C%10[CH](CC1=CC=CC=C1)N%11.CC(C)[CH](C(O)=O)N%10	25.5_2
C%11(COC1=CC2=CC=C(Br)C=C2C=C1)=O.O=C%10[CH](CC1=CC=CC=C1)N%11.CC(C)C[CH](C(O)=O)N%10	25.5_3
C%11(COC1=CC2=CC=C(Br)C=C2C=C1)=O.O=C%10[CH](CC1=CC=CC=C1)N%11.C[CH](C(O)=O)N%10	25.5_4
C%11(COC1=CC2=CC=C(Br)C=C2C=C1)=O.O=C%10[CH](CC1=CC=CC=C1)N%11.O=C(O)[CH](CC1=CC=CC=C1)N%10	25.5_5
C%11(COC1=CC2=CC=C(Br)C=C2C=C1)=O.O=C%10[CH](CC1=CC=CC=C1)N%11.CC[CH](C)[CH](C(O)=O)N%10	25.5_6
C%11(COC1=CC2=CC=C(Br)C=C2C=C1)=O.O=C%10[CH](CC1=CC=CC=C1)N%11.CSCC[CH](C(O)=O)N%10	25.5_7
C%11(COC1=CC2=CC=C(Br)C=C2C=C1)=O.O=C%10[CH](CC1=CC=CC=C1)N%11.OC(C=C1)=CC=C1C[CH](C(O)=O)N%10	25.5_8
C%11(COC1=CC2=CC=C(Br)C=C2C=C1)=O.O=C%10[CH](CC1=CC=CC=C1)N%11.O=C(O)[CH](CC1=C(F)C(F)=C(F)C(F)=C1F)N%10	25.5_9
C%11(COC1=CC2=CC=C(Br)C=C2C=C1)=O.CC[CH](C)[CH](C%10=O)N%11.O=C(O)CN%10	25.6_1
C%11(COC1=CC2=CC=C(Br)C=C2C=C1)=O.CC[CH](C)[CH](C%10=O)N%11.CC(C)[CH](C(O)=O)N%10	25.6_2
C%11(COC1=CC2=CC=C(Br)C=C2C=C1)=O.CC[CH](C)[CH](C%10=O)N%11.CC(C)C[CH](C(O)=O)N%10	25.6_3

C%11(COC1=CC2=CC=C(Br)C=C2C=C1)=O.CC[CH](C)[CH](C%10=O)N%11.C[CH](C(O)=O)N%10	25.6_4
C%11(COC1=CC2=CC=C(Br)C=C2C=C1)=O.CC[CH](C)[CH](C%10=O)N%11.O=C(O)[CH](CC1=CC=CC=C1)N%10	25.6_5
C%11(COC1=CC2=CC=C(Br)C=C2C=C1)=O.CC[CH](C)[CH](C%10=O)N%11.CC[CH](C)[CH](C(O)=O)N%10	25.6_6
C%11(COC1=CC2=CC=C(Br)C=C2C=C1)=O.CC[CH](C)[CH](C%10=O)N%11.CSCC[CH](C(O)=O)N%10	25.6_7
C%11(COC1=CC2=CC=C(Br)C=C2C=C1)=O.CC[CH](C)[CH](C%10=O)N%11.OC(C=C1)=CC=C1C[CH](C(O)=O)N%10	25.6_8
C%11(COC1=CC2=CC=C(Br)C=C2C=C1)=O.CC[CH](C)[CH](C%10=O)N%11.O=C(O)[CH](CC1=C(F)C(F)=C(F)C(F)=C1F)N%10	25.6_9
C%11(COC1=CC2=CC=C(Br)C=C2C=C1)=O.CSCC[CH](C%10=O)N%11.O=C(O)CN%10	25.7_1
C%11(COC1=CC2=CC=C(Br)C=C2C=C1)=O.CSCC[CH](C%10=O)N%11.CC(C)[CH](C(O)=O)N%10	25.7_2
C%11(COC1=CC2=CC=C(Br)C=C2C=C1)=O.CSCC[CH](C%10=O)N%11.CC(C)C[CH](C(O)=O)N%10	25.7_3
C%11(COC1=CC2=CC=C(Br)C=C2C=C1)=O.CSCC[CH](C%10=O)N%11.C[CH](C(O)=O)N%10	25.7_4
C%11(COC1=CC2=CC=C(Br)C=C2C=C1)=O.CSCC[CH](C%10=O)N%11.O=C(O)[CH](CC1=CC=CC=C1)N%10	25.7_5
C%11(COC1=CC2=CC=C(Br)C=C2C=C1)=O.CSCC[CH](C%10=O)N%11.CC[CH](C)[CH](C(O)=O)N%10	25.7_6
C%11(COC1=CC2=CC=C(Br)C=C2C=C1)=O.CSCC[CH](C%10=O)N%11.CSCC[CH](C(O)=O)N%10	25.7_7
C%11(COC1=CC2=CC=C(Br)C=C2C=C1)=O.CSCC[CH](C%10=O)N%11.OC(C=C1)=CC=C1C[CH](C(O)=O)N%10	25.7_8
C%11(COC1=CC2=CC=C(Br)C=C2C=C1)=O.CSCC[CH](C%10=O)N%11.O=C(O)[CH](CC1=C(F)C(F)=C(F)C(F)=C1F)N%10	25.7_9
C%11(COC1=CC2=CC=C(Br)C=C2C=C1)=O.OC(C=C1)=CC=C1C[CH](C%10=O)N%11.O=C(O)CN%10	25.8_1
C%11(COC1=CC2=CC=C(Br)C=C2C=C1)=O.OC(C=C1)=CC=C1C[CH](C%10=O)N%11.CC(C)[CH](C(O)=O)N%10	25.8_2
C%11(COC1=CC2=CC=C(Br)C=C2C=C1)=O.OC(C=C1)=CC=C1C[CH](C%10=O)N%11.CC(C)C[CH](C(O)=O)N%10	25.8_3
C%11(COC1=CC2=CC=C(Br)C=C2C=C1)=O.OC(C=C1)=CC=C1C[CH](C%10=O)N%11.C[CH](C(O)=O)N%10	25.8_4
C%11(COC1=CC2=CC=C(Br)C=C2C=C1)=O.OC(C=C1)=CC=C1C[CH](C%10=O)N%11.O=C(O)[CH](CC1=CC=CC=C1)N%10	25.8_5
C%11(COC1=CC2=CC=C(Br)C=C2C=C1)=O.OC(C=C1)=CC=C1C[CH](C%10=O)N%11.CC[CH](C)[CH](C(O)=O)N%10	25.8_6
C%11(COC1=CC2=CC=C(Br)C=C2C=C1)=O.OC(C=C1)=CC=C1C[CH](C%10=O)N%11.CSCC[CH](C(O)=O)N%10	25.8_7
C%11(COC1=CC2=CC=C(Br)C=C2C=C1)=O.OC(C=C1)=CC=C1C[CH](C%10=O)N%11.OC(C=C1)=CC=C1C[CH](C(O)=O)N%10	25.8_8
C%11(COC1=CC2=CC=C(Br)C=C2C=C1)=O.OC(C=C1)=CC=C1C[CH](C%10=O)N%11.O=C(O)[CH](CC1=C(F)C(F)=C(F)C(F)=C1F)N%10	25.8_9
C%10(COC1=CC2=CC=C(Br)C=C2C=C1)=O.O=C(O)CN%10	25.9_1
C%10(COC1=CC2=CC=C(Br)C=C2C=C1)=O.CC(C)[CH](C(O)=O)N%10	25.9_2
C%10(COC1=CC2=CC=C(Br)C=C2C=C1)=O.CC(C)C[CH](C(O)=O)N%10	25.9_3

C%10(COC1=CC2=CC=C(Br)C=C2C=C1)=O.C[CH](C(O)=O)N%10	25.9_4
C%10(COC1=CC2=CC=C(Br)C=C2C=C1)=O.O=C(O)[CH](CC1=CC=CC=C1)N%10	25.9_5
C%10(COC1=CC2=CC=C(Br)C=C2C=C1)=O.CC[CH](C)[CH](C(O)=O)N%10	25.9_6
C%10(COC1=CC2=CC=C(Br)C=C2C=C1)=O.CSCC[CH](C(O)=O)N%10	25.9_7
C%10(COC1=CC2=CC=C(Br)C=C2C=C1)=O.OC(C=C1)=CC=C1C[CH](C(O)=O)N%10	25.9_8
C%10(COC1=CC2=CC=C(Br)C=C2C=C1)=O.O=C(O)[CH](CC1=C(F)C(F)=C(F)C(F)=C1F)N%10	25.9_9

699 Compound Virtual Library Within Applicability Domain of SVM, RF and NN Models

CC(C)CC(NC(=O)COc1cccc2ccccc12)C(=O)NCC(=O)O
CC(C)CC(NC(=O)COc1ccc2ccccc2c1Br)C(=O)NCC(=O)O
CCC(C)C(NC(=O)C(NC(=O)COc1cccc2ccccc12)C(C)C)C(=O)O
CC(C)CC(NC(=O)CNC(=O)COc1cccc2ccccc12)C(=O)O
CCC(C)C(NC(=O)C(NC(=O)COc1cccc2ccccc12)C(C)CC)C(=O)O
CC(C)CC(NC(=O)COc1cccc2ccccc12)C(=O)O
CCC(C)C(NC(=O)CNC(=O)COc1cccc2ccccc12)C(=O)O
CC(C)CC(NC(=O)COc1cccc2cc(Br)ccc12)C(=O)NCC(=O)O
CCC(C)C(NC(=O)COc1cccc2ccccc12)C(=O)O
CC(C)CC(NC(=O)CNC(=O)COc1cccc2cc(Br)ccc12)C(=O)O
CC(C)CC(NC(=O)COc1ccc2ccccc2c1)C(=O)NCC(=O)O
CC(C)CC(NC(=O)CNC(=O)COc1ccc2ccccc2c1)C(=O)O
CCC(C)C(NC(=O)C(CC(C)C)NC(=O)COc1cccc2ccccc12)C(=O)O
CC(C)CC(NC(=O)C(NC(=O)COc1cccc2ccccc12)C(C)C)C(=O)O
CC(C)CC(NC(=O)COc1ccc(Cl)c2ccccc12)C(=O)NCC(=O)O
CC(C)CC(NC(=O)COc1cccc2cc(Br)ccc12)C(=O)O
CC(C)CC(NC(=O)CNC(=O)COc1ccc(Cl)c2ccccc12)C(=O)O
CCC(C)C(NC(=O)COc1cccc2cc(ccc12)C#N)C(=O)O
CC(C)CC(NC(=O)CNC(=O)COc1cc(Cl)c2ccccc2c1)C(=O)O
CC(C)CC(NC(=O)COc1cc(Cl)c2ccccc2c1)C(=O)NCC(=O)O
CC(C)C(NC(=O)C(NC(=O)COc1cccc2ccccc12)C(C)C)C(=O)O
CCC(C)C(NC(=O)CNC(=O)COc1ccc2ccccc2c1)C(=O)O
CCC(C)C(NC(=O)COc1cccc2ccccc12)C(=O)NC(C(C)C)C(=O)O
CC(C)CC(NC(=O)COc1ccc(Cl)c2ccccc12)C(=O)O
CCC(C)C(NC(=O)C(NC(=O)COc1ccc2ccccc2c1)C(C)C)C(=O)O
CCC(C)C(NC(=O)CNC(=O)COc1cccc2cc(Br)ccc12)C(=O)O
CCC(C)C(NC(=O)CNC(=O)COc1cccc2cc(ccc12)C#N)C(=O)O
CC(C)CC(NC(=O)COc1cccc2cc(ccc12)C#N)C(=O)O
CCC(C)C(NC(=O)C(NC(=O)COc1cccc2cc(ccc12)C#N)C(C)C)C(=O)O
CC(C)CC(NC(=O)CNC(=O)COc1cccc2cc(ccc12)C#N)C(=O)O
CCC(C)C(NC(=O)COc1cccc2cc(Br)ccc12)C(=O)O
CCC(C)C(NC(=O)COc1cccc2ccccc12)C(=O)NC(CC(C)C)C(=O)O
CC(C)CC(NC(=O)COc1ccc2ccccc2c1)C(=O)O
CCC(C)C(NC(=O)COc1cccc2ccccc12)C(=O)NCC(=O)O
CC(C)CC(NC(=O)COc1ccc2CCCCc2c1)C(=O)NCC(=O)O
CC(C)CC(NC(=O)COc1cccc2cc(ccc12)C#N)C(=O)NCC(=O)O
CCC(C)C(NC(=O)C(NC(=O)COc1ccc(Cl)c2ccccc12)C(C)C)C(=O)O
CC(C)CC(NC(=O)COc1cc(Cl)c2ccccc2c1)C(=O)O
CCC(C)C(NC(=O)CNC(=O)COc1ccc(Cl)c2ccccc12)C(=O)O
CC(C)CC(NC(=O)COc1cccc2ccccc12)C(=O)NC(C(C)C)C(=O)O
CCC(C)C(NC(=O)C(NC(=O)COc1cccc2cc(Br)ccc12)C(C)C)C(=O)O

CCC(C)C(NC(=O)C(NC(=O)COc1ccccc1)C(C)C)C(=O)O
CCC(C)C(NC(=O)CNC(=O)COc1cc(Cl)c2ccccc2c1)C(=O)O
CCC(C)C(NC(=O)CNC(=O)COc1ccc2CCCCc2c1)C(=O)O
CCC(C)C(NC(=O)COc1ccc(Cl)c2ccccc12)C(=O)O
CC(C)CC(NC(=O)CNC(=O)COc1ccc2CCCCc2c1)C(=O)O
CCC(C)C(NC(=O)C(NC(=O)COc1cc(Cl)c2ccccc2c1)C(C)C)C(=O)O
CCC(C)C(NC(=O)COc1ccc2ccccc2c1)C(=O)O
CC(C)CC(NC(=O)CNC(=O)COc1ccc2cc(Br)ccc2c1)C(=O)O
CCCCCCCCCCCCCC(=O)NCC(=O)NC(CC(C)C)C(=O)O
CCC(C)C(NC(=O)COc1cc(Cl)c2ccccc2c1)C(=O)O
CCC(C)C(NC(=O)C(NC(=O)COc1cccc2cc(ccc12)C#N)C(C)CC)C(=O)O
CC(C)C(NC(=O)COc1cccc2ccccc12)C(=O)NCC(=O)O
CC(C)C(NC(=O)CNC(=O)COc1cccc2ccccc12)C(=O)O
CC(C)C(NC(=O)COc1cccc2ccccc12)C(=O)O
CCC(C)C(NC(=O)C(NC(=O)COc1ccc(Cl)c2ccccc12)C(C)CC)C(=O)O
CCC(C)C(NC(=O)C(CC(C)C)NC(=O)COc1ccc2ccccc2c1)C(=O)O
CCC(C)C(NC(=O)C(NC(=O)COc1ccc2CCCCc2c1)C(C)C)C(=O)O
CCC(C)C(NC(=O)C(NC(=O)COc1ccccc1)C(C)CC)C(=O)O
CC(C)CC(NC(=O)COc1ccc2cc(Br)ccc2c1)C(=O)NCC(=O)O
CC(C)CC(NC(=O)C(CC(C)C)NC(=O)COc1cccc2ccccc12)C(=O)O
COc1cc2ccccc2cc1OCC(=O)NC(CC(C)C)C(=O)O
CCC(C)C(NC(=O)C(CC(C)C)NC(=O)COc1ccccc1)C(=O)O
COc1cc2ccccc2cc1OCC(=O)NCC(=O)NC(CC(C)C)C(=O)O
CC(C)CC(NC(=O)C(NC(=O)COc1cccc2cc(Br)ccc12)C(C)C)C(=O)O
CC(C)C(NC(=O)COc1cccc2ccccc12)C(=O)NC(C)C(=O)O
CCC(C)C(NC(=O)C(C)NC(=O)COc1cccc2ccccc12)C(=O)O
CC(C)CC(NC(=O)C(NC(=O)COc1ccc(Cl)c2ccccc12)C(C)C)C(=O)O
CC(C)CC(NC(=O)COc1cccc2ccccc12)C(=O)NC(C)C(=O)O
CCC(C)C(NC(=O)COc1cc2ccccc2cc1OC)C(=O)O
CCC(C)C(NC(=O)COc1ccc2CCCCc2c1)C(=O)O
CCC(C)C(NC(=O)COc1cccc2ccccc12)C(=O)NC(C)C(=O)O
CC(C)CC(NC(=O)COc1ccc2CCCCc2c1)C(=O)O
COc1ccc2ccc(OCC(=O)NCC(=O)NC(CC(C)C)C(=O)O)cc2c1
CCC(C)C(NC(=O)C(CC(C)C)NC(=O)COc1cccc2cc(ccc12)C#N)C(=O)O
CCC(C)C(NC(=O)C(CC(C)C)NC(=O)COc1ccc(Cl)c2ccccc12)C(=O)O
COc1cc2ccccc2cc1OCC(=O)NC(CC(C)C)C(=O)NCC(=O)O
CCC(C)C(NC(=O)C(NC(=O)COc1cc(Cl)c2ccccc2c1)C(C)CC)C(=O)O
CC(C)C(NC(=O)COc1ccc2CCCCc2c1)C(=O)NCC(=O)O
CCCCCCCCCCCCCC(=O)NCC(=O)O
CC(C)CC(NC(=O)CNC(=O)COc1ccc2cc(ccc2c1)C#N)C(=O)O
CCC(C)C(NC(=O)C(NC(=O)COc1ccc2ccccc2c1)C(C)CC)C(=O)O
CC(C)C(NC(=O)COc1cccc2cc(ccc12)C#N)C(=O)O
COc1ccc2ccc(OCC(=O)NC(CC(C)C)C(=O)O)cc2c1
COc1ccc2ccc(OCC(=O)NC(CC(C)C)C(=O)NCC(=O)O)cc2c1

CC(C)CC(NC(=O)C(NC(=O)COc1ccccc1)C(C)C)C(=O)O
CCC(C)C(NC(=O)CNC(=O)COc1cccc2CCCCc12)C(=O)O
CC(C)CC(NC(=O)C(NC(=O)COc1cccc2cc(ccc12)C#N)C(C)C)C(=O)O
CCCCCCCCCCCCCC(=O)NCC(=O)NC(C(C)CC)C(=O)O
CCC(C)C(NC(=O)CNC(=O)COc1cc2ccccc2cc1OC)C(=O)O
CCCCCCCCCCCCCC(=O)NC(CC(C)C)C(=O)NCC(=O)O
CC(C)CC(NC(=O)C(C)NC(=O)COc1cccc2ccccc12)C(=O)O
CCC(C)C(NC(=O)C(NC(=O)COc1cccc2cc(Br)ccc12)C(C)CC)C(=O)O
COc1ccc2cc(OCC(=O)NCC(=O)NC(CC(C)C)C(=O)O)ccc2c1
CCC(C)C(NC(=O)C(CC(C)C)NC(=O)COc1cc(Cl)c2ccccc2c1)C(=O)O
CCC(C)C(NC(=O)C(NC(=O)COc1ccc2CCCCc2c1)C(C)CC)C(=O)O
CC(C)C(NC(=O)C(NC(=O)COc1cccc2cc(ccc12)C#N)C(C)C)C(=O)O
CCC(C)C(NC(=O)C(CC(C)C)NC(=O)COc1cccc2cc(Br)ccc12)C(=O)O
CCC(C)C(NC(=O)C(NC(=O)COc1cc2ccccc2cc1OC)C(C)C)C(=O)O
CC(C)C(NC(=O)C(NC(=O)COc1ccc2ccccc2c1)C(C)C)C(=O)O
CCCCCCCCCCCCCCCC(=O)NCC(=O)NC(CC(C)C)C(=O)O
CC(C)CC(NC(=O)COc1cccc2CCCCc12)C(=O)NCC(=O)O
CC(C)CC(NC(=O)COc1ccc2cc(ccc2c1)C#N)C(=O)NCC(=O)O
CCCCCCCCCCCCCCCC(=O)NCC(=O)O
CC(C)CC(NC(=O)COc1ccc2cc(Br)ccc2c1)C(=O)O
COc1ccc2cc(OCC(=O)NC(CC(C)C)C(=O)O)ccc2c1
CC(C)CC(NC(=O)C(NC(=O)COc1cc(Cl)c2ccccc2c1)C(C)C)C(=O)O
COc1ccc2cc(OCC(=O)NC(CC(C)C)C(=O)NCC(=O)O)ccc2c1
CC(C)C(NC(=O)C(NC(=O)COc1cccc2cc(Br)ccc12)C(C)C)C(=O)O
CC(C)CC(NC(=O)CNC(=O)COc1cccc2CCCCc12)C(=O)O
CC(C)C(NC(=O)CNC(=O)COc1cccc2cc(Br)ccc12)C(=O)O
CCC(C)C(NC(=O)COc1cccc2cc(ccc12)C#N)C(=O)NC(C(C)C)C(=O)O
CCCCCCCCCCCCCC(=O)NCC(=O)NCC(=O)O
CCC(C)C(NC(=O)CNC(=O)COc1ccc2ccc(OC)cc2c1)C(=O)O
CC(C)CC(NC(=O)C(NC(=O)COc1ccc2ccccc2c1)C(C)C)C(=O)O
CCC(C)C(NC(=O)C(NC(=O)COc1cccc2CCCCc12)C(C)C)C(=O)O
CCC(C)C(NC(=O)COc1ccc(Cl)c2ccccc12)C(=O)NCC(=O)O
CCC(C)C(NC(=O)C(CC(C)C)NC(=O)COc1ccc2CCCCc2c1)C(=O)O
CC(C)C(NC(=O)CNC(=O)COc1ccc2ccccc2c1)C(=O)O
CCC(C)C(NC(=O)COc1cccc2CCCCc12)C(=O)O
CCC(C)C(NC(=O)COc1cccc2cc(ccc12)C#N)C(=O)NCC(=O)O
CCCCCCCCCCCCCCCC(=O)NCC(=O)NC(C(C)CC)C(=O)O
CC(C)C(NC(=O)COc1cccc2cc(Br)ccc12)C(=O)NCC(=O)O
CC(C)C(NC(=O)COc1ccc(Cl)c2ccccc12)C(=O)NCC(=O)O
CC(C)C(NC(=O)C(NC(=O)COc1ccc(Cl)c2ccccc12)C(C)C)C(=O)O
CC(C)C(NC(=O)COc1cccc2cc(Br)ccc12)C(=O)O
CC(C)CC(NC(=O)COc1ccc2cc(ccc2c1)C#N)C(=O)O
CCC(C)C(NC(=O)COc1ccc2ccc(OC)cc2c1)C(=O)O
CC(C)CC(NC(=O)COc1cccc2cc(Br)ccc12)C(=O)NC(C(C)C)C(=O)O

CC(C)C(NC(=O)C(NC(=O)COc1cc(Cl)c2ccccc2c1)C(C)C)C(=O)O
CC(C)CC(NC(=O)COc1cccc2cc(ccc12)C#N)C(=O)NC(C(C)C)C(=O)O
CCC(C)C(NC(=O)COc1ccc(Cl)c2ccccc12)C(=O)NC(C(C)C)C(=O)O
CC(C)C(NC(=O)COc1cccc2CCCCc12)C(=O)NCC(=O)O
CCCCCCCCCCCCCCCC(=O)NCC(=O)NCC(=O)O
CCC(C)C(NC(=O)COc1cccc2cc(Br)ccc12)C(=O)NCC(=O)O
CC(C)CC(NC(=O)COc1ccc2ccccc2c1)C(=O)NC(C(C)C)C(=O)O
CC(C)C(NC(=O)C(NC(=O)COc1ccc2CCCCc2c1)C(C)C)C(=O)O
CCC(C)C(NC(=O)C(NC(=O)COc1cc2ccccc2cc1OC)C(C)CC)C(=O)O
CC(C)CC(NC(=O)CNC(=O)Cn1c2ccccc2c3ccccc13)C(=O)O
CCCCCCCCCCCCCCCC(=O)NC(CC(C)C)C(=O)NCC(=O)O
CC(C)C(NC(=O)CNC(=O)COc1ccc2CCCCc2c1)C(=O)O
CC(C)CC(NC(=O)COc1ccc(Cl)c2ccccc12)C(=O)NC(C(C)C)C(=O)O
CC(C)CC(NC(=O)C(NC(=O)COc1ccc2CCCCc2c1)C(C)C)C(=O)O
CCC(C)C(NC(=O)C(CC(C)C)NC(=O)COc1cc2ccccc2cc1OC)C(=O)O
CC(C)C(NC(=O)CNC(=O)COc1cc(Cl)c2ccccc2c1)C(=O)O
CCC(C)C(NC(=O)C(NC(=O)COc1ccc2ccc(OC)cc2c1)C(C)C)C(=O)O
CC(C)C(NC(=O)C(NC(=O)COc1ccccc1)C(C)C)C(=O)O
CC(C)C(NC(=O)COc1cc(Cl)c2ccccc2c1)C(=O)NCC(=O)O
CCC(C)C(NC(=O)COc1cccc2cc(Br)ccc12)C(=O)NC(C(C)C)C(=O)O
CCC(C)C(NC(=O)COc1ccc(Cl)c2ccccc12)C(=O)NC(CC(C)C)C(=O)O
CC(C)CC(NC(=O)COc1cccc2CCCCc12)C(=O)O
CCCCCCCCCCCCCC(=O)NCC(=O)NC(C(C)C)C(=O)O
CCC(C)C(NC(=O)CNC(=O)COc1ccc2cc(ccc2c1)C#N)C(=O)O
CC(C)C(NC(=O)COc1ccc2ccccc2c1)C(=O)NCC(=O)O
CC(C)C(NC(=O)CNC(=O)COc1ccc(Cl)c2ccccc12)C(=O)O
COc1cc2ccccc2cc1OCC(=O)NC(C(C)C)C(=O)NC(CC(C)C)C(=O)O
CC(C)CC(NC(=O)COc1ccccc1)C(=O)NC(C(C)C)C(=O)O
CCC(C)C(NC(=O)C(NC(=O)COc1cccc2CCCCc12)C(C)CC)C(=O)O
CCC(C)C(NC(=O)COc1ccccc1)C(=O)NC(CC(C)C)C(=O)O
CCCCCCCCCCCCCC(=O)NC(C(C)C)C(=O)NCC(=O)O
CCC(C)C(NC(=O)COc1cc(Cl)c2ccccc2c1)C(=O)NCC(=O)O
CCC(C)C(NC(=O)COc1cccc2cc(ccc12)C#N)C(=O)NC(CC(C)C)C(=O)O
CC(C)C(NC(=O)CNC(=O)COc1cccc2CCCCc12)C(=O)O
CCC(C)C(NC(=O)C(C)NC(=O)COc1cccc2cc(ccc12)C#N)C(=O)O
CC(C)CC(NC(=O)COc1cc(Cl)c2ccccc2c1)C(=O)NC(C(C)C)C(=O)O
CC(C)CC(NC(=O)COc1cccc2cc(Br)ccc12)C(=O)NC(C)C(=O)O
CC(C)C(NC(=O)COc1ccc2ccccc2c1)C(=O)O
CCC(C)C(NC(=O)CNC(=O)COc1ccc2cc(OC)ccc2c1)C(=O)O
CC(C)C(NC(=O)COc1cccc2cc(ccc12)C#N)C(=O)NC(C)C(=O)O
CCC(C)C(NC(=O)COc1ccc2CCCCc2c1)C(=O)NCC(=O)O
CCC(C)C(NC(=O)CNC(=O)COc1ccc2cc(Br)ccc2c1)C(=O)O
CCC(C)C(NC(=O)COc1ccccc1)C(=O)NC(C(C)C)C(=O)O
CCC(C)C(NC(=O)COc1cc(Cl)c2ccccc2c1)C(=O)NC(C(C)C)C(=O)O

CC(C)C(NC(=O)C(NC(=O)COc1cccc2CCCCc12)C(C)C)C(=O)O
CC(C)C(NC(=O)COc1ccc(Cl)c2ccccc12)C(=O)O
CC(C)CC(NC(=O)C(CC(C)C)NC(=O)COc1ccccc1)C(=O)O
CC(C)C(NC(=O)COc1cc(Cl)c2ccccc2c1)C(=O)O
CCC(C)C(NC(=O)C(CC(C)C)NC(=O)COc1ccc2ccc(OC)cc2c1)C(=O)O
CC(C)CC(NC(=O)C(CC(C)C)NC(=O)COc1cccc2cc(Br)ccc12)C(=O)O
CCC(C)C(NC(=O)C(C)NC(=O)COc1cccc2cc(Br)ccc12)C(=O)O
CC(C)CC(NC(=O)COc1ccc(Cl)c2ccccc12)C(=O)NC(C)C(=O)O
CCC(C)C(NC(=O)COc1ccc2cc(OC)ccc2c1)C(=O)O
CC(NC(=O)COc1cccc2ccccc12)C(=O)O
CC(C)CC(NC(=O)COc1cccc2cc(ccc12)C#N)C(=O)NC(C)C(=O)O
CCC(C)C(NC(=O)COc1cccc2cc(Br)ccc12)C(=O)NC(CC(C)C)C(=O)O
CC(C)C(NC(=O)COc1ccc2CCCCc2c1)C(=O)O
CC(C)C(NC(=O)COc1cccc2cc(Br)ccc12)C(=O)NC(C)C(=O)O
COc1cc2ccccc2cc1OCC(=O)NCC(=O)NC(C(C)C)C(=O)O
CCCCCCCCCCCCCC(=O)NC(C(C)C)C(=O)NC(C(C)CC)C(=O)O
CC(C)CC(NC(=O)C(CC(C)C)NC(=O)COc1cccc2cc(ccc12)C#N)C(=O)O
CC(C)CC(NC(=O)C(CC(C)C)NC(=O)COc1ccc(Cl)c2ccccc12)C(=O)O
CCC(C)C(NC(=O)COc1ccc2cc(ccc2c1)C#N)C(=O)O
CCCCCCCCCCCCCCCC(=O)NCC(=O)NC(C(C)C)C(=O)O
CCC(C)C(NC(=O)C(C)NC(=O)COc1ccc(Cl)c2ccccc12)C(=O)O
CC(C)CC(NC(=O)COc1ccc2ccccc2c1)C(=O)NC(C)C(=O)O
CC(C)CC(NC(=O)C(C)NC(=O)COc1cccc2cc(Br)ccc12)C(=O)O
CCC(C)C(NC(=O)COc1cccc2CCCCc12)C(=O)NCC(=O)O
CCC(C)C(NC(=O)COc1cc(Cl)c2ccccc2c1)C(=O)NC(CC(C)C)C(=O)O
COc1cc2ccccc2cc1OCC(=O)NC(C(C)C)C(=O)NC(C(C)C)C(=O)O
CC(C)CC(NC(=O)C(CC(C)C)NC(=O)COc1ccc2ccccc2c1)C(=O)O
CCC(C)C(NC(=O)COc1cccc2cc(ccc12)C#N)C(=O)NC(C)C(=O)O
CCC(C)C(NC(=O)C(NC(=O)COc1ccc2cc(OC)ccc2c1)C(C)C)C(=O)O
CCC(C)C(NC(=O)CNC(=O)Cn1c2ccccc2c3ccccc13)C(=O)O
CC(C)C(NC(=O)C(C)NC(=O)COc1cccc2ccccc12)C(=O)O
CC(C)CC(NC(=O)COc1ccccc1)C(=O)NC(C)C(=O)O
CC(C)C(NC(=O)COc1cccc2CCCCc12)C(=O)O
COc1cc2ccccc2cc1OCC(=O)NC(C(C)C)C(=O)O
CCC(C)C(NC(=O)C(CC(C)C)NC(=O)COc1cccc2CCCCc12)C(=O)O
COc1cc2ccccc2cc1OCC(=O)NC(C(C)C)C(=O)NCC(=O)O
CCCCCCCCCCCCCCCC(=O)NC(C(C)C)C(=O)NCC(=O)O
CC(C)CC(NC(=O)C(NC(=O)COc1cccc2CCCCc12)C(C)C)C(=O)O
OC(=O)CNC(=O)COc1cccc2cc(Br)ccc12
CCC(C)C(NC(=O)COc1ccc2ccccc2c1)C(=O)NC(C(C)C)C(=O)O
CC(C)C(NC(=O)COc1ccc2CCCCc2c1)C(=O)NC(C)C(=O)O
CCC(C)C(NC(=O)C(NC(=O)COc1ccc2cc(ccc2c1)C#N)C(C)C)C(=O)O
COc1cc2ccccc2cc1OCC(=O)NC(CC(C)C)C(=O)NC(C(C)C)C(=O)O
CC(C)C(NC(=O)COc1ccc(Cl)c2ccccc12)C(=O)NC(C)C(=O)O

CC(C)CC(NC(=O)C(C)NC(=O)COc1cccc2cc(ccc12)C#N)C(=O)O
CC(C)CC(NC(=O)COc1cc(Cl)c2ccccc2c1)C(=O)NC(C)C(=O)O
CC(C)CC(NC(=O)COc1ccc2CCCCc2c1)C(=O)NC(C(C)C)C(=O)O
CCCCCCCCCCCCCCC(=O)NC(C(C)C)C(=O)NC(C(C)CC)C(=O)O
CC(C)C(NC(=O)COc1ccc2ccccc2c1)C(=O)NC(C)C(=O)O
CCC(C)C(NC(=O)COc1ccc(Cl)c2ccccc12)C(=O)NC(C)C(=O)O
CCC(C)C(NC(=O)COc1cccc2cc(Br)ccc12)C(=O)NC(C)C(=O)O
CCC(C)C(NC(=O)C(CC(C)C)NC(=O)COc1ccc2cc(OC)ccc2c1)C(=O)O
COc1ccc2ccc(OCC(=O)NC(C(C)C)C(=O)NC(CC(C)C)C(=O)O)cc2c1
CC(C)CC(NC(=O)C(C)NC(=O)COc1ccc(Cl)c2ccccc12)C(=O)O
CC(C)CC(NC(=O)C(CC(C)C)NC(=O)COc1cc(Cl)c2ccccc2c1)C(=O)O
CCC(C)C(NC(=O)C(C)NC(=O)COc1cc(Cl)c2ccccc2c1)C(=O)O
CC(C)C(NC(=O)COc1cc(Cl)c2ccccc2c1)C(=O)NC(C)C(=O)O
CCC(C)C(NC(=O)COc1ccc2ccccc2c1)C(=O)NCC(=O)O
CCC(C)C(NC(=O)C(NC(=O)COc1ccc2ccc(OC)cc2c1)C(C)CC)C(=O)O
CCC(C)C(NC(=O)COc1cc2ccccc2cc1OC)C(=O)NC(C(C)C)C(=O)O
CCC(C)C(NC(=O)COc1ccc2CCCCc2c1)C(=O)NC(C(C)C)C(=O)O
CCCCCCCCCCCCCC(=O)NC(CC(C)C)C(=O)NC(C(C)CC)C(=O)O
CCC(C)C(NC(=O)COc1cc2ccccc2cc1OC)C(=O)NCC(=O)O
CCC(C)C(NC(=O)COc1ccc2cc(Br)ccc2c1)C(=O)O
CCCCCCCCCCCCCC(=O)NC(C(C)CC)C(=O)NC(C(C)CC)C(=O)O
OC(=O)C(Cc1c(F)c(F)c(F)c(F)c1F)NC(=O)CNC(=O)COc2cccc3ccccc23
CCCCCCCCCCCCCC(=O)NC(C(C)C)C(=O)NC(CC(C)C)C(=O)O
CCC(C)C(NC(=O)C(CC(C)C)NC(=O)COc1ccc2cc(ccc2c1)C#N)C(=O)O
OC(=O)C(Cc1ccccc1)NC(=O)CNC(=O)COc2cccc3ccccc23
CCC(C)C(NC(=O)COc1cccc2CCCCc12)C(=O)NC(C(C)C)C(=O)O
CCC(C)C(NC(=O)C(C)NC(=O)COc1ccccc1)C(=O)O
CC(C)C(NC(=O)COc1cccc2CCCCc12)C(=O)NC(C)C(=O)O
OC(=O)CNC(=O)COc1cc(Cl)c2ccccc2c1
CCCCCCCCCCCCCC(=O)NC(C(C)CC)C(=O)NCC(=O)O
OC(=O)C(Cc1ccccc1)NC(=O)CNC(=O)COc2ccc3ccccc3c2
OC(=O)CNC(=O)COc1ccc(Cl)c2ccccc12
CCC(C)C(NC(=O)COc1cc2ccccc2cc1OC)C(=O)NC(CC(C)C)C(=O)O
CC(C)C(NC(=O)COc1cccc2ccccc12)C(=O)NC(Cc3ccccc3)C(=O)O
CCCCCCCCCCCCCC(=O)NC(C(C)C)C(=O)NC(C(C)C)C(=O)O
CCCCCCCCCCCCCCCC(=O)NC(CC(C)C)C(=O)NC(C(C)CC)C(=O)O
OC(=O)C(Cc1c(F)c(F)c(F)c(F)c1F)NC(=O)CNC(=O)COc2ccc3ccccc3c2
CCC(C)C(NC(=O)COc1ccc2CCCCc2c1)C(=O)NC(CC(C)C)C(=O)O
CCCCCCCCCCCCCCCC(=O)NC(C(C)CC)C(=O)NC(C(C)CC)C(=O)O
CCC(C)C(NC(=O)COc1cc(Cl)c2ccccc2c1)C(=O)NC(C)C(=O)O
CCCCCCCCCCCCCC(=O)NCC(=O)NC(C)C(=O)O
CC(C)CC(NC(=O)C(C)NC(=O)COc1cc(Cl)c2ccccc2c1)C(=O)O
COc1cc2ccccc2cc1OCC(=O)NC(CC(C)C)C(=O)NC(CC(C)C)C(=O)O
CC(C)CC(NC(=O)C(C)NC(=O)COc1ccccc1)C(=O)O

CCCCCCCCCCCCCCCC(=O)NC(C(C)C)C(=O)NC(CC(C)C)C(=O)O
COc1cc2ccccc2cc1OCC(=O)NC(C(C)C)C(=O)NC(C)C(=O)O
CCC(C)C(NC(=O)C(C)NC(=O)COc1ccc2ccccc2c1)C(=O)O
CCC(C)C(NC(=O)COc1ccccc1)C(=O)NC(C)C(=O)O
CC(NC(=O)COc1cccc2cc(ccc12)C#N)C(=O)O
CCC(C)C(NC(=O)C(NC(=O)COc1ccc2cc(Br)ccc2c1)C(C)C)C(=O)O
COc1ccc2cc(OCC(=O)NC(C(C)C)C(=O)NC(CC(C)C)C(=O)O)ccc2c1
CC(NC(=O)CNC(=O)COc1cccc2cc(Br)ccc12)C(=O)O
CC(C)CC(NC(=O)COc1cccc2CCCCc12)C(=O)NC(C(C)C)C(=O)O
CC(C)CC(NC(=O)COc1ccc2CCCCc2c1)C(=O)NC(C)C(=O)O
CCC(C)C(NC(=O)C(CC(C)C)NC(=O)COc1ccc2cc(Br)ccc2c1)C(=O)O
CCCCCCCCCCCCCCCC(=O)NC(C(C)C)C(=O)NC(C(C)C)C(=O)O
COc1ccc2ccc(OCC(=O)NCC(=O)NC(C(C)C)C(=O)O)cc2c1
CCC(C)C(NC(=O)C(C)NC(=O)COc1cccc2CCCCc12)C(=O)O
COc1cc2ccccc2cc1OCC(=O)NC(CC(C)C)C(=O)NC(C)C(=O)O
CC(C)CC(NC(=O)C(CC(C)C)NC(=O)COc1ccc2CCCCc2c1)C(=O)O
COc1ccc2ccc(OCC(=O)NC(CC(C)C)C(=O)NC(C(C)C)C(=O)O)cc2c1
CCC(C)C(NC(=O)COc1ccc2ccccc2c1)C(=O)NC(CC(C)C)C(=O)O
CC(C)C(NC(=O)COc1cccc2ccccc12)C(=O)NC(Cc3c(F)c(F)c(F)c(F)c3F)C(=O)O
CCC(C)C(NC(=O)C(Cc1ccccc1)NC(=O)COc2cccc3ccccc23)C(=O)O
CCCCCCCCCCCCCCCC(=O)NC(C(C)CC)C(=O)NCC(=O)O
CCCCCCCCCCCCCC(=O)NC(C(C)C)C(=O)NC(C)C(=O)O
CC(C)CC(NC(=O)Cn1c2ccccc2c3ccccc13)C(=O)NCC(=O)O
CCC(C)C(NC(=O)C(C)NC(=O)COc1cc2ccccc2cc1OC)C(=O)O
OC(=O)C(Cc1ccccc1)NC(=O)CNC(=O)COc2cccc3cc(ccc23)C#N
COc1ccc2ccc(OCC(=O)NC(C(C)C)C(=O)NCC(=O)O)cc2c1
OC(=O)C(Cc1c(F)c(F)c(F)c(F)c1F)NC(=O)CNC(=O)COc2ccccc2
CCCCCCCCCCCCCCCC(=O)NCC(=O)NC(C)C(=O)O
OC(=O)C(Cc1c(F)c(F)c(F)c(F)c1F)NC(=O)CNC(=O)COc2cccc3cc(ccc23)C#N
CCC(C)C(NC(=O)C(C)NC(=O)COc1ccc2CCCCc2c1)C(=O)O
CCC(C)C(NC(=O)C(NC(=O)COc1ccc2cc(OC)ccc2c1)C(C)CC)C(=O)O
COc1ccc2ccc(OCC(=O)NC(C(C)C)C(=O)NC(C(C)C)C(=O)O)cc2c1
CCCCCCCCCCCCCC(=O)NC(CC(C)C)C(=O)NC(C(C)C)C(=O)O
CCC(C)C(NC(=O)COc1cccc2CCCCc12)C(=O)NC(CC(C)C)C(=O)O
CC(C)CC(NC(=O)C(NC(=O)COc1ccc2cc(ccc2c1)C#N)C(C)C)C(=O)O
CC(C)CC(NC(=O)C(NC(=O)COc1ccc2cc(Br)ccc2c1)C(C)C)C(=O)O
OC(=O)C(Cc1c(F)c(F)c(F)c(F)c1F)NC(=O)CNC(=O)COc2ccc3CCCCc3c2
CCCCCCCCCCCCCC(=O)NC(C(C)CC)C(=O)NC(CC(C)C)C(=O)O
CC(C)CC(NC(=O)C(C)NC(=O)COc1ccc2ccccc2c1)C(=O)O
OC(=O)C(Cc1ccccc1)NC(=O)CNC(=O)COc2cccc3cc(Br)ccc23
CCC(C)C(NC(=O)C(NC(=O)COc1ccc2cc(ccc2c1)C#N)C(C)CC)C(=O)O
COc1ccc2ccc(OCC(=O)NC(CC(C)C)C(=O)NC(CC(C)C)C(=O)O)cc2c1
CCCCCCCCCCCCCCCC(=O)NC(C(C)C)C(=O)NC(C)C(=O)O
CCC(C)C(NC(=O)COc1cccc2ccccc12)C(=O)NC(Cc3ccccc3)C(=O)O

CCCCCCCCCCCCCC(=O)NC(CC(C)C)C(=O)NC(C)C(=O)O
CCCCCCCCCCCCCCCC(=O)NC(CC(C)C)C(=O)NC(C(C)C)C(=O)O
CCCCCCCCCCCCCC(=O)NC(CC(C)C)C(=O)NC(CC(C)C)C(=O)O
CCC(C)C(NC(=O)COc1ccc2CCCCc2c1)C(=O)NC(C)C(=O)O
OC(=O)C(Cc1ccccc1)NC(=O)CNC(=O)COc2ccc3CCCCc3c2
CC(C)C(NC(=O)C(C)NC(=O)COc1cccc2cc(ccc12)C#N)C(=O)O
CC(C)CC(NC(=O)COc1cccc2ccccc12)C(=O)NC(Cc3ccccc3)C(=O)O
CCC(C)C(NC(=O)COc1cc2ccccc2cc1OC)C(=O)NC(C)C(=O)O
COc1ccc2ccc(OCC(=O)NC(C(C)C)C(=O)O)cc2c1
COc1cc2ccccc2cc1OCC(=O)NC(C)C(=O)NC(CC(C)C)C(=O)O
CC(NC(=O)COc1cccc2cc(Br)ccc12)C(=O)O
CC(C)CC(NC(=O)COc1cccc2CCCCc12)C(=O)NC(C)C(=O)O
CCCCCCCCCCCCCC(=O)NC(C(C)CC)C(=O)NC(C(C)C)C(=O)O
OC(=O)C(Cc1c(F)c(F)c(F)c(F)c1F)NC(=O)CNC(=O)COc2cccc3cc(Br)ccc23
CC(C)CC(NC(=O)COc1cccc2ccccc12)C(=O)NC(Cc3c(F)c(F)c(F)c(F)c3F)C(=O)O
COc1ccc2cc(OCC(=O)NCC(=O)NC(C(C)C)C(=O)O)ccc2c1
CCC(C)C(NC(=O)COc1ccc2ccccc2c1)C(=O)NC(C)C(=O)O
CCC(C)C(NC(=O)COc1cccc2CCCCc12)C(=O)NC(C)C(=O)O
CCCCCCCCCCCCCCCC(=O)NC(C(C)CC)C(=O)NC(CC(C)C)C(=O)O
CC(C)C(NC(=O)COc1ccccc1)C(=O)NC(Cc2c(F)c(F)c(F)c(F)c2F)C(=O)O
COc1ccc2cc(OCC(=O)NC(CC(C)C)C(=O)NC(C(C)C)C(=O)O)ccc2c1
CCC(C)C(NC(=O)COc1ccc2ccc(OC)cc2c1)C(=O)NCC(=O)O
COc1ccc2ccc(OCC(=O)NC(CC(C)C)C(=O)NC(C)C(=O)O)cc2c1
CC(C)CC(NC(=O)Cn1c2ccccc2c3ccccc13)C(=O)O
CC(C)CC(NC(=O)C(C)NC(=O)COc1ccc2CCCCc2c1)C(=O)O
CC(C)CC(NC(=O)C(C)NC(=O)COc1cccc2CCCCc12)C(=O)O
CCCCCCCCCCCCCC(=O)NCC(=O)NC(Cc1c(F)c(F)c(F)c(F)c1F)C(=O)O
CCCCCCCCCCCCCCCC(=O)NC(CC(C)C)C(=O)NC(CC(C)C)C(=O)O
OC(=O)C(Cc1ccccc1)NC(=O)CNC(=O)COc2cc(Cl)c3ccccc3c2
CCCCCCCCCCCCCCCC(=O)NC(CC(C)C)C(=O)NC(C)C(=O)O
CCC(C)C(NC(=O)COc1cccc2ccccc12)C(=O)NC(Cc3c(F)c(F)c(F)c(F)c3F)C(=O)O
CCCCCCCCCCCCCCCC(=O)NC(C(C)CC)C(=O)NC(C(C)C)C(=O)O
CCC(C)C(NC(=O)COc1ccc2ccc(OC)cc2c1)C(=O)NC(CC(C)C)C(=O)O
COc1ccc2cc(OCC(=O)NC(C(C)C)C(=O)NCC(=O)O)ccc2c1
OC(=O)C(Cc1ccccc1)NC(=O)CNC(=O)COc2ccc(Cl)c3ccccc23
COc1cc2ccccc2cc1OCC(=O)NCC(=O)NC(Cc3c(F)c(F)c(F)c(F)c3F)C(=O)O
CCC(C)C(NC(=O)C(Cc1ccccc1)NC(=O)COc2ccccc2)C(=O)O
CC(C)C(NC(=O)COc1ccccc1)C(=O)NC(Cc2ccccc2)C(=O)O
CCC(C)C(NC(=O)COc1ccc2ccc(OC)cc2c1)C(=O)NC(C(C)C)C(=O)O
CCCCCCCCCCCCCC(=O)NC(C)C(=O)NC(C(C)CC)C(=O)O
COc1ccc2cc(OCC(=O)NC(CC(C)C)C(=O)NC(CC(C)C)C(=O)O)ccc2c1
CC(C)CC(NC(=O)COc1ccccc1)C(=O)NC(Cc2c(F)c(F)c(F)c(F)c2F)C(=O)O
COc1cc2ccccc2cc1OCC(=O)NCC(=O)NC(Cc3ccccc3)C(=O)O
OC(=O)C(Cc1c(F)c(F)c(F)c(F)c1F)NC(=O)CNC(=O)COc2cc(Cl)c3ccccc3c2

CC(C)CC(NC(=O)C(CC(C)C)NC(=O)COc1cccc2CCCCc12)C(=O)O
CC(C)C(NC(=O)COc1cccc2cc(ccc12)C#N)C(=O)NC(Cc3ccccc3)C(=O)O
CC(C)CC(NC(=O)COc1ccc2cc(ccc2c1)C#N)C(=O)NC(C(C)C)C(=O)O
OC(=O)C(Cc1c(F)c(F)c(F)c(F)c1F)NC(=O)CNC(=O)COc2ccc(Cl)c3ccccc23
CCCCCCCCCCCCCC(=O)NC(C)C(=O)NC(CC(C)C)C(=O)O
CCC(C)C(NC(=O)C(Cc1ccccc1)NC(=O)COc2cccc3cc(ccc23)C#N)C(=O)O
CCCCCCCCCCCCCCCC(=O)NCC(=O)NC(Cc1c(F)c(F)c(F)c(F)c1F)C(=O)O
CC(C)C(NC(=O)COc1cccc2cc(ccc12)C#N)C(=O)NC(Cc3c(F)c(F)c(F)c(F)c3F)C(=O)O
COc1ccc2ccc(OCC(=O)NC(C(C)C)C(=O)NC(C)C(=O)O)cc2c1
CCCCCCCCCCCCCC(=O)NC(C(C)CC)C(=O)NC(C)C(=O)O
COc1ccc2cc(OCC(=O)NC(C(C)C)C(=O)NC(C(C)C)C(=O)O)ccc2c1
CC(C)C(NC(=O)CNC(=O)COc1ccc2cc(Br)ccc2c1)C(=O)O
CC(NC(=O)COc1ccc(Cl)c2ccccc12)C(=O)O
CC(C)C(NC(=O)C(NC(=O)COc1ccc2cc(ccc2c1)C#N)C(C)C)C(=O)O
CC(C)C(NC(=O)C(C)NC(=O)COc1cccc2cc(Br)ccc12)C(=O)O
CCC(C)C(NC(=O)C(NC(=O)COc1ccc2cc(Br)ccc2c1)C(C)CC)C(=O)O
OC(=O)C(Cc1ccccc1)NC(=O)CNC(=O)COc2cccc3CCCCc23
OC(=O)CNC(=O)COc1ccc2cc(Br)ccc2c1
OC(=O)C(Cc1c(F)c(F)c(F)c(F)c1F)NC(=O)CNC(=O)COc2cccc3CCCCc23
CC(C)CC(NC(=O)COc1ccc2cc(Br)ccc2c1)C(=O)NC(C(C)C)C(=O)O
COc1ccc2ccc(OCC(=O)NCC(=O)NC(Cc3ccccc3)C(=O)O)cc2c1
CCCCCCCCCCCCCCCC(=O)NC(C)C(=O)NC(C(C)CC)C(=O)O
COc1ccc2ccc(OCC(=O)NCC(=O)NC(Cc3c(F)c(F)c(F)c(F)c3F)C(=O)O)cc2c1
COc1ccc2cc(OCC(=O)NC(CC(C)C)C(=O)NC(C)C(=O)O)ccc2c1
CC(NC(=O)COc1ccc2CCCCc2c1)C(=O)O
OC(=O)C(Cc1c(F)c(F)c(F)c(F)c1F)NC(=O)COc2cccc3ccccc23
CC(C)C(NC(=O)C(Cc1ccccc1)NC(=O)COc2cccc3ccccc23)C(=O)O
CC(C)CC(NC(=O)COc1ccccc1)C(=O)NC(Cc2ccccc2)C(=O)O
CCC(C)C(NC(=O)C(NC(=O)Cn1c2ccccc2c3ccccc13)C(C)C)C(=O)O
CC(C)C(NC(=O)C(C)NC(=O)COc1cccc2CCCCc12)C(=O)O
CC(C)CC(NC(=O)C(CC(C)C)NC(=O)COc1ccc2cc(ccc2c1)C#N)C(=O)O
CC(NC(=O)COc1ccc2ccccc2c1)C(=O)O
CC(C)CC(NC(=O)C(CC(C)C)NC(=O)COc1ccc2cc(Br)ccc2c1)C(=O)O
CC(C)C(NC(=O)COc1ccc2CCCCc2c1)C(=O)NC(Cc3c(F)c(F)c(F)c(F)c3F)C(=O)O
COc1ccc2cc(OCC(=O)NC(C(C)C)C(=O)O)ccc2c1
OC(=O)C(Cc1ccccc1)NC(=O)COc2cccc3ccccc23
CC(NC(=O)COc1cc(Cl)c2ccccc2c1)C(=O)O
CC(C)CC(NC(=O)COc1ccc2cc(ccc2c1)C#N)C(=O)NC(C)C(=O)O
CC(C)C(NC(=O)C(C)NC(=O)COc1ccc(Cl)c2ccccc12)C(=O)O
CCCCCCCCCCCCCC(=O)NCC(=O)NC(Cc1ccccc1)C(=O)O
CC(C)CC(NC(=O)C(Cc1ccccc1)NC(=O)COc2cccc3ccccc23)C(=O)O
CC(C)C(NC(=O)COc1ccc2cc(ccc2c1)C#N)C(=O)O
CCC(C)C(NC(=O)Cn1c2ccccc2c3ccccc13)C(=O)O
CC(C)CC(NC(=O)COc1ccc2cc(Br)ccc2c1)C(=O)NC(C)C(=O)O

COc1cc2ccccc2cc1OCC(=O)NC(C)C(=O)O
CCCCCCCCCCCCCCCC(=O)NC(C)C(=O)NC(CC(C)C)C(=O)O
CC(C)CC(NC(=O)COc1cccc2cc(ccc12)C#N)C(=O)NC(Cc3ccccc3)C(=O)O
CCCCCCCCCCCCCCCC(=O)NC(C(C)CC)C(=O)NC(C)C(=O)O
CCC(C)C(NC(=O)C(CC(C)C)NC(=O)Cn1c2ccccc2c3ccccc13)C(=O)O
CCC(C)C(NC(=O)COc1ccc2cc(OC)ccc2c1)C(=O)NC(CC(C)C)C(=O)O
CCC(C)C(NC(=O)COc1ccc2cc(OC)ccc2c1)C(=O)NCC(=O)O
OC(=O)C(Cc1ccccc1)NC(=O)CNC(=O)COc2ccc3cc(ccc3c2)C#N
CC(C)C(NC(=O)COc1ccc(Cl)c2ccccc12)C(=O)NC(Cc3c(F)c(F)c(F)c(F)c3F)C(=O)O
CC(C)C(NC(=O)COc1ccc2cc(Br)ccc2c1)C(=O)NCC(=O)O
OC(=O)CNC(=O)C(Cc1ccccc1)NC(=O)COc2cccc3ccccc23
CCC(C)C(NC(=O)C(C)NC(=O)COc1ccc2ccc(OC)cc2c1)C(=O)O
CC(C)C(NC(=O)COc1ccc2CCCCc2c1)C(=O)NC(Cc3ccccc3)C(=O)O
CC(C)C(NC(=O)COc1ccc(Cl)c2ccccc12)C(=O)NC(Cc3ccccc3)C(=O)O
CCCCCCCCCCCCCC(=O)NC(C)C(=O)NCC(=O)O
OC(=O)C(Cc1c(F)c(F)c(F)c(F)c1F)NC(=O)CNC(=O)COc2ccc3cc(ccc3c2)C#N
CC(C)C(NC(=O)COc1cccc2cc(Br)ccc12)C(=O)NC(Cc3ccccc3)C(=O)O
CCCCCCCCCCCCCCCC(=O)NCC(=O)NC(Cc1ccccc1)C(=O)O
COc1ccc2cc(OCC(=O)NCC(=O)NC(Cc3ccccc3)C(=O)O)ccc2c1
CC(C)C(NC(=O)C(C)NC(=O)COc1cc(Cl)c2ccccc2c1)C(=O)O
CCC(C)C(NC(=O)C(Cc1ccccc1)NC(=O)COc2cccc3cc(Br)ccc23)C(=O)O
CCC(C)C(NC(=O)COc1ccc2cc(ccc2c1)C#N)C(=O)NCC(=O)O
CC(C)C(NC(=O)COc1cccc2CCCCc12)C(=O)NC(Cc3ccccc3)C(=O)O
CC(NC(=O)COc1cccc2ccccc12)C(=O)NC(Cc3ccccc3)C(=O)O
CC(NC(=O)COc1cccc2ccccc12)C(=O)NC(Cc3c(F)c(F)c(F)c(F)c3F)C(=O)O
CC(C)C(NC(=O)C(NC(=O)COc1ccc2cc(Br)ccc2c1)C(C)C)C(=O)O
COc1ccc2cc(OCC(=O)NCC(=O)NC(Cc3c(F)c(F)c(F)c(F)c3F)C(=O)O)ccc2c1
CC(C)CC(NC(=O)C(NC(=O)Cn1c2ccccc2c3ccccc13)C(C)C)C(=O)O
CCC(C)C(NC(=O)COc1ccc2cc(OC)ccc2c1)C(=O)NC(C(C)C)C(=O)O
CCC(C)C(NC(=O)C(Cc1ccccc1)NC(=O)COc2ccc(Cl)c3ccccc23)C(=O)O
CC(C)C(NC(=O)COc1cccc2CCCCc12)C(=O)NC(Cc3c(F)c(F)c(F)c(F)c3F)C(=O)O
CCC(C)C(NC(=O)C(Cc1ccccc1)NC(=O)COc2cccc3CCCCc23)C(=O)O
CCC(C)C(NC(=O)COc1ccccc1)C(=O)NC(Cc2c(F)c(F)c(F)c(F)c2F)C(=O)O
COc1ccc2ccc(OCC(=O)NC(C)C(=O)NC(CC(C)C)C(=O)O)cc2c1
OC(=O)C(Cc1c(F)c(F)c(F)c(F)c1F)NC(=O)COc2cccc3cc(ccc23)C#N
COc1cc2ccccc2cc1OCC(=O)NC(C)C(=O)NC(C(C)C)C(=O)O
COc1ccc2cc(OCC(=O)NC(C(C)C)C(=O)NC(C)C(=O)O)ccc2c1
CCC(C)C(NC(=O)COc1ccc2ccc(OC)cc2c1)C(=O)NC(C)C(=O)O
OC(=O)C(Cc1ccccc1)NC(=O)COc2cccc3cc(ccc23)C#N
CCCCCCCCCCCCCCCC(=O)NC(C)C(=O)NCC(=O)O
CCC(C)C(NC(=O)C(Cc1ccccc1)NC(=O)COc2ccc3CCCCc3c2)C(=O)O
OC(=O)C(Cc1ccccc1)NC(=O)CNC(=O)COc2ccc3cc(Br)ccc3c2
CCC(C)C(NC(=O)COc1ccc2cc(ccc2c1)C#N)C(=O)NC(CC(C)C)C(=O)O
CC(C)C(NC(=O)COc1cc(Cl)c2ccccc2c1)C(=O)NC(Cc3c(F)c(F)c(F)c(F)c3F)C(=O)O

CCC(C)C(NC(=O)COc1cccc2cc(ccc12)C#N)C(=O)NC(Cc3ccccc3)C(=O)O
CC(C)C(NC(=O)C(C)NC(=O)COc1ccc2CCCCc2c1)C(=O)O
OC(=O)C(Cc1c(F)c(F)c(F)c(F)c1F)NC(=O)COc2ccccc2
CC(C)C(NC(=O)COc1ccc2cc(Br)ccc2c1)C(=O)O
OC(=O)C(Cc1c(F)c(F)c(F)c(F)c1F)NC(=O)COc2ccc3CCCCc3c2
CCC(C)C(NC(=O)COc1ccccc1)C(=O)NC(Cc2ccccc2)C(=O)O
CCCCCCCCCCCCCC(=O)NC(C)C(=O)NC(C(C)C)C(=O)O
CC(C)C(NC(=O)COc1ccc2cc(ccc2c1)C#N)C(=O)NC(C)C(=O)O
CC(C)CC(NC(=O)COc1cccc2cc(Br)ccc12)C(=O)NC(Cc3ccccc3)C(=O)O
CCC(C)C(NC(=O)COc1ccc2cc(ccc2c1)C#N)C(=O)NC(C(C)C)C(=O)O
CC(C)CC(NC(=O)COc1ccc2CCCCc2c1)C(=O)NC(Cc3c(F)c(F)c(F)c(F)c3F)C(=O)O
CC(C)CC(NC(=O)C(Cc1ccccc1)NC(=O)COc2ccccc2)C(=O)O
CC(C)C(NC(=O)COc1cc(Cl)c2ccccc2c1)C(=O)NC(Cc3ccccc3)C(=O)O
CC(C)C(NC(=O)C(C)NC(=O)COc1ccc2ccccc2c1)C(=O)O
CCC(C)C(NC(=O)COc1ccc2cc(Br)ccc2c1)C(=O)NCC(=O)O
OC(=O)CNC(=O)C(Cc1ccccc1)NC(=O)COc2cccc3cc(ccc23)C#N
OC(=O)C(Cc1c(F)c(F)c(F)c(F)c1F)NC(=O)CNC(=O)COc2ccc3cc(Br)ccc3c2
CCCCCCCCCCCCCC(=O)NC(Cc1c(F)c(F)c(F)c(F)c1F)C(=O)O
COc1cc2ccccc2cc1OCC(=O)NC(C(C)C)C(=O)NC(Cc3ccccc3)C(=O)O
CC(C)CC(NC(=O)COc1ccc(Cl)c2ccccc12)C(=O)NC(Cc3ccccc3)C(=O)O
OC(=O)C(Cc1ccccc1)NC(=O)COc2ccccc2
OC(=O)CNC(=O)C(Cc1ccccc1)NC(=O)COc2ccc3CCCCc3c2
CCC(C)C(NC(=O)C(Cc1ccccc1)NC(=O)COc2ccc3ccccc3c2)C(=O)O
CCC(C)C(NC(=O)C(NC(=O)Cn1c2ccccc2c3ccccc13)C(C)CC)C(=O)O
CCC(C)C(NC(=O)C(Cc1ccccc1)NC(=O)COc2cc(Cl)c3ccccc3c2)C(=O)O
CC(NC(=O)COc1cccc2cc(Br)ccc12)C(=O)NCC(=O)O
OC(=O)C(Cc1c(F)c(F)c(F)c(F)c1F)NC(=O)COc2ccc3ccccc3c2
CC(C)C(NC(=O)COc1ccc2ccccc2c1)C(=O)NC(Cc3ccccc3)C(=O)O
CCC(C)C(NC(=O)C(Cc1ccccc1)NC(=O)COc2cc3ccccc3cc2OC)C(=O)O
CC(C)CC(NC(=O)C(Cc1ccccc1)NC(=O)COc2cccc3cc(ccc23)C#N)C(=O)O
COc1cc2ccccc2cc1OCC(=O)NC(C(C)C)C(=O)NC(Cc3c(F)c(F)c(F)c(F)c3F)C(=O)O
OC(=O)C(Cc1ccccc1)NC(=O)COc2ccc3ccccc3c2
CC(C)C(NC(=O)C(Cc1ccccc1)NC(=O)COc2cccc3cc(ccc23)C#N)C(=O)O
CCCCCCCCCCCCCCCC(=O)NC(C)C(=O)NC(C(C)C)C(=O)O
CCC(C)C(NC(=O)COc1ccc2cc(Br)ccc2c1)C(=O)NC(CC(C)C)C(=O)O
COc1ccc2cc(OCC(=O)NC(C)C(=O)NC(CC(C)C)C(=O)O)ccc2c1
CC(C)CC(NC(=O)C(C)NC(=O)COc1ccc2cc(ccc2c1)C#N)C(=O)O
OC(=O)C(Cc1c(F)c(F)c(F)c(F)c1F)NC(=O)COc2cccc3cc(Br)ccc23
CC(C)C(NC(=O)COc1ccc2ccccc2c1)C(=O)NC(Cc3c(F)c(F)c(F)c(F)c3F)C(=O)O
OC(=O)CNC(=O)C(Cc1ccccc1)NC(=O)COc2cccc3CCCCc23
OC(=O)C(Cc1ccccc1)NC(=O)COc2cccc3cc(Br)ccc23
CCCCCCCCCCCCCC(=O)NC(C(C)C)C(=O)NC(Cc1c(F)c(F)c(F)c(F)c1F)C(=O)O
OC(=O)CNC(=O)C(Cc1ccccc1)NC(=O)COc2ccc3ccccc3c2
CC(C)CC(NC(=O)COc1ccc2CCCCc2c1)C(=O)NC(Cc3ccccc3)C(=O)O

COc1cc2ccccc2cc1OCC(=O)NC(Cc3c(F)c(F)c(F)c(F)c3F)C(=O)O
OC(=O)C(Cc1ccccc1)NC(=O)COc2ccc3CCCCc3c2
CC(C)CC(NC(=O)C(C)NC(=O)COc1ccc2cc(Br)ccc2c1)C(=O)O
COc1cc2ccccc2cc1OCC(=O)NC(Cc3ccccc3)C(=O)O
CCCCCCCCCCCCCCCC(=O)NC(Cc1c(F)c(F)c(F)c(F)c1F)C(=O)O
CCC(C)C(NC(=O)C(C)NC(=O)COc1ccc2cc(OC)ccc2c1)C(=O)O
OC(=O)CNC(=O)C(Cc1ccccc1)NC(=O)COc2cccc3cc(Br)ccc23
CCC(C)C(NC(=O)C(C)NC(=O)COc1ccc2cc(ccc2c1)C#N)C(=O)O
CC(C)CC(NC(=O)COc1ccc2ccccc2c1)C(=O)NC(Cc3c(F)c(F)c(F)c(F)c3F)C(=O)O
CC(C)C(NC(=O)C(Cc1ccccc1)NC(=O)COc2ccccc2)C(=O)O
CC(NC(=O)COc1cccc2cc(ccc12)C#N)C(=O)NC(Cc3ccccc3)C(=O)O
CC(C)CC(NC(=O)COc1ccc2ccccc2c1)C(=O)NC(Cc3ccccc3)C(=O)O
COc1cc2ccccc2cc1OCC(=O)NC(CC(C)C)C(=O)NC(Cc3ccccc3)C(=O)O
OC(=O)C(Cc1c(F)c(F)c(F)c(F)c1F)NC(=O)COc2ccc(Cl)c3ccccc23
CC(C)CC(NC(=O)COc1cc(Cl)c2ccccc2c1)C(=O)NC(Cc3ccccc3)C(=O)O
CCC(C)C(NC(=O)COc1ccc(Cl)c2ccccc12)C(=O)NC(Cc3ccccc3)C(=O)O
OC(=O)C(Cc1c(F)c(F)c(F)c(F)c1F)NC(=O)CNC(=O)Cn2c3ccccc3c4ccccc24
CCC(C)C(NC(=O)COc1ccc2cc(OC)ccc2c1)C(=O)NC(C)C(=O)O
OC(=O)C(Cc1ccccc1)NC(=O)COc2ccc(Cl)c3ccccc23
CC(NC(=O)COc1cccc2cc(Br)ccc12)C(=O)NC(Cc3ccccc3)C(=O)O
CC(C)CC(NC(=O)COc1cccc2CCCCc12)C(=O)NC(Cc3ccccc3)C(=O)O
CC(NC(=O)COc1cccc2cc(ccc12)C#N)C(=O)NC(Cc3c(F)c(F)c(F)c(F)c3F)C(=O)O
OC(=O)C(Cc1ccccc1)NC(=O)CNC(=O)Cn2c3ccccc3c4ccccc24
CC(C)CC(NC(=O)Cn1c2ccccc2c3ccccc13)C(=O)NC(C(C)C)C(=O)O
CC(C)C(NC(=O)C(Cc1ccccc1)NC(=O)COc2cccc3CCCCc23)C(=O)O
OC(=O)CNC(=O)C(Cc1ccccc1)NC(=O)COc2ccc(Cl)c3ccccc23
CC(C)CC(NC(=O)COc1cccc2CCCCc12)C(=O)NC(Cc3c(F)c(F)c(F)c(F)c3F)C(=O)O
CC(C)C(NC(=O)COc1ccc2cc(Br)ccc2c1)C(=O)NC(C)C(=O)O
COc1cc2ccccc2cc1OCC(=O)NC(Cc3ccccc3)C(=O)NCC(=O)O
COc1ccc2ccc(OCC(=O)NC(C)C(=O)O)cc2c1
CCC(C)C(NC(=O)COc1ccc2cc(Br)ccc2c1)C(=O)NC(C(C)C)C(=O)O
CC(C)CC(NC(=O)C(Cc1ccccc1)NC(=O)COc2cccc3cc(Br)ccc23)C(=O)O
CC(NC(=O)C(C)NC(=O)COc1cccc2cc(Br)ccc12)C(=O)O
CCCCCCCCCCCCCC(=O)NC(Cc1ccccc1)C(=O)NCC(=O)O
OC(=O)C(Cc1c(F)c(F)c(F)c(F)c1F)NC(=O)COc2cc(Cl)c3ccccc3c2
CCC(C)C(NC(=O)Cn1c2ccccc2c3ccccc13)C(=O)NCC(=O)O
OC(=O)C(Cc1ccccc1)NC(=O)COc2cccc3CCCCc23
CCC(C)C(NC(=O)COc1cccc2cc(Br)ccc12)C(=O)NC(Cc3ccccc3)C(=O)O
CCCCCCCCCCCCCC(=O)NC(Cc1ccccc1)C(=O)O
OC(=O)C(Cc1ccccc1)NC(=O)COc2cc(Cl)c3ccccc3c2
CC(C)C(NC(=O)C(Cc1ccccc1)NC(=O)COc2ccc3CCCCc3c2)C(=O)O
OC(=O)CNC(=O)C(Cc1ccccc1)NC(=O)COc2cc(Cl)c3ccccc3c2
CCC(C)C(NC(=O)COc1ccc2cc(ccc2c1)C#N)C(=O)NC(C)C(=O)O
CCC(C)C(NC(=O)Cn1c2ccccc2c3ccccc13)C(=O)NC(CC(C)C)C(=O)O

OC(=O)C(Cc1c(F)c(F)c(F)c(F)c1F)NC(=O)COc2cccc3CCCCc23
CC(C)CC(NC(=O)C(CC(C)C)NC(=O)Cn1c2ccccc2c3ccccc13)C(=O)O
CC(C)CC(NC(=O)C(Cc1ccccc1)NC(=O)COc2ccc(Cl)c3ccccc23)C(=O)O
CCCCCCCCCCCCC(=O)NC(C)C(=O)NC(C)C(=O)O
CCC(C)C(NC(=O)COc1ccc2CCCCc2c1)C(=O)NC(Cc3c(F)c(F)c(F)c(F)c3F)C(=O)O
CCCCCCCCCCCCC(=O)NC(C(C)C)C(=O)NC(Cc1ccccc1)C(=O)O
CCC(C)C(NC(=O)COc1cccc2CCCCc12)C(=O)NC(Cc3ccccc3)C(=O)O
COc1ccc2ccc(OCC(=O)NC(Cc3c(F)c(F)c(F)c(F)c3F)C(=O)O)cc2c1
COc1ccc2ccc(OCC(=O)NC(Cc3ccccc3)C(=O)O)cc2c1
CC(C)C(NC(=O)C(Cc1ccccc1)NC(=O)COc2cccc3cc(Br)ccc23)C(=O)O
CC(NC(=O)C(C)NC(=O)COc1cccc2CCCCc12)C(=O)O
CC(NC(=O)C(Cc1ccccc1)NC(=O)COc2cccc3ccccc23)C(=O)O
CC(C)CC(NC(=O)C(Cc1ccccc1)NC(=O)COc2cccc3CCCCc23)C(=O)O
CC(NC(=O)CNC(=O)COc1ccc2cc(Br)ccc2c1)C(=O)O
CCC(C)C(NC(=O)C(Cc1ccccc1)NC(=O)COc2ccc3ccc(OC)cc3c2)C(=O)O
CC(C)C(NC(=O)C(Cc1ccccc1)NC(=O)COc2ccc(Cl)c3ccccc23)C(=O)O
CC(NC(=O)COc1ccc(Cl)c2ccccc12)C(=O)NC(Cc3ccccc3)C(=O)O
CCC(C)C(NC(=O)C(C)NC(=O)COc1ccc2cc(Br)ccc2c1)C(=O)O
CCCCCCCCCCCCCCC(=O)NC(Cc1ccccc1)C(=O)NCC(=O)O
CC(C)CC(NC(=O)C(Cc1ccccc1)NC(=O)COc2ccc3CCCCc3c2)C(=O)O
CCC(C)C(NC(=O)COc1cccc2CCCCc12)C(=O)NC(Cc3c(F)c(F)c(F)c(F)c3F)C(=O)O
CC(NC(=O)COc1ccccc1)C(=O)NC(Cc2ccccc2)C(=O)O
COc1ccc2ccc(OCC(=O)NC(C(C)C)C(=O)NC(Cc3ccccc3)C(=O)O)cc2c1
COc1ccc2ccc(OCC(=O)NC(Cc3ccccc3)C(=O)NCC(=O)O)cc2c1
CCCCCCCCCCCCCCC(=O)NC(Cc1ccccc1)C(=O)O
CCCCCCCCCCCCC(=O)NC(Cc1ccccc1)C(=O)NC(C(C)CC)C(=O)O
CC(C)C(NC(=O)C(NC(=O)Cn1c2ccccc2c3ccccc13)C(C)C)C(=O)O
CC(NC(=O)C(C)NC(=O)COc1ccc(Cl)c2ccccc12)C(=O)O
CC(NC(=O)COc1cccc2CCCCc12)C(=O)NC(Cc3ccccc3)C(=O)O
CC(NC(=O)COc1ccccc1)C(=O)NC(Cc2c(F)c(F)c(F)c(F)c2F)C(=O)O
COc1cc2ccccc2cc1OCC(=O)NC(Cc3ccccc3)C(=O)NC(CC(C)C)C(=O)O
CC(C)C(NC(=O)C(Cc1ccccc1)NC(=O)COc2ccc3ccccc3c2)C(=O)O
CCCCCCCCCCCCCCC(=O)NC(C(C)C)C(=O)NC(Cc1ccccc1)C(=O)O
CCCCCCCCCCCCCCC(=O)NC(C)C(=O)NC(C)C(=O)O
CC(NC(=O)COc1cc(Cl)c2ccccc2c1)C(=O)NC(Cc3ccccc3)C(=O)O
CC(C)CC(NC(=O)C(Cc1ccccc1)NC(=O)COc2ccc3ccccc3c2)C(=O)O
CCC(C)C(NC(=O)COc1cc(Cl)c2ccccc2c1)C(=O)NC(Cc3ccccc3)C(=O)O
COc1ccc2ccc(OCC(=O)NC(C(C)C)C(=O)NC(Cc3c(F)c(F)c(F)c(F)c3F)C(=O)O)cc2c1
CCC(C)C(NC(=O)COc1ccc2cc(Br)ccc2c1)C(=O)NC(C)C(=O)O
CC(NC(=O)COc1ccc2CCCCc2c1)C(=O)NC(Cc3ccccc3)C(=O)O
CC(C)CC(NC(=O)Cn1c2ccccc2c3ccccc13)C(=O)NC(C)C(=O)O
CC(NC(=O)C(C)NC(=O)COc1cc(Cl)c2ccccc2c1)C(=O)O
COc1cc2ccccc2cc1OCC(=O)NC(Cc3ccccc3)C(=O)NC(C(C)C)C(=O)O
COc1ccc2cc(OCC(=O)NC(Cc3ccccc3)C(=O)O)ccc2c1

CC(C)CC(NC(=O)C(Cc1ccccc1)NC(=O)COc2cc(Cl)c3ccccc3c2)C(=O)O
CCC(C)C(NC(=O)COc1ccc2CCCCc2c1)C(=O)NC(Cc3ccccc3)C(=O)O
CCC(C)C(NC(=O)COc1cc2ccccc2cc1OC)C(=O)NC(Cc3ccccc3)C(=O)O
CCCCCCCCCCCCCCCC(=O)NC(Cc1ccccc1)C(=O)NC(C(C)CC)C(=O)O
CC(NC(=O)COc1cccc2CCCCc12)C(=O)NC(Cc3c(F)c(F)c(F)c(F)c3F)C(=O)O
CCC(C)C(NC(=O)C(Cc1ccccc1)NC(=O)COc2ccc3cc(ccc3c2)C#N)C(=O)O
CC(NC(=O)COc1ccc2ccccc2c1)C(=O)NC(Cc3ccccc3)C(=O)O
CC(C)C(NC(=O)C(Cc1ccccc1)NC(=O)COc2cc(Cl)c3ccccc3c2)C(=O)O
CC(NC(=O)COc1ccc(Cl)c2ccccc12)C(=O)NC(Cc3c(F)c(F)c(F)c(F)c3F)C(=O)O
COc1ccc2cc(OCC(=O)NC(Cc3c(F)c(F)c(F)c(F)c3F)C(=O)O)ccc2c1
CCC(C)C(NC(=O)Cn1c2ccccc2c3ccccc13)C(=O)NC(C(C)C)C(=O)O
COc1ccc2ccc(OCC(=O)NC(CC(C)C)C(=O)NC(Cc3ccccc3)C(=O)O)cc2c1
CC(C)C(NC(=O)Cn1c2ccccc2c3ccccc13)C(=O)O
CCC(C)C(NC(=O)C(Cc1ccccc1)NC(=O)COc2ccc3cc(OC)ccc3c2)C(=O)O
CC(NC(=O)C(C)NC(=O)COc1ccc2CCCCc2c1)C(=O)O
CC(NC(=O)COc1ccc2ccccc2c1)C(=O)NC(Cc3c(F)c(F)c(F)c(F)c3F)C(=O)O
CCCCCCCCCCCCCC(=O)NC(CC(C)C)C(=O)NC(Cc1ccccc1)C(=O)O
CC(NC(=O)COc1ccc2CCCCc2c1)C(=O)NC(Cc3c(F)c(F)c(F)c(F)c3F)C(=O)O
COc1ccc2cc(OCC(=O)NC(Cc3ccccc3)C(=O)NCC(=O)O)ccc2c1
COc1ccc2cc(OCC(=O)NC(C)C(=O)O)ccc2c1
COc1ccc2cc(OCC(=O)NC(C(C)C)C(=O)NC(Cc3ccccc3)C(=O)O)ccc2c1
OC(=O)CNC(=O)C(Cc1ccccc1)NC(=O)COc2ccc3cc(ccc3c2)C#N
COc1cc2ccccc2cc1OCC(=O)NC(C)C(=O)NC(Cc3ccccc3)C(=O)O
CC(NC(=O)COc1ccc2cc(ccc2c1)C#N)C(=O)O
COc1cc2ccccc2cc1OCC(=O)NC(C)C(=O)NC(Cc3c(F)c(F)c(F)c(F)c3F)C(=O)O
CC(C)C(NC(=O)COc1ccc2cc(ccc2c1)C#N)C(=O)NC(Cc3ccccc3)C(=O)O
COc1ccc2ccc(OCC(=O)NC(C)C(=O)NC(C(C)C)C(=O)O)cc2c1
CC(NC(=O)C(Cc1ccccc1)NC(=O)COc2cccc3cc(ccc23)C#N)C(=O)O
CC(C)CC(NC(=O)COc1ccc2cc(ccc2c1)C#N)C(=O)NC(Cc3ccccc3)C(=O)O
CC(C)CC(NC(=O)C(C)NC(=O)Cn1c2ccccc2c3ccccc13)C(=O)O
CCCCCCCCCCCCCCCC(=O)NC(CC(C)C)C(=O)NC(Cc1ccccc1)C(=O)O
CC(NC(=O)COc1cc(Cl)c2ccccc2c1)C(=O)NC(Cc3c(F)c(F)c(F)c(F)c3F)C(=O)O
OC(=O)C(Cc1ccccc1)NC(=O)COc2ccc3cc(ccc3c2)C#N
COc1ccc2cc(OCC(=O)NC(C(C)C)C(=O)NC(Cc3c(F)c(F)c(F)c(F)c3F)C(=O)O)ccc2c1
COc1ccc2cc(OCC(=O)NC(CC(C)C)C(=O)NC(Cc3ccccc3)C(=O)O)ccc2c1
CC(C)C(NC(=O)COc1ccc2cc(ccc2c1)C#N)C(=O)NC(Cc3c(F)c(F)c(F)c(F)c3F)C(=O)O
OC(=O)C(Cc1c(F)c(F)c(F)c(F)c1F)NC(=O)COc2ccc3cc(ccc3c2)C#N
CCC(C)C(NC(=O)C(C)NC(=O)Cn1c2ccccc2c3ccccc13)C(=O)O
CCCCCCCCCCCCCC(=O)NC(Cc1ccccc1)C(=O)NC(C(C)C)C(=O)O
OC(=O)CNC(=O)C(Cc1ccccc1)NC(=O)COc2ccc3cc(Br)ccc3c2
CC(NC(=O)C(Cc1ccccc1)NC(=O)COc2cccc3CCCCc23)C(=O)O
CCCCCCCCCCCCCC(=O)NC(Cc1ccccc1)C(=O)NC(CC(C)C)C(=O)O
COc1ccc2ccc(OCC(=O)NC(Cc3ccccc3)C(=O)NC(CC(C)C)C(=O)O)cc2c1
CC(C)C(NC(=O)Cn1c2ccccc2c3ccccc13)C(=O)NC(C)C(=O)O

CCC(C)C(NC(=O)C(Cc1ccccc1)NC(=O)COc2ccc3cc(Br)ccc3c2)C(=O)O
OC(=O)C(Cc1ccccc1)NC(=O)COc2ccc3cc(Br)ccc3c2
OC(=O)C(Cc1c(F)c(F)c(F)c(F)c1F)NC(=O)COc2ccc3cc(Br)ccc3c2
CC(NC(=O)C(Cc1ccccc1)NC(=O)COc2ccccc2)C(=O)O
COc1ccc2ccc(OCC(=O)NC(Cc3ccccc3)C(=O)NC(C(C)C)C(=O)O)cc2c1
CC(NC(=O)C(Cc1ccccc1)NC(=O)COc2cccc3cc(Br)ccc23)C(=O)O
CCCCCCCCCCCCCCCC(=O)NC(Cc1ccccc1)C(=O)NC(C(C)C)C(=O)O
CCCCCCCCCCCCCC(=O)NC(C(C)CC)C(=O)NC(Cc1ccccc1)C(=O)O
CC(NC(=O)COc1ccc2cc(Br)ccc2c1)C(=O)O
CCCCCCCCCCCCCCCC(=O)NC(Cc1ccccc1)C(=O)NC(CC(C)C)C(=O)O
CCC(C)C(NC(=O)COc1ccc2ccccc2c1)C(=O)NC(Cc3c(F)c(F)c(F)c(F)c3F)C(=O)O
CC(NC(=O)C(Cc1ccccc1)NC(=O)COc2ccc3CCCCc3c2)C(=O)O
CCC(C)C(NC(=O)Cn1c2ccccc2c3ccccc13)C(=O)NC(C)C(=O)O
CC(C)CC(NC(=O)COc1ccc2cc(Br)ccc2c1)C(=O)NC(Cc3ccccc3)C(=O)O
COc1ccc2ccc(OCC(=O)NC(C)C(=O)NC(Cc3ccccc3)C(=O)O)cc2c1
CC(C)C(NC(=O)COc1ccc2cc(Br)ccc2c1)C(=O)NC(Cc3ccccc3)C(=O)O
CC(C)CC(NC(=O)C(Cc1ccccc1)NC(=O)COc2ccc3cc(ccc3c2)C#N)C(=O)O
CCCCCCCCCCCCCC(=O)NC(C)C(=O)NC(Cc1c(F)c(F)c(F)c(F)c1F)C(=O)O
COc1ccc2cc(OCC(=O)NC(Cc3ccccc3)C(=O)NC(CC(C)C)C(=O)O)ccc2c1
CCCCCCCCCCCCCCCC(=O)NC(C(C)CC)C(=O)NC(Cc1ccccc1)C(=O)O
CCC(C)C(NC(=O)COc1ccc2ccccc2c1)C(=O)NC(Cc3ccccc3)C(=O)O
CC(NC(=O)C(Cc1ccccc1)NC(=O)COc2ccc3ccccc3c2)C(=O)O
CC(NC(=O)COc1ccc2cc(Br)ccc2c1)C(=O)NC(Cc3ccccc3)C(=O)O
CCCCCCCCCCCCCCCC(=O)NC(C)C(=O)NC(Cc1c(F)c(F)c(F)c(F)c1F)C(=O)O
CC(NC(=O)C(Cc1ccccc1)NC(=O)COc2ccc(Cl)c3ccccc23)C(=O)O
CC(NC(=O)COc1ccc2cc(ccc2c1)C#N)C(=O)NC(Cc3ccccc3)C(=O)O
CCCCCCCCCCCCCC(=O)NC(C)C(=O)NC(Cc1ccccc1)C(=O)O
COc1ccc2cc(OCC(=O)NC(C)C(=O)NC(C(C)C)C(=O)O)ccc2c1
COc1ccc2cc(OCC(=O)NC(C)C(=O)NC(Cc3ccccc3)C(=O)O)ccc2c1
COc1ccc2cc(OCC(=O)NC(Cc3ccccc3)C(=O)NC(C(C)C)C(=O)O)ccc2c1
CCCCCCCCCCCCCCCC(=O)NC(C)C(=O)NC(Cc1ccccc1)C(=O)O
CC(NC(=O)C(Cc1ccccc1)NC(=O)COc2cc(Cl)c3ccccc3c2)C(=O)O
COc1ccc2ccc(OCC(=O)NC(C)C(=O)NC(Cc3c(F)c(F)c(F)c(F)c3F)C(=O)O)cc2c1
COc1cc2ccccc2cc1OCC(=O)NC(Cc3ccccc3)C(=O)NC(C)C(=O)O
CC(C)C(NC(=O)C(Cc1ccccc1)NC(=O)COc2ccc3cc(ccc3c2)C#N)C(=O)O
CC(C)C(NC(=O)C(C)NC(=O)COc1ccc2cc(ccc2c1)C#N)C(=O)O
CC(C)CC(NC(=O)C(Cc1ccccc1)NC(=O)COc2ccc3cc(Br)ccc3c2)C(=O)O
CCC(C)C(NC(=O)COc1ccc2ccc(OC)cc2c1)C(=O)NC(Cc3ccccc3)C(=O)O
CC(C)C(NC(=O)Cn1c2ccccc2c3ccccc13)C(=O)NC(Cc4c(F)c(F)c(F)c(F)c4F)C(=O)O
CCC(C)C(NC(=O)C(Cc1ccccc1)NC(=O)Cn2c3ccccc3c4ccccc24)C(=O)O
CC(C)C(NC(=O)Cn1c2ccccc2c3ccccc13)C(=O)NC(Cc4ccccc4)C(=O)O
CC(NC(=O)COc1ccc2cc(ccc2c1)C#N)C(=O)NC(Cc3c(F)c(F)c(F)c(F)c3F)C(=O)O
COc1ccc2cc(OCC(=O)NC(C)C(=O)NC(Cc3c(F)c(F)c(F)c(F)c3F)C(=O)O)ccc2c1
CC(C)CC(NC(=O)Cn1c2ccccc2c3ccccc13)C(=O)NC(Cc4ccccc4)C(=O)O

COc1ccc2ccc(OCC(=O)NC(Cc3ccccc3)C(=O)NC(C)C(=O)O)cc2c1
OC(=O)CNC(=O)C(Cc1ccccc1)NC(=O)Cn2c3ccccc3c4ccccc24
CCCCCCCCCCCCCC(=O)NC(Cc1ccccc1)C(=O)NC(C)C(=O)O
CCC(C)C(NC(=O)COc1ccc2cc(OC)ccc2c1)C(=O)NC(Cc3ccccc3)C(=O)O
CC(C)C(NC(=O)C(Cc1ccccc1)NC(=O)COc2ccc3cc(Br)ccc3c2)C(=O)O
CCC(C)C(NC(=O)COc1ccc2cc(ccc2c1)C#N)C(=O)NC(Cc3ccccc3)C(=O)O
CCCCCCCCCCCCCCCC(=O)NC(Cc1ccccc1)C(=O)NC(C)C(=O)O
CC(NC(=O)C(Cc1ccccc1)NC(=O)COc2ccc3cc(ccc3c2)C#N)C(=O)O
COc1ccc2cc(OCC(=O)NC(Cc3ccccc3)C(=O)NC(C)C(=O)O)ccc2c1
CC(C)C(NC(=O)C(C)NC(=O)COc1ccc2cc(Br)ccc2c1)C(=O)O
OC(=O)C(Cc1c(F)c(F)c(F)c(F)c1F)NC(=O)Cn2c3ccccc3c4ccccc24
CC(C)CC(NC(=O)C(Cc1ccccc1)NC(=O)Cn2c3ccccc3c4ccccc24)C(=O)O
OC(=O)C(Cc1ccccc1)NC(=O)Cn2c3ccccc3c4ccccc24
CC(NC(=O)Cn1c2ccccc2c3ccccc13)C(=O)O
CC(NC(=O)COc1ccc2cc(Br)ccc2c1)C(=O)NCC(=O)O
CC(NC(=O)C(Cc1ccccc1)NC(=O)COc2ccc3cc(Br)ccc3c2)C(=O)O
CCC(C)C(NC(=O)Cn1c2ccccc2c3ccccc13)C(=O)NC(Cc4ccccc4)C(=O)O
CC(NC(=O)Cn1c2ccccc2c3ccccc13)C(=O)NC(Cc4ccccc4)C(=O)O
CC(C)C(NC(=O)C(C)NC(=O)Cn1c2ccccc2c3ccccc13)C(=O)O
CC(C)C(NC(=O)C(Cc1ccccc1)NC(=O)Cn2c3ccccc3c4ccccc24)C(=O)O
CCC(C)C(NC(=O)COc1ccc2cc(Br)ccc2c1)C(=O)NC(Cc3ccccc3)C(=O)O
CC(NC(=O)Cn1c2ccccc2c3ccccc13)C(=O)NC(Cc4c(F)c(F)c(F)c(F)c4F)C(=O)O
CC(NC(=O)C(C)NC(=O)COc1ccc2cc(Br)ccc2c1)C(=O)O
CCCCCCCCCCCCCC(=O)NC(Cc1ccccc1)C(=O)NC(Cc2ccccc2)C(=O)O
CCCCCCCCCCCCCCCC(=O)NC(Cc1ccccc1)C(=O)NC(Cc2ccccc2)C(=O)O
CC(NC(=O)C(Cc1ccccc1)NC(=O)Cn2c3ccccc3c4ccccc24)C(=O)O
OC(=O)C(Cc1ccccc1)NC(=O)C(Cc2ccccc2)NC(=O)COc3ccccc3
OC(=O)C(Cc1ccccc1)NC(=O)C(Cc2ccccc2)NC(=O)COc3cccc4ccccc34
OC(=O)C(Cc1ccccc1)NC(=O)C(Cc2ccccc2)NC(=O)COc3cccc4CCCCc34
OC(=O)C(Cc1c(F)c(F)c(F)c(F)c1F)NC(=O)C(Cc2ccccc2)NC(=O)COc3ccccc3
OC(=O)C(Cc1ccccc1)NC(=O)C(Cc2ccccc2)NC(=O)COc3cccc4cc(ccc34)C#N
OC(=O)C(Cc1ccccc1)NC(=O)C(Cc2ccccc2)NC(=O)COc3ccc4CCCCc4c3
COc1cc2ccccc2cc1OCC(=O)NC(Cc3ccccc3)C(=O)NC(Cc4ccccc4)C(=O)O
OC(=O)C(Cc1ccccc1)NC(=O)C(Cc2ccccc2)NC(=O)COc3cccc4cc(Br)ccc34
COc1ccc2ccc(OCC(=O)NC(Cc3ccccc3)C(=O)NC(Cc4ccccc4)C(=O)O)cc2c1
OC(=O)C(Cc1ccccc1)NC(=O)C(Cc2ccccc2)NC(=O)COc3ccc(Cl)c4ccccc34
OC(=O)C(Cc1ccccc1)NC(=O)C(Cc2ccccc2)NC(=O)COc3ccc4ccccc4c3
OC(=O)C(Cc1ccccc1)NC(=O)C(Cc2ccccc2)NC(=O)COc3cc(Cl)c4ccccc4c3
COc1ccc2cc(OCC(=O)NC(Cc3ccccc3)C(=O)NC(Cc4ccccc4)C(=O)O)ccc2c1
OC(=O)C(Cc1ccccc1)NC(=O)C(Cc2ccccc2)NC(=O)COc3ccc4cc(ccc4c3)C#N
OC(=O)C(Cc1ccccc1)NC(=O)C(Cc2ccccc2)NC(=O)COc3ccc4cc(Br)ccc4c3
OC(=O)C(Cc1ccccc1)NC(=O)C(Cc2ccccc2)NC(=O)Cn3c4ccccc4c5ccccc35

Table S5 List of compound codes and SMILES codes. The codes in the right hand column can be used to request predictions from the authors

References

(1) Chen, L.; Revel, S.; Morris, K.; Serpell, L. C.; Adams, D. J. *Langmuir* **2010**, *26*, 13466.

(2) Chen, L.; Morris, K.; Laybourn, A.; Elias, D.; Hicks, M. R.; Rodger, A.; Serpell, L.; Adams, D. J. *Langmuir* **2010**, *26*, 5232.

(3) Houton, K. A.; Morris, K. L.; Chen, L.; Schmidtmann, M.; Jones, J. T. A.; Serpell, L. C.; Lloyd, G. O.; Adams, D. J. *Langmuir* **2012**, *28*, 9797.

(4) Raeburn, J.; McDonald, T. O.; Adams, D. J. *Chemical Communications* **2012**, *48*, 9355.

(5) Chen, L.; McDonald, T. O.; Adams, D. J. *RSC Advances* **2013**, *3*, 8714.

(6) Colquhoun, C.; Draper, E. R.; Eden, E. G. B.; Cattoz, B. N.; Morris, K. L.; Chen, L.; McDonald, T. O.; Terry, A. E.; Griffiths, P. C.; Serpell, L. C.; Adams, D. J. *Nanoscale* **2014**, *6*, 13719.

(7) Awhida, S.; Draper, E. R.; McDonald, T. O.; Adams, D. J. *Journal of Colloid and Interface Science* **2015**, *455*, 24.

(8) Draper, E. R.; McDonald, T. O.; Adams, D. J. *Chemical Communications* **2015**, *51*, 12827.

(9) Adams, D. J.; Young, I. *Journal of Polymer Science Part A: Polymer Chemistry* **2008**, *46*, 6082.

(10) http://www.cambridgesoft.com/software/overview.aspx.

(11) http://accelrys.com/products/collaborative-science/biovia-pipeline-pilot/.

(12) Rogers, D.; Hahn, M. *Journal of Chemical Information and Modeling* **2010**, *50*, 742.

(13) Team, R. C.; R Foundation for Statistical Computing: 2015.

(14) Venables, W. N.; Ripley, B. D. *Modern Applied Statistics with S. Fourth Edition.*; Springer, 2002.

(15) Kuhn, M.; Wing, J.; Weston, S.; Williams, A.; Keefer, C.; Engelhardt, A.; Cooper, T.; Mayer, Z.; Kenkel, B.; Team, R. C.; Benesty, M.; Lescarbeau, R.; Ziem, A.; Scrucca, L. 2015.

(16) M. Leite, D.; Barbu, E.; J. Pilkington, G.; Lalatsa, A. *Current Topics in Medicinal Chemistry* **2015**, *15*, 2277.

(17) Osolodkin, D. I.; Radchenko, E. V.; Orlov, A. A.; Voronkov, A. E.; Palyulin, V. A.; Zefirov, N. S. *Expert Opinion on Drug Discovery* **2015**, *10*, 959.

(18) Lipinski, C. A. *Drug Discovery Today: Technologies* **2004**, *1*, 337.

(19) Nell, P. G.; Mundt, S. M. *High-Throughput Screening in Drug Discovery, Chapter 8*; Wiley, 2006; Vol. 35.
